# Photooxidative Molecular Rearrangement of Vouacapane Diterpenes Generates Proapoptotic Spirocaesalmins

**DOI:** 10.1021/acsomega.6c03239

**Published:** 2026-08-13

**Authors:** Laura Hernández-Padilla, Leonardo R. Álvarez-García, Eva E. Soto-Guzmán, Gabriela Rodríguez-García, Edna M. Silva-García, Lorena Martínez-Alcántar, Jesús Campos-García, Christine Thomassigny, Rosa E. del Río, Armando Talavera-Alemán, Mario A. Gómez-Hurtado, Carlos M. Cerda-García-Rojas

**Affiliations:** † Instituto de Investigaciones Químico Biológicas, Universidad Michoacana de San Nicolás de Hidalgo, Ciudad Universitaria, Morelia, Michoacán 58030, Mexico; ‡ Universidad La Salle, Av. Universidad 500, Cto. Erandeni I, Tarímbaro, Michoacán 58880, Mexico; § LV-UMR CNRS 8180, Université de Versailles St-Quentin-en-Yvelines, 45 Avenue des États-Unis, 78035 Versailles, France; ∥ Departamento de Química, Centro de Investigación y de Estudios Avanzados del Instituto Politécnico Nacional, Apartado 14-740, Mexico City 07000, Mexico

## Abstract

The photooxidative reactivity of vouacapane derivatives and their potential as apoptotic inducers are explored in the present research. We report the obtention of the new diastereomeric spiroepoxylactones (+)-(5*S*,6*R*,​8*S*,9*S*,​10*R*,13*S*​,14*R*,15*R*,​16*S*)-15,16-epoxy-6-hydroxy-13-spirocassan-12,16-olide (**2a**), (−)-(5*S*,​6*R*,8*S*,​9*S*,10*R*,​13*R*,14*R*,​15*S*,16*R*)-15,16-epoxy-6-hydroxy-13-spirocassan-12,16-olide (**2b**), (+)-(5*S*,​8*S*,9*S*,​10*R*,13*S*,​14*R*,15*R*,​16*S*)-15,16-epoxy-6-oxo-13-spirocassan-12,16-olide (**3a**), (−)-(5*S*,​8*S*,9*S*,​10*R*,13*R*,​14*R*,15*S*,​16*R*)-15,16-epoxy-6-oxo-13-spirocassan-12,16-olide (**3b**), (+)-(5*R*,​6*R*,7*R*,​8*S*,9*S*,​10*R*,13*S*,​14*R*,15*R*,​16*S*)-6-benzoyloxy-15,16-epoxy-5,7-dihydroxy-13-spirocassan-12,16-olide (**4a**), and (−)-(5*R*,​6*R*,7*R*,​8*S*,9*S*,​10*R*,13*R*,​14*R*,15*S*,​16*R*)-6-benzoyloxy-15,16-epoxy-5,7-dihydroxy-13-spirocassan-12,16-olide (**4b**) by photooxidation of 6β-hydroxyvouacapane (**2**), 6-oxovouacapane (**3**), and isovouacapenol C (**4**), respectively. Known spirolactones (+)-(5*S*,6*R*,8*S*,9*S*,10*R*,13*S*,14*R*,15*R*,16*S*)-6-acetoxy-15,16-epoxy-13-spirocassan-12,16-olide (**1a**) and (−)-(5*S*,6*R*,8*S*,9*S*,10*R*,13*R*,14*R*,15*S*,16*R*)-6-acetoxy-15,16-epoxy-13-spirocassan-12,16-olide (**1b**) were also obtained from 6β-acetoxyvouacapane (**1**). All compounds were characterized by their physical and spectroscopic properties, while their absolute configurations were established by single-crystal X-ray analysis of **2a** and **3a**, and computational calculations of their specific rotation values. The cytotoxicity and apoptosis induction potential of the compounds were determined in vitro using HeLa and MDA-MB-231 human cancer cell lines, in comparison with CCD-18Co normal cell lines. The results showed that rearranged compounds **1b**, **2a**, and **4a** had the highest cytotoxic activity against HeLa cells, while for MDA-MB-231 cells, the best results were obtained with derivatives **1b**, **2a**, and **3b**.

## Graphical abstract

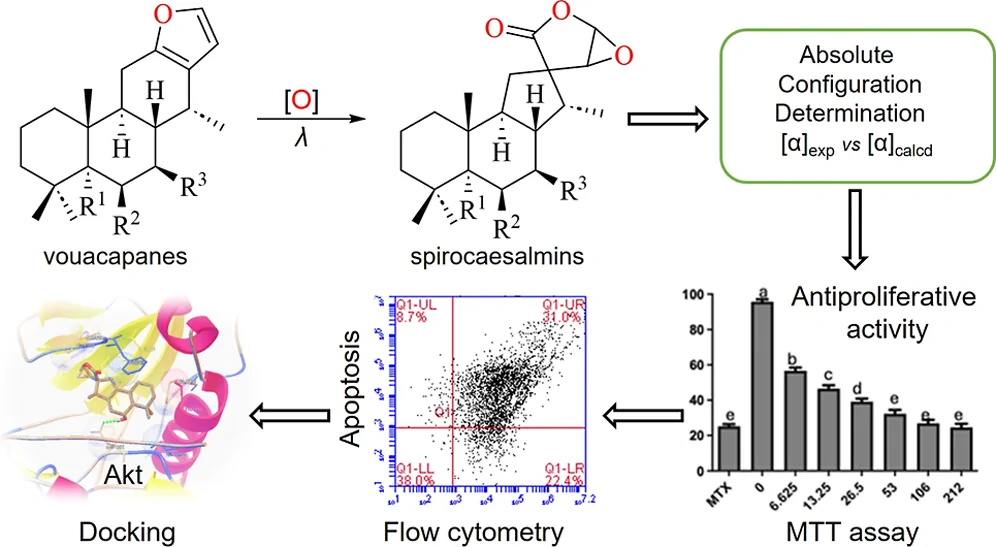

## 1 Introduction

Molecular transformations of natural products have provided a vast source of new compounds for developing advanced chemical protocols to treat cancer. Systematic structural modifications of this class of substances have notably expanded their therapeutic potential in terms of bioactivity and selectivity.^1^ Nowadays, the most common types of cancer that require chemotherapy include leukemia, lymphoma, multiple myeloma, central nervous system cancers, lung, prostate, colorectal, stomach, liver, thyroid, cervical, and breast cancers.^2^ In particular, cervical and breast cancers account for significant mortality among women worldwide.^3–5^ Current treatments for these types of cancers involve surgery, radiotherapy, and chemotherapy as adjuvant therapy to improve outcomes.^6,7^ Since chemotherapy causes multiple undesirable side effects,^8,9^ scientific research aimed at discovering new chemotherapeutic agents remains pertinent, and research on natural products, including their chemical modifications, is a promising strategy. In particular, the biological potential of spirolactones derived from vouacapanes for these purposes has been scarcely explored. In this context, in vitro assays are a strategic tool for discovering new active principles. On this basis, HeLa cells, the first continuous cancer cell line, have been a cornerstone of cancer research since their isolation over 50 years ago from an aggressive glandular cervical cancer in a young woman. This cell line has contributed to understanding nearly every process in human cells.^10,11^ By itself, the MDA-MB-231, a highly aggressive, invasive, and poorly differentiated triple-negative breast cancer (TNBC) cell line isolated at the MD Anderson Cancer Center from the pleural effusion of a patient with invasive ductal carcinoma, is widely used as a model for late-stage breast cancer.^12^

Structural modification of natural products with confirmed cytotoxicity constitutes a valuable alternative approach to research on the discovery of new anticancer compounds.^13,14^ Among natural products with potential anticancer activity include the cassane diterpenoids, particularly those of the vouacapane type,^15,16^ whose structural skeleton is based on a methylated perhydrophenantrene core fused to a furan moiety. These compounds, commonly isolated from Fabaceae species,^17^ possess an interesting chemical versatility, providing diverse structural modifications, including molecular transpositions,^18^ oxidative degradation,^19,20^ dimerization,^21,22^ aromatization of ring C,^22,23^ ring C contraction,^20,22,24^ furan ring opening,^21,22^ and lactonization.^19,20^ These modifications could promote new or improved biological activities.

This work aimed to conduct oxidative modifications on the natural 6β-acetoxyvouacapane (**1**) from *Coulteria platyloba* (S.Watson) N.Zamora (basionym: *Caesalpinia platyloba* S.Watson),^25^ and its described derivatives 6β-hydroxyvouacapane (**2**), and 6-oxovouacapane (**3**),^25^ as well as the known isovouacapenol C (**4**) from *Caesalpinia pulcherrima* L. Sw^26,27^ to provide the respective epoxyspirolactone derivatives (+)-(5*S*,6*R*,8*S*,9*S*,10*R*,13*S*,14*R*,15*R*,16*S*)-15,16-epoxy-6-hydroxy-13-spirocassan-12,16-olide (**2a**), (−)-(5*S*,​6*R*,8*S*,​9*S*,10*R*,​13*R*,14*R*,​15*S*,​16*R*)-15,16-epoxy-6-hydroxy-13-spirocassan-12,16-olide (**2b**), (+)-(5*S*,8*S*,​9*S*,10*R*,​13*S*,14*R*,​15*R*,16*S*)-15,16-epoxy-6-oxo-13-spirocassan-12,16-olide (**3a**), (−)-(5*S*,​8*S*,9*S*,​10*R*,13*R*,​14*R*,15*S*,​16*R*)-15,16-epoxy-6-oxo-13-spirocassan-12,16-olide (**3b**), (+)-(5*R*,​6*R*,7*R*,​8*S*,9*S*,​10*R*,13*S*,​14*R*,15*R*,​16*S*)-6-benzoyloxy-15,16-epoxy-5,7-dihydroxy-13-spirocassan-12,16-olide (**4a**), and (−)-(5*R*,​6*R*,7*R*,​8*S*,9*S*,​10*R*,13*R*,​14*R*,15*S*,​16*R*)-6-benzoyloxy-15,16-epoxy-5,7-dihydroxy-13-spirocassan-12,16-olide (**4b**). All new compounds were characterized by their physical and spectroscopic data, and their absolute configuration was established by single-crystal X-ray diffraction, NOESY experiments, and supported with [α]_D_ calculations with the DFT B3LYP ccp-VDZ and DGDZVP level of theory. Antiproliferative activities of the oxidized compounds (**1a–4b**) and their precursors (**1–4**) were evaluated using the MTT method with HeLa and MDA-MB-231 cell lines, in comparison with healthy CCD-18Co cell lines. The potential of the antiproliferative compounds to induce apoptosis was assessed by flow cytometry. Molecular docking studies provided an approach to understanding the structure–activity relationships of the bioactive derivatives. These experimental strategies may enhance the biological potential of organic molecules, including natural products, as is the case for vouacapane diterpenes.

## 2 Results and Discussion

### 2.1 Photochemistry and Spectroscopy

Diastereomeric epoxyspirolactones can be obtained by photooxidation of 6β-acetoxyvouacapane (**1**) from *C. platyloba*, as it was previously discovered using a simplistic methodology, allowing the formation of diastereomers **1a** and **1b**, whose absolute configuration was determined by single-crystal X-ray diffraction means.^24^ The conformational restrictions and variety of moieties in these compounds could confer interesting biological potential, particularly those against cancer, as described for several cassane-type derivatives.^15^ Additionally, the structural properties, together with the well-known configurational status of the natural starting material,^24,25^ allow for a simplistic and confident configurational analysis, as NOESY experiments, thus providing complete structural elucidation. Thus, preparation of new epoxyspirolactone derivatives (**2a–4a** and **2b–4b**) was achieved from vouacapanes **2** and **3**, which were obtained in turn from **1** isolated from *C. platyloba*, as well as from isovouacapenol C (**4**) isolated from *C. pulcherrima* (Figure 1). The physical and spectroscopic data of compounds **2–4** agreed with the previous reports (see experimental section and Supporting Information, Figures S2–S4).^25–27^

**Figure 1:**
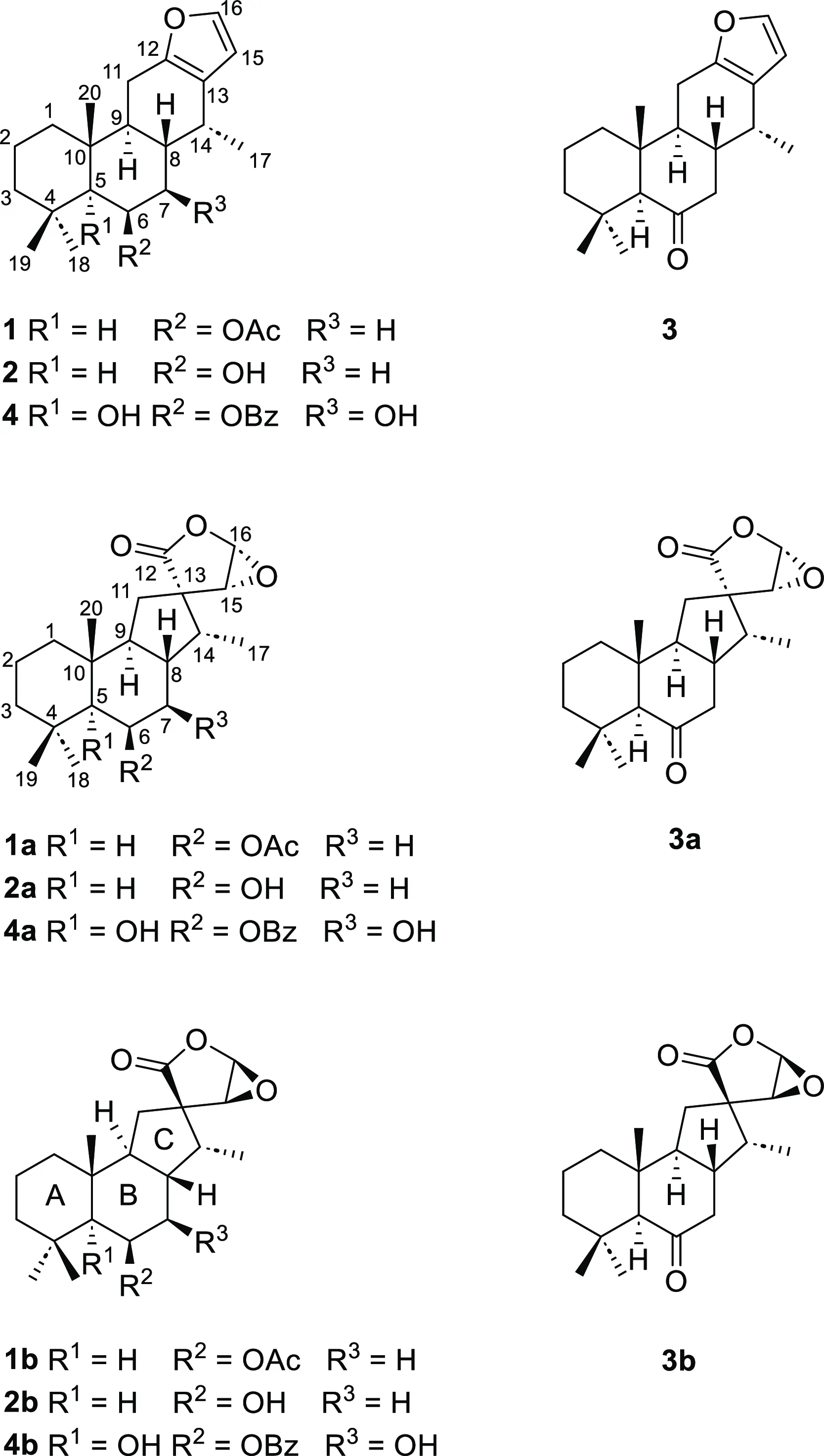
Formulas of vouacapane **1–4** and their epoxyspirolactone derivatives **1a**-**4a** and **1b**-**4b**.

Photooxidation of vouacapane **2** yielded two products, which were purified by chromatography. The first compound (**2a**) was isolated as a white solid (mp 193–196 °C) in 12% yield, whose HRESIMS showed the [M + H]^+^ ion at *m*/*z* 335.2225 (calcd for C_20_H_30_O_4_+H^+^ 335.2222). A dextrorotatory value of [α]_D_ +55 (*c* 0.5, CHCl_3_) was observed. The IR spectrum revealed a vibrational band at 1770 cm^–1^, attributed to the lactone motif.^28,29^ Its ^1^H NMR displayed two doublets at δ 5.54 and 3.61 (*J* = 2.3 Hz) associated with the H-16 and H-15, respectively from an epoxide moiety.^28,30^ The resonance of H-14 appeared as a quintet at δ 2.64 (*J* = 7.5 Hz) while the signals for CH_2_–11 wereobserved as doublet of doublets at δ 1.94 (H-11β *J* = 13.3, 8.3 Hz) and δ 1.55 (H-11α dd, *J* = 13.3, 10.2 Hz), suggesting the contraction of ring C from the cassane skeleton.^24,30^ In the ^13^C NMR spectrum, the signal at δ 178.5 (C-12) was observed and assigned to the lactone motif, while the signals at δ 78.2 and 60.0 confirmed the presence of the epoxide moiety. The signal at δ 54.8 (C-13) indicated C-ring contraction.^28–30^ The unequivocal assignment of these carbons was supported by an HMBC experiment, where correlations of C-12 with hydrogens H-16, H-15, H-14, and CH_2_–11 were observed. Similarly, C-13 revealed correlations with H-15, H-14, H-8, CH_2_–11, and CH_3_-17. The complete analysis of ^1^H and ^13^C NMR spectra (Tables 1 and 2) of this compound (see Figures S11 and S14) revealed that rings A and B from the diterpene skeleton remained without structural modifications. Their 1D NMR assignments were supported by 2D NMR experiments (see Figures S12, S13, S15, and S16). The absolute configuration of compound **2a** was concluded from that of its precursor (**2**)^25^ and from NOESY experiments for the newly created asymmetric carbon atoms C-13, C-15, and C-16, as H-14 correlated with H-8 and H-15. In addition, the ^13^C chemical shifts for C-12 and C-15 were similar to those recorded for **1a**. In consequence, the structure and full NMR assignment of (+)-(5*S*,6*R*,8*S*,9*S*,10*R*,13*S*,14*R*,15*R*,16*S*)-15,16-epoxy-6-hydroxy-13-spirocassan-12,16-olide (**2a**) were determined. The next compound isolated from the column chromatography was **2b** as a white solid (mp 187–189 °C), also in 12% yield. Its IR spectrum revealed a vibrational band at 1787 cm^–1^, while a levorotatory value of [α]_D_ −47 (*c* 0.58, CHCl_3_) was found. The HRMS showed the [M + H]^+^ ion at *m*/*z* 335.2272 (calcd for C_20_H_30_O_4_+H^+^ 335.2222). The ^1^H NMR spectrum (see Figure S19) revealed a similar signal pattern to that of **2a**. However, significant variations in the chemical shifts of H-11β (δ 1.82 dd, *J* = 13.5, 11.2 Hz), H-11α (δ 1.70 dd, *J* = 13.5, 7.9 Hz), H-14 (δ 2.37 quint, *J* = 7.4 Hz), and CH_3_-17 (δ 1.06 d, *J* = 7.4 Hz) were observed. The ^13^C NMR spectrum (Figure S20) also supported the formation of the spirolactone epoxide derivative **2b** since the signals attributed to C-12 and C-15 appeared at δ 180.8 and 57.6, respectively. The complete assignment of its 1D NMR spectra (Tables 1 and 2) was supported by 2D NMR experiments (see Figures S21 and S22). The absolute configuration of compound **2b** was established by its comparison with **2a** due to their epimeric correlation. Thereby, compound **2b** was named as (−)-(5*S*,6*R*,8*S*,9*S*,10*R*,13*R*,14*R*,15*S*,16*R*)-15,16-epoxy-6-hydroxy-13-spirocassan-12,16-olide.

**Table 1:** ^1^H NMR Data for Compounds **2a–4a** and **2b–4b** (300 MHz, CDCl_3_)­^a^

H	**2a**	**2b**	**3a**	**3b**	**4a**	**4b**
1β	1.41, m	1.41, m	1.46, m	1.46, m	1.68, m	1.69, m
1α	1.11, m	1.05, m	1.20, m	1.19, m	1.23, m	1.23, m
2β	1.65, m	1.65, m	1.60, m	1.60, m	1.73, m	1.73, m
2α	1.44, m	1.40, m	1.48, m	1.48, m	1.51, m	1.51, m
3β	1.40, m	1.41, m	1.35, brd (13.2)	1.37, brd (13.4)	1.73, m	1.73, m
3α	1.21, m	1.21, m	1.10, td (13.7, 4.4)	1.15, m	1.18, m	1.21, m
5	0.89, d (2.0)	0.85, d (2.1)	2.07, s	2.01, s		
6	4.50, s	4.48, s			5.73, d (3.9)	5.73, d (3.9)
7β	1.81, m	1.83, m	2.28, m	2.31, m	4.39, dd (11.0, 4.10)	4.35, brs
7α	1.48, m	1.47, m				
8	2.39, m	2.64, m	2.28, m	2.36, m	2.26, ddd (13.3, 11.2, 7.3)	2.59, m
9	1.77, m	1.50, m	2.28, m	2.11, ddd (13.0, 10.8, 8.4)	2.77, m	2.59, m
11β	1.94, dd (13.3, 8.3)	1.82, dd (13.5, 11.2)	1.98, dd (13.3, 7.1)	1.77, m	1.95, dd (13.3, 8.4)	1.89, m
11α	1.55, dd (13.3, 10.2)	1.70, dd (13.5, 7.9)	1.48, m		1.61, m	1.69, m
14	2.64, quint (7.5)	2.37, quint (7.4)	2.67, quint, (7.4)	2.61, m	2.89, quint, (7.3)	2.59, m
15	3.61, d (2.3)	3.53, d (2.3)	3.59, d (2.3)	3.59, d (2.3)	3.64, d (2.3)	3.58, d (2.3)
16	5.54, d (2.3)	5.56, d (2.3)	5.56, d (2.3)	5.60, d (2.3)	5.56, d (2.3)	5.58, d (2.3)
17	0.94, d (7.5)	1.06, d (7.4)	0.99, d (7.5)	1.15, d (7.4)	1.09, d (7.3)	1.23, d (7.0)
18	0.99, s	0.99, s	0.95, s	0.97, s	1.06, s	1.08, s
19	1.23, s	1.24, s	1.21, s	1.23, s	1.16, s	1.15, s
20	1.21, s	1.20, s	0.84, s	0.86, s	1.49, s	1.47, s
3′, 7′					8.05, brd (7.3)	8.07, d (7.4)
4′, 6′					7.47, t (7.3)	7.60 t (7.4)
5′					7.60, tt (7.3, 1.5)	7.48,t (7.4)

a All assignments were supported by gCOSY, gNOESY, gHSQC, and gHMBC experiments.

**Table 2:** ^13^C NMR Data for Compounds **2a–4a** and **2b–4b** (75.4 MHz, CDCl_3_)­^a^

C	**2a**	**2b**	**3a**	**3b**	**4a**	**4b**
1	42.5	41.9	39.6	40.0	34.7	34.9
2	18.9	18.9	18.3	18.4	18.1	18.0
3	44.4	44.5	43.2	43.2	38.0	38.0
4	34.0	34.0	32.2	32.2	39.2	39.1
5	57.3	57.4	65.5	65.8	79.5	79.6
6	68.3	68.2	211.3	210.8	74.9	74.7
7	37.5	37.6	45.5	45.3	69.5	69.4
8	37.2	37.2	41.2	41.2	44.0	44.3
9	55.7	55.7	54.9	54.5	45.5	45.2
10	37.5	37.5	39.3	39.9	40.5	40.5
11	32.7	33.4	32.4	32.3	32.8	33.0
12	178.5	180.8	178.0	180.4	178.3	180.6
13	54.8	55.5	54.6	55.5	54.2	55.1
14	42.4	41.9	43.0	43.0	40.7	39.9
15	60.0	57.6	59.9	57.3	60.0	57.3
16	78.2	77.8	78.3	77.9	78.3	77.7
17	12.4	12.2	13.0	11.9	12.2	11.4
18	33.9	34.0	32.7	32.8	28.0	27.8
19	24.4	24.2	21.8	21.7	25.3	25.0
20	16.2	16.3	14.8	14.7	16.1	15.8
1′					167.3	170.0
2′					130.0	129.8
3′, 7′					130.0	130.0
4′, 6′					128.8	128.7
5′					133.5	133.4

a All assignments were supported by gHSQC and gHMBC experiments.

Photooxidation of **3** yielded a mixture of two compounds (**3a** and **3b**), which were also isolated by chromatographic means. The derivative **3a** was isolated as a white solid (mp 208–211 °C) in 12% yield. The HRMS revealed a [M + H]^+^ ion at *m*/*z* 333.2059 (calcd for C_20_H_28_O_4_H^+^ 333.2066). The IR spectrum showed two vibrational bands at 1779 and 1701 cm^–1^, attributed to the C=O groups for lactone and ketone, respectively. Its specific rotation was [α]_D_ +54 (*c* 0.65, CHCl_3_). The ^1^H NMR spectrum revealed two doublets at δ 5.56 and 3.59 (*J* = 2.3 Hz), attributed to H-16 and H-15, respectively, confirming the epoxide motif. The resonance pattern involving H-8 (δ 2.28, m), H-14 (δ 2.67, quint, *J* = 7.4 Hz), and CH_3_-17 (0.99, d, *J* = 7.5 Hz) indicates contraction of ring C. The ^13^C NMR spectrum of **3a** showed 20 signals, including the signal for C-12 at δ 178.0, thus confirming the presence of a lactone moiety. The signals at δ 78.3 and 59.9 were attributed to the epoxide moiety, whose 15*R*,16*S* configuration is directly related to that established for compounds **1a** and **2a**, as indicated by the observed chemical shift trend. Contraction of ring C to give **3a** leads to the chemical shift of the C-13 signal appearing at δ 54.6, in agreement with the 13*S* configuration, as similar data were observed in the NMR analysis of **1a** and **2a**. The NOESY experiment confirmed the 13*S*, 15*R*, 16*S* configuration based on the correlations observed between H-8 and H-14, and between H-14 and H-15. According to these results, the obtention of (+)-(5*S*,8*S*,9*S*,10*R*,13*S*,14*R*,15*R*,16*S*)-15,16-epoxy-6-oxo-13-spirocassan-12,16-olide (**3a**) was established. The complete assignment of the ^1^H and ^13^C spectra is presented in Tables 1 and 2 and was supported by 2D NMR experiments (see Figures S25–S29). Purification of **3b** from the crude reaction by column chromatography was unsuccessful in our hands, limiting in part the present photooxidative methodology. Alternatively, the oxidation process of **2b** with pyridinium chlorochromate (PCC) yielded **3b** (70%) as a white solid (mp 223–226 °C) and [α]_D_ −56 (*c* 1.04, CHCl_3_). Thus, this option provided a simplistic solution to easily obtain the expected derivative **3b** in pure form. The HRESIMS revealed a [M + H]^+^ ion at *m*/*z* 333.2058, in concordance with the formula C_20_H_28_O_4_ + H (calcd 333.2060). The IR spectrum showed intense vibrational bands at 1784 and 1702 cm^–1^, attributed to the C=O stretching vibration of the lactone and ketone groups. The ^1^H NMR spectrum exhibited a similar signal pattern to that of **1b** and **2b**. Herein, the signals from H-16 and H-15 appeared at δ 5.60 and 3.59 (d, *J* = 2.3 Hz), respectively, while the signal attributed to CH_3_-17 appeared at δ 1.15 (d, *J* = 7.4 Hz). The ^13^C NMR spectrum showed 20 signals, of which the C-12 signal appeared at δ 180.4, and the spiro-carbon (C-13) was observed at δ 55.5, a chemical shift similar to those of **1b** and **2b**, thereby indicating the 13*R* configuration. Signals assigned to C-15 (δ 57.3) and C-16 (δ 77.9) also show chemical shifts very close to those of analogues **1b** and **2b**, in agreement with the 15*S*, 16*R* configuration. The complete assignment of the ^1^H and ^13^C NMR is listed in Tables 1 and 2 and supported with 2D NMR experiments (see Supporting Information Figures S33, S34, S36, and S37). According to these results the obtention of (−)-(5*S*,8*S*,9*S*,10*R*,13*R*,14*R*,15*S*,16*R*)-15,16-epoxy-6-oxo-13-spirocassan-12,16-olide (**3b**) was established.

The photooxidation process of isovouacapenol C (**4**) yielded derivatives **4a** and **4b** in 11% and 10%, respectively, after column chromatography. Compound **4a** was obtained as a colorless oil, whose IR spectrum revealed a vibrational band at 1786 cm^–1^, confirming the formation of the lactone motif. A dextrorotatory value of [α]_D_ +24 (*c* 0.69, MeOH) was observed, suggesting the obtention of a spirolactone with the 13*S*, 15*R*, 16*S* configuration due to the observed trend of this data when compared with analogues **1a**-**3a** (see Experimental Section). Its HRMS showed the [M + Na]^+^ ion at *m*/*z* 493.2206 (calcd for C_27_H_34_O_7_ + Na 493.2202). The ^1^H NMR showed doublets at δ 5.56 and 3.64 (*J* = 2.3 Hz) related to H-16 and H-15, respectively, evidencing the formation of an epoxide motif. Contraction of ring C was revealed by the resonances of H-14 (δ 2.89 quint, *J* = 7.3 Hz) and H-11β (δ 1.95, dd, *J* = 13.3, 8.4 Hz) and H-11α (δ 1.61 m). The signal pattern in the range of δ 8.05–7.47 confirmed the integrity of the benzoate moiety. The ^13^C NMR showed 25 signals, including those at δ 167.3, 133.5, 130.0 (×2), 130.0, and 128.8 (×2) associated with the benzoyloxy motif^31,32^ (see Table 2). The signals at δ 178.3 (C-12) and 54.2 (C-13) confirmed the formation of a spirolactone system. The signals at δ 78.3 (C-16) and 60.0 (C-15) suggested the presence of an epoxide moiety in the reaction product. The chirality of the new stereogenic centers can be related if [α]_D_ value and NMR results are compared with the analogues **1a–3a**, thereby the obtention of (+)-(5*R*,6*R*,7*R*,8*S*,9*S*,10*R*,13*S*,14*R*,15*R*,16*S*)-6-benzoyloxy-15,16-epoxy-5,7-dihydroxy-13-spirocassan-12,16-olide (**4a**) was established. The complete assignment of ^1^H and ^13^C NMR is presented in Tables 1 and 2 and was supported by 2D NMR experiments (see Figures S41, S42, S44, and S45). Correspondingly, compound **4b** was also isolated as a colorless oil with levorotatory value of [α]_D_ −17 (c 0.83, MeOH). The HRMS analysis showed the [M + H]^+^ ion at *m*/*z* 471.2365 related to the formula C_27_H_34_O_7_ + H^+^ (calcd for 471.2383). The ^1^H NMR revealed the presence of an epoxide moiety by the resonances of H-16 (δ 5.58 d, *J* = 2.3 Hz) and H-15 (δ 3.58 d, *J* = 2.3 Hz), while the expected ring C contraction was revealed by the recorded resonances at δ 2.59 (H-14 m), 1.89 (H-11β m), and 1.69 (H-11α m). The ^13^C NMR spectrum showed signals for C-12 and C-13 at δ 180.6 and 55.1, characteristic of the spirolactone system. The signals at δ 77.7 and 57.3 revealed the presence of the epoxide moiety. According to the analogy of [α]_D_ value and NMR results, the stereochemistry of **4b** resulted in the same as that established for compounds **1b**-**3b**; consequently, the obtention of (−)-(5*R*,6*R*,7*R*,8*S*,9*S*,10*R*,13*R*,14*R*,15*S*,16*R*)-6-benzoyloxy-15,16-epoxy-5,7-dihydroxy-13-spirocassan-12,16-olide (**4b**). Complete assignment of the ^1^H and ^13^C NMR spectra is shown in Tables 1 and 2 and was supported with 2D NMR experiments (see Figures S49, S50, S52, and S53).

**Table 3:** Crystallographic Data of Compounds **2a** and **3a**

crystallographic data	**2a**	**3a**
Empirical formula	C_20_H_30_O_4_	C_20_H_28_O_4_
formula weight (g/mol)	334.44	332.42
crystal size (mm^3^)	0.37 × 0.2 × 0.09	0.15 × 0.12 × 0.02
crystal system	triclinic	orthorhombic
space group	*P*1	*P*2_1_2_1_2_1_
*a* (Å)	6.211(3)	7.303(3)
*b* (Å)	7.454(3)	11.389(5)
*c* (Å)	21.325(9)	20.777(9)
α (°)	87.839(9)	90
β (°)	85.546(9)	90
γ (°)	66.036(8)	90
*V* (Å^3^)	899.5(7)	1728.2(13)
*Z*	2	4
*D*_calcd_ (mg/mm^3^)	1.235	1.278
μ (mm^–1^)	0.08	0.09
*F*(000)	364.0	720.0
GoF^2^	1.027	1.066
temperature (K)	296(2)	295(2)
θ range [°]	2.9 to 19.1	2.6 to 18.0
reflections collected	29434	33738
independent reflections	5106	1846
*R*_int_	0.202	0.280
*R*_1_, *wR*_2_ [*I* > 2σ(*I*)[%]	0.088%, 0.221%	0.066%, 0.164%
largest diff. peak/hole [eÅ^–3^]	0.201/–0.202	0.202/–0.195

### 2.2 Single Crystal X-ray Diffraction Analysis

Evaporation of the solvent at room temperature using acetonitrile/hexanes (1:2) and hexanes-CH_2_Cl_2_ (2:3) afforded appropriate crystals for single-crystal X-ray analysis of compounds **2a** (Figure 2a), and **3a** (Figure 2b). Two molecules per unit cell were observed in **2a**, which has space group *P*1. While a *P*2_1_2_1_2_1_ space group with *Z* = 4 was observed for **3a**. Additional crystallographic data are listed in Table 3.

**Figure 2:**
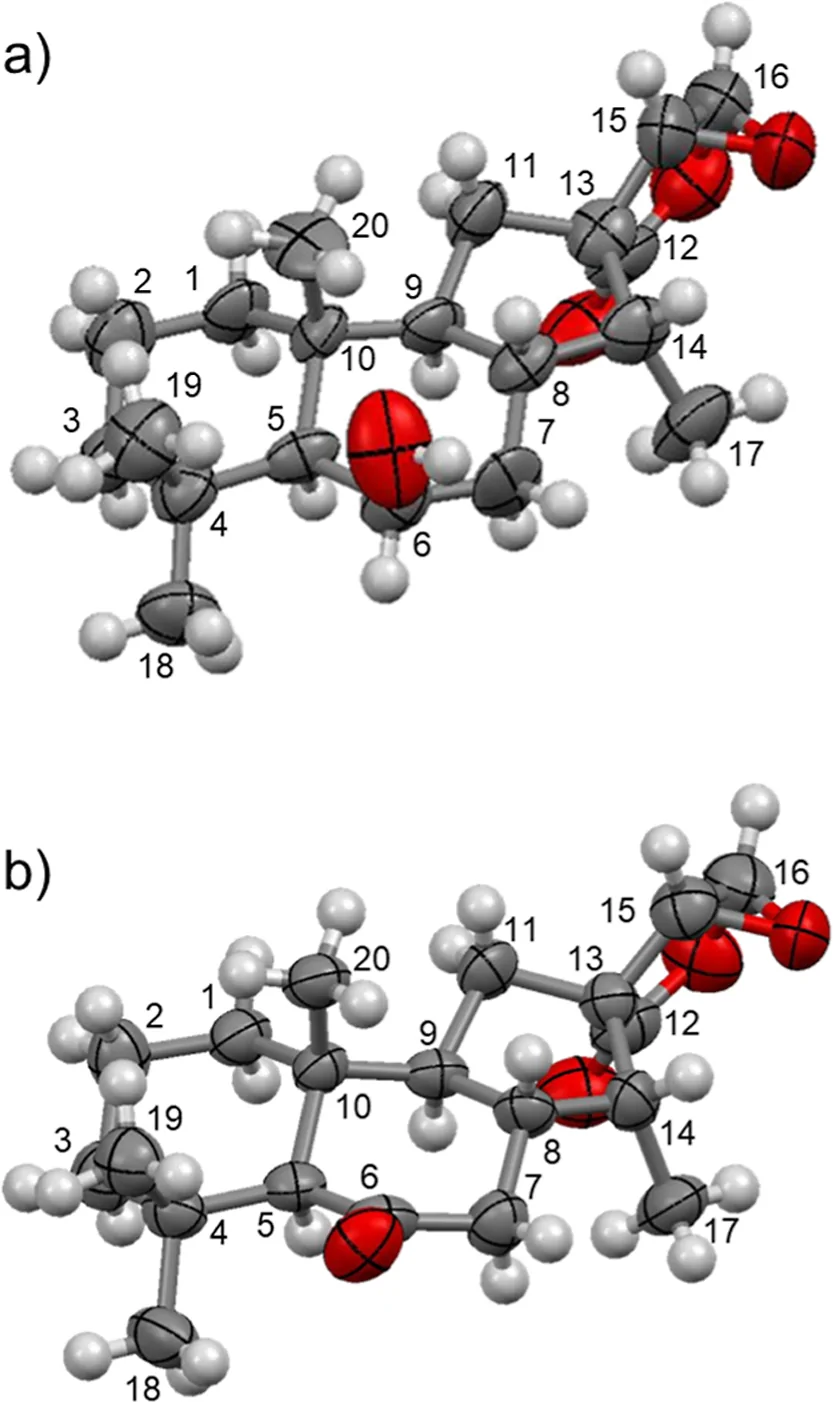
X-ray diffraction structure of (a) (+)-(5*S*,​6*R*,8*S*,​9*S*,10*R*,​13*S*,14*R*,​15*R*,16*S*)-15,16-epoxy-6-hydroxy-13-spirocassan-12,16-olide (**2a**) and (b) (+)-(5*S*,​8*S*,9*S*,​10*R*,13*S*,​14*R*,15*R*,​16*S*)-15,16-epoxy-6-oxo-13-spirocassan-12,16-olide (**3a**). Ellipsoids are drawn at 50% probability level.

As observed in both structures, the stereochemistry at C-5, C-8, C-9, C-10, and C-14 was crucial for confirming the chirality of the molecules, since these positions are part of the carbon skeleton of the starting materials and remain unchanged. It follows that three new stereogenic centers are formed after the oxidative processes (C-13, C-15, and C-16). Thus, the descriptors of the asymmetric carbons in **2a** and **3a** are 5*S*, 8*S*, 9*S*, 10*R*, and 14*R*. In consequence, the asymmetric carbons C-13, C-15, and C-16 resulted as 13*S*,15*R*,16*S*. It is worth noting that configurational restrictions in oxidized diterpenoids **2a** and **3a** are evident in these structures, which can be extended to all spirolactones from this study. Thus, valuable support from the NOESY experiments is considered in the configurational approaches of the molecules herein documented. According to these results, the absolute configurations of **2b**, **3b**, **4a**, and **4b** are confirmed.

### 2.3 Molecular Modeling

To complement the absolute configuration determination of compounds **1a–4a** and **1b–4b**, computational calculations of their specific rotations were carried out. This methodology has been very useful in supporting the absolute configuration analyses of diterpene compounds.^33^ Furthermore, the evident conformational restrictions in all epoxyspirolactones herein synthesized are convenient for this theoretical strategy due to the resultant computational economy. For this purpose, molecular models of all epoxyspirolactones (**1a–4a** and **1b–4b**) were independently constructed in the Spartan’14 program and submitted to the Monte Carlo method using the Merck Molecular Force Field (MMFF), as implemented in the software mentioned above. Conformers within the 0–5 kcal/mol gap were energy-optimized using the Density Functional Theory (DFT) at the B3LYP/6-31G** functional/basis set. Those conformers within the 0–3 kcal/mol energy window were geometry optimized using DFT at the B3LYP/DGDZVP functional/basis set. This protocol has been successfully employed in configurational studies on natural products.^34–36^ The specific rotation values of conformers in the 0–3 kcal/mol energy gap were calculated using the DFT B3LYP/ccp-VDZ or B3LYP/DGDZVP functional/basis set, which are useful for configurational analysis.^37–39^ The results are listed and compared with those experimental values in Table 4. Herein, calculations for **1a–3a** and **1b–3b** revealed a single conformer in each case, confirming higher strain in each molecule. For compounds **4a** and **4b**, four and five conformers, respectively, were obtained from the geometry optimization process. Consequently, the Boltzmann-averaged results were considered in each case (Table 4). Thermochemical results of these calculations are listed in Table S1. Comparison of the experimental and calculated [α]_D_ values supported the absolute configuration of compounds **2a–4a** and **2b–4b** and confirmed the absolute configuration of the previously described spirolactones **1a** and **1b**.^24^ As observed in Table 4, the trend of the sign for the [α]_D_ values was validated since calculations of those compounds with the 13*S*,15*R*,16*S* configuration (**1a–4a**) exhibited a dextrorotatory property. In contrast, the calculations of derivatives with the 13*R*,15*S*,16*R* configuration (**1b–4b**) remained for levorotatory data. For the above, the spiro portion, including the lactone and epoxide motifs, could directly influence the [α]_D_ value. Interestingly, the experimental [α]_D_ values from compounds **3a** and **3b** were significantly similar to those calculated with the DFT B3LYP/cc-pVDZ basis set (Δ_1_ = +6.6 and Δ_1_ = −0.3, respectively), probably associated with the higher conformational restrictions compared with the rest of the spirolactone derivatives herein obtained.

**Table 4:** Comparison of Experimental Specific Rotation Data with the DFT Calculated Values

Compd^a^	[α]_D_(expt)^b^	[α]_D_(calc)^b^ ^,^ ^c^	Δ_1_ ^d^	Δ_2_ ^e^	[α]_D_(calc)^b^ ^,^ ^f^	Δ_1_ ^d^	Δ_2_ ^e^
**1a**	+32	+19.8	+12.2	+51.8	+13.3	+18.7	+45.3
**1b**	–84	–100.4	+16.4	–184.4	–106.8	+22.8	–190.8
**2a**	+55	+45.8	+9.2	+100.8	+36.2	+18.8	+91.2
**2b**	–47	–73.9	+26.9	–120.9	–76.7	+29.7	–123.7
**3a**	+54	+47.4	+6.6	+101.4	+36.3	+17.7	+90.3
**3b**	–56	–55.8	–0.3	–111.8	–61.0	+5.0	–117.0
**4a**	+24	+37.8	–13.8	+61.8	+37.2	–13.2	+61.2
**4b**	–17	–27.5	+10.5	–44.5	–33.3	+16.3	–50.3

a The stereochemical descriptors (SD) for **1a**-**3a** are 5*S*,6*R*,8*S*,9*S*,10*R*,13*S*,14*R*,15*R*,16*S*. The SD for **1b–3b** are 5*S*,6*R*,8*S*,9*S*,10*R*,13*R*,14*R*,15*S*,16*R*. The SD for **4a** are 5*R*,6*R*,7*R*,8*S*,9*S*,10*R*,13*S*,14*R*,15*R*,16*S*. The SD for **4b** are 5*R*,6*R*,7*R*,8*S*,9*S*,10*R*,13*R*,14*R*,15*S*,16*R*.

b [α]_D_ in deg·[dm·g/cm^3^]^−1^.

c Calculated by using DFT B3LYP/cc-pVDZ.

d Δ_1_ = [α]_D_(expt) – [α]_D_(calc).

e Δ_2_ = [α]_D_(expt) – [α]_D_ (calc for the respective enantiomer).

f Using DFT B3LYP DGDZVP.

It should be noted that cassane-type spirolactones are rare compounds in nature, since few studies on their isolation have been reported.^28,30,40,41^ These compounds, named spirocaesalmins, were first described in 2001 from *Caesalpinia minax*.^30^ Currently, spirocaesalmin,^30,41,42^ spirocaesalmin B,^43^ and spirocaesalmin C^44^ are described. This nomenclature results in a practical manner to evoke this class of compounds; thereby, we suggest complementing this block of compounds as follows: spirocaesalmin D (**1a**), isospirocaesalmin D (**1b**), spirocaesalmin E (**2a**), isospirocaesalmin E (**2b**), spirocaesalmin F (**3a**), isospirocaesalmin F (**3b**), spirocaesalmin G (**4a**), and isospirocaesalmin G (**4b**). Consequently, our research offers a straightforward semisynthesis strategy for obtaining multifunctionalized spirocaesalmin compounds. Moreover, this chemical strategy could lead to the obtention of potential antiviral^30^ and cytotoxic derivatives.^22,28^

### 2.4 Cytotoxicity and Apoptosis Studies

Based on the above, the epoxylactones **1a–4a** and **1b–4b**, along with their respective precursors **1–4**, were tested to determine their cytotoxicity potential and to establish a scientific basis for considering them as prospective compounds in cancer therapy, as well as other diseases related to dysregulated apoptosis pathway, such as cardiovascular diseases, neurodegenerative disorders, or autoimmune diseases.^45^ For this purpose, in vitro assays using the human cervical cancer (HeLa cell line), human breast cancer (MDA-MB231 cell line), and normal colon CCD-18Co cell line were conducted. These cell lines, like many others, have long been used as preclinical models due to their high-throughput capability for pharmaceutical drug screening.^46,47^ Thus, assays using this valuable biological material to test synthetic and natural products and their derivatives may serve as an effective experimental approach for discovering new therapeutic agents, particularly against the most common cancer types, such as breast and cervical cancers.^5,48,49^

First, the cytotoxicity of all compounds (**1–4**, **1a–4a**, and **1b–4b**) herein obtained was determined using the MTT assay.^50^ The results revealed that compounds **4a** and **1b** provided the highest activity against the HeLa cell line, showing IC_50_ values of 9.2 (7.5–28.7) and 13.9 (13.3–20.2) μM, respectively (Table 5, Figure 3a–d). Compounds **1a**, **2**, **2a**, **3**, and **4b** exhibited IC_50_ values in the 16.7 (16.5–18.5) to 38.6 (37.1–55.5) μM range, while vouacapanes **1** and **4**, as well as the lactones **2b**, **3a**, and **3b**, showed IC_50_ values above 50 μM.^51–53^ The assays on MDA-MB-231 cell line (Table 5, Figure 3e–h) revealed that spirolactones **2a**, **3b**, and **1b** had the best activity showing IC_50_ values of 8.7 (8.4–11.8), 8.79 (8.5–11.3), and 11.3 (10.9–15.7) μM, respectively. For its part, compounds **1a**, **2b**, **3**, **4a**, and **4b** showed IC_50_ values in the 14.4 (13.9–19.4) to 49.3 (48.3–61.2) μM range (Table 5), while vouacapanes **1**, **2**, and **4**, as well as the spirolactone **3a**, were poorly active against this cancer cell line. Interestingly, the cytotoxic effects on the CCD-18Co normal cell line (Table 5, Figure 3i–l) were considered low for compounds **1b**, **2a**, **3**, **3a**, **3b**, **4**, **4a**, and **4b** as the IC_50_ values fell within the 24.6 (21.6–59.8) to 47.7 (46.4–63.4) μM range. Compounds **1**, **1a**, **2**, and **2b** yielded IC_50_ values higher than 50 μM for CCD-18Co cell line. It is worth noting that the cytotoxic activity of the active compounds showed dose-dependent behavior across all tested cell lines (Figures S56–S61). The antiproliferative positive control (methotrexate) showed IC_50_ values of 25.2 (24.3–35.8), 23.5 (22.8–35.8), and 29.6 (29.1–35.2) μM for the HeLa, MDA-MB-231, and CCD18-Co cell lines, respectively. The results suggested that the contraction of ring C and oxidation of the furan moiety increase the cytotoxic activity; moreover, the observed results on both normal and cancer cells are promising, favoring this class of rearranged compounds.^54^

**Table 5:** Cytotoxicity IC_50_ of the Vouacapane Derivatives (2–4) and Epoxyspirolactones (**1a–4a** and **1b–4b**) in Human Cell Lines^a^ ^b^

Compound	HeLa	MDA-MB-231	CCD-18Co
**2**	38.6 (37.1–55.5)	–^c^	–^c^
**3**	34.8 (33.9–45.5)	19.4 (18.7–26.9)	37.7 (36.6–48.3)
**4**	–^c^	–^c^	47.7 (46.4–63.4)
**1a**	33.9 (33.6–36.4)	49.3 (48.3–61.2)	–^c^
**1b**	13.9 (13.3–20.2)	11.3 (10.9–15.7)	–^c^
**2a**	16.7 (16.5–18.5)	8.7 (8.4–11.8)	30.7 (30.1–36.9)
**2b**	–^c^	14.4 (13.9–19.4)	–^c^
**3a**	–^c^	–^c^	24.6 (21.6–59.8)
**3b**	–^c^	8.79 (8.5–11.3)	34.3 (33.3–46.2)
**4a**	9.2 (7.5–28.7)	22.1 (19.6–52.2)	39.8 (37.3–69.9)
**4b**	–^c^	42.9 (40.7–68.6)	27.9 (26.9–39.8)

a Inhibitory concentrations IC_50_ in μM [95% confidence intervals] were determined after 24 h of treatment. Nonlinear regression (curve fit), log (inhibitor/inductor) vs response (variable slope), *R* ^2^ = 0.93–0.98 were calculated with GraphPad Prism 10.0. Methotrexate (100 μM) was used as the positive control showing for HeLa 25.2 [24.3–35.8], for MDA-MB-231 23.5 [22.8–35.8], and for CCD-18Co 29.6 [29.1–35.2].

b Confidence intervals were calculated with [AVG – 0.52 × SD–AVG + 6.29 × SD].

c Nonactive (values above 50 μM).

**Figure 3:**
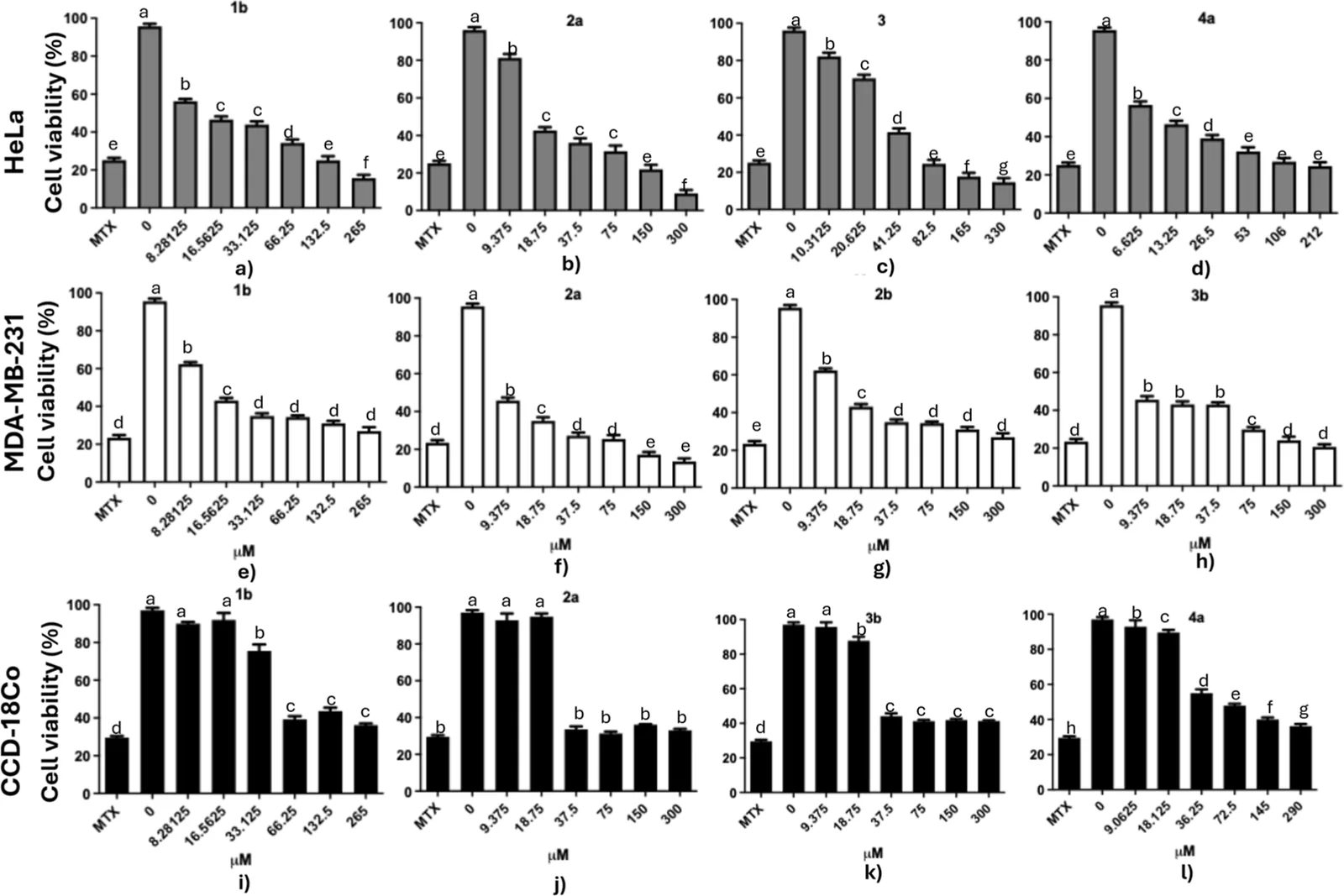
Cytotoxic effect of synthesized compounds on the viability of HeLa, MDA-MB-231, and CCD-18Co cell lines after treatment for 24 h. MTX was used as the positive reference compound at 100 μM. (a–d) The percentage of viability corresponding to HeLa treated with (a) **1b**; (b) **2a**; (c) **3**; (d) **4a**. (e–h) The percentage of viability corresponding to MDA-MB-231 treated with (e) **1b**; (f) **2a**; (g) **2b**; (h) **3b**. (i–l) The percentage of viability corresponding to CCD-18Co treated with (i) **1b**; (j) **2a**; (k) **3b**; (l) **4a**. Viability was determined using the MTT assay. Results were expressed as a percentage of viable cells. Lowercase letters denote significant differences within the treatments [one-way analysis of variance was carried out with Tukey’s post hoc test; n = 3 (P < 0.05)]. Bars correspond to the mean ± SE of three independent experiments. Abbreviations: MTX, methotrexate.

The MTT assay showed that some derivatives exhibited significant cytotoxic activity (Table 5, Figure 1). Compound **2b** showed good cytotoxicity against the MDA-MB-231 cancer cell line, whereas IC_50_ values >50 μM were observed in HeLa and CCD-18Co cell lines. By its part, the hydroxy derivative **2** exhibited moderate activity against HeLa cells but was inactive against MDA-MB-231 and CCD-18Co cell lines. On the other hand, **1a** and **1b** showed moderate to good cytotoxic activity against both cancer cell lines but were inactive against the normal cell line (IC_50_ > 50 μM). Compounds **3b** and **4b** were active against MDA-MB-231 cells, while showing moderate activity against CCD-18Co cells. Compounds **3a** and **4** exhibited selective antiproliferative activity on the CCD-18Co cell line, and compounds **2a**, **3**, and **4a** were active against the three tested cell lines. It is worth noting that only one report on the antiproliferative activity of epoxyspirolactones derived from natural vouacapanes is available for adherent human liver HePG2, human myeloid leukemia K562, human prostate cancer DU145, and HeLa cell lines, with IC_50_ = 12.1 μM for the HeLa cell line, compared with our results.^28^

To determine the apoptosis induction by compounds that exhibited the most cytotoxic activity, flow cytometry analysis was performed using HeLa and MDA-MB-231 cell lines, including compounds **1a**, **1b**, **2a**, **3**, **3b**, and **4a** (Figure 4).

**Figure 4:**
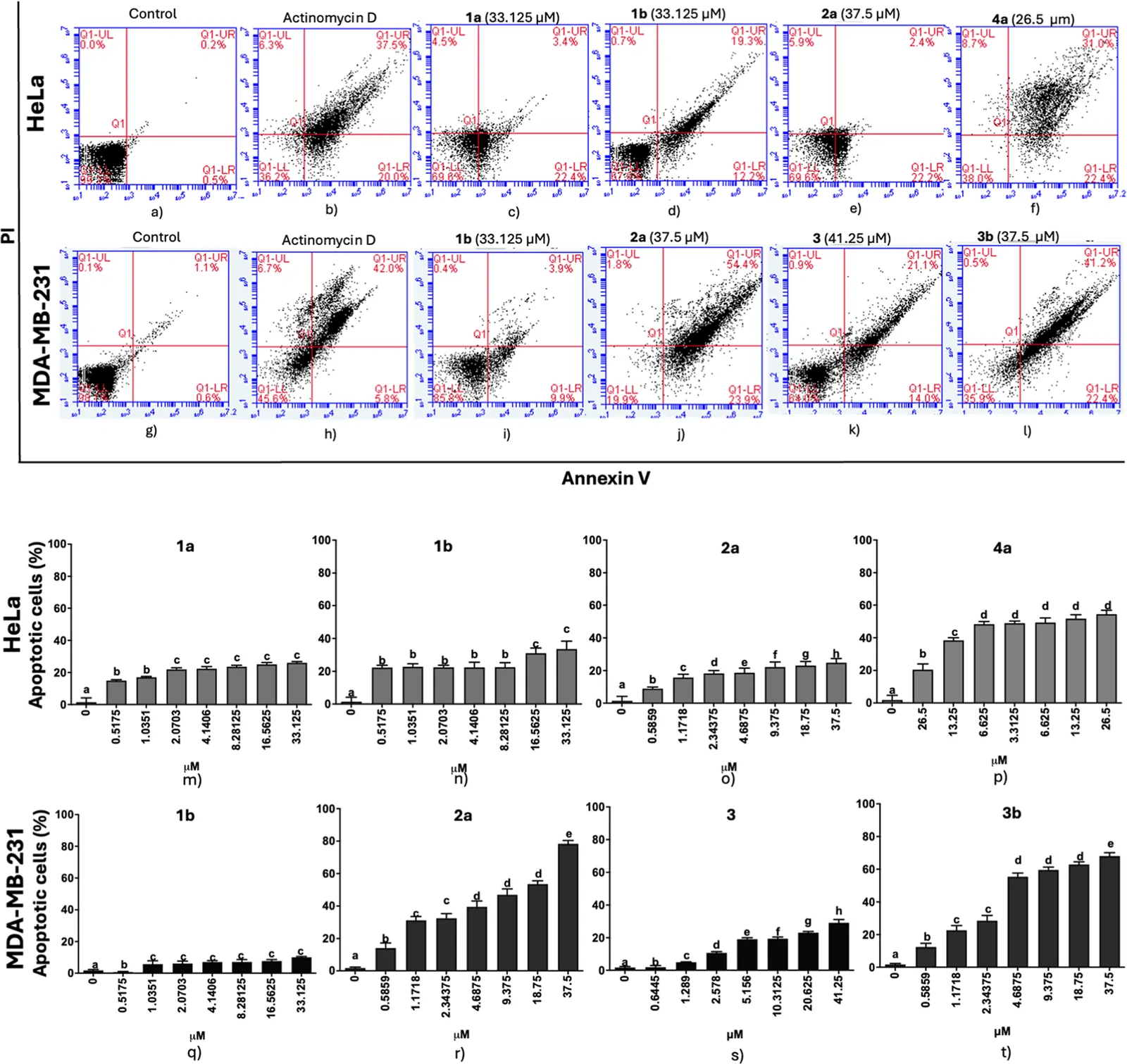
Induction of apoptosis in HeLa cells by compounds **1a**, **1b**, **2a**, **4a**, and MDA-MB-231 cells by compounds **1b**, **2a**, **3**, and **3b**. Cells were incubated with the compounds for 24 h, developed using annexin V and propidium iodide, and analyzed by flow cytometry. Percentages of fluorescent cells, determined from dot plots, were used in this analysis. (a–l) Schematic diagrams of dot plots, showing the quadrant divisions for the determination of apoptosis using the annexin V and PI probes. Cell populations for each treatment were gated in the forward scatter and side scatter analyses to eliminate dead cells and cell debris. Populations corresponding to autofluorescence or basal fluorescence are in the lower-left quadrants. Cells with increased fluorescence emission of at least one log unit are in the lower-right quadrants. The percentage of fluorescent cells is indicated in the dot plots. [(a)–(f)] HeLa cell treatment: (a) DMSO (0.05%; negative control); (b) actinomycin D (20 mg/mL; positive control); (c) 33.125 μM **1a**; (d) 33.125 μM **1b**; (e) 37.5 μM **2a**; (f) 26.5 μM **4a**. [(g)–(l)] MDA-MB-231 cell treatment: (g) DMSO (0.05%; negative control); (h) actinomycin D (20 mg/mL; positive control); (i) 33.125 μM **1b**; (j) 33.125 μM **2a**; (k) 41.25 μM **3**; (l) 37.5 μM **3b**. [(m)-(t)] Dose–response plot of apoptotic cell induction by compound treatments. Percentages of fluorescent cells, determined from dot plots, were used in this analysis. Lowercase letters denote significant differences within the treatments [one-way analysis of variance was carried out with Tukey’s post hoc test; *n* = 3 (*P* < 0.05)]. The bars correspond to the mean ± SE of three independent experiments.

The percentage of fluorescent cells (PFC), representing cells positive for the annexin V apoptosis marker, was less than 2% in the negative controls for both HeLa and MDA-MB-231 cells (Figure 4). However, when actinomycin D was used as an apoptosis inducer, the PFC increased significantly to 50% in HeLa and MDA-MB-231 cells (Figure 4b,h). Importantly, HeLa cells treated with compound **1b** exhibited a significant increase in PFC values, with an apoptosis value of 35%, of which the lower-right quadrant reached 50% at a concentration of 33.125 μM (Figure 4d,n). On the other hand, compound **4a** showed more than 50% of total apoptosis at a concentration of 26.5 μM; and compounds that exhibited a moderate activity, such as **1a** and **2a** displayed less than 25% of PFC at concentrations of 33.1 and 37.5 μM (Figure 4m,n,p) (see Figure S65). MDA-MB-231 cells were treated with compounds **1b**, **2a**, **3**, and **3b**. Compounds **2a** and **3b** exhibited higher PFC values of 78% and 64% at concentrations of 37.4 and 37.6 μM, respectively (Figure 4r,t). Cells treated with compound **3** exhibited PFC values of 35%, with the lower-right quadrant reaching 40% at a concentration of 33.125 μM. The treatment with **1b** showed the lower PFC values (<20%) (Figures 4q and S63). Regarding PI staining for necrosis detection in HeLa and MDA-MB-231 cell cultures, no PI-positive cells were observed under the same treatment conditions (Figure 4). In addition, microscopic observations confirmed these findings, as HeLa and MDA-MB-231 cells treated with the diterpenes displayed characteristic apoptotic morphology,^55^ similar to the pattern observed in methotrexate-treated cells (Figures S62 and S63). Quantitative annexin V/PI analysis further demonstrated that several spiroepoxylactone derivatives promoted both early and late apoptotic events (Table 6). In HeLa cells, compounds **1a** (33.1 μM), **2a** (37.5 μM), and **4a** (26.5 μM) induced early apoptosis levels close to or above 20%, while compound **4a** also produced elevated late apoptosis values [30.2% (29.7–35.2)]. In MDA-MB-231 cells, compounds **2a** (37.5 μM) and **3b** (37.5 μM) displayed particularly strong apoptotic responses, combining high levels of early apoptosis [22.9% (22.4–28.5) and 21.5% (21.0–27.1), respectively] with markedly increased late apoptosis populations [53.5% (53.0–58.5) and 40.8% (40.5–43.3), respectively]. These findings suggest that the analyzed compounds not only initiate apoptosis but also promote progression toward irreversible apoptotic stages. Induction of early apoptosis is considered one of the main goals of many chemotherapeutic and targeted anticancer therapies because it enables controlled elimination of tumor cells while maintaining plasma membrane integrity, thereby avoiding inflammatory damage commonly associated with necrotic cell death.^56^ Unlike necrosis, apoptotic cell death represents a cleaner and more immunologically controlled mechanism for tumor elimination. Consistent with these results, PI staining revealed minimal necrotic populations in both HeLa and MDA-MB-231 cultures under the same treatment conditions (Figure 4), indicating that loss of viability was primarily associated with apoptosis rather than nonspecific membrane disruption. Although this screening remains limited to a 24 h MTT assay, one normal cell line, and qualitative/semiquantitative annexin V/PI follow-up on selected compounds, the data indicate that oxidized vouacapanes, particularly spirolactone derivatives, showed cytotoxic effects on the HeLa and MDA-MB-231 cancer cell lines by inducing apoptosis more efficiently than the anticancer compound methotrexate. Collectively, these results suggest that vouacapane-derived spiroepoxylactones reduce the viability of HeLa and MDA-MB-231 cells predominantly through activation of apoptotic pathways.

**Table 6:** Quantitative Analysis of Apoptosis and Necrosis Induced by Spiroepoxylactones in HeLa and MDA-MB-231 Cells after Annexin V/PI Staining^a^

	HeLa				MDA-MB-231			
	**1a** (33.1 μM)	**1b** (33.1 μM)	**2a** (37.5 μM)	**4a** (26.5 μM)	**1b** (33.1 μM)	**2a** (37.5 μM)	**3** (41.3 μM)	**3b** (37.5 μM)
early apoptosis	21.8 (21.4–25.5)	11.3 (10.8–16.3)	21.3 (20.8–26.9)	22.1 (21.8–24.6)	9.2 (8.8–13.6)	22.9 (22.4–28.5)	13.5 (13.2–16.6)	21.5 (21.0–27.1)
late apoptosis	3.1 (2.9–4.3)	19.0 (18.7–21.5)	1.9 (1.6–4.4)	30.2 (29.7–35.2)	3.3 (3.0–6.4)	53.5 (53.0–58.5)	19.6 (18.7–29.6)	40.8 (40.5–43.3)
necrosis	3.9 (3.8–4.5)	0.6 (0.5–1.2)	5.3 (4.9–9.0)	8.2 (7.9–11.3)	0.5 (0.4–1.1)	1.6 (1.4–2.8)	0.8 (0.7–1.4)	0.4 (0.3–1.0)

a Data represent the mean [95% confidence intervals] of three independent experiments. Early apoptosis corresponds to annexin V+/PI– cells, late apoptosis to annexin V+/PI + cells, and necrosis to annexin V–/PI + cells. Statistical analysis was performed using one-way ANOVA followed by Tukey’s post hoc test. Confidence intervals were calculated with [AVG – 0.52 × SD–AVG + 6.29 × SD].

It has been reported that key pathways involved in cell survival, such as the phosphoinositide 3-kinase/Akt/mTOR pathway, are essential targets for developing therapeutic strategies for cancer.^57–60^ In particular, protein kinase B (Akt) plays an important role in growth and division, metabolism, and the suppression of apoptosis. The PI3K/Akt/mTOR pathway is overactivated in several types of cancer, and various efforts have been undertaken to identify new anticancer drugs that target this signaling pathway.^61,62^ Vouacapanes have been studied for their capacity to inhibit the PI3K/Akt/mTOR signaling pathway, a mechanism underlying their pro-apoptotic and immunomodulatory activities.^63–65^

### 2.5 Docking Analysis

Given the importance of these antecedents and their analogies with the current experimental results, an in silico study of the interactions of the cassane compounds **1b**, **2a**, **3**, **3b**, and **4a** with the protein kinase B (Akt) was performed. The results included binding affinity energies, intermolecular interactions, and their types (Table 7). Figure 5 presents a didactic representation of the results obtained in this study. All compounds showed interactions in the allosteric modulatory ATP-binding site,^66,67^ which is recognized to have an inhibitory effect.^68–70^ As seen in Table 7, recurrent interactions were determined in each analyzed cassane with the amino acid residues Leu181 and Phe161 (Table 7). These interactions were also observed with the native ligand. Particularly, compound **1b** showed the highest binding affinity of −9.87 kcal/mol, and the highest number of interactions, including a hydrogen bond with Ser7 residue that surely stabilizes the binding interaction. Other important intermolecular interactions of **1b** in the Akt protein are those with amino acids Thr160, Phe161, Glu191, and His194, which are in the ATP-binding cleft.^71^ Compound **2a** displayed a binding energy of −8.69 kcal/mol, whose intermolecular interactions include a hydrogen bond with Asp292 as an acceptor residue, and a dipole–dipole interaction with Thr195. Additional interactions are listed in Table 7. For its part, spirolactone **3b** exhibited a binding energy of −8.06 kcal/mol, whose stronger intermolecular interactions included dipole–dipole interactions with Asp292, Thr195, and Ser7. Induced dipole and van der Waals forces were also established as intermolecular interactions and are listed in Table 7. Lactone **4a** displayed a binding energy of −8.41 kcal/mol, where its hydrogen bond intermolecular interaction with Asp292 as an acceptor residue was highlighted. Interestingly, vouacapane **3** showed the highest similarity in interactions compared to the control, but lacked hydrogen-bond interactions (Table 7, Figure 5), which could be related to its binding energy of −8.61 kcal/mol.

**Table 7:** Binding Affinity and Molecular Interactions of Ligands **1b**, **2a**, **3**, **3b**, **4a** with the Akt Protein

Ligand	Binding Affinity^a^	amino acids of the Akt protein forming intermolecular interactions	type of Interaction
**1b**	–9.87	Ser7	hydrogen bond, acceptor residue
		Glu198, Thr195, Glu191	dipole–dipole
		Asp292, Lys179, His194, Asp274, Tyr272, Thr6, Thr197, Lys276, Asn279, Thr291, Asn199, Lys189, Cys310, Asp292, Thr160	induced dipole
		Ile186, Leu196, Leu223, Phe293, Ala9, Ala193, Leu181, Phe161, Phe225, Phe8, Leu295, Gly294	van der Waals forces
**2a**	–8.69	Asp292	hydrogen bond, acceptor residue
		Thr195	dipole–dipole
		Gly159, Asp292, Phe161	induced dipole
		Lis179, Gly162, Glu191, Leu181, Gly294	van der Waals forces
**3**	–8.61	Glu234, Leu156, Gly157, Gly159, Phe161, Gly162	dipole–dipole
		Leu156, Gly157, Gly159, Phe161, Gly162, Lys163, Leu181, Phe442, Lys158, Val164, Lys179, Leu156	van der Waals forces
**3b**	–8.06	Asp292, Thr195, Ser7	dipole–dipole
		Ile186, Thr195, Ser7	Induced dipole
		Leu181, Asp292, Phe161, Glu191	van der Waals forces
**4a**	–8.41	Asp292	hydrogen bond, acceptor residue
		Glu191, Thr195, Lys179	dipole–dipole
		Gly159, Thr6	Induced dipole
		Phe161, Gly162, Val164, Leu181, Asp292	van der Waals forces
control^b^	–9.88	Lys158, Ala230	hydrogen bond, donor residue
		Ala230, Glu234	hydrogen bond, acceptor residue
		Glu228	dipole–dipole
		Thr160, Gly159, Gly162, Phe161, Tyr229	induced dipole
		Lys163, Glu184, Lys158, Lys182, Val185, Met281, Val164, Ala177, Leu181, Thr211, Glu234, Phe442, Phe438, Met227, Leu156, Gly157, Ala230	van der Waals forces

a Binding energy (Δ*G*) is given in kcal/mol and was determined with AutoDock v. 4.2.6.

b *N*-{(2*S*)-3-[(3*S*)-8′,9′-dihydro-1*H*,3′*H*-spiro­[piperidine-3,7′-pyrano­[3,2-*e*]­indazol]-1-yl]-2-hydroxypropyl}-*N*-(2-ethoxyethyl)-2,6-dimethylbenzenesulfonamide) is the native ligand.

**Figure 5:**
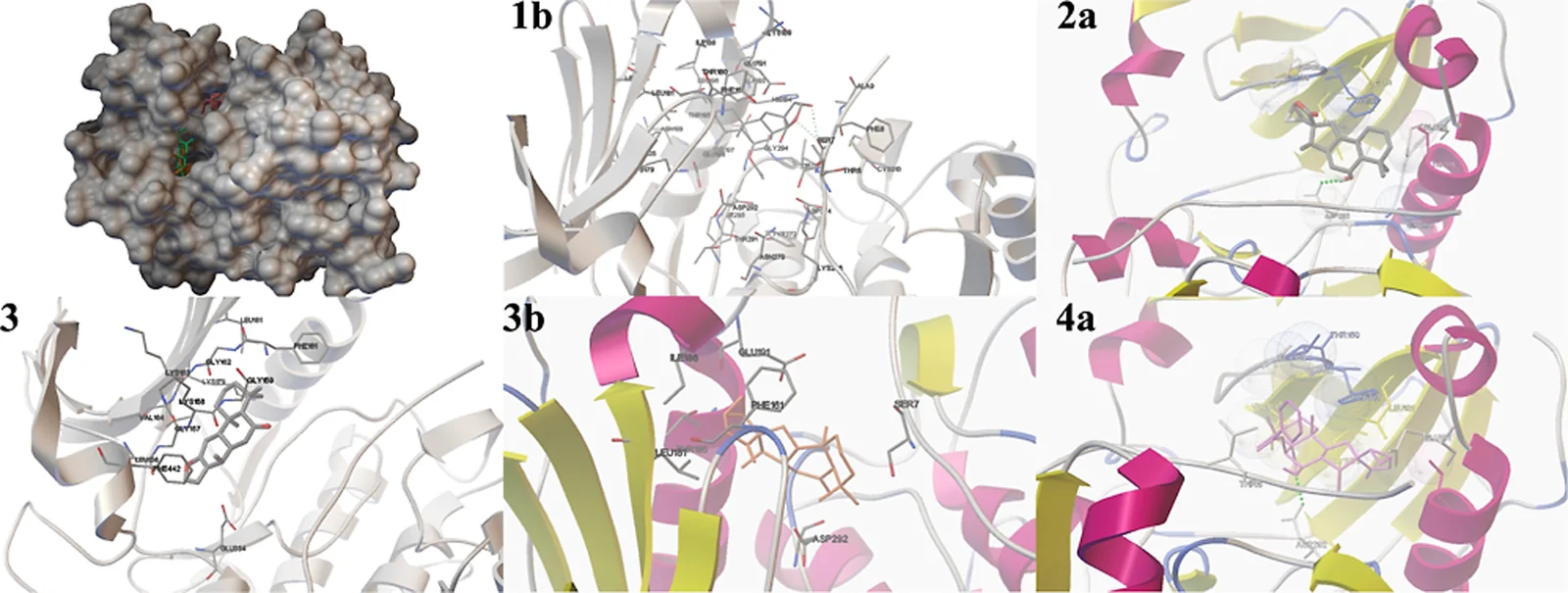
Merging of ligands at the highest affinity binding site on the Akt protein (upper and left) and docking interactions of compounds **1b** (upper and center), **2a** (up and right), **3** (lower and left), **3b** (lower and center), and **4a** (lower and right) in the allosteric modulation ATP-binding site.

## 3 Conclusion

In summary, diastereomeric epoxyspirolactones **1a–4a** and **1b–4b** were readily prepared by photooxidation of natural vouacapane derivatives, for which the absolute configurations were established by single crystal X-ray diffraction and 2D NMR spectra, and complemented with computational methods. The reaction yields of the photooxidation procedures may represent a limitation of the methodology, particularly for the obtention of **3b**; however, replicability and a simple purification methodology suggest a viable strategy for obtaining compounds of biological interest. It is evident that the apoptotic induction of vouacapane compounds was enhanced by chemical modifications to their skeletal frameworks, as shown by derivatives **1a**, **3b**, and **4a**. Thus, preparing the corresponding spirolactone derivatives is an interesting alternative to improve the biological potential of organic molecules, including natural products such as vouacapane diterpenes. If the wide variety of vouacapane derivatives from nature is considered a source for preparing novel spirolactone compounds, a mine of promising bioactive compounds could be visualized.

## 4 Experimental Section

### 4.1 General Experimental Procedures

Reagents were used as purchased from Sigma-Aldrich Co (USA), and solvents, including CHCl_3_ ≥99.8%, CH_3_OH ≥99.8%, ethyl acetate ≥99.5%, hexanes ≥99%, and acetone ≥99%, were distilled before use. Melting points (uncorrected) were measured on a Fisher-Johns apparatus (Thermo Fisher Scientific Inc., Waltham, MA, USA). IR spectra were measured on a Thermo Scientific Nicolet IS10 spectrophotometer using the ATR technique (Thermo Fisher Scientific Inc., Waltham, MA, USA), calibrated with the built-in System Performance Verification (SPV) system. Optical rotations were recorded at 20 °C in CHCl_3_ or CH_3_OH solutions on a PerkinElmer 341 polarimeter (PerkinElmer, Inc., Waltham, MA, USA) whose calibration was verified with a known sample (L-menthol, ≥99%, Sigma-Aldrich Co). The NMR spectra were acquired at 400 MHz for ^1^H and 100 MHz for ^13^C on a Varian Mercury 400 spectrometer (Varian, Inc., Palo Alto, CA, USA) from CDCl_3_ 99.8 atom % D solutions using TMS as the internal standard or residual solvent peak. High-resolution mass spectra (HRMS) were obtained with a Waters Xevo QTOF instrument (Agilent Technologies, Inc., Santa Clara, CA, USA) equipped with an electrospray ionization source (ESI+), using enkephaline leucine as an internal calibrant or by electrospray ionization (HRESIMS) on a Thermo Fisher Scientific Orbitrap Exploris 120 mass spectrometer (Thermo Fisher Scientific Inc., Waltham, MA, USA) at Laboratorios Centrales, Cinvestav, Mexico City. Column chromatography was performed using Merck silica gel 60 (230–400 mesh). The natural vouacapane derivatives **1** and **4** were isolated from *Coulteria platyloba* ^25^ and *C. pulcherrima*,^26^ respectively, following the published methodologies. Derivatives **2** and **3** were prepared according to the previously reported method.^25^ Compounds **1–4** were characterized by their physical and spectroscopic data and compared with the literature.^25,26^

### 4.2 General Procedure for the Photooxidation Reaction

A mixture of **1**, **2**, **3**, or **4** (0.3 mmol, 103, 97, 91, and 132 mg, respectively) with methylene blue BioReagent (3 mol %) was dissolved in acetonitrile 99.5% (1 M) and stirred and irradiated with white light (Sylvania Mini-Lynx T 20W) in an open vessel at room temperature for 0.5–5 h. The reactions were monitored by TLC and stopped once the starting material was consumed. Then, the solvent was evaporated, and the crude reaction product was purified by flash column chromatography using hexanes-CH_2_Cl_2_ mixtures as the mobile phase.

#### (+)-(5*S*,6*R*,8*S*,9*S*,10*R*,13*S*,14*R*,15*R*,16*S*)-6-Acetoxy-15,16-epoxy-13-spirocassan-12,16-olide (**1a**)

Prepared from **1** using the general methodology and 2 h of reaction time. Compound **1a** (17 mg, 15%) was obtained as a white solid (mp 202–203 °C) after column chromatography of the crude reaction (120 mg) using hexanes-CH_2_Cl_2_ (1:2) as the eluent, collecting fractions of 5 mL each. [α]_D_ +32 (*c* 0.94 CHCl_3_), IR, and NMR data are concordant with published data.^24^

#### (−)-(5*S*,6*R*,8*S*,9*S*,10*R*,13*R*,14*R*,15*S*,16*R*)-6-Acetoxy-15,16-epoxy-13-spirocassan-12,16-olide (**1b**)

Prepared from **1** using the general methodology and 2 h of reaction time. Compound **1b** (17 mg, 15%) was obtained as a white solid (mp 197–198 °C) after column chromatography of the crude reaction (120 mg) using hexanes-CH_2_Cl_2_ (1:2) as the eluent, collecting fractions of 5 mL each. [α]_D_ −84 (*c* 1.05 CHCl_3_), IR, and NMR data are concordant with published data.^24^

#### (+)-(5*S*,6*R*,8*S*,9*S*,10*R*,13*S*,14*R*,15*R*,16*S*)-15,16-Epoxy-6-hydroxy-13-spirocassan-12,16-olide (**2a**)

Prepared from **2** using the general methodology at 3 h of reaction time. Compound **2a** (12.5 mg, 12%) was isolated as an amorphous powder after column chromatography (110 mg) using hexanes-CH_2_Cl_2_ (1:3) as the eluent and collecting fractions of 5 mL each. Colorless crystals (via slow evaporation of solvent at room temperature using hexanes/CH_3_CN, 2:1) mp 205–207 °C. IR ν_max_ (cm^–1^) 3573, 3559, 2971, 2917, 2866, 2845, 1770, 1468, 1395, 1322, 1233, 1159, 1081, 1056, 1020, 951. HRMS TOF ES^+^ *m*/*z* 335.2225 [M + H]^+^ (calcd for C_20_H_30_O_4_ + H^+^: 335.2222). [α]_D_ +55, [α]_578_ +58, [α]_546_ +65, [α]_436_ +109, [α]_365_ +169 (*c* 0.5, CHCl_3_). ^1^H and ^13^C NMR data, see Tables 1 and 2, respectively.

#### (−)-(5*S*,6*R*,8*S*,9*S*,10*R*,13*R*,14*R*,15*S*,16*R*)-15,16-Epoxy-6-hydroxy-13-spirocassan-12,16-olide (**2b**)

Prepared from **2** using the general methodology at 3 h of reaction time. Compound **2b** (12.3 mg, 12%) was isolated as a white solid (mp 187–189 °C) after column chromatography (110 mg) using hexanes-CH_2_Cl_2_ (1:2) as the eluent, collecting fractions of 5 mL each. IR ν_max_ (cm^–1^) 3546, 3091, 2905, 2861, 2844, 1787, 1459, 1446, 1399, 1376, 1325, 1261, 1236, 1211, 1153, 1091, 1047, 961, 850. HRMS TOF ES^+^ *m*/*z* 335.2272 [M + H]^+^ (calcd for C_20_H_30_O_4_ + H^+^: 335.2222). [α]_D_ −47, [α]_578_ –55, [α]_546_ –63, [α]_436_ –106, [α]_365_ –163 (*c* 0.58, CHCl_3_). ^1^H and ^13^C NMR data, see Tables 1 and 2, respectively.

#### (+)-(5*S*,8*S*,9*S*,10*R*,13*S*,14*R*,15*R*,16*S*)-15,16-Epoxy-6-oxo-13-spirocassan-12,16-olide (**3a**)

Prepared from **3** using the general methodology at 3 h of reaction time. Compound **3a** (12 mg, 12%) was isolated as a white solid after column chromatography (110 mg) using hexanes-CH_2_Cl_2_ (2:3) as the eluent, collecting fractions of 5 mL each. Colorless crystals (via slow evaporation of solvent at room temperature using CHCl_3_-acetone 1:1) mp 224–226 °C. IR ν_max_ (cm^–1^) 3100, 2991, 2975, 2932, 2920, 2864, 1779, 1701, 1460, 1445, 1435, 1392, 1383, 1361, 1336, 1327, 1256, 1218, 1152, 1112, 1091, 1067, 1038, 1020, 982, 965, 879, 853. HRMS TOF ES^+^ *m*/*z* 333.2059 [M + H]^+^ (calcd for C_20_H_28_O_4_ + H^+^: 333.2066). [α]_D_ +54, [α]_578_ +57, [α]_546_ +63, [α]_436_ +102, [α]_365_ +134 (*c* 0.65, CHCl_3_). ^1^H and ^13^C NMR data, see Tables 1 and 2, respectively.

#### (−)-(5*S*,8*S*,9*S*,10*R*,13*R*,14*R*,15*S*,16*R*)-15,16-Epoxy-6-oxo-13-spirocassan-12,16-olide (**3b**)

A batch of **2b** (0.15 mmol, 50 mg) was dissolved in CH_2_Cl_2_ (1.5 mL), and PCC 98% (0.375 mmol, 80 mg) was added. The mixture was stirred at room temperature for 30 min. After the solvent was evaporated and the crude reaction was purified by column chromatography using a hexanes-CH_2_Cl_2_ mixture (2:3) as the eluent, collecting fractions of 5 mL each. Compound **3b** (35 mg, 70%) was isolated as a white solid (mp 223–226 °C). IR ν_max_ (cm^–1^) 3067, 2937, 2919, 2849, 1784, 1702, 1468, 1440, 1403, 1385, 1357, 1320, 1293, 1268, 1217, 1149, 1111, 1092, 1077, 1054, 1040, 1015, 995, 961, 855. HRESIMS *m*/*z* 333.2058 [M + H]^+^ (calcd for C_20_H_28_O_4_ + H^+^: 333.2060). [α]_D_ −56, [α]_578_ –59, [α]_546_ –67, [α]_436_ –116, [α]_365_ –191 (*c* 1.04, CHCl_3_). ^1^H and ^13^C NMR data, see Tables 1 and 2, respectively.

#### (+)-(5*R*,6*R*,7*R*,8*S*,9*S*,10*R*,13*S*,14*R*,15*R*,16*S*)-6-Benzoyloxy-15,16-epoxy-5,7-dihydroxy-13-spirocassan-12,16-olide (**4a**)

Prepared from vouacapane **4** using the general methodology at 5 h of reaction time. Compound **4a** (16 mg, 11%) was isolated as a colorless oil after column chromatography using a hexanes-EtOAc (7:3) mixture as the eluent, collecting fractions of 5 mL each. IR ν_max_ (cm^–1^) 3538, 2942, 2874, 2860, 1786, 1709, 1602, 1584, 1466, 1450, 1396, 1314, 1275, 1175, 1111, 1065, 987, 971, 853. HRMS TOF ES^+^ *m*/*z* 493.2206 [M + Na]^+^ (calcd for C_27_H_34_O_7_ + Na: 493.2202). [α]_D_ +24, [α]_578_ +31, [α]_546_ +36, [α]_436_ +59 (*c* 0.69, MeOH). ^1^H and ^13^C NMR data, see Tables 1 and 2, respectively.

#### (−)-(5*R*,6*R*,7*R*,8*S*,9*S*,10*R*,13*R*,14*R*,15*S*,16*R*)-6-Benzoyloxy-15,16-epoxy-5,7-dihydroxy-13-spirocassan-12,16-olide (**4b**)

Prepared from vouacapane **4** using the general methodology at 5 h of reaction time. Compound **4b** (14 mg, 10%) was isolated as a colorless oil after column chromatography using a hexanes-EtOAc (7:3) mixture as the eluent and collecting fractions of 5 mL each. IR ν_max_ (cm^–1^) 3532, 2935, 2890, 1778, 1709, 1473, 1450, 1397, 1274, 1177, 1149, 1119, 1070, 1027, 857. HRMS TOF ES^+^ *m*/*z* 471.2365 [M + H]^+^ (calcd for C_27_H_34_O_7_ + H^+^: 471.2383. [α]_D_ −17, [α]_578_ –18, [α]_546_ –20, [α]_436_ –34 (c 0.83, MeOH). ^1^H and ^13^C NMR: see Tables 1 and 2, respectively.

### 4.3 Cell Cultures

The human cancer cell lines HeLa, MDA-MB-231, and CCD-18Co were acquired from the American Type Culture Collection (ATCC, Manassas, VA, USA). Cells were grown at 37 °C in an atmosphere of 5% CO_2_ and 80% humidity in Dulbecco’s modified Eagle’s medium supplemented with 10% (v/v) fetal bovine serum and 1% antibiotic (10,000 units of penicillin, 10 mg streptomycin, and 25 μg of amphotericin B per mL).

### 4.4 Antiproliferative Assays

Dulbecco’s modified Eagle’s medium (DMEM), fetal bovine serum (FBS), antibiotic antimycotic solution (100X) penicillin, streptomycin, amphotericin B, 3-(4,5-dimethylthiazol-2-yl)-2,5-diphenyltetrazolium bromide (MTT) were purchased from Sigma-Aldrich Co (BioReagent grade). Alexa Fluor 488 annexin V and the PI/dead cell apoptosis kit were obtained from Invitrogen Life Technologies (Carlsbad, CA, USA). Tissue culture plasticware was acquired from Corning (Tewksbury, MA, USA).

The in vitro antiproliferative activity of compounds **1–4**, **1a–4a**, and **1b–4b** was determined using 3-(4,5-dimethylthiazol-2-yl)-2,5-diphenyltetrazolium bromide (MTT, Sigma-Aldrich Co) dye uptake assay.^39^ HeLa, MDA-MB-231, and CCD-18CO cells were individually seeded at a density of 3 × 10^4^ cells per well into 96-well flat-bottomed plates (Costar) in 200 μL of serum-enriched medium and incubated for 24 h. Compounds **1–4**, **1a–4a**, and **1b–4b** were individually dissolved with DMSO ≥99.9% to prepare a 10 mg/mL concentration as stock solution; therefore, the concentration in mM is 29.05 for **1**, 26.58 for **1a** and **1b**, 33.08 for **2**, 29.92 for **2a** and **2b**, 33.31 for **3**, 30.10 for **3a** and **3b**, 22.8 for **4**, 21.26 for **4a** and **4b**. Afterward, each compound was individually added in a geometric progression (*r* = 2) of concentrations from 100 to 3.12 μg/mL. It means 290.5–9.06 μM for **1**, 265.8–8.29 μM for **1a** and **1b**, 330.88–10.32 μM for **2**, 299.21–9.33 μM for **2a** and **2b**, 333.11–10.39 μM for **3**, 301.03–9.39 μM for **3a** and **3b**, 228.18–7.11 μM for **4**, and 212.66–6.63 μM for **4a** and **4b**. DMSO (≤0.1% in culture medium) was used as the vehicle control, and methotrexate BioReagent was employed as the positive control. After the treatment period (24 h), 10 μL MTT (5 mg/mL in PBS) was added to each well and incubated for 4 h at 37 °C. Then, 100 μL of 2-propanol BioReagent/1 M HCl (19:1 v/v) was added to dissolve the formazan crystals. Absorbance measurements were done in a microplate spectrophotometer (iMarK Microplate Reader, BIO-RAD, Hercules, CA, USA) at 595 nm. All assays were performed in triplicate (*n* = 3). Averaged IC_50_ values are reported in μM (μg/mL), including the corresponding standard error. Cultured cells were photographed before the MTT addition using an inverted phase-contrast microscope (Carl-Zeiss HB0–50, San Diego, CA, USA) equipped with an AxioCam/Cc1 digital camera.

### 4.5 Necrosis and Apoptosis Assays

HeLa and MDA-MB-231 cells were individually seeded in 96-well flat-bottomed plates at a density of 3 × 10^4^ cells per well in 200 μL of serum-enriched medium and incubated for 24 h. Cell culture was synchronized with serum-free medium for 12 h at 37 °C. Compounds **1a**, **1b**, **2a**, and **4a**, for HeLa cells, and **1b**, **2a**, **2b**, **3**, **3b**, **4a** for MDA-MB-231 cells, were individually added in a geometric progression (r = 2) of concentrations from 12.5 to 0.195 μg/mL. It means 33.22–0.51 μM for **1a** and **1b**, 37.40–0.58 μM for **2a** and **2b**, 41.6–0.64 μM for **3**, 37.62–0.58 μM for **3b**, 26.58–0.41 μM for **4a**. DMSO was used as the vehicle control. To determine the apoptotic effect, cells were collected by centrifugation at 2000*g* for 10 min and carefully washed with cold phosphate-buffered saline. The pellet was suspended in 20 μL of annexin buffer and treated with annexin V and propidium iodide (PI) (Dead Cell Apoptosis Kit; Molecular Probes, Invitrogen Life Technologies, Carlsbad, CA, USA) and incubated for 15 min in darkness. Fluorescence was quantified by flow cytometry (FC) using a BD Accuri C6 Flow Cytometer (BD Biosciences, San Jose, CA, USA) within 1 h to ensure optimal detections. Cell populations for each treatment were gated in forward scatter and side scatter dot plots to eliminate cell debris. Populations corresponding to auto- or basal-fluorescence were located in the left quadrant, and cells with emission of fluorescence increasing at least one log unit value were located in the right quadrant of the dot plots. In addition, the percentage of fluorescent cells (PFC) and median fluorescence intensity (FI) were determined in monoparametric histograms of fluorescence emission obtained from the dot plots, labeled as PFC and as relative units of fluorescence. The equipment was calibrated using Spherotech 8-peak (FL1–FL3) and 6-peak (FL-4) validation beads (BD Accuri, San Jose, CA, USA). For apoptosis and necrosis assays, fluorescence for annexin V was monitored in the emission fluorescence channel FL1 at 495/519 nm, and for propidium iodide in the FL2 channel at 535/617 nm. A minimum of 10,000 cellular events were analyzed.

### 4.6 Single-Crystal X-ray Diffraction

The single crystal X-ray data were collected on a Bruker APEX-II diffractometer (Bruker, Corp., Billerica, MA, USA) using Mo Kα monochromated radiation (λ = 0.71073 A) at 295 (2) K in the ω/2θ scan mode. Crystal data of compounds **2a** and **4a** are listed in Table 1. Additional data are listed in Tables S3 and S4. The structure was solved by direct methods using the SHELXS-97 program included in the WinGX v1.70.01 crystallographic software package. The non-hydrogen atoms were treated anisotropically for structural refinement, and the hydrogen atoms, included in the structure factor calculation, were refined isotropically. Crystallographic data (excluding structure factors) have been deposited at the Cambridge Crystallographic Data Center under deposition numbers 2556254 for **2a** and 2556255 for **3a**. Copies of the data can be obtained free of charge on application to the CCDC, 12 Union Road, Cambridge CB2 IEZ, United Kingdom (Fax: +44-(0)­1223-336033 or e-mail: deposit@ccdc.cam.ac.uk).

### 4.7 Specific Rotation Calculations

The in silico constructed molecular models of **1a**-**4a** and **1b**-**4b** were independently subjected to a Monte Carlo search protocol in a 10 kcal/mol energy gap using the Merck Molecular Force Field (MMFF94) as implemented in the Spartan’14 program (Wave function, Inc. & Q-Chem, Irvine, CA, USA). The resulting conformers in the 0–5 kcal/mol energy gap were submitted to single-point energy calculations using the DFT B3LYP/6-31G­(d) level of theory in the Spartan’14 program. Those conformers in the 0–3 kcal/mol range were geometry-optimized by DFT calculations using the B3LYP functional and the DGDZVP basis set employing the Gaussian 09 program (Wallingford, CT, USA). Then, the specific rotation was calculated at 298 K and 1 atm for those conformers in the 0–3 kcal/mol range using the B3LYP/DGDZVP as well as the B3LYP/ccp-VDZ levels of theory (see Supplementary Data). Thermochemical parameters were also determined. All minimum energy structures were verified for the absence of imaginary frequencies, and their relative free energies were employed to calculate their Boltzmann population. The Boltzmann-weighted [α]_D_ values were determined and considered as the [α]_D_ computed result. Molecular visualization was carried out using GaussView 6.0. Conformers in the 0–3 kcal/mol and thermochemical analysis for compounds **1a**-**4a** and **1b**-**4b** are presented in the Supporting Information (Figure S65 and Table S1). Geometry optimization required approximately 2 h of CPU time per conformer when using a CPU E5–2680 v3 with 96 GB RAM operating at 2.5 GHz × 48 core processors.

### 4.8 Molecular Docking

AKT protein (PDB ID: 3QKL) was used for docking analysis. Protein and active site coordinates were directly extracted from PDB ID: 3QKL from the Protein Data Bank Web site https://www.rcsb.org/ (accessed on 28 June 2023). The UCSF Quimera 1.15 Software (RBVI, San Francisco, CA, USA) was employed to delete unwanted residues from the protein. The AutoDock Vina program 4.2.6 (Scripps Research Institute, La Jolla, CA, USA) was used for molecular docking. The visualizations were run using AutoDock Tools 4.2 and Pymol 1.3 for Windows. Computational simulations were performed on an Intel Core i7-2670QM CPU at 2.20 GHz, 8 GB of RAM, and a NVIDIA GeForce GT video card with 550 MB of memory.

A grid box size of 50 × 50 × 62 Å in *x*, *y*, and *z* dimensions was primarily used, centered at *x* = −1.8 Å, *y* = 0.9 Å, and *z* = 24.5 Å. The Lamarckian Genetic Algorithm (GA-LS) was used to search for the best conformations, with 100 runs performed for each ligand. The population size was 150, the maximum number of evaluation energy was 2.5 × 10^6^, and the maximum number of generations was 2.7 × 10^4^. The refinement step was done using a smaller box size (40 × 40 × 40 Å) in *x*, *y*, and *z*, centered at coordinates *x* = −10.145, *y* = 5.727, and *z* = 18.185 Å to cover the best binding site with the lowest energy (Edock) and the highest number of interactions between the ligand and the protein.

Ligands **1b** and **3** were constructed and minimized using MMFF as the calculation force in the Spartan’04 program (Wave function, Inc.), followed by exploration of conformers using the Monte Carlo method with an energy of HF minimization 3-21G. The global minimum was optimized using DFT at the B3LYP/DGDZVP level of theory using the Gaussian 03W program revision C.02 (Gaussian, Inc.).

## Supplementary Material











## Notes

CIF file for compound 2a.cif (CIF)
CIF file for compound 3a.cif (CIF)
1D and 2D NMR spectra and HRMS of **1a–4a** and **1b–4b**. Thermochemical analysis for **1a–4a** and **1b–4b**. The most populated conformers of **1a–4a** and **1b–4b**. Effects of compounds **1–4**, **1a–4a**, and **1b–4b** on three cell lines (PDF)
X-ray tables for 2a.pdf (PDF)
X-ray tables for 3a.pdf (PDF)
