# Supplementary material for: Photooxidative Molecular Rearrangement of Vouacapane Diterpenes Generates Proapoptotic Spirocaesalmins
Source: ACS Omega. 2026 Aug 13;11(33):49592–606. doi: 10.1021/acsomega.6c03239 (PMC13520548; doi:10.1021/acsomega.6c03239)
Supplement: Supplementary file 1 [file ao6c03239_si_003.pdf]

Supporting information

Photooxidative molecular rearrangement of vouacapane diterpenes generates proapoptotic spirocaesalmins

Laura Hernández-Padilla[1], Leonardo R. Álvarez-García[1], Eva E. Soto-Guzmán[1], Gabriela Rodríguez-García[1], Edna M. Silva-García[2], Lorena Martínez-Alcántar[1], Jesús Campos-García[1], Christine Thomassigny[3], Rosa E. del Río[1], Armando Talavera-Alemán[1,*], Mario A. Gómez-Hurtado[1,*], Carlos M. Cerda-García-Rojas[4,*]

[1] Instituto de Investigaciones Químico Biológicas, Universidad Michoacana de San Nicolás de Hidalgo, Ciudad Universitaria, Morelia, Michoacán 58030, Mexico;

[2] Universidad La Salle, Av. Universidad 500, Cto. Erandeni I, Tarímbaro, Michoacán 58880, México;

[3] ILV-UMR CNRS 8180, Université de Versailles St-Quentin-en-Yvelines, 45 Avenue des États-Unis, 78035 Versailles, France;

[4] Departamento de Química, Centro de Investigación y de Estudios Avanzados del Instituto Politécnico Nacional, Apartado 14-740, Mexico City 07000, Mexico.

*Corresponding authors. E-mails: armando.talavera@umich.mx (A. T-A.); mario.gomez@umich.mx (M. A. G-H.); ccerda@cinvestav.mx (C. M. C-G-R.)

Table of contents

Figure S1. ^{1}H NMR spectrum of 6*β*-acetoxyvouacapane (**1**), (400 MHz, $CDCl_3$). 1

Figure S2. ^{1}H NMR spectrum of 6*β*-hydroxyvouacapane (**2**), (400 MHz, $CDCl_3$). 2

Figure S3. ^{1}H NMR spectrum of 6-oxovouacapane (**3**), (400 MHz, $CDCl_3$). 3

Figure S4. ^{1}H NMR spectrum of isovouacapenol C (**4**), (400 MHz, $CDCl_3$). 4

Figure S5. ^{1}H NMR spectrum of (+)-(5*S*,6*R*,8*S*,9*S*,10*R*,13*S*,14*R*,15*R*,16*S*)-6-acetoxy-15,16-epoxy-13-spirocassan-12,16-olide (spirocaesalmin D) (**1a**), (300 MHz, $CDCl_3$). 5

Figure S6. NOESY spectrum of (+)-(5*S*,6*R*,8*S*,9*S*,10*R*,13*S*,14*R*,15*R*,16*S*)-6-acetoxy-15,16-epoxy-13-spirocassan-12,16-olide (spirocaesalmin D) (**1a**), (300 MHz, $CDCl_3$). 6

Figure S7. ^{13}C NMR spectrum of (+)-(5*S*,6*R*,8*S*,9*S*,10*R*,13*S*,14*R*,15*R*,16*S*)-6-acetoxy-15,16-epoxy-13-spirocassan-12,16-olide (spirocaesalmin D) (**1a**), (75 MHz, $CDCl_3$). 7

Figure S8. ^{1}H NMR spectrum of (−)-(5*S*,6*R*,8*S*,9*S*,10*R*,13*R*,14*R*,15*S*,16*R*)-6-acetoxy-15,16-epoxy-13-spirocassan-12,16-olide (isospirocaesalmin D) (**1b**), (300 MHz, $CDCl_3$). 8

Figure S9. NOESY spectrum of (−)-(5*S*,6*R*,8*S*,9*S*,10*R*,13*R*,14*R*,15*S*,16*R*)-6-acetoxy-15,16-epoxy-13-spirocassan-12,16-olide (isospirocaesalmin D) (**1b**), (300 MHz, $CDCl_3$). 9

Figure S10. ^{13}C NMR spectrum of (−)-(5*S*,6*R*,8*S*,9*S*,10*R*,13*R*,14*R*,15*S*,16*R*)-6-acetoxy-15,16-epoxy-13-spirocassan-12,16-olide (isospirocaesalmin D) (**1b**), (75 MHz, $CDCl_3$). 10

Figure S11. ^{1}H NMR spectrum of (+)-(5*S*,6*R*,8*S*,9*S*,10*R*,13*S*,14*R*,15*R*,16*S*)-15,16-epoxy-6-hydroxy-13-spirocassan-12,16-olide (spirocaesalmin E) (**2a**), (300 MHz, $CDCl_3$). 11

Figure S12. COSY spectrum of (+)-(5*S*,6*R*,8*S*,9*S*,10*R*,13*S*,14*R*,15*R*,16*S*)-15,16-epoxy-6-hydroxy-13-spirocassan-12,16-olide (spirocaesalmin E) (**2a**), (300 MHz, $CDCl_3$). 12

Figure S13. NOESY spectrum of (+)-(5*S*,6*R*,8*S*,9*S*,10*R*,13*S*,14*R*,15*R*,16*S*)-15,16-epoxy-6-hydroxy-13-spirocassan-12,16-olide (spirocaesalmin E) (**2a**), (300 MHz, $CDCl_3$). 13

Figure S14. ^{13}C NMR spectrum of (+)-(5*S*,6*R*,8*S*,9*S*,10*R*,13*S*,14*R*,15*R*,16*S*)-15,16-epoxy-6-hydroxy-13-spirocassan-12,16-olide (spirocaesalmin E) (**2a**), (75 MHz, $CDCl_3$). 14

Figure S15. HSQC spectrum of (+)-(5*S*,6*R*,8*S*,9*S*,10*R*,13*S*,14*R*,15*R*,16*S*)-15,16-epoxy-6-hydroxy-13-spirocassan-12,16-olide (spirocaesalmin E) (**2a**), (300/75 MHz, $CDCl_3$). 15

Figure S16. HMBC spectrum of (+)-(5*S*,6*R*,8*S*,9*S*,10*R*,13*S*,14*R*,15*R*,16*S*)-15,16-epoxy-6-hydroxy-13-spirocassan-12,16-olide (spirocaesalmin E) (**2a**), (300/75 MHz, $CDCl_3$). 16

Figure S17. IR spectrum of (+)-(5*S*,6*R*,8*S*,9*S*,10*R*,13*S*,14*R*,15*R*,16*S*)-15,16-epoxy-6-hydroxy-13-spirocassan-12,16-olide (spirocaesalmin E) (**2a**). 17

Figure S18. HRMS spectrum of (+)-(5*S*,6*R*,8*S*,9*S*,10*R*,13*S*,14*R*,15*R*,16*S*)-15,16-epoxy-6-hydroxy-13-spirocassan-12,16-olide (spirocaesalmin E) (**2a**). 18

Figure S19. ^{1}H NMR spectrum of (−)-(5*S*,6*R*,8*S*,9*S*,10*R*,13*R*,14*R*,15*S*,16*R*)-15,16-epoxy-6-hydroxy-13-spirocassan-12,16-olide (isospirocaesalmin E) (**2b**), (300 MHz, $CDCl_3$). 19

Figure S20. ^{13}C NMR spectrum of (−)-(5*S*,6*R*,8*S*,9*S*,10*R*,13*R*,14*R*,15*S*,16*R*)-15,16-epoxy-6-hydroxy-13-spirocassan-12,16-olide (isospirocaesalmin E) (**2b**), (75 MHz, $CDCl_3$). 20

Figure S21. HSQC spectrum of (−)-(5*S*,6*R*,8*S*,9*S*,10*R*,13*R*,14*R*,15*S*,16*R*)-15,16-epoxy-6-hydroxy-13-spirocassan-12,16-olide (isospirocaesalmin E) (**2b**), (300/75 MHz, $CDCl_3$). 21

Figure S22. HMBC spectrum of (−)-(5*S*,6*R*,8*S*,9*S*,10*R*,13*R*,14*R*,15*S*,16*R*)-15,16-epoxy-6-hydroxy-13-spirocassan-12,16-olide (isospirocaesalmin E) (**2b**), (300/75 MHz, $CDCl_3$). 22

Figure S23. IR spectrum of (−)-(5*S*,6*R*,8*S*,9*S*,10*R*,13*R*,14*R*,15*S*,16*R*)-15,16-epoxy-6-hydroxy-13-spirocassan-12,16-olide (isospirocaesalmin E) (**2b**). 23

Figure S24. HRMS spectrum of (−)-(5*S*,6*R*,8*S*,9*S*,10*R*,13*R*,14*R*,15*S*,16*R*)-15,16-epoxy-6-hydroxy-13-spirocassan-12,16-olide (isospirocaesalmin E) (**2b**). 24

Figure S25. ^{1}H NMR spectrum of (+)-(5*S*,8*S*,9*S*,10*R*,13*S*,14*R*,15*R*,16*S*)-15,16-epoxy-6-oxo-13-spirocassan-12,16-olide (spirocaesalmin F) (**3a**), (300 MHz, $CDCl_3$). 25

Figure S26. COSY spectrum of (+)-(5*S*,8*S*,9*S*,10*R*,13*S*,14*R*,15*R*,16*S*)-15,16-epoxy-6-oxo-13-spirocassan-12,16-olide (spirocaesalmin F) (**3a**), (300 MHz, $CDCl_3$). 26

Figure S27. NOESY spectrum of (+)-(5*S*,8*S*,9*S*,10*R*,13*S*,14*R*,15*R*,16*S*)-15,16-epoxy-6-oxo-13-spirocassan-12,16-olide (spirocaesalmin F) (**3a**), (300 MHz, $CDCl_3$). 27

Figure S28. ^{13}C NMR spectrum of (+)-(5*S*,8*S*,9*S*,10*R*,13*S*,14*R*,15*R*,16*S*)-15,16-epoxy-6-oxo-13-spirocassan-12,16-olide (spirocaesalmin F) (**3a**), (75 MHz, $CDCl_3$). 28

Figure S29. HSQC spectrum of (+)-(5*S*,8*S*,9*S*,10*R*,13*S*,14*R*,15*R*,16*S*)-15,16-epoxy-6-oxo-13-spirocassan-12,16-olide (spirocaesalmin F) (**3a**), (300/75 MHz, $CDCl_3$). 29

Figure S30. IR spectrum of (+)-(5*S*,8*S*,9*S*,10*R*,13*S*,14*R*,15*R*,16*S*)-15,16-epoxy-6-oxo-13-spirocassan-12,16-olide (spirocaesalmin F) (**3a**). 30

Figure S31. HRMS spectrum of (+)-(5*S*,8*S*,9*S*,10*R*,13*S*,14*R*,15*R*,16*S*)-15,16-epoxy-6-oxo-13-spirocassan-12,16-olide (spirocaesalmin F) (**3a**). 31

Figure S32. ^{1}H NMR spectrum of (−)-(5*S*,8*S*,9*S*,10*R*,13*R*,14*R*,15*S*,16*R*)-15,16-epoxy-6-oxo-13-spirocassan-12,16-olide (isospirocaesalmin F) (**3b**), (300 MHz, $CDCl_3$). 32

Figure S33. COSY spectrum of (−)-(5*S*,8*S*,9*S*,10*R*,13*R*,14*R*,15*S*,16*R*)-15,16-epoxy-6-oxo-13-spirocassan-12,16-olide (isospirocaesalmin F) (**3b**), (300 MHz, $CDCl_3$). 33

Figure S34. NOESY spectrum of (−)-(5*S*,8*S*,9*S*,10*R*,13*R*,14*R*,15*S*,16*R*)-15,16-epoxy-6-oxo-13-spirocassan-12,16-olide (isospirocaesalmin F) (**3b**), (300 MHz, $CDCl_3$). 34

Figure S35. ^{13}C NMR spectrum of (−)-(5*S*,8*S*,9*S*,10*R*,13*R*,14*R*,15*S*,16*R*)-15,16-epoxy-6-oxo-13-spirocassan-12,16-olide (isospirocaesalmin F) (**3b**), (75 MHz, $CDCl_3$). 35

Figure S36. HSQC spectrum of (−)-(5*S*,8*S*,9*S*,10*R*,13*R*,14*R*,15*S*,16*R*)-15,16-epoxy-6-oxo-13-spirocassan-12,16-olide (isospirocaesalmin F) (**3b**), (300/75 MHz, $CDCl_3$). 36

Figure S37. HMBC spectrum of (−)-(5*S*,8*S*,9*S*,10*R*,13*R*,14*R*,15*S*,16*R*)-15,16-epoxy-6-oxo-13-spirocassan-12,16-olide (isospirocaesalmin F) (**3b**), (300/75 MHz, $CDCl_3$). 37

Figure S38. IR spectrum of (−)-(5*S*,8*S*,9*S*,10*R*,13*R*,14*R*,15*S*,16*R*)-15,16-epoxy-6-oxo-13-spirocassan-12,16-olide (isospirocaesalmin F) (**3b**). 38

Figure S39. HRMS spectrum of (−)-(5*S*,8*S*,9*S*,10*R*,13*R*,14*R*,15*S*,16*R*)-15,16-epoxy-6-oxo-13-spirocassan-12,16-olide (isospirocaesalmin F) (**3b**). 39

Figure S40. ^{1}H NMR spectrum of (+)-(5*R*,6*R*,7*R*,8*S*,9*S*,10*R*,13*S*,14*R*,15*R*,16*S*)-6-benzoyloxy-15,16-epoxy-5,7-dihydroxy-13-spirocassan-12,16-olide (spirocaesalmin G) (**4a**), (400 MHz, $CDCl_3$). 40

Figure S41. COSY spectrum of (+)-(5*R*,6*R*,7*R*,8*S*,9*S*,10*R*,13*S*,14*R*,15*R*,16*S*)-6-benzoyloxy-15,16-epoxy-5,7-dihydroxy-13-spirocassan-12,16-olide (spirocaesalmin G) (**4a**), (400 MHz, $CDCl_3$). 41

Figure S42. NOESY spectrum of (+)-(5*R*,6*R*,7*R*,8*S*,9*S*,10*R*,13*S*,14*R*,15*R*,16*S*)-6-benzoyloxy-15,16-epoxy-5,7-dihydroxy-13-spirocassan-12,16-olide (spirocaesalmin G) (**4a**), (400 MHz, $CDCl_3$). 42

Figure S43. ^{13}C NMR spectrum of (+)-(5*R*,6*R*,7*R*,8*S*,9*S*,10*R*,13*S*,14*R*,15*R*,16*S*)-6-benzoyloxy-15,16-epoxy-5,7-dihydroxy-13-spirocassan-12,16-olide (spirocaesalmin G) (**4a**), (100 MHz, $CDCl_3$). 43

Figure S44. HSQC spectrum of (+)-(5*R*,6*R*,7*R*,8*S*,9*S*,10*R*,13*S*,14*R*,15*R*,16*S*)-6-benzoyloxy-15,16-epoxy-5,7-dihydroxy-13-spirocassan-12,16-olide (spirocaesalmin G) (**4a**), (400/100 MHz, $CDCl_3$). 44

Figure S45. HMBC spectrum of (+)-(5*R*,6*R*,7*R*,8*S*,9*S*,10*R*,13*S*,14*R*,15*R*,16*S*)-6-benzoyloxy-15,16-epoxy-5,7-dihydroxy-13-spirocassan-12,16-olide (spirocaesalmin G) (**4a**), (400/100 MHz, $CDCl_3$). 45

Figure S46. IR spectrum of (+)-(5*R*,6*R*,7*R*,8*S*,9*S*,10*R*,13*S*,14*R*,15*R*,16*S*)-6-benzoyloxy-15,16-epoxy-5,7-dihydroxy-13-spirocassan-12,16-olide (spirocaesalmin G) (**4a**). 46

Figure S47. HRMS spectrum of (+)-(5*R*,6*R*,7*R*,8*S*,9*S*,10*R*,13*S*,14*R*,15*R*,16*S*)-6-benzoyloxy-15,16-epoxy-5,7-dihydroxy-13-spirocassan-12,16-olide (spirocaesalmin G) (**4a**). 47

Figure S48. ^{1}H NMR spectrum of (+)-(5*R*,6*R*,7*R*,8*S*,9*S*,10*R*,13*S*,14*R*,15*R*,16*S*)-6-benzoyloxy-15,16-epoxy-5,7-dihydroxy-13-spirocassan-12,16-olide (isospirocaesalmin G) (**4b**), (400 MHz, $CDCl_3$). 48

Figure S49. COSY spectrum of (−)-(5*R*,6*R*,7*R*,8*S*,9*S*,10*R*,13*R*,14*R*,15*S*,16*R*)-6-benzoyloxy-15,16-epoxy-5,7-dihydroxy-13-spirocassan-12,16-olide (isospirocaesalmin G) (**4b**), (400 MHz, $CDCl_3$). 49

Figure S50. NOESY spectrum of (−)-(5*R*,6*R*,7*R*,8*S*,9*S*,10*R*,13*R*,14*R*,15*S*,16*R*)-6-benzoyloxy-15,16-epoxy-5,7-dihydroxy-13-spirocassan-12,16-olide (isospirocaesalmin G) (**4b**), (400 MHz, $CDCl_3$). 50

Figure S51. ^{13}C NMR spectrum of (−)-(5*R*,6*R*,7*R*,8*S*,9*S*,10*R*,13*R*,14*R*,15*S*,16*R*)-6-benzoyloxy-15,16-epoxy-5,7-dihydroxy-13-spirocassan-12,16-olide (isospirocaesalmin G) (**4b**), (100 MHz, $CDCl_3$). 51

Figure S52. HMQC spectrum of (−)-(5*R*,6*R*,7*R*,8*S*,9*S*,10*R*,13*R*,14*R*,15*S*,16*R*)-6-benzoyloxy-15,16-epoxy-5,7-dihydroxy-13-spirocassan-12,16-olide (isospirocaesalmin G) (**4b**), (400/100 MHz, $CDCl_3$). 52

Figure S53. HMBC spectrum of (−)-(5*R*,6*R*,7*R*,8*S*,9*S*,10*R*,13*R*,14*R*,15*S*,16*R*)-6-benzoyloxy-15,16-epoxy-5,7-dihydroxy-13-spirocassan-12,16-olide (isospirocaesalmin G) (**4b**), (400/100 MHz, $CDCl_3$). 53

Figure S54. IR spectrum of (−)-(5*R*,6*R*,7*R*,8*S*,9*S*,10*R*,13*R*,14*R*,15*S*,16*R*)-6-benzoyloxy-15,16-epoxy-5,7-dihydroxy-13-spirocassan-12,16-olide (isospirocaesalmin G) (**4b**). 54

Figure S55. HRMS spectrum of (−)-(5*R*,6*R*,7*R*,8*S*,9*S*,10*R*,13*R*,14*R*,15*S*,16*R*)-6-benzoyloxy-15,16-epoxy-5,7-dihydroxy-13-spirocassan-12,16-olide (isospirocaesalmin G) (**4b**). 55

Figure S56. Effect of compounds **1**, **1a**, **1b**, **2**, **2a**, **2b**, **3**, **3a**, **3b**, **4**, **4a**, and **4b** on HeLa cell viability. 56

Figure S57. Dose-response effect of compounds **1-4**, **1a**, **1b**, **2a**, **2b**, **3a**, **3b**, **4a**, and **4b** on HeLa cell viability. 57

Figure S58. Effect of compounds **1**, **1a**, **1b**, **2**, **2a**, **2b**, **3**, **3a**, **3b**, **4**, **4a**, and **4b** on MDA-MB-231 cell viability. 58

Figure S59. Dose-response effect of compounds **1-4**, **1a**, **1b**, **2a**, **2b**, **3a**, **3b**, **4a**, and **4b** on MDA-MB-231 cell viability. 59

Figure S60. Effect of compounds **1**, **1a**, **1b**, **2**, **2a**, **2b**, **3**, **3a**, **3b**, **4**, **4a**, and **4b** on CCD-18Co Cell viability. 60

Figure S61. Dose-response effect of compounds **1-4**, **1a**, **1b**, **2a**, **2b**, **3a**, **3b**, **4a**, and **4b** on CCD-18Co cell viability. 61

Figure S62. Morphological changes in HeLa cells, induced by compounds **1**, **1a**, **1b**, **2**, **2a**, **2b**, **3**, **3a**, **3b**, **4**, **4a**, and **4b**. 62

Figure S63. Morphological changes in MDA-MB-231 cells, induced by compounds **1**, **1a**, **1b**, **2a**, **2b**, **3**, **3a**, **3b**, **4**, **4a**, and **4b**. 63

Figure S64. Morphological changes in CCD-18Co cells, induced by compounds **1**, **1a**, **1b**, **2**, **2a**, **2b**, **3**, **3a**, **3b**, **4**, **4a**, and **4b**. 64

Figure S65. Induction of apoptosis in HeLa cells by compounds **1a**, **1b**, **2a**, and **4a** 65

Figure S66. Induction of apoptosis in MDA-MB-231 cells by compounds **1b**, **2a**, **3**, and **3b**. 66

Figure S67. Docking interactions of N-{(2S)-3-[(3S)-8',9'-dihydro-1H,3'H-spiro[piperidine-3,7'-pyrano[3,2-e]indazol]-1-yl]-2-hydroxypropyl}-N-(2-ethoxyethyl)-2,6-dimethylbenzenesulfonamide in the allosteric modulation ATP-binding site on Akt protein. 67

Figure S68. Calculated conformers of **1a**, **1b**, **2a**, **2b**, **3a**, **3b**, **4a**, and **4b** at the DFT B3LYP DGDZVP basis set 69

Table S1. Thermochemical analysis of (+)-(5*R*,6*R*,7*R*,8*S*,9*S*,10*R*,13*S*,14*R*,15*R*,16*S*)-6-benzoyloxy-15,16-epoxy-5,7-dihydroxy-13-spirocassan-12,16-olide (**4a**), and (−)-(5*R*,6*R*,7*R*,8*S*,9*S*,10*R*,13*R*,14*R*,15*S*,16*R*)-6-benzoyloxy-15,16-epoxy-5,7-dihydroxy-13-spirocassan-12,16-olide (**4b**) 70

Table S2. Cytotoxicity IC_{50} in µg/mL of vouacapane derivatives (**2**-**4**) and epoxyspirolactones (**1a**-**4a** and **1b**-**4b**) in human cell lines 71

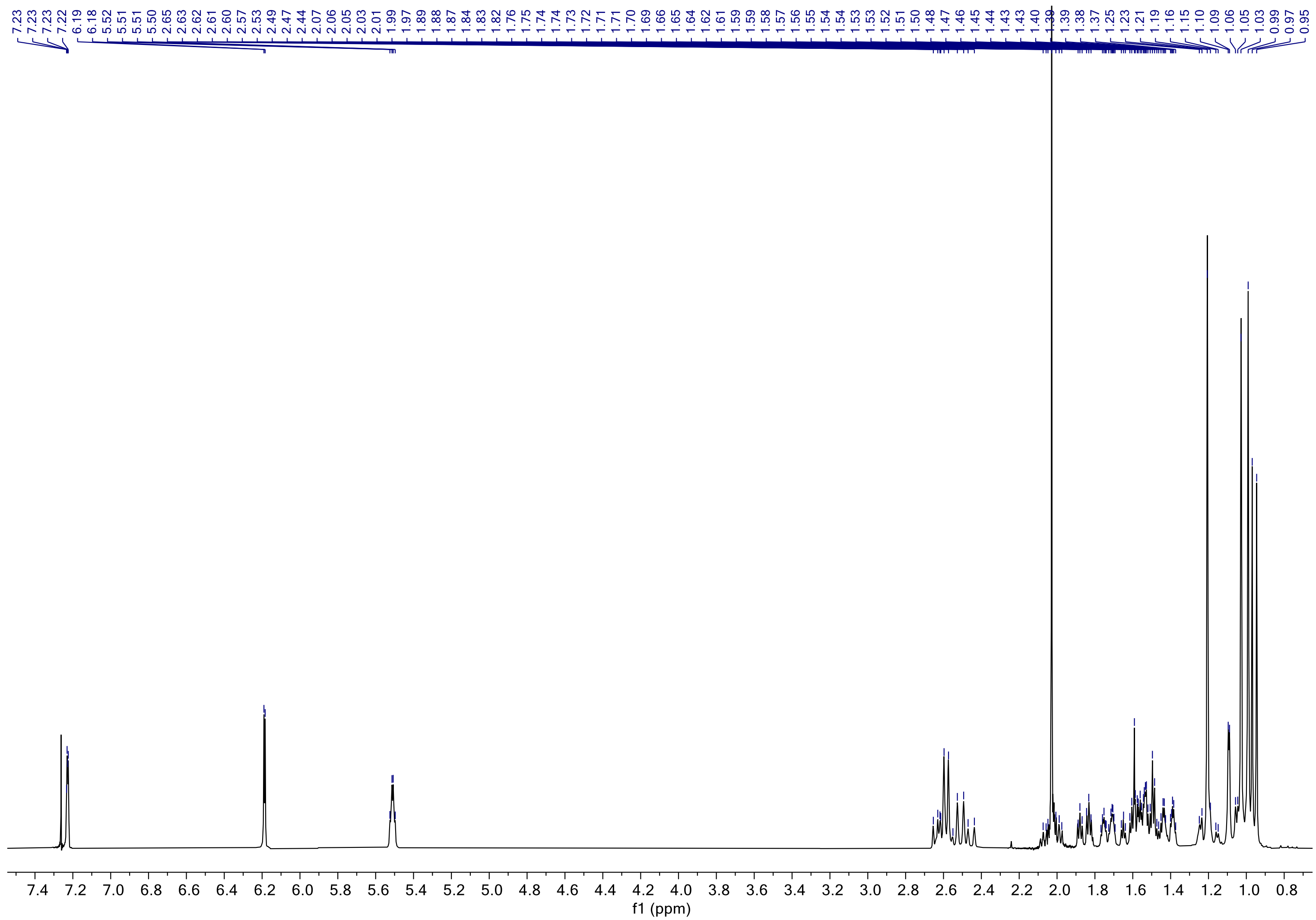

Figure S1. ^{1}H NMR spectrum of 6β-acetoxyvouacapane (**1**), (400 MHz, $CDCl_3$).

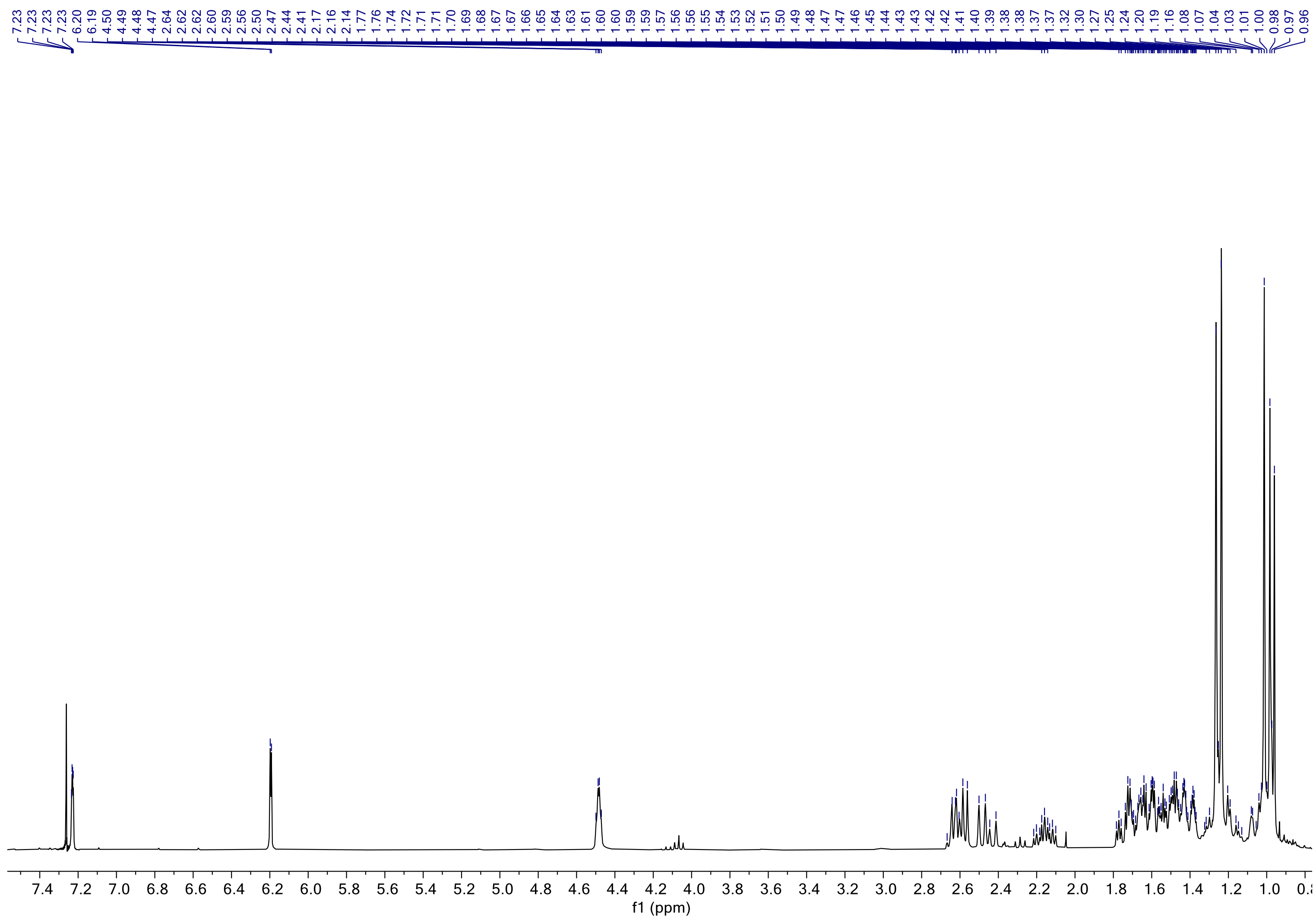

Figure S2. ^{1}H NMR spectrum of 6β-hydroxyvouacapane (**2**), (400 MHz, $CDCl_3$).

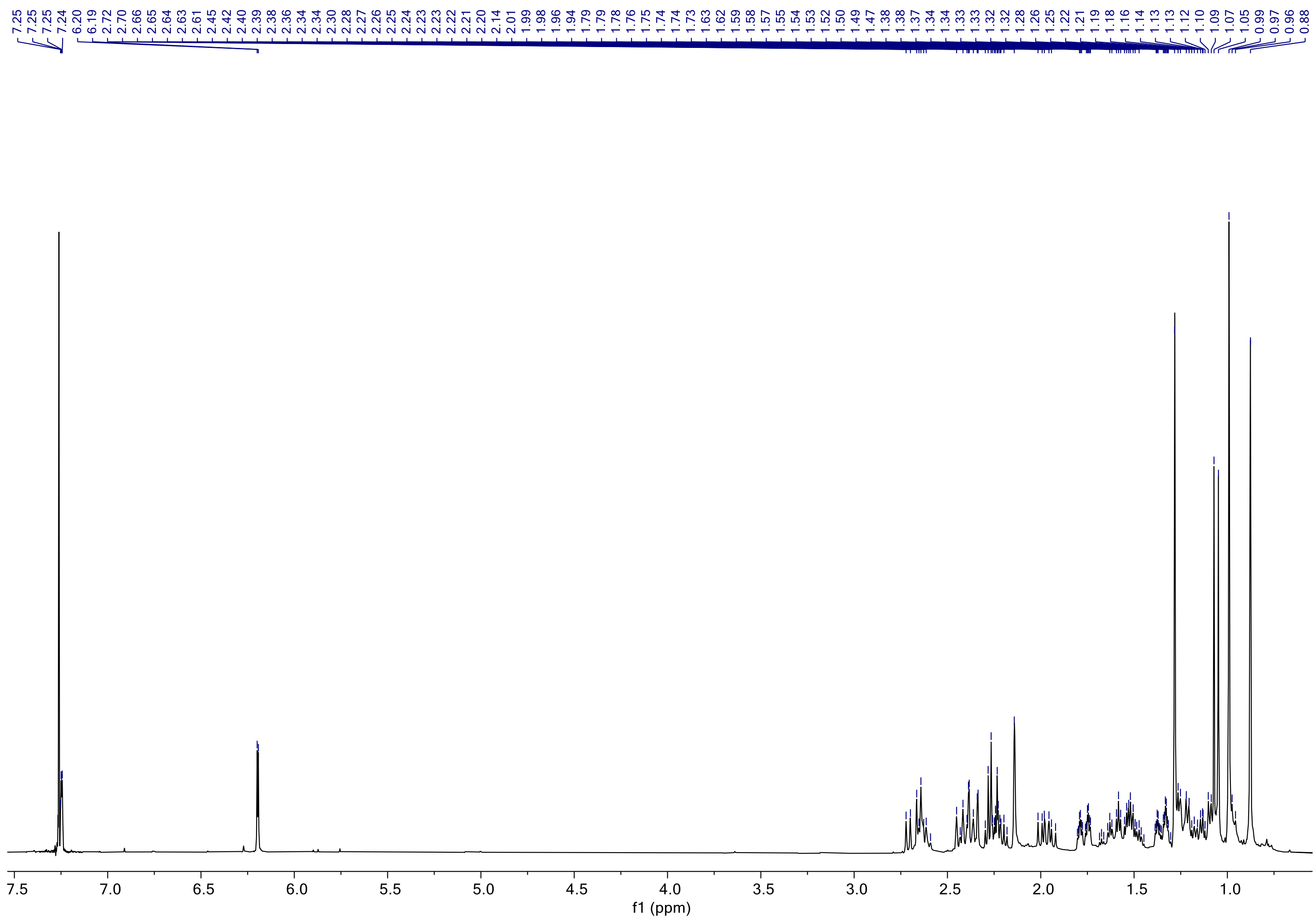

Figure S3. ^{1}H NMR spectrum of 6-oxovouacapane (**3**), (400 MHz, $CDCl_3$).

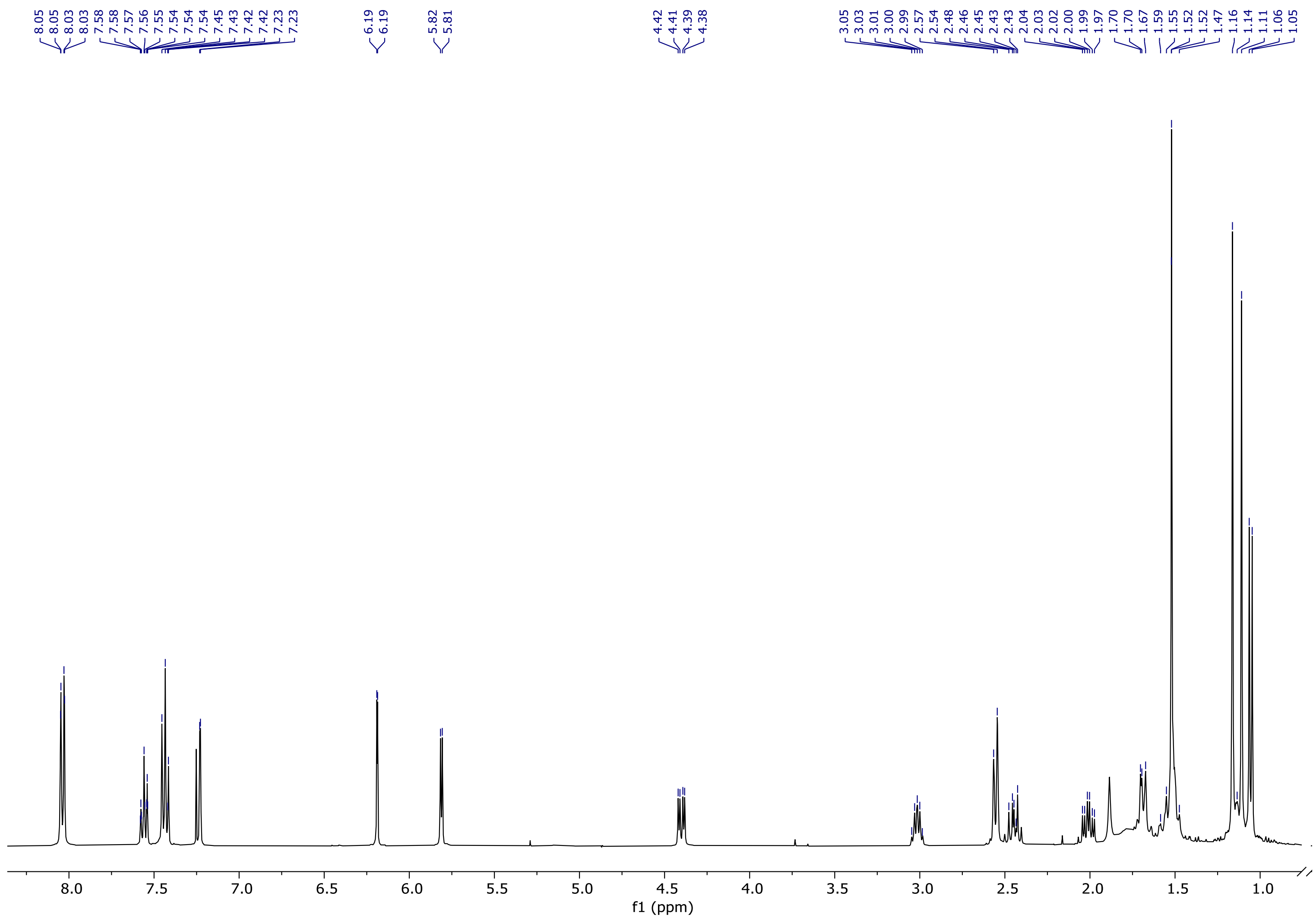

Figure S4. ^{1}H NMR spectrum of isovouacapenol C (**4**), (400 MHz, $CDCl_3$).

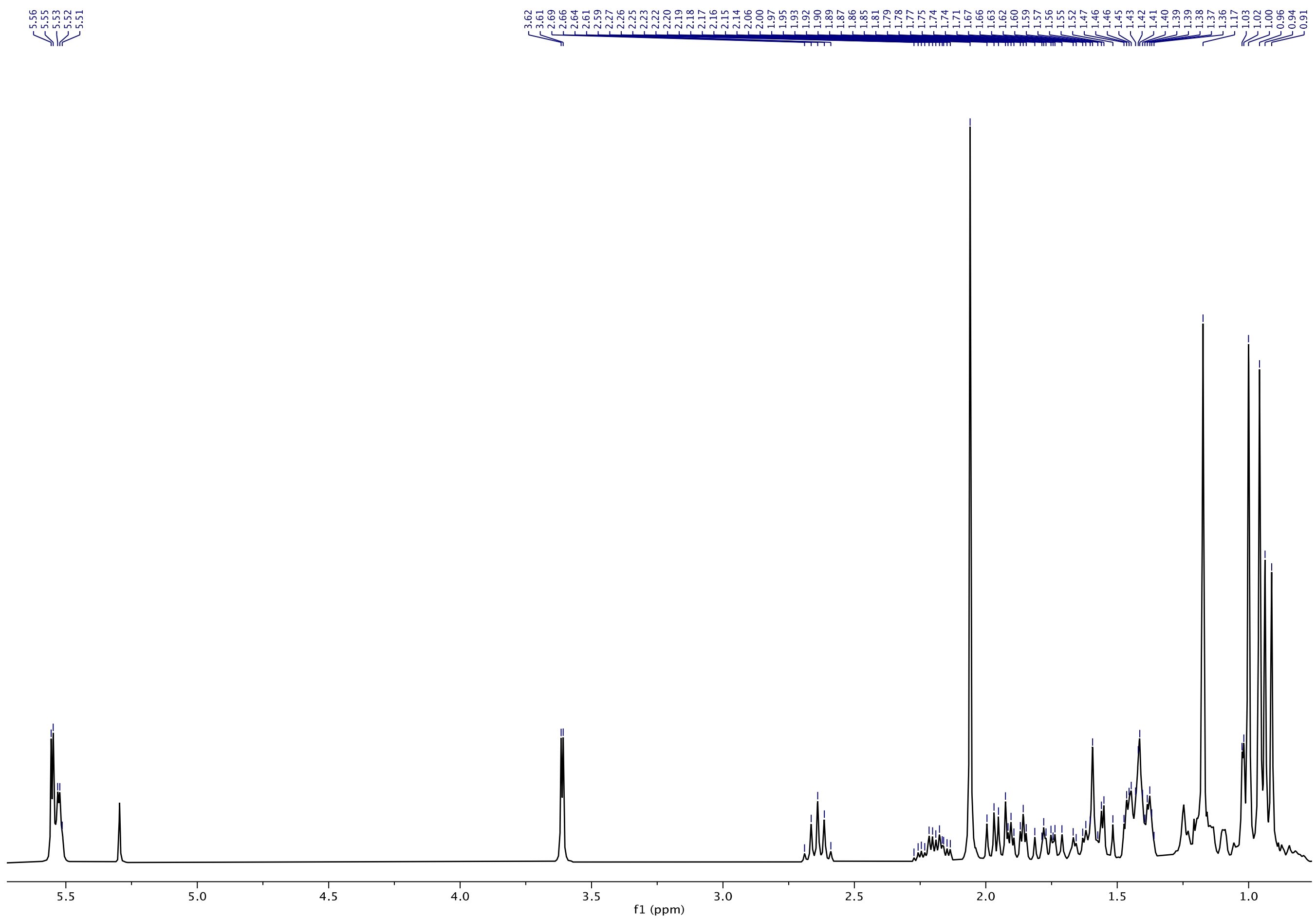

Figure S5. ^{1}H NMR spectrum of (+)-(5*S*,6*R*,8*S*,9*S*,10*R*,13*S*,14*R*,15*R*,16*S*)-6-acetoxy-15,16-epoxy-13-spirocassan-12,16-olide (spirocaesalmin D) (**1a**), (300 MHz, $CDCl_3$).

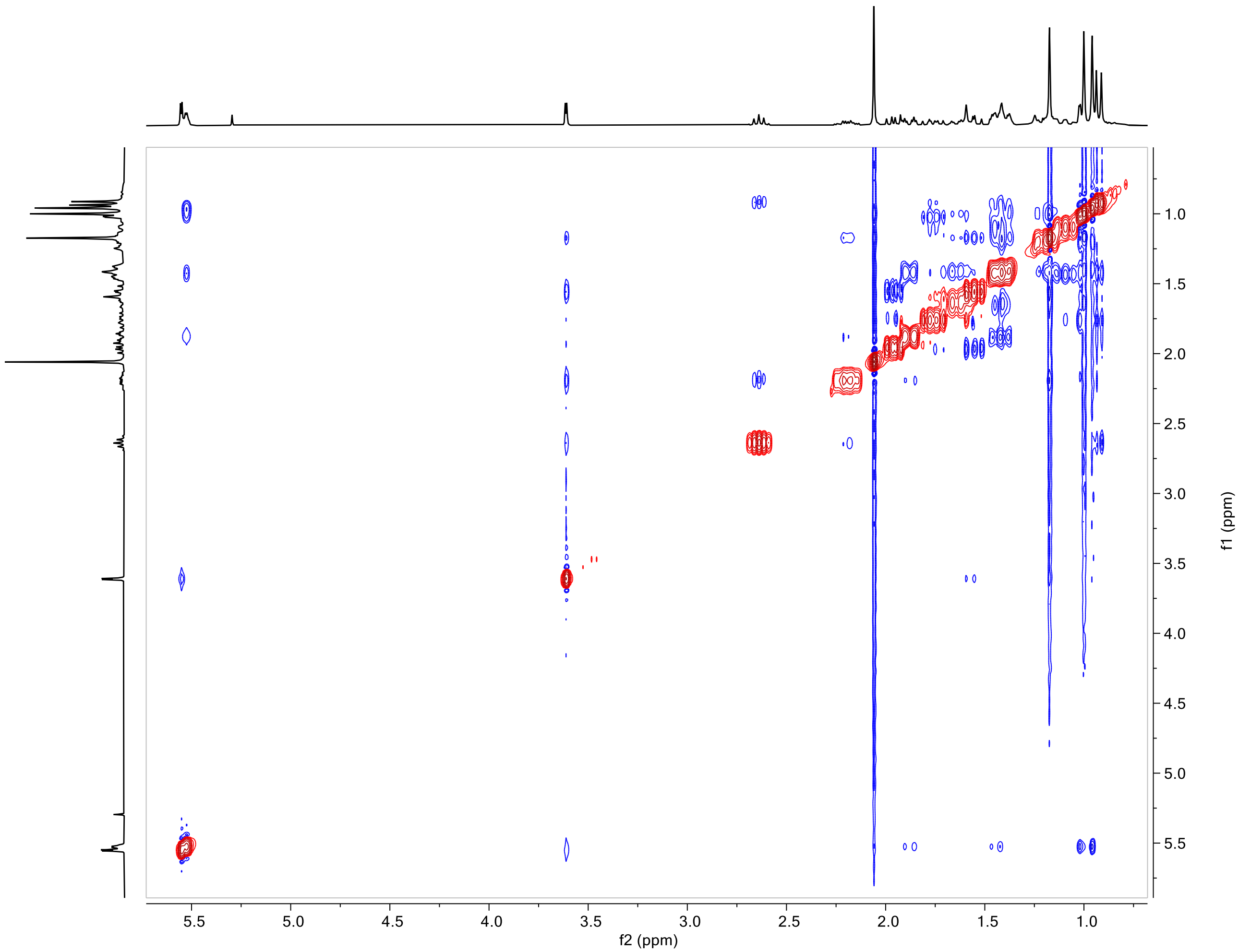

Figure S6. NOESY spectrum of (+)-(5*S*,6*R*,8*S*,9*S*,10*R*,13*S*,14*R*,15*R*,16*S*)-6-acetoxy-15,16-epoxy-13-spirocassan-12,16-olide (spirocaesalmin D) (**1a**), (300 MHz, $CDCl_3$).

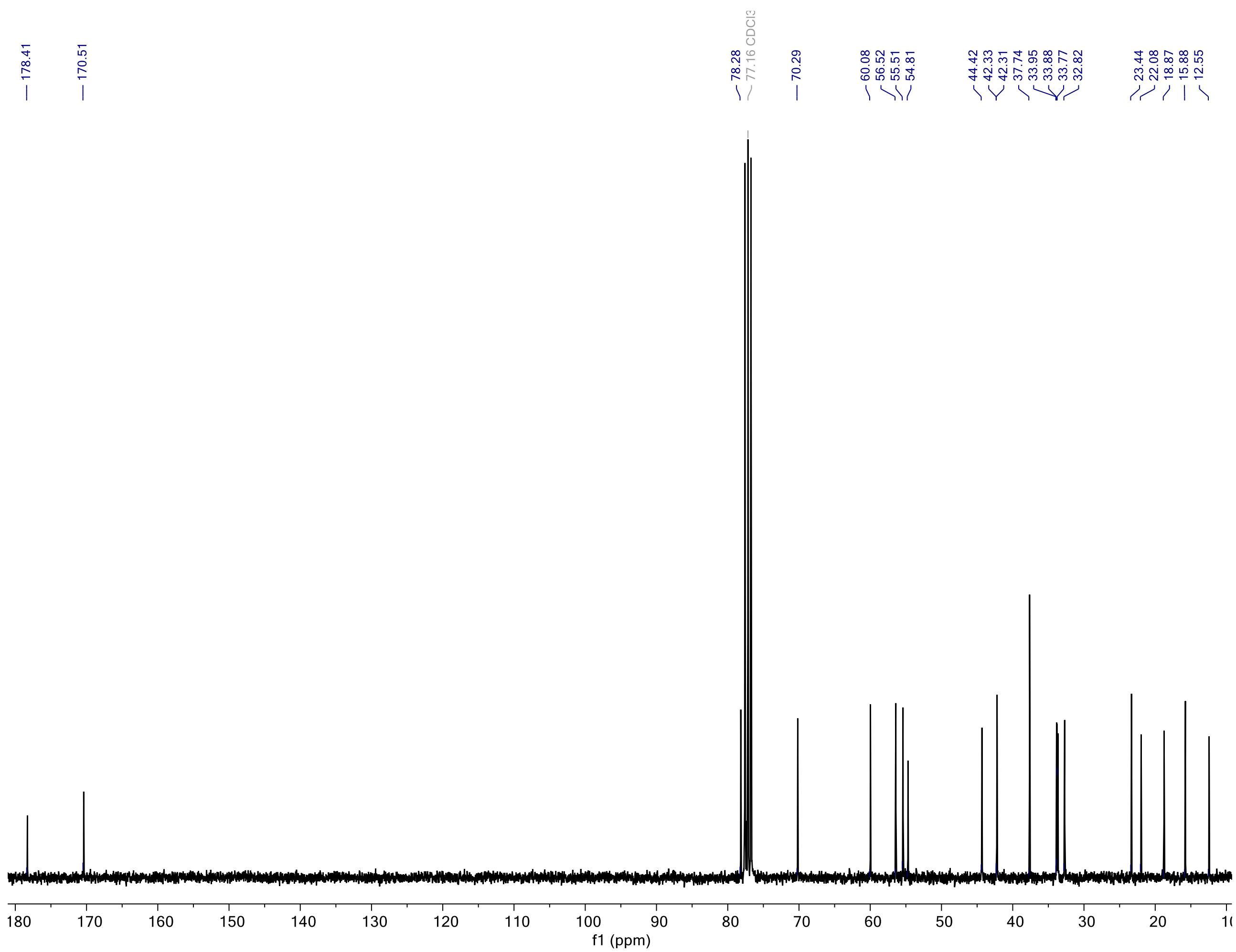

Figure S7. ^{13}C NMR spectrum of (+)-(5*S*,6*R*,8*S*,9*S*,10*R*,13*S*,14*R*,15*R*,16*S*)-6-acetoxy-15,16-epoxy-13-spirocassan-12,16-olide (spirocaesalmin D) (**1a**), (75 MHz, $CDCl_3$).

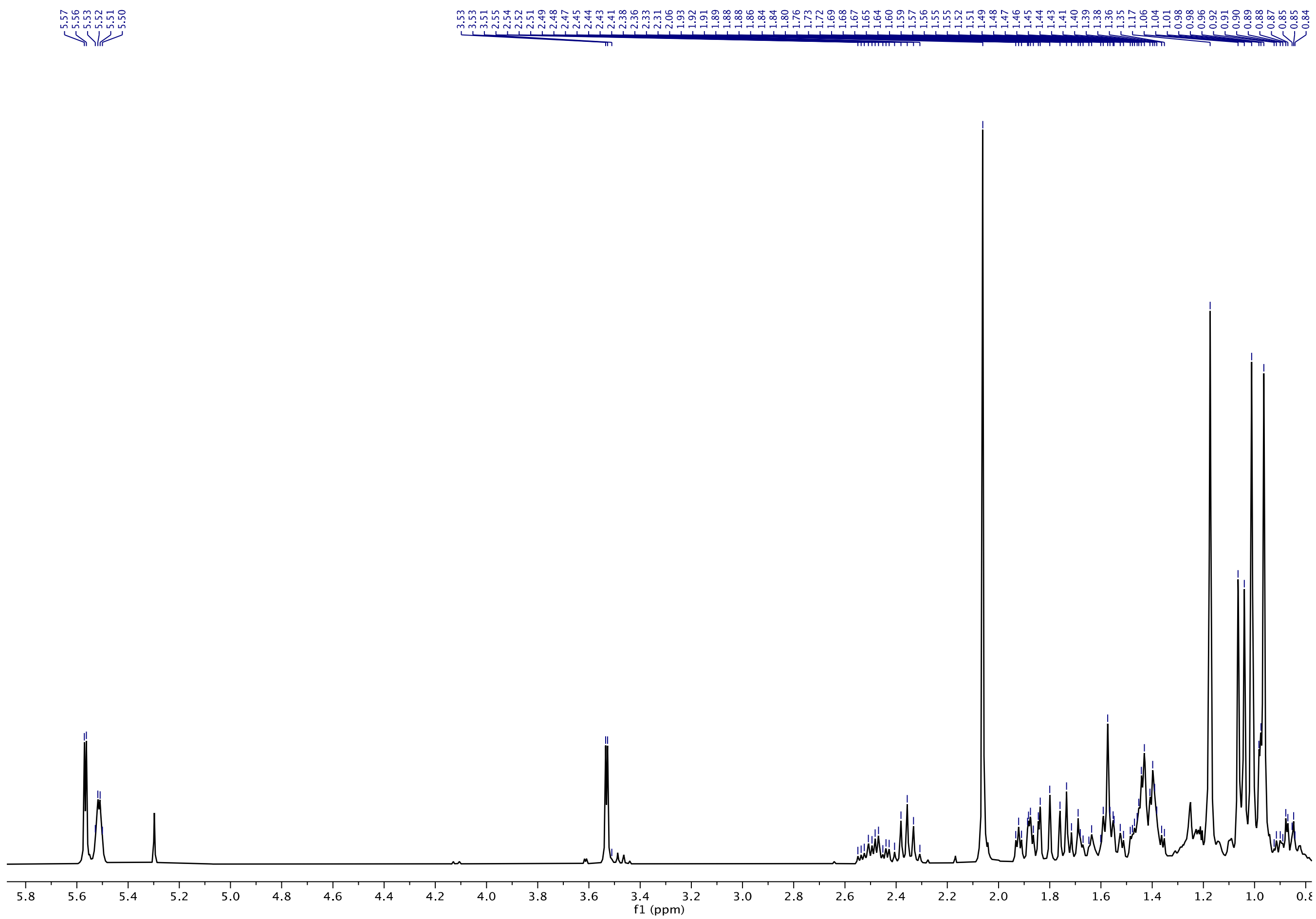

Figure S8. ^{1}H NMR spectrum of (−)-(5*S*,6*R*,8*S*,9*S*,10*R*,13*R*,14*R*,15*S*,16*R*)-6-acetoxy-15,16-epoxy-13-spirocassan-12,16-olide (isospirocaesalmin D) (**1b**), (300 MHz, $CDCl_3$).

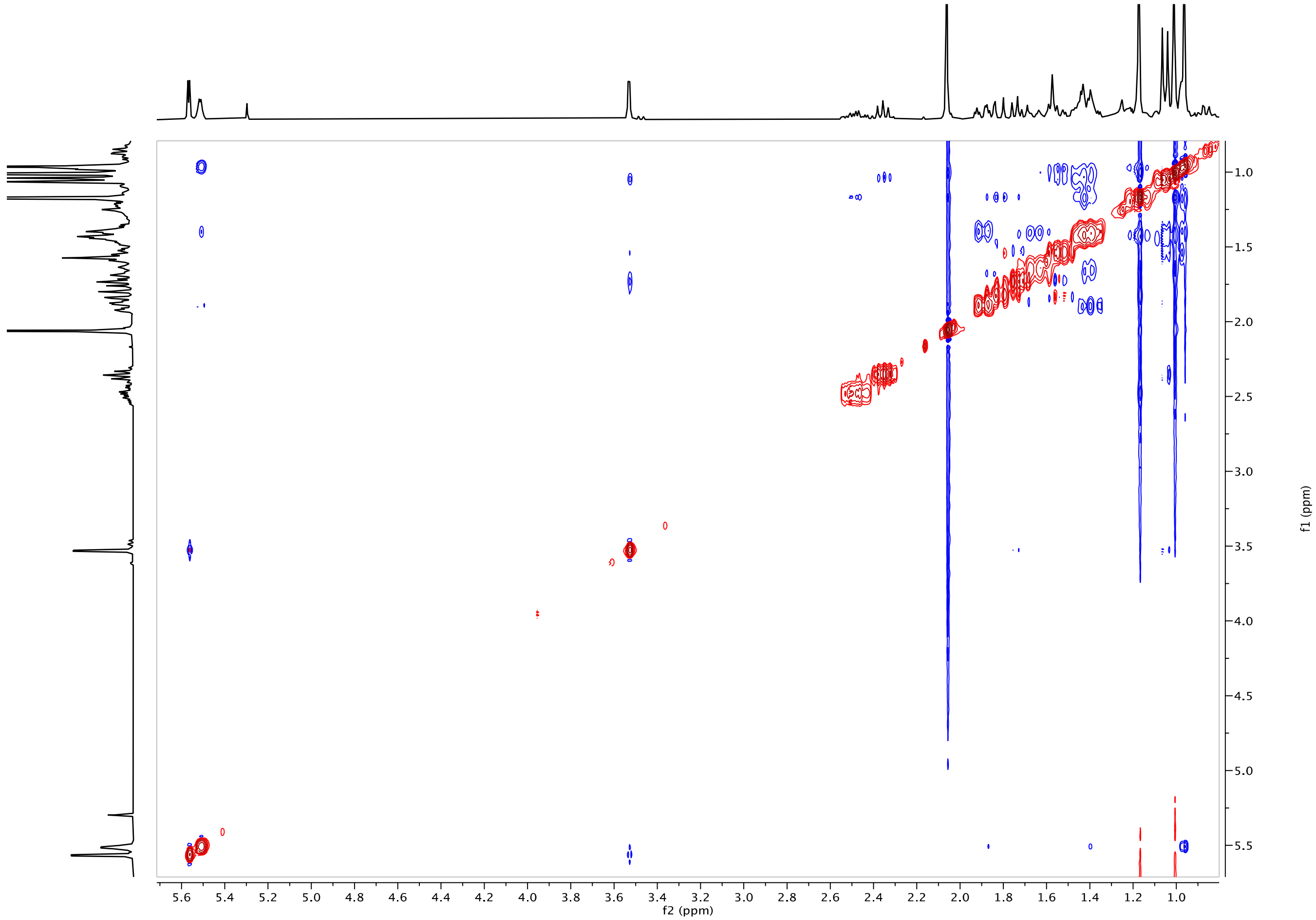

Figure S9. NOESY spectrum of (−)-(5*S*,6*R*,8*S*,9*S*,10*R*,13*R*,14*R*,15*S*,16*R*)-6-acetoxy-15,16-epoxy-13-spirocassan-12,16-olide (isospirocaesalmin D) (**1b**), (300 MHz, $CDCl_3$).

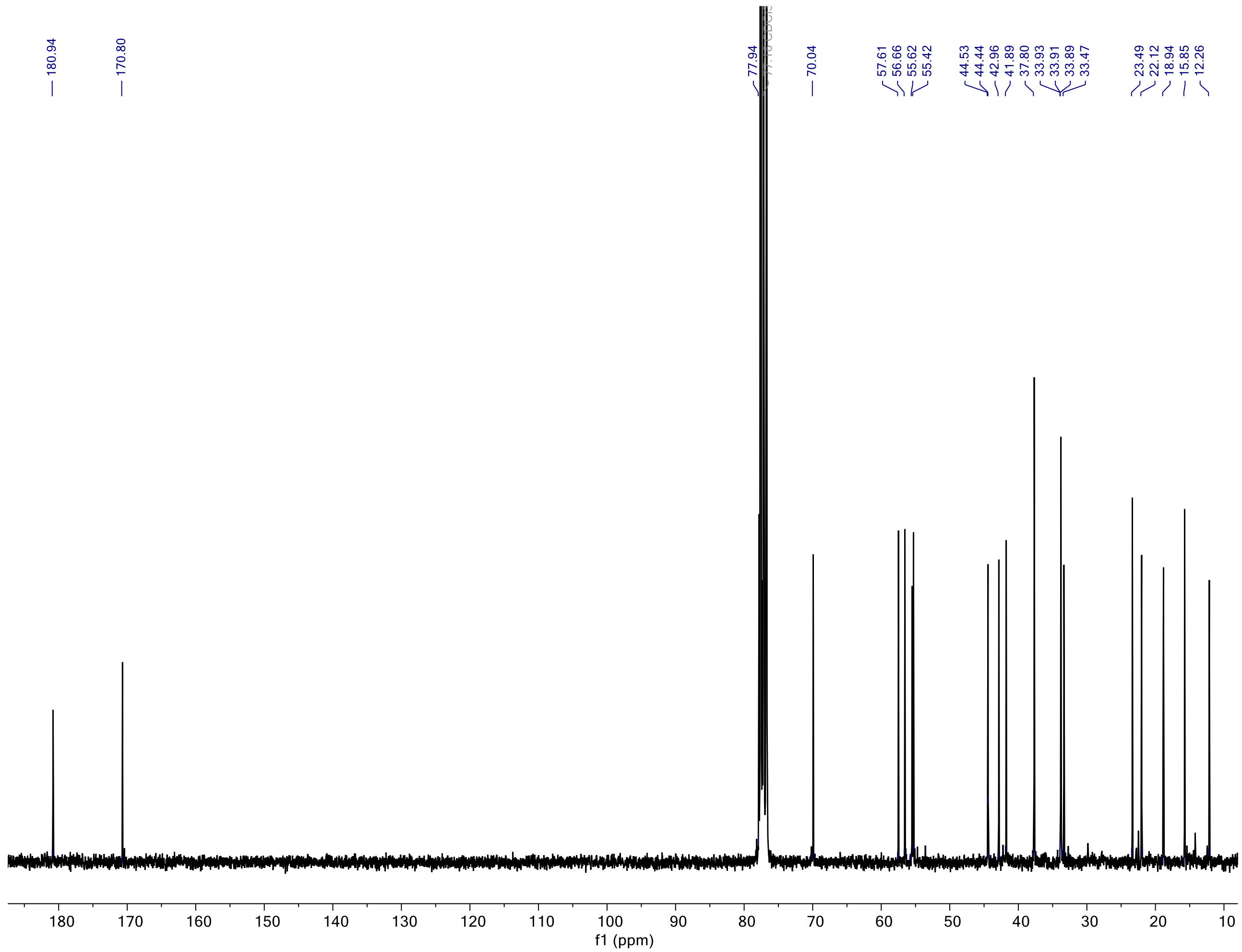

Figure S10. ^{13}C NMR spectrum of (−)-(5*S*,6*R*,8*S*,9*S*,10*R*,13*R*,14*R*,15*S*,16*R*)-6-acetoxy-15,16-epoxy-13-spirocassan-12,16-olide (isospirocaesalmin D) (**1b**), (75 MHz, $CDCl_3$).

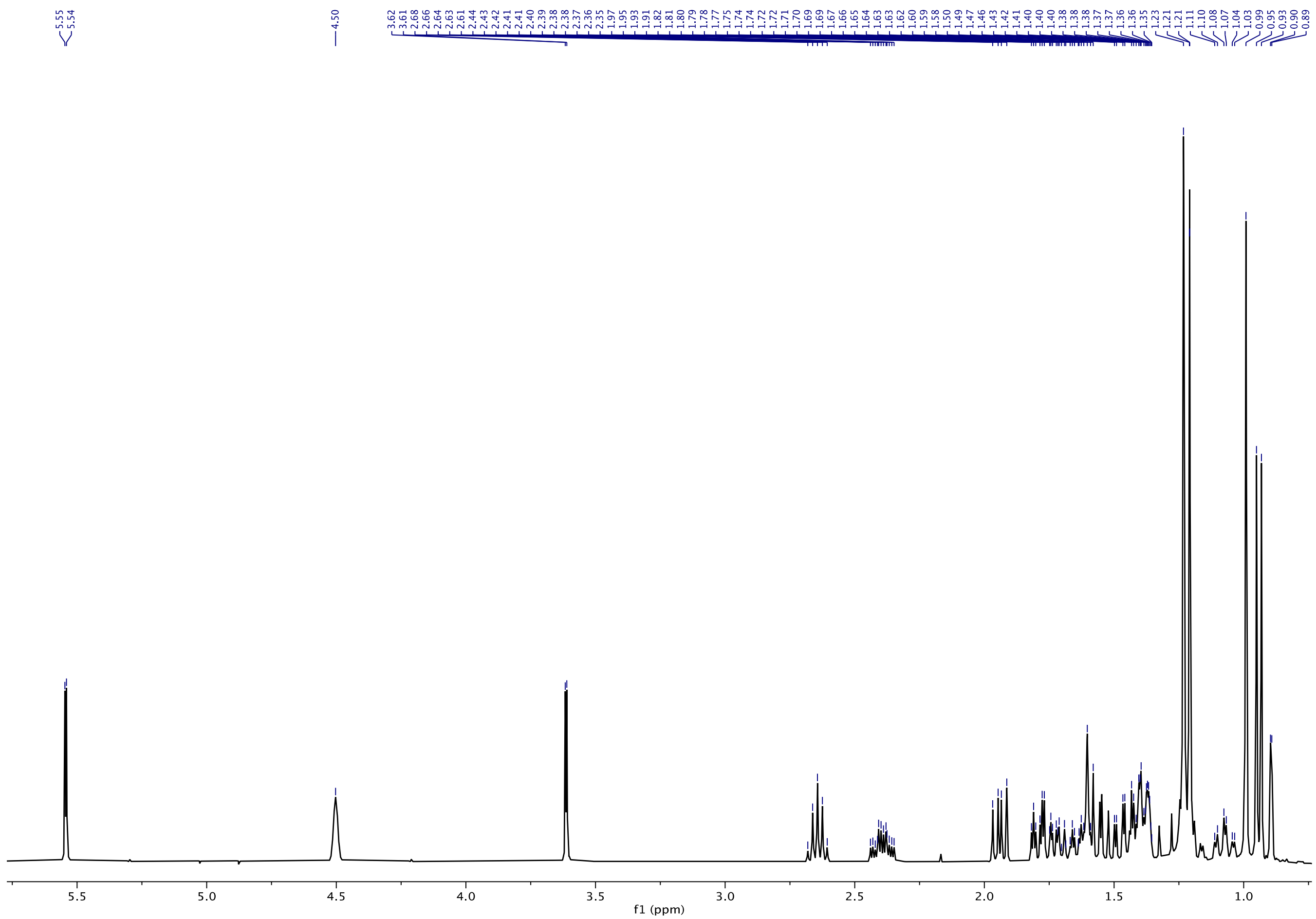

Figure S11. ^{1}H NMR spectrum of (+)-(5*S*,6*R*,8*S*,9*S*,10*R*,13*S*,14*R*,15*R*,16*S*)-15,16-epoxy-6-hydroxy-13-spirocassan-12,16-olide (spirocaesalmin E) (**2a**), (300 MHz, $CDCl_3$).

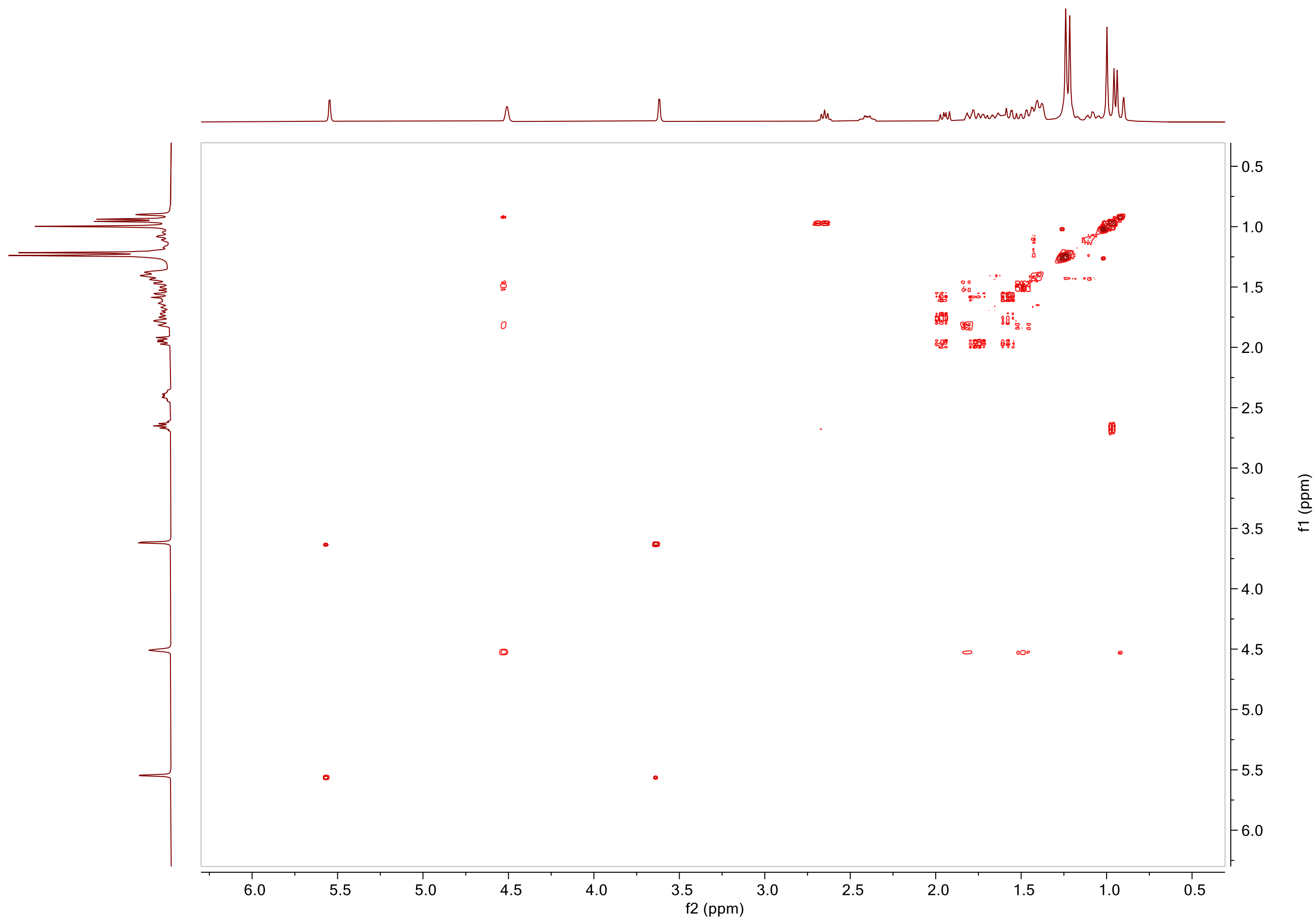

Figure S12. COSY spectrum of (+)-(5*S*,6*R*,8*S*,9*S*,10*R*,13*S*,14*R*,15*R*,16*S*)-15,16-epoxy-6-hydroxy-13-spirocassan-12,16-olide (spirocaesalmin E) (**2a**), (300 MHz, $CDCl_3$).

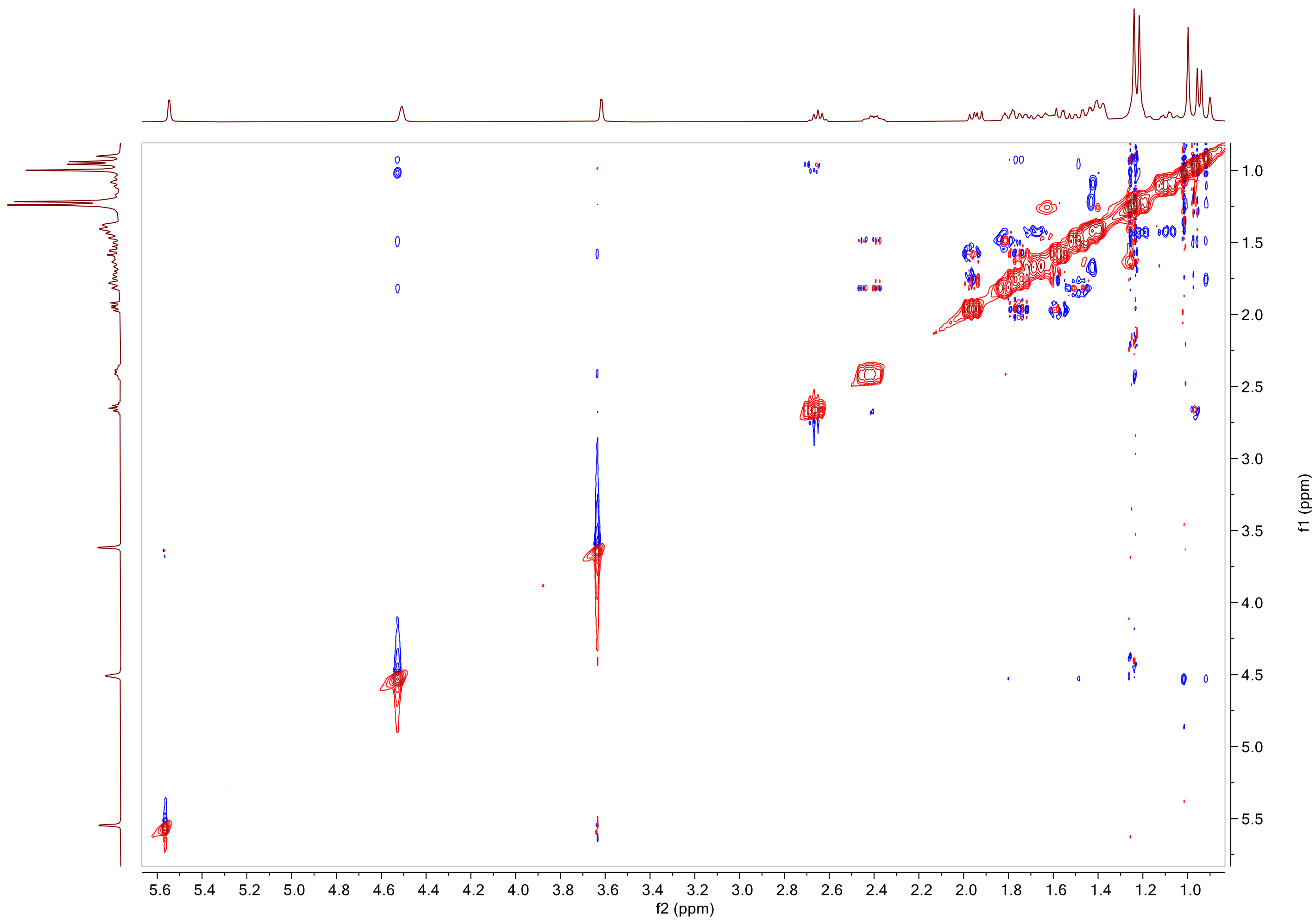

Figure S13. NOESY spectrum of (+)-(5*S*,6*R*,8*S*,9*S*,10*R*,13*S*,14*R*,15*R*,16*S*)-15,16-epoxy-6-hydroxy-13-spirocassan-12,16-olide (spirocaesalmin E) (**2a**), (300 MHz, $CDCl_3$).

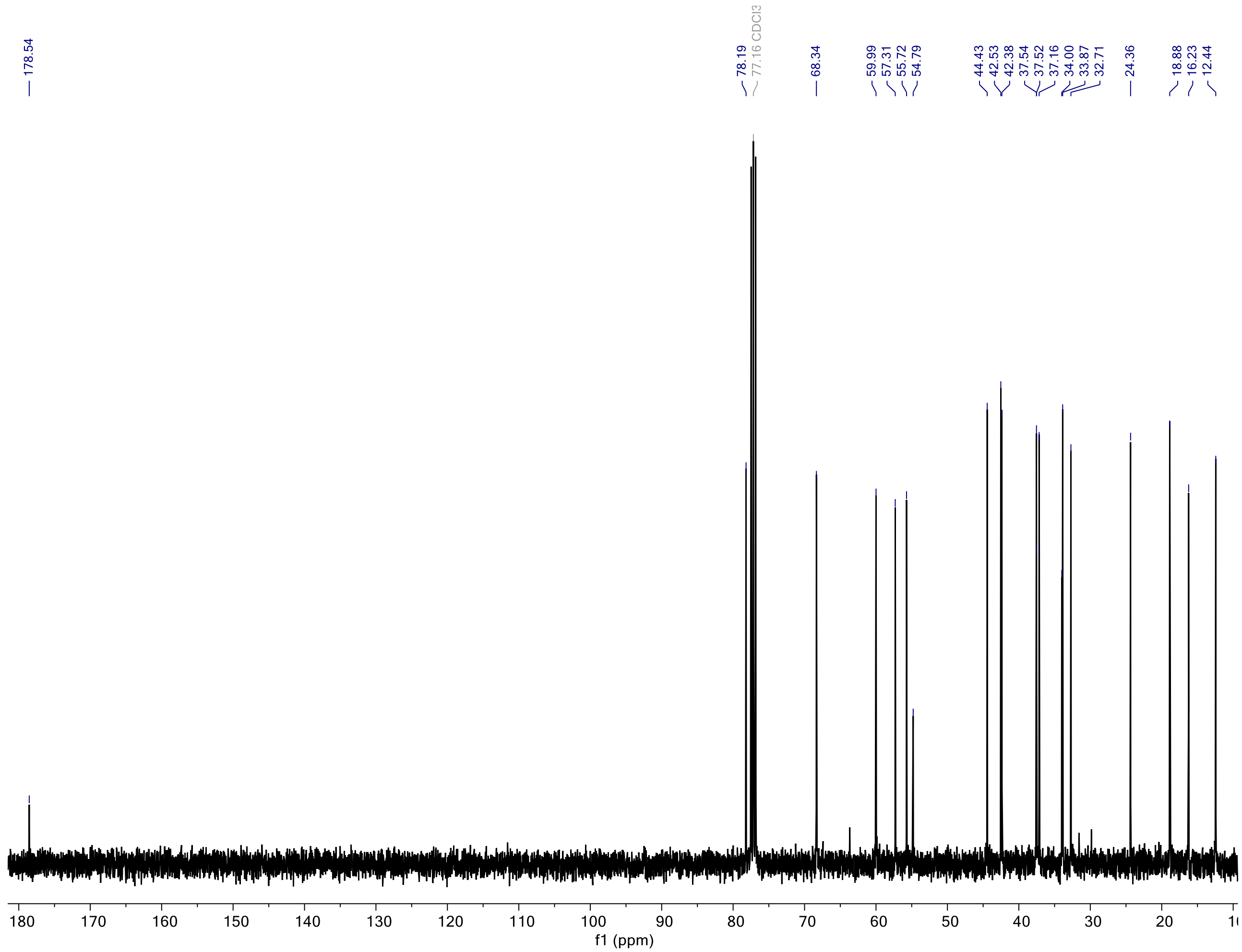

Figure S14. ^{13}C NMR spectrum of (+)-(5*S*,6*R*,8*S*,9*S*,10*R*,13*S*,14*R*,15*R*,16*S*)-15,16-epoxy-6-hydroxy-13-spirocassan-12,16-olide (spirocaesalmin E) (**2a**), (75 MHz, $CDCl_3$).

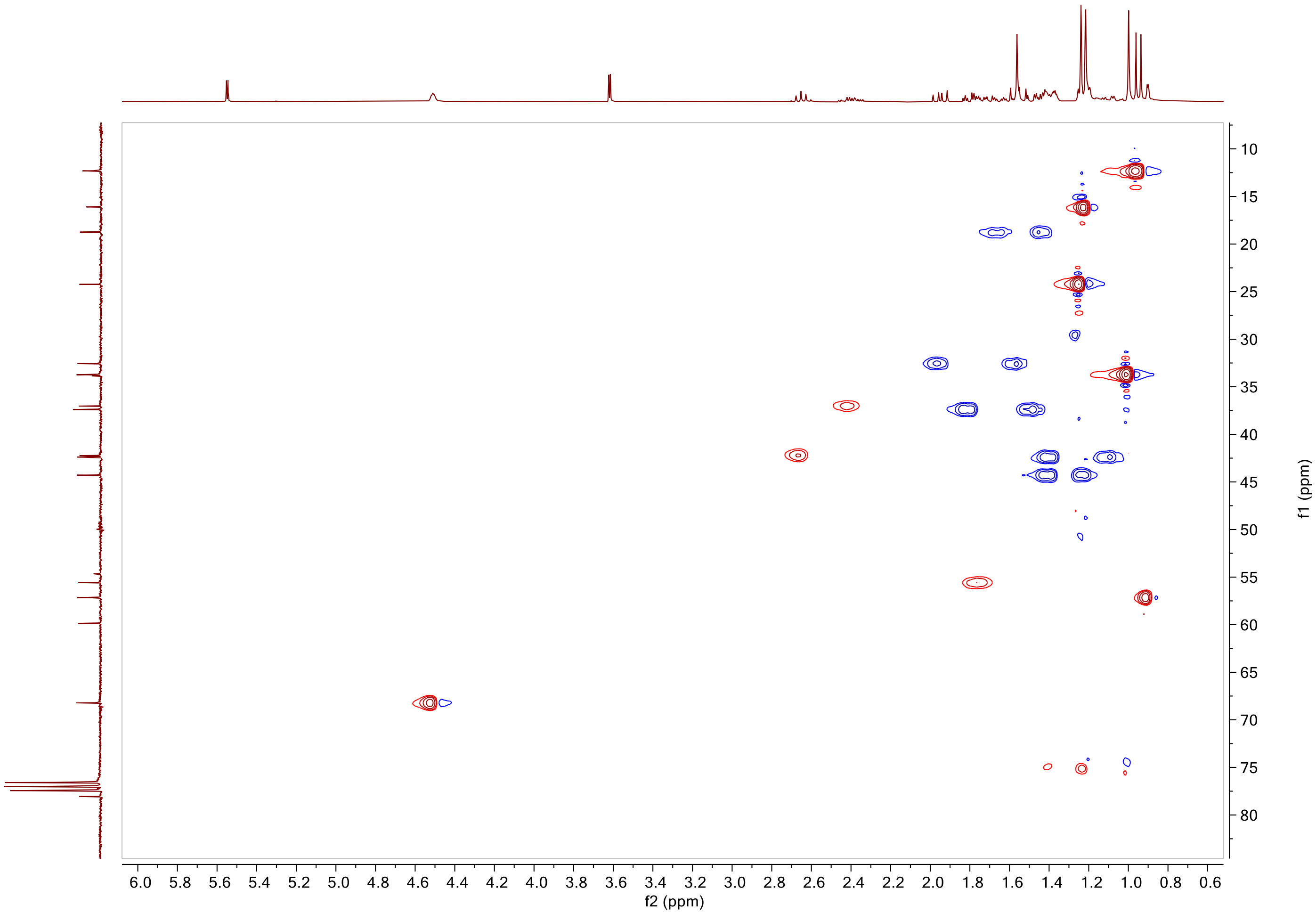

Figure S15. HSQC spectrum of (+)-(5*S*,6*R*,8*S*,9*S*,10*R*,13*S*,14*R*,15*R*,16*S*)-15,16-epoxy-6-hydroxy-13-spirocassan-12,16-olide (spirocaesalmin E) (**2a**), (300/75 MHz, $CDCl_3$).

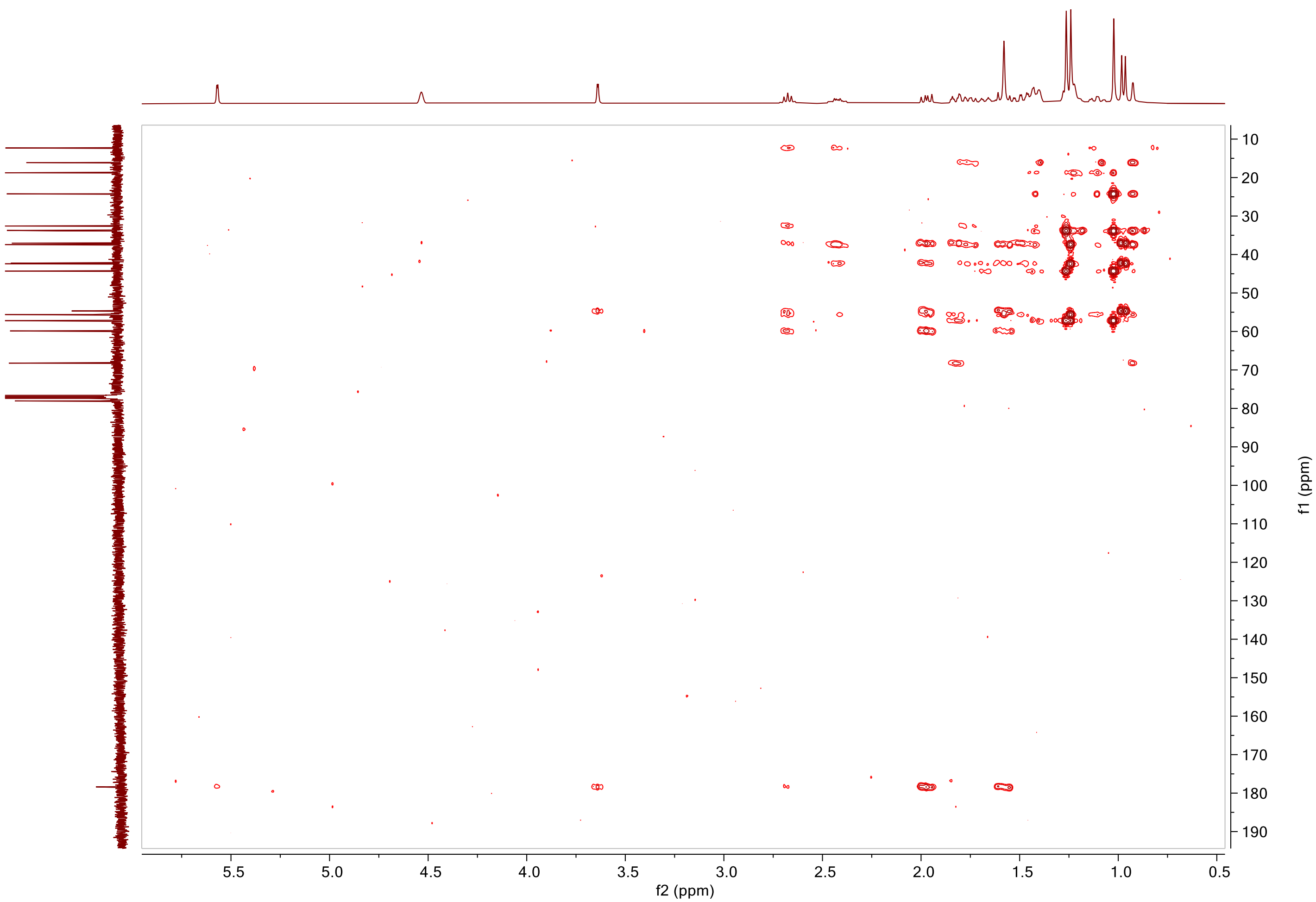

Figure S16. HMBC spectrum of (+)-(5*S*,6*R*,8*S*,9*S*,10*R*,13*S*,14*R*,15*R*,16*S*)-15,16-epoxy-6-hydroxy-13-spirocassan-12,16-olide (spirocaesalmin E) (**2a**), (300/75 MHz, $CDCl_3$).

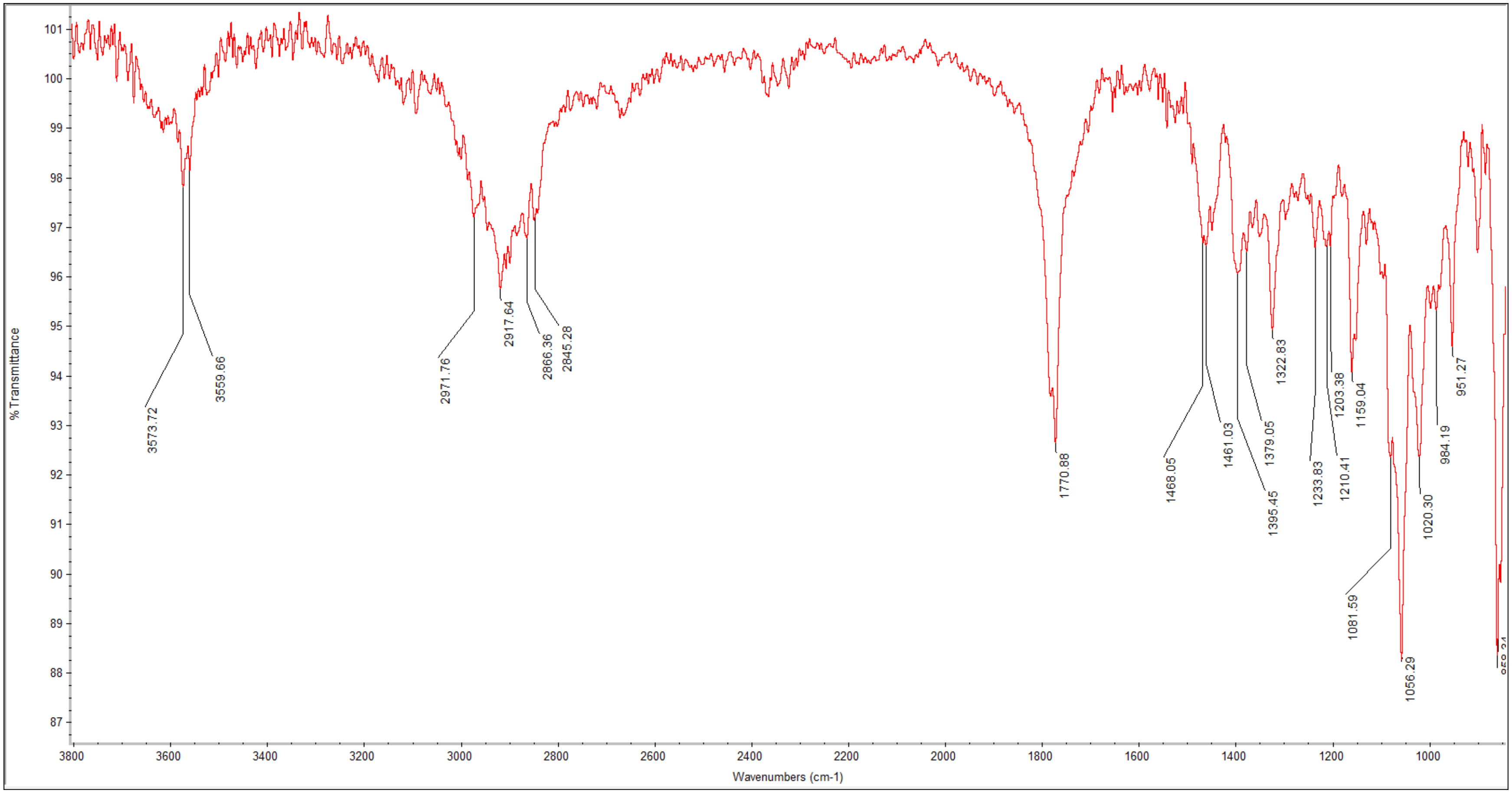

Figure S17. IR spectrum of (+)-(5*S*,6*R*,8*S*,9*S*,10*R*,13*S*,14*R*,15*R*,16*S*)-15,16-epoxy-6-hydroxy-13-spirocassan-12,16-olide (spirocaesalmin E) (**2a**).

Elemental Composition Report **Page 1**

Single Mass Analysis
Tolerance = 5.0 PPM / DBE: min = -1.5, max = 150.0
Element prediction: Off
Number of isotope peaks used for i-FIT = 3

Monoisotopic Mass, Even Electron Ions
149 formula(e) evaluated with 1 results within limits (all results (up to 1000) for each mass)
Elements Used:
C: 0-100 H: 0-100 O: 0-10 Na: 0-1 Br: 0-1

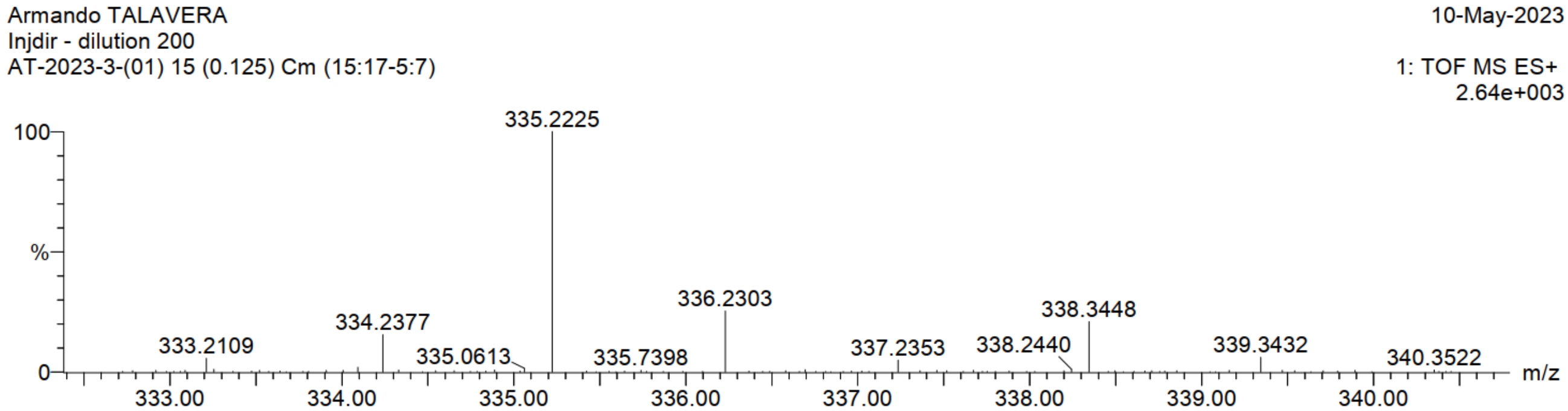

Minimum:				-1.5			
Maximum:		5.0	5.0	150.0			
Mass	Calc. Mass	mDa	PPM	DBE	i-FIT	i-FIT (Norm)	Formula
335.2225	335.2222	0.3	0.9	5.5	116.8	0.0	C20 H31 O4

Figure S18. HRMS spectrum of (+)-(5*S*,6*R*,8*S*,9*S*,10*R*,13*S*,14*R*,15*R*,16*S*)-15,16-epoxy-6-hydroxy-13-spirocassan-12,16-olide (spirocaesalmin E) (**2a**).

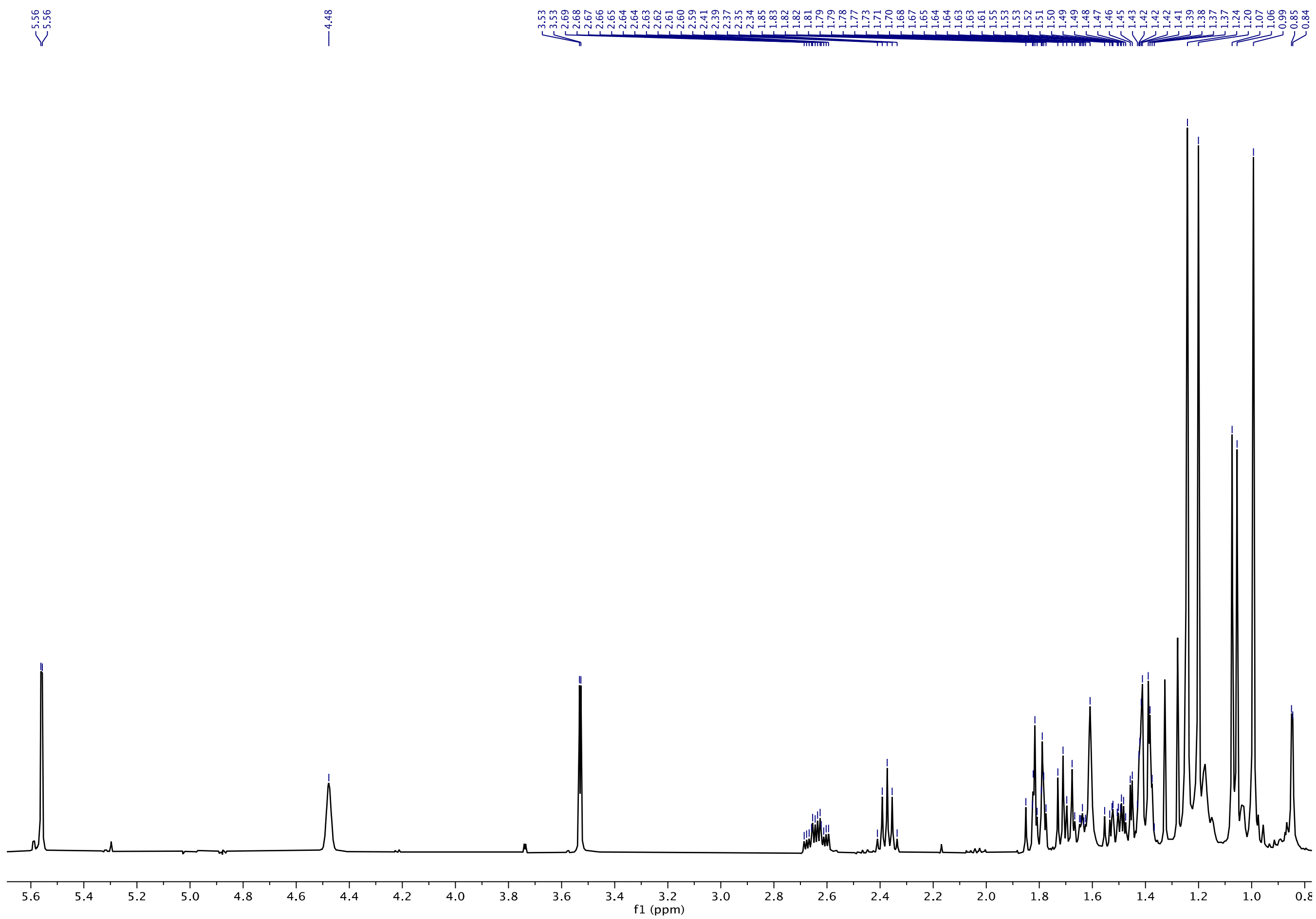

Figure S19. ^{1}H NMR spectrum of (−)-(5*S*,6*R*,8*S*,9*S*,10*R*,13*R*,14*R*,15*S*,16*R*)-15,16-epoxy-6-hydroxy-13-spirocassan-12,16-olide (isospirocaesalmin E) (**2b**), (300 MHz, $CDCl_3$).

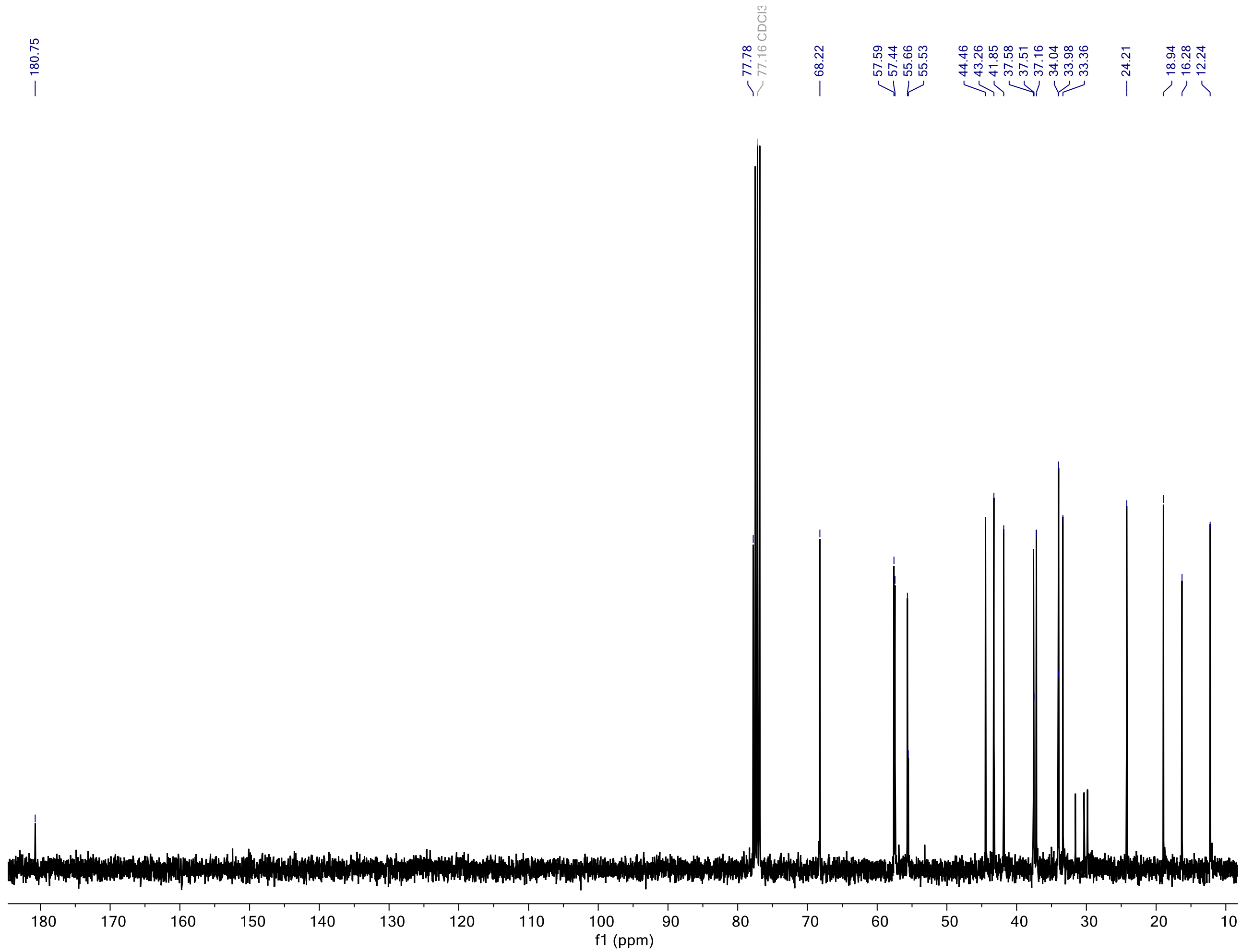

Figure S20. ^{13}C NMR spectrum of (−)-(5*S*,6*R*,8*S*,9*S*,10*R*,13*R*,14*R*,15*S*,16*R*)-15,16-epoxy-6-hydroxy-13-spirocassan-12,16-olide (isospirocaesalmin E) (**2b**), (75 MHz, $CDCl_3$).

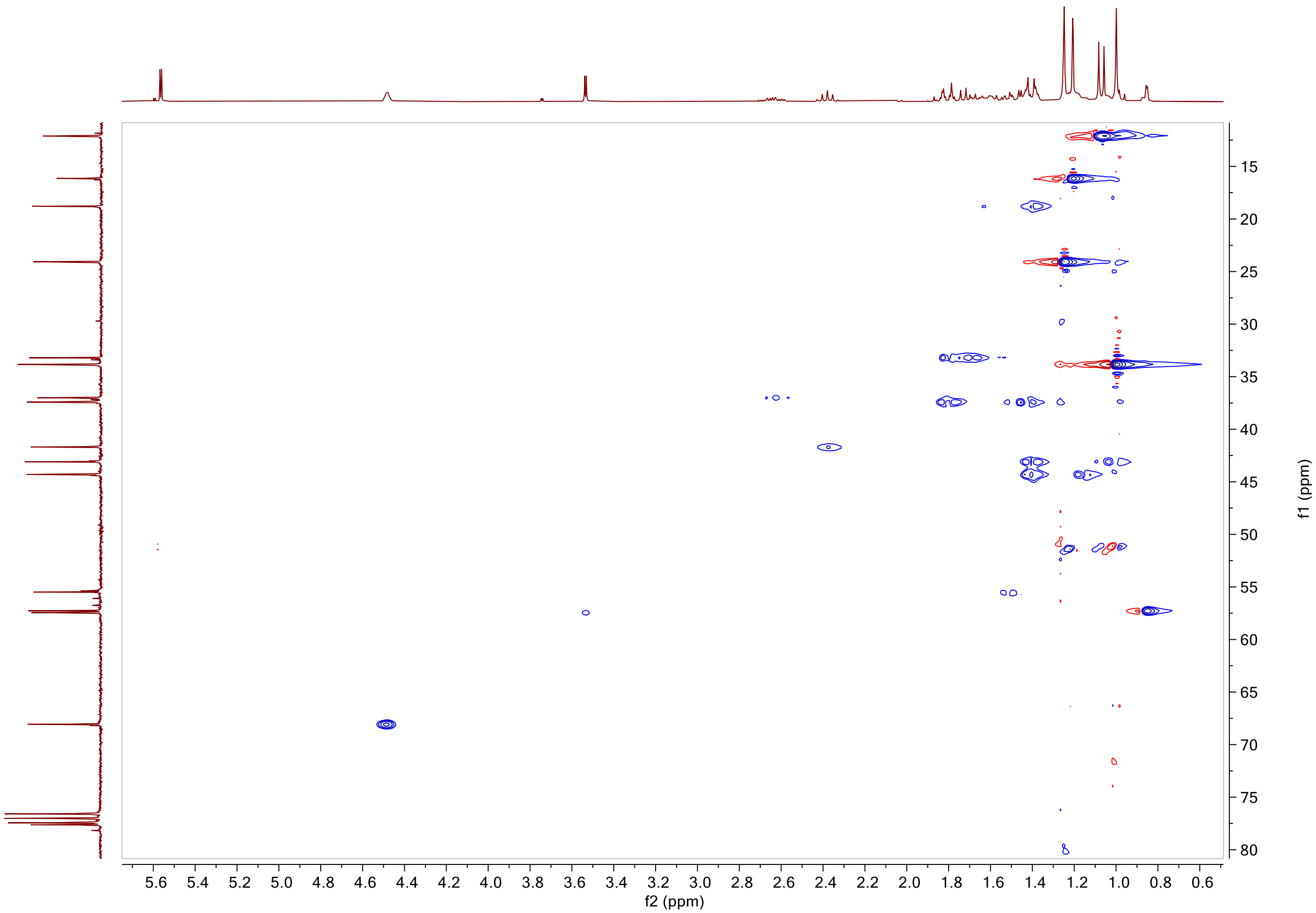

Figure S21. HSQC spectrum of (−)-(5*S*,6*R*,8*S*,9*S*,10*R*,13*R*,14*R*,15*S*,16*R*)-15,16-epoxy-6-hydroxy-13-spirocassan-12,16-olide (isospirocaesalmin E) (**2b**), (300/75 MHz, $CDCl_3$).

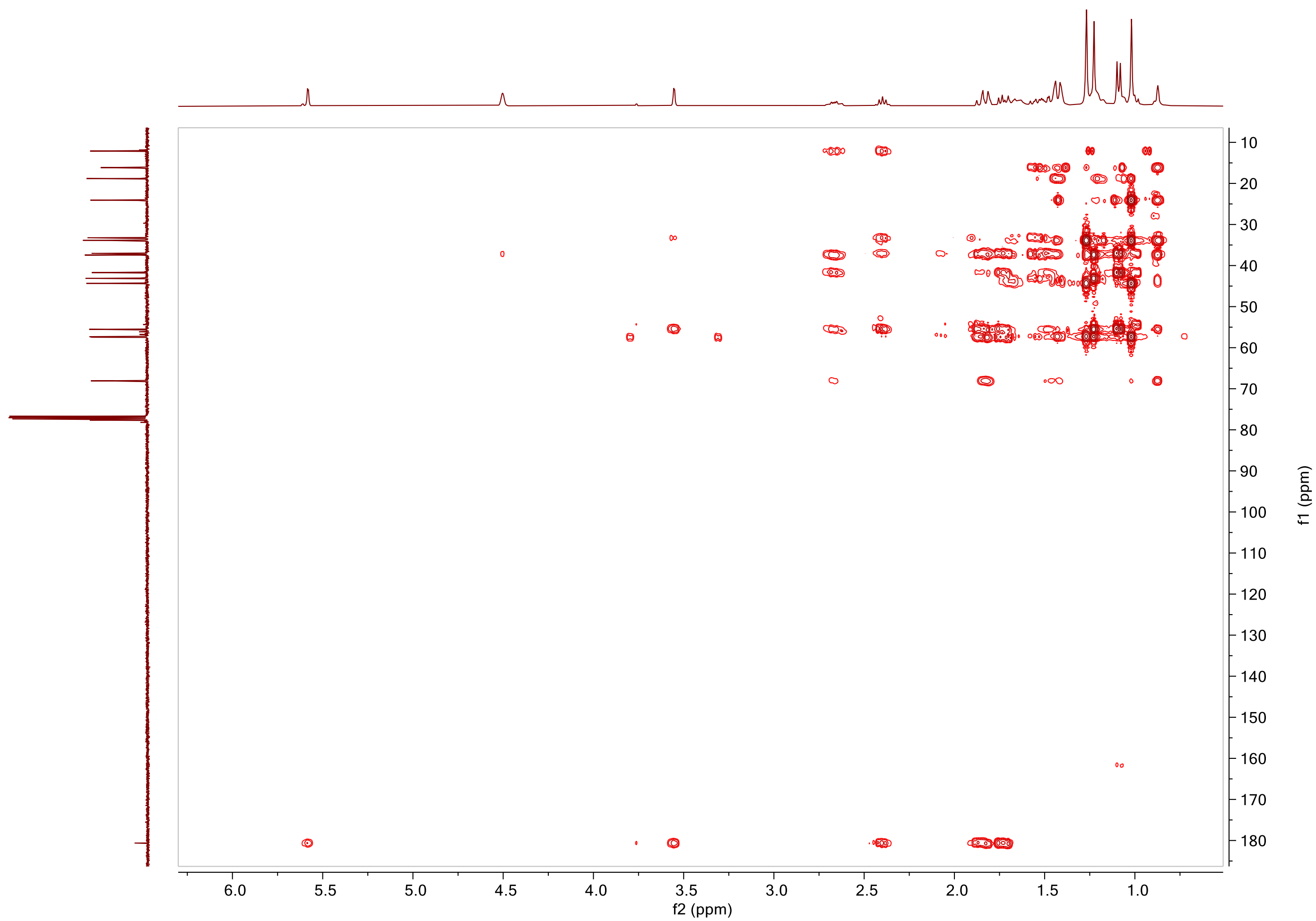

Figure S22. HMBC spectrum of (−)-(5*S*,6*R*,8*S*,9*S*,10*R*,13*R*,14*R*,15*S*,16*R*)-15,16-epoxy-6-hydroxy-13-spirocassan-12,16-olide (isospirocaesalmin E) (**2b**), (300/75 MHz, $CDCl_3$).

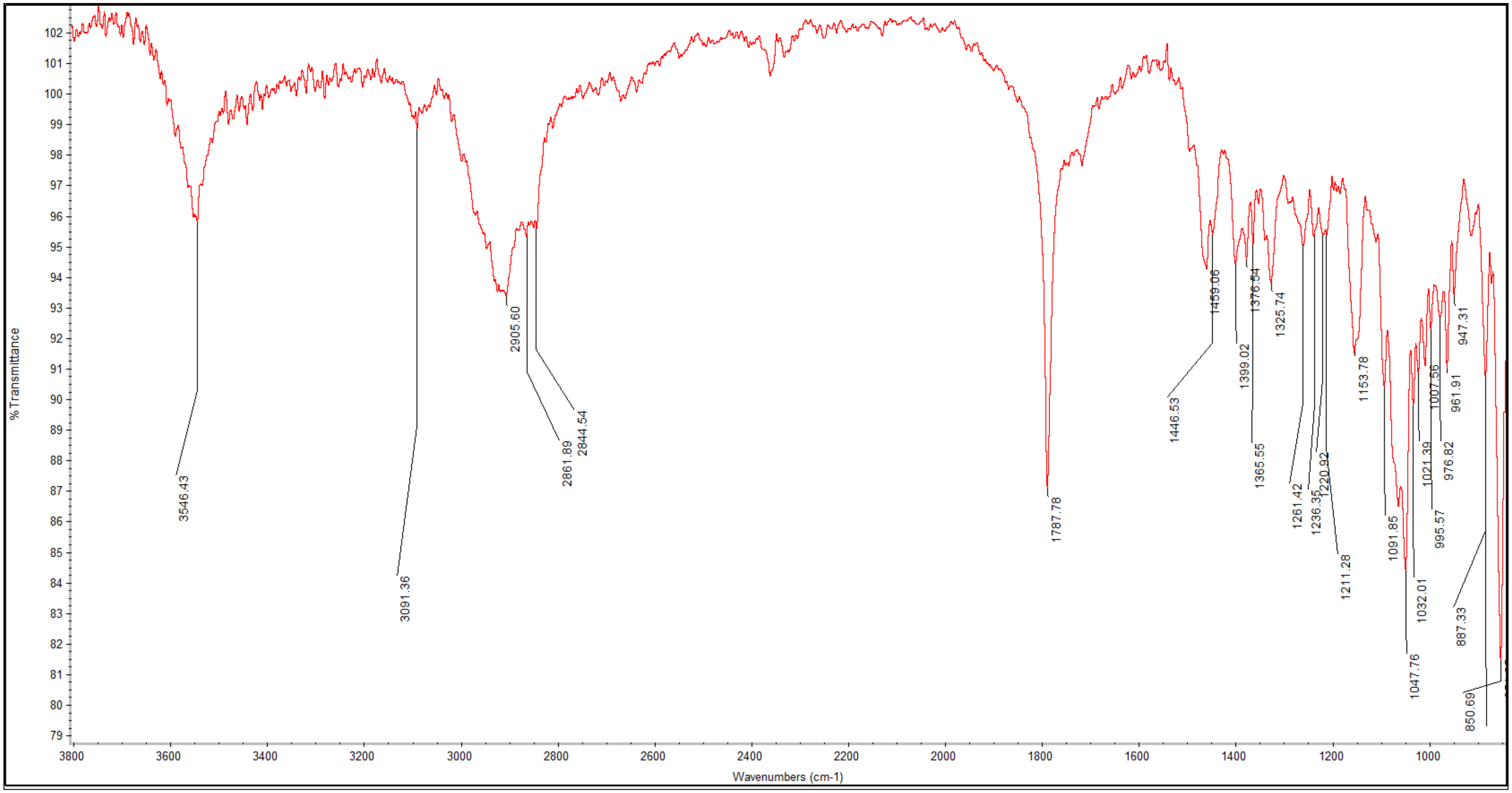

Figure S23. IR spectrum of (−)-(5*S*,6*R*,8*S*,9*S*,10*R*,13*R*,14*R*,15*S*,16*R*)-15,16-epoxy-6-hydroxy-13-spirocassan-12,16-olide (isospirocaesalmin E) (**2b**).

Elemental Composition Report

Single Mass Analysis
Tolerance = 15.0 PPM / DBE: min = -1.5, max = 150.0
Element prediction: Off
Number of isotope peaks used for i-FIT = 3

Monoisotopic Mass, Even Electron Ions
642 formula(e) evaluated with 5 results within limits (all results (up to 1000) for each mass)
Elements Used:
C: 0-100 H: 0-100 N: 0-10 O: 0-10 I: 0-1

Armando TALAVERA 10-May-2023
Injdir - dilution 200
AT-2023-4-(01) 13 (0.112) Cm (12:14) 1: TOF MS ES+ 4.63e+003

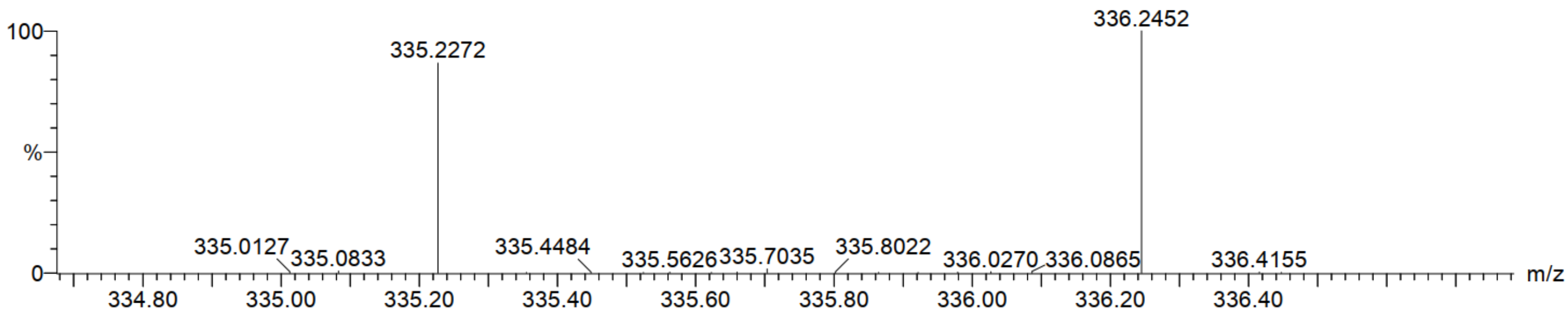

Mass	Calc. Mass	mDa	PPM	DBE	i-FIT	i-FIT (Norm)	Formula
Minimum:				-1.5			
Maximum:		5.0	15.0	150.0			
335.2272	335.2268	0.4	1.2	2.5	109.0	1.4	C10 H27 N10 O3
	335.2308	-3.6	-10.7	6.5	109.0	1.4	C15 H27 N8 O
	335.2294	-2.2	-6.6	1.5	109.2	1.6	C14 H31 N4 O5
	335.2236	3.6	10.7	10.5	109.4	1.8	C21 H27 N4
	335.2222	5.0	14.9	5.5	109.5	1.9	C20 H31 O4

Figure S24. HRMS spectrum of (−)-(5*S*,6*R*,8*S*,9*S*,10*R*,13*R*,14*R*,15*S*,16*R*)-15,16-epoxy-6-hydroxy-13-spirocassan-12,16-olide (isospirocaesalmin E) (**2b**).

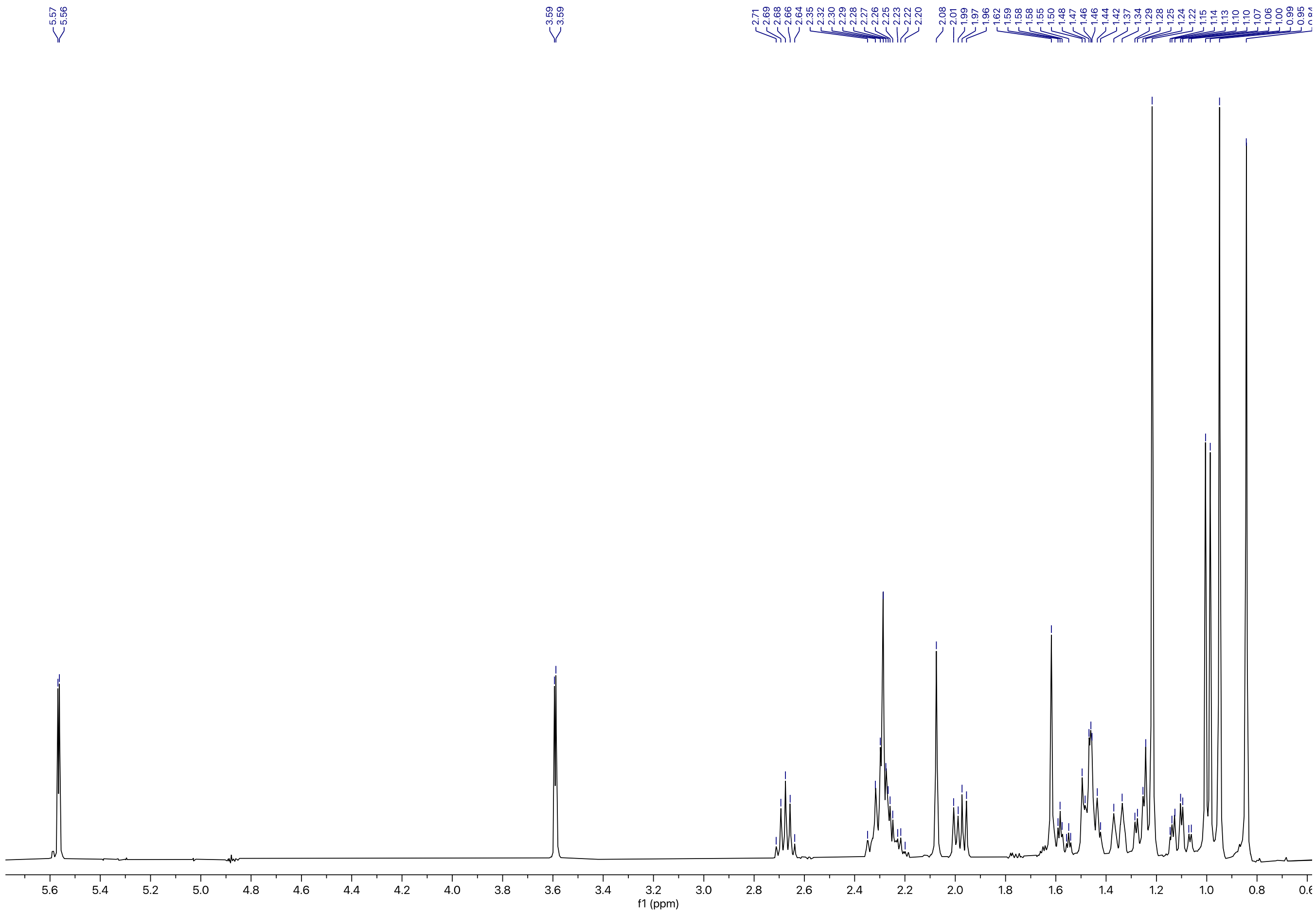

Figure S25. ^{1}H NMR spectrum of (+)-(5*S*,8*S*,9*S*,10*R*,13*S*,14*R*,15*R*,16*S*)-15,16-epoxy-6-oxo-13-spirocassan-12,16-olide (spirocaesalmin F) (**3a**), (300 MHz, $CDCl_3$).

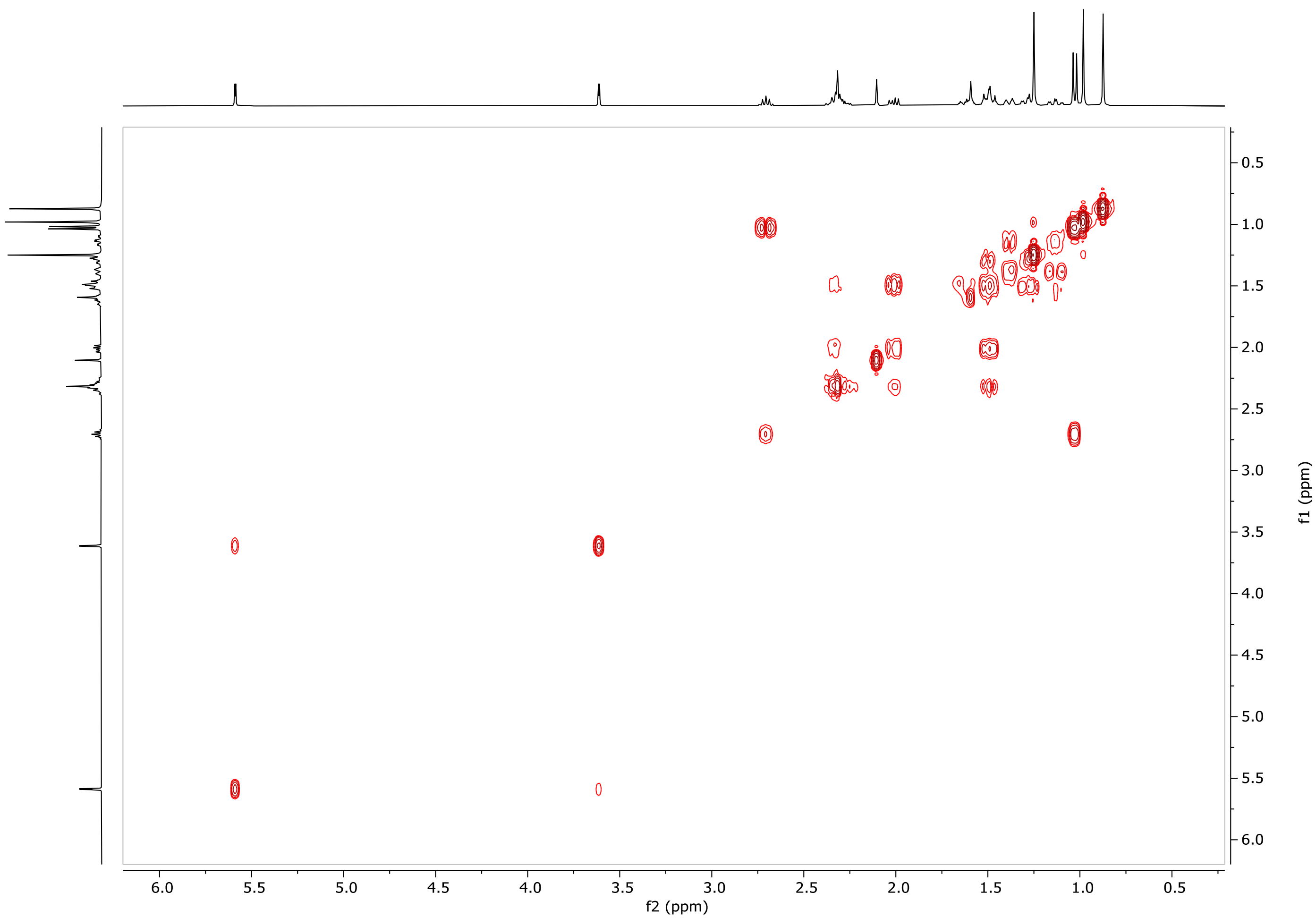

Figure S26. COSY spectrum of (+)-(5*S*,8*S*,9*S*,10*R*,13*S*,14*R*,15*R*,16*S*)-15,16-epoxy-6-oxo-13-spirocassan-12,16-olide (spirocaesalmin F) (**3a**), (300 MHz, $CDCl_3$).

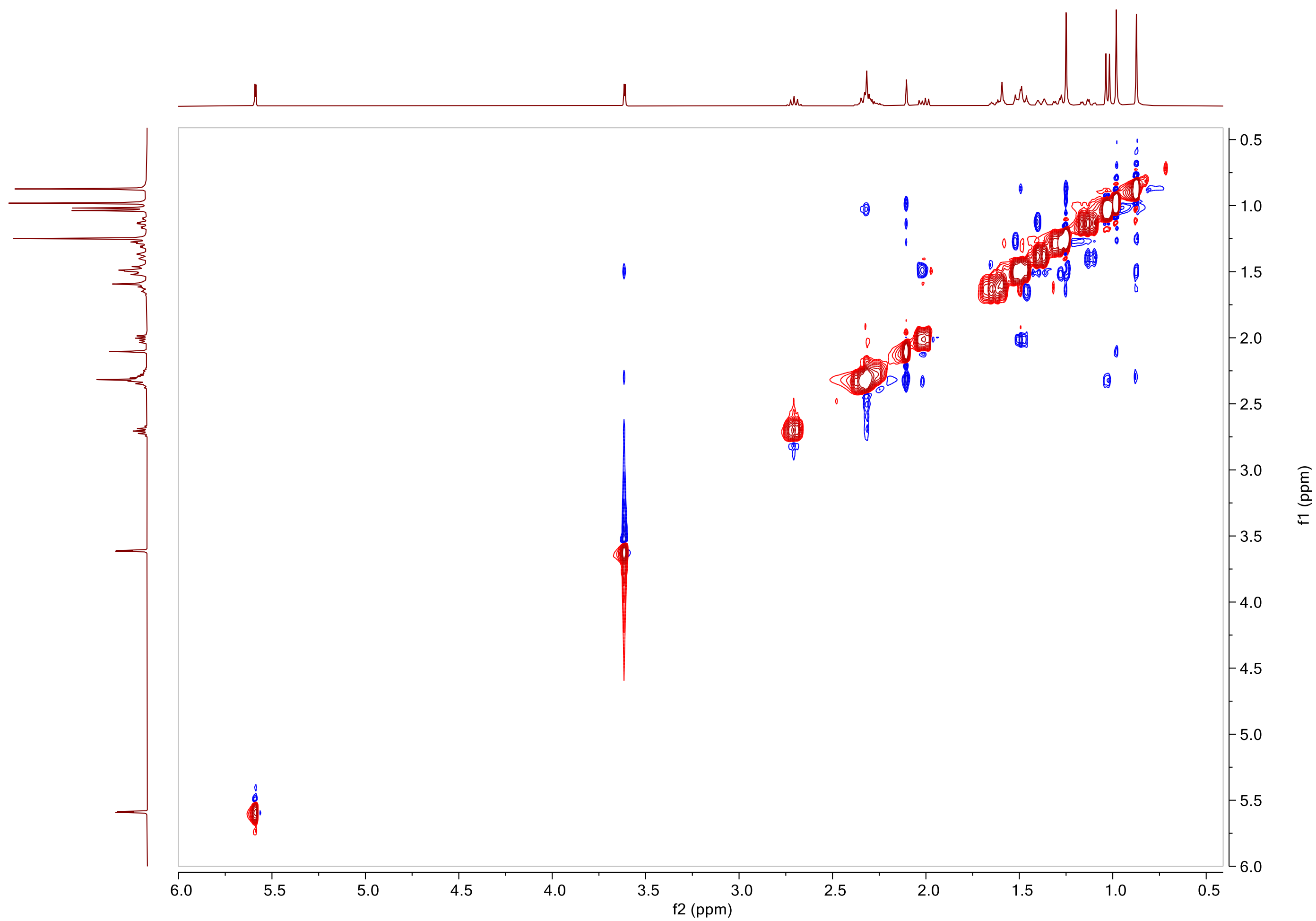

Figure S27. NOESY spectrum of (+)-(5*S*,8*S*,9*S*,10*R*,13*S*,14*R*,15*R*,16*S*)-15,16-epoxy-6-oxo-13-spirocassan-12,16-olide (spirocaesalmin F) (**3a**), (300 MHz, $CDCl_3$).

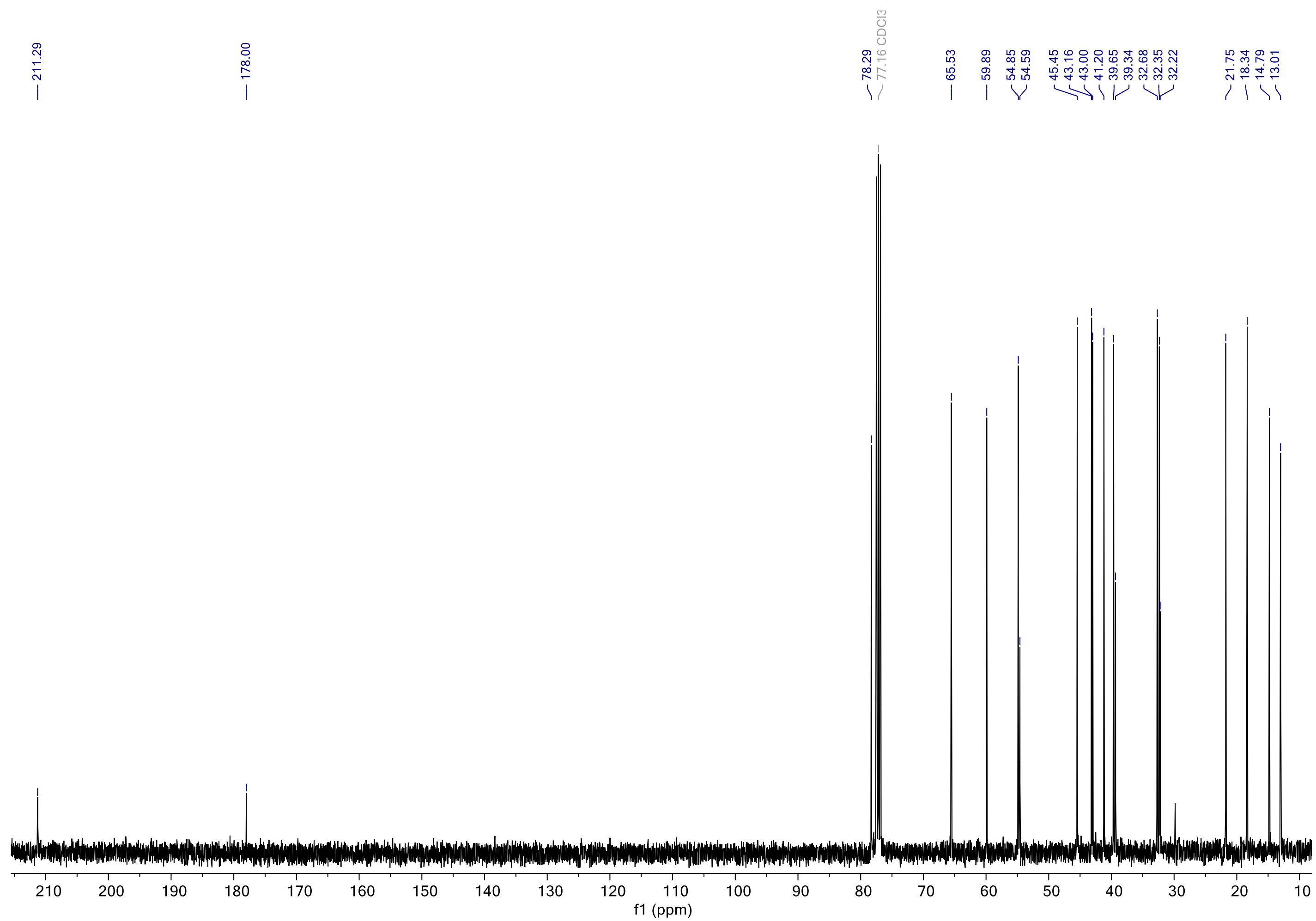

Figure S28. ^{13}C NMR spectrum of (+)-(5*S*,8*S*,9*S*,10*R*,13*S*,14*R*,15*R*,16*S*)-15,16-epoxy-6-oxo-13-spirocassan-12,16-olide (spirocaesalmin F) (**3a**), (75 MHz, $CDCl_3$).

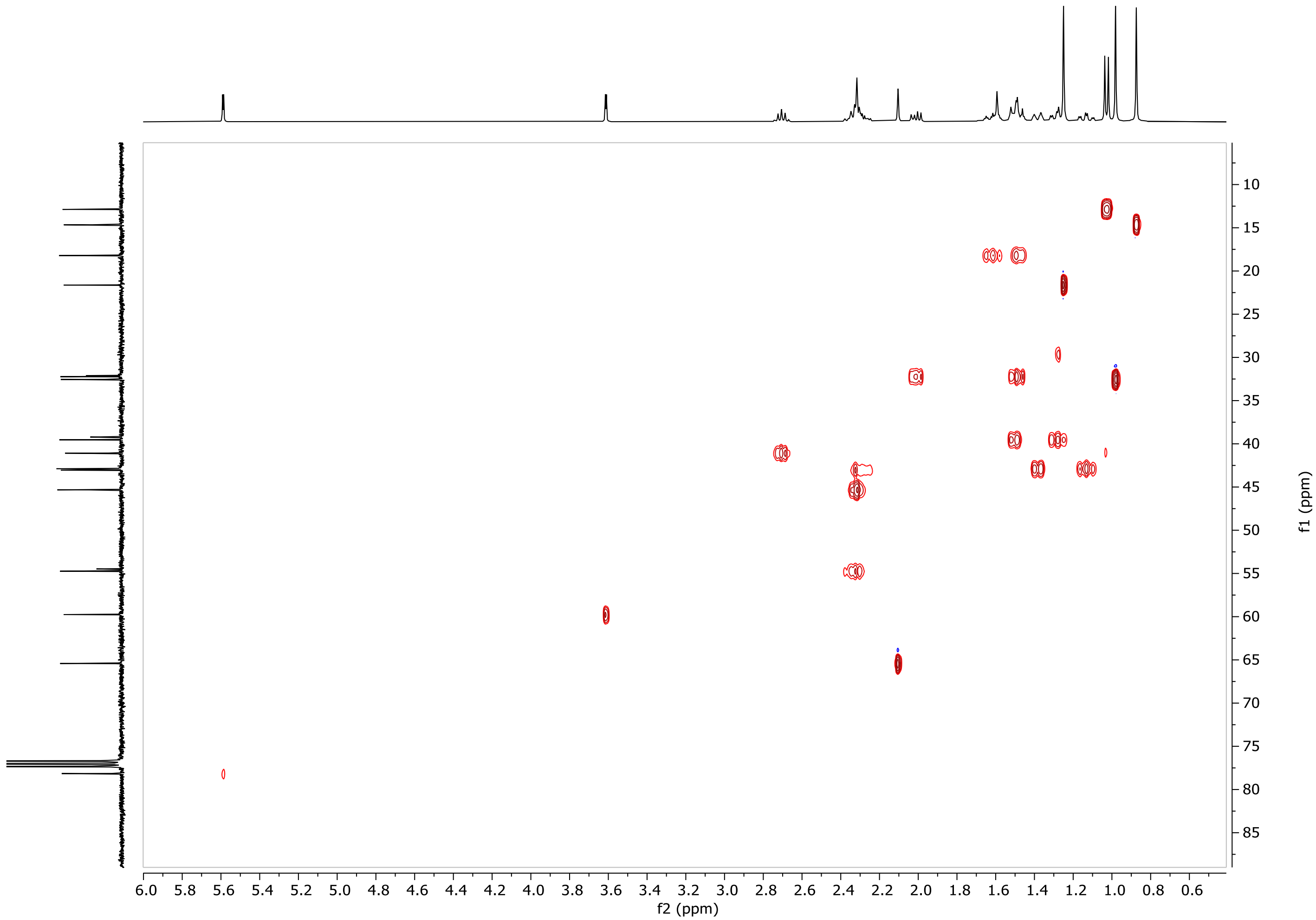

Figure S29. HSQC spectrum of (+)-(5*S*,8*S*,9*S*,10*R*,13*S*,14*R*,15*R*,16*S*)-15,16-epoxy-6-oxo-13-spirocassan-12,16-olide (spirocaesalmin F) (**3a**), (300/75 MHz, $CDCl_3$).

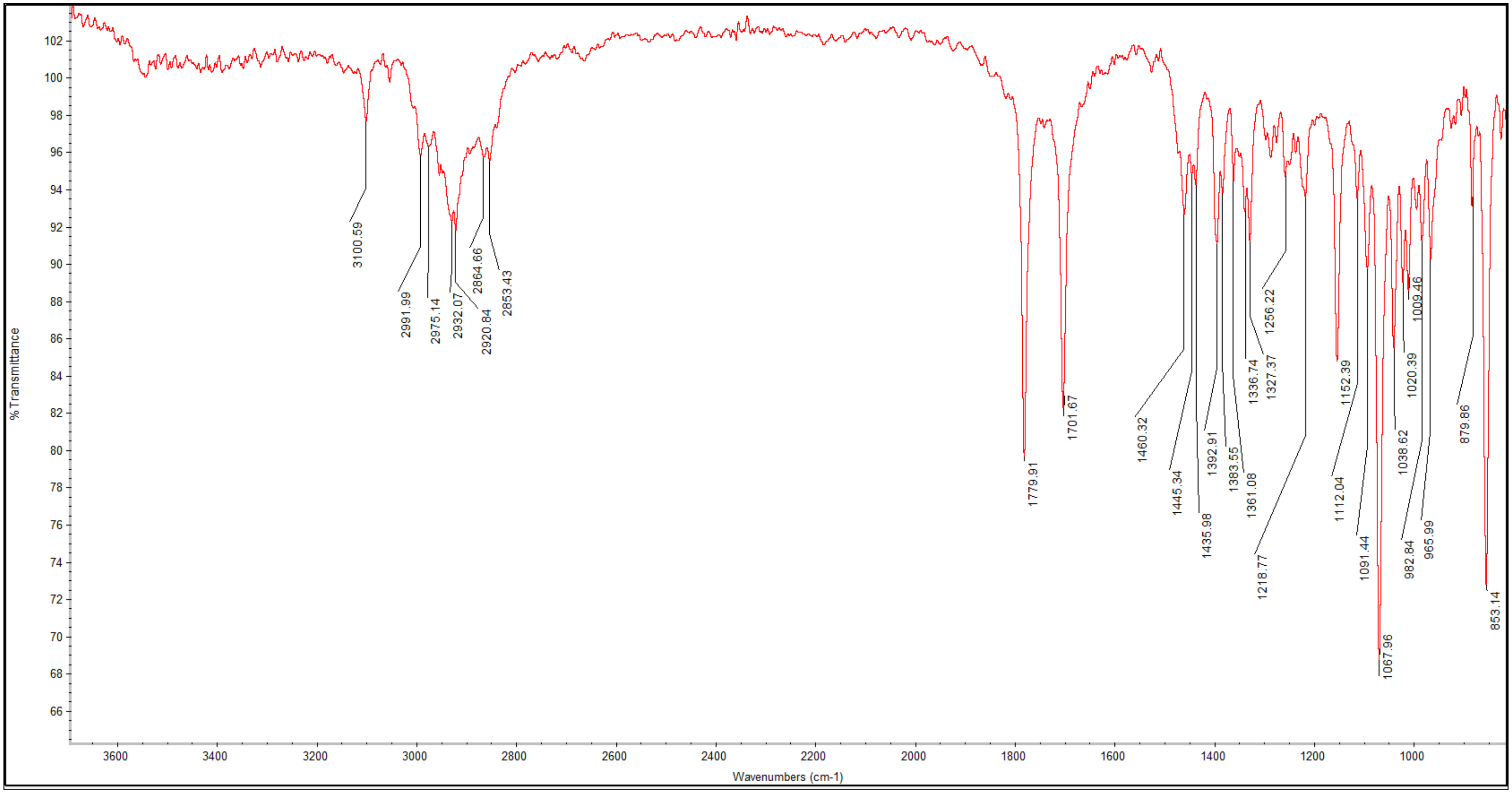

Figure S30. IR spectrum of (+)-(5*S*,8*S*,9*S*,10*R*,13*S*,14*R*,15*R*,16*S*)-15,16-epoxy-6-oxo-13-spirocassan-12,16-olide (spirocaesalmin F) (**3a**).

Elemental Composition Report

Single Mass Analysis

Tolerance = 10.0 PPM / DBE: min = -1.5, max = 150.0
Element prediction: Off
Number of isotope peaks used for i-FIT = 3

Monoisotopic Mass, Even Electron Ions
630 formula(e) evaluated with 3 results within limits (all results (up to 1000) for each mass)
Elements Used:
C: 0-100 H: 0-100 N: 0-10 O: 0-10 I: 0-1

Armando TALAVERA
Injdir - dilution 200
AT-2023-5-(01) 10 (0.091) Cm (9:11-1:5)

10-May-2023
1: TOF MS ES+
1.45e+004

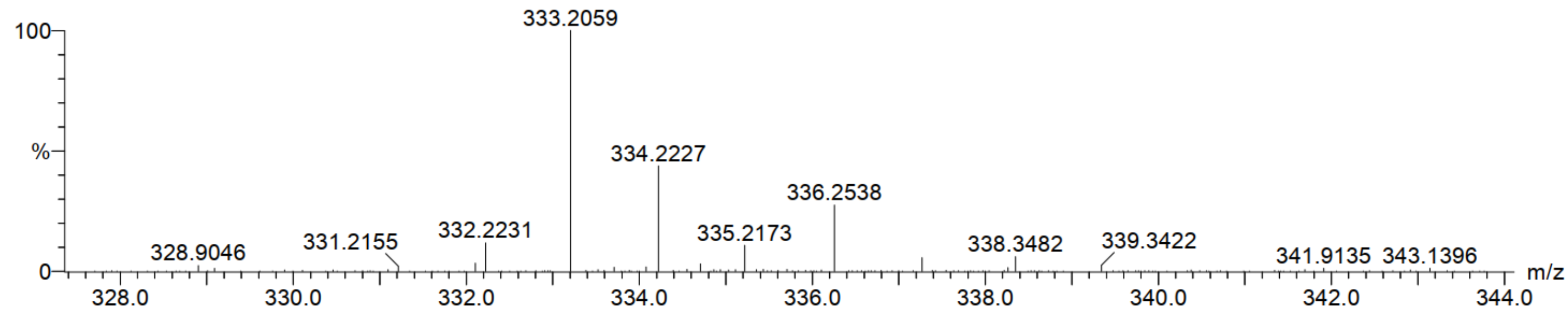

Minimum:				-1.5			
Maximum:		5.0	10.0	150.0			
Mass	Calc. Mass	mDa	PPM	DBE	i-FIT	i-FIT (Norm)	Formula
333.2059	333.2066	-0.7	-2.1	6.5	212.3	1.0	C20 H29 O4
	333.2079	-2.0	-6.0	11.5	212.1	0.8	C21 H25 N4
	333.2039	2.0	6.0	7.5	213.1	1.8	C16 H25 N6 O2

Figure S31. HRMS spectrum of (+)-(5*S*,8*S*,9*S*,10*R*,13*S*,14*R*,15*R*,16*S*)-15,16-epoxy-6-oxo-13-spirocassan-12,16-olide (spirocaesalmin F) (**3a**).

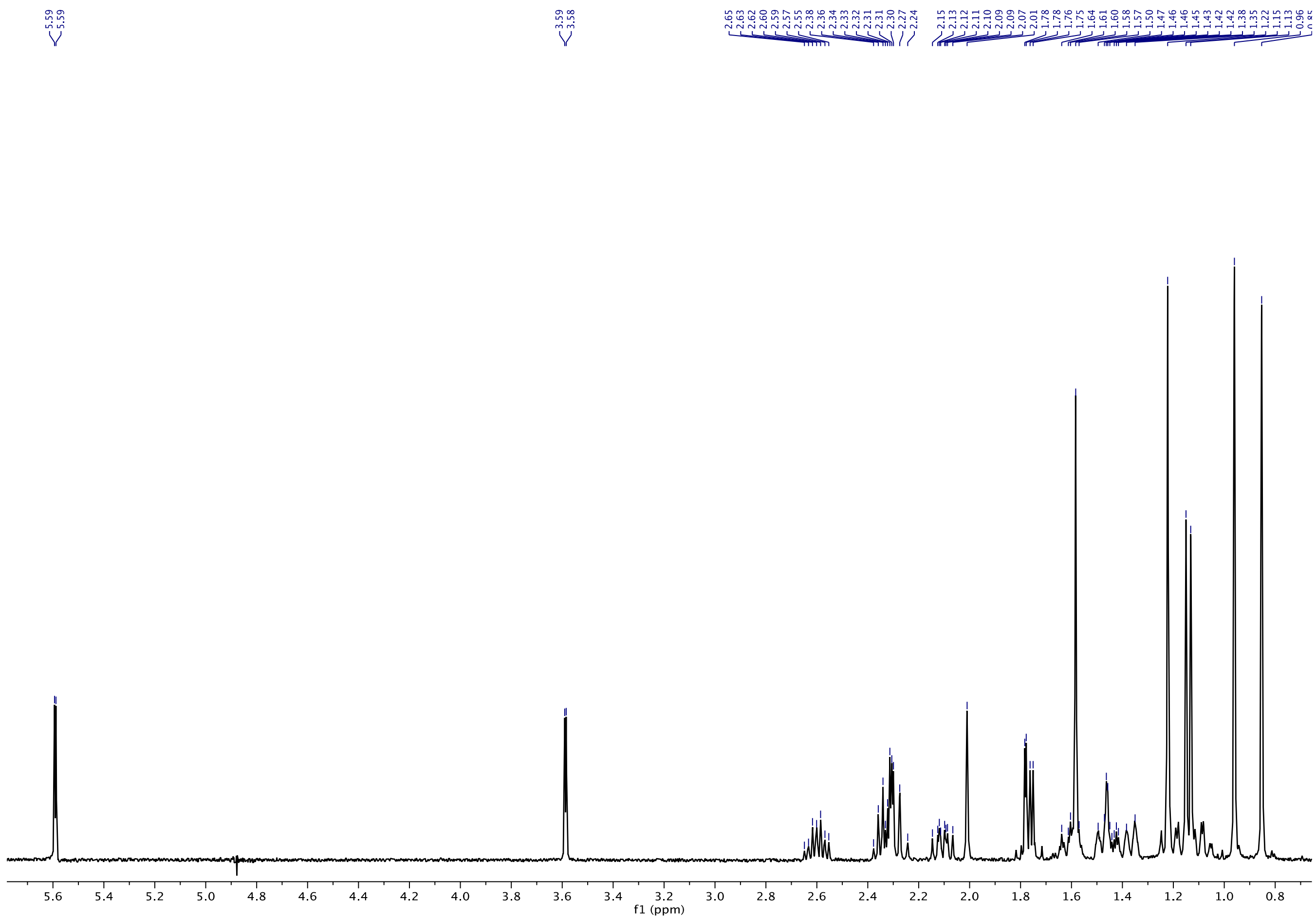

Figure S32. ^{1}H NMR spectrum of (−)-(5*S*,8*S*,9*S*,10*R*,13*R*,14*R*,15*S*,16*R*)-15,16-epoxy-6-oxo-13-spirocassan-12,16-olide (isospirocaesalmin F) (**3b**), (300 MHz, $CDCl_3$).

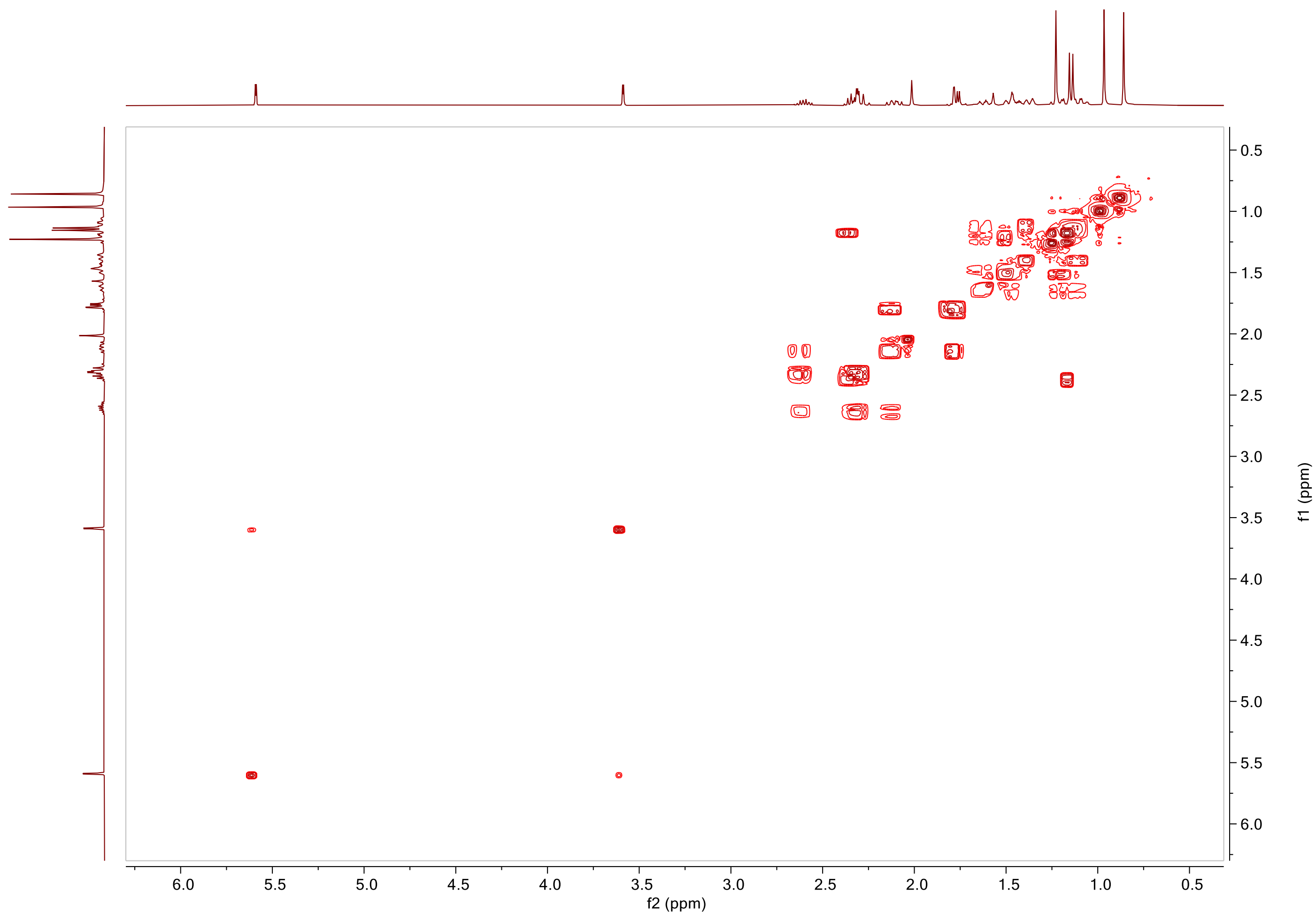

Figure S33. COSY spectrum of (−)-(5*S*,8*S*,9*S*,10*R*,13*R*,14*R*,15*S*,16*R*)-15,16-epoxy-6-oxo-13-spirocassan-12,16-olide (isospirocaesalmin F) (**3b**), (300 MHz, $CDCl_3$).

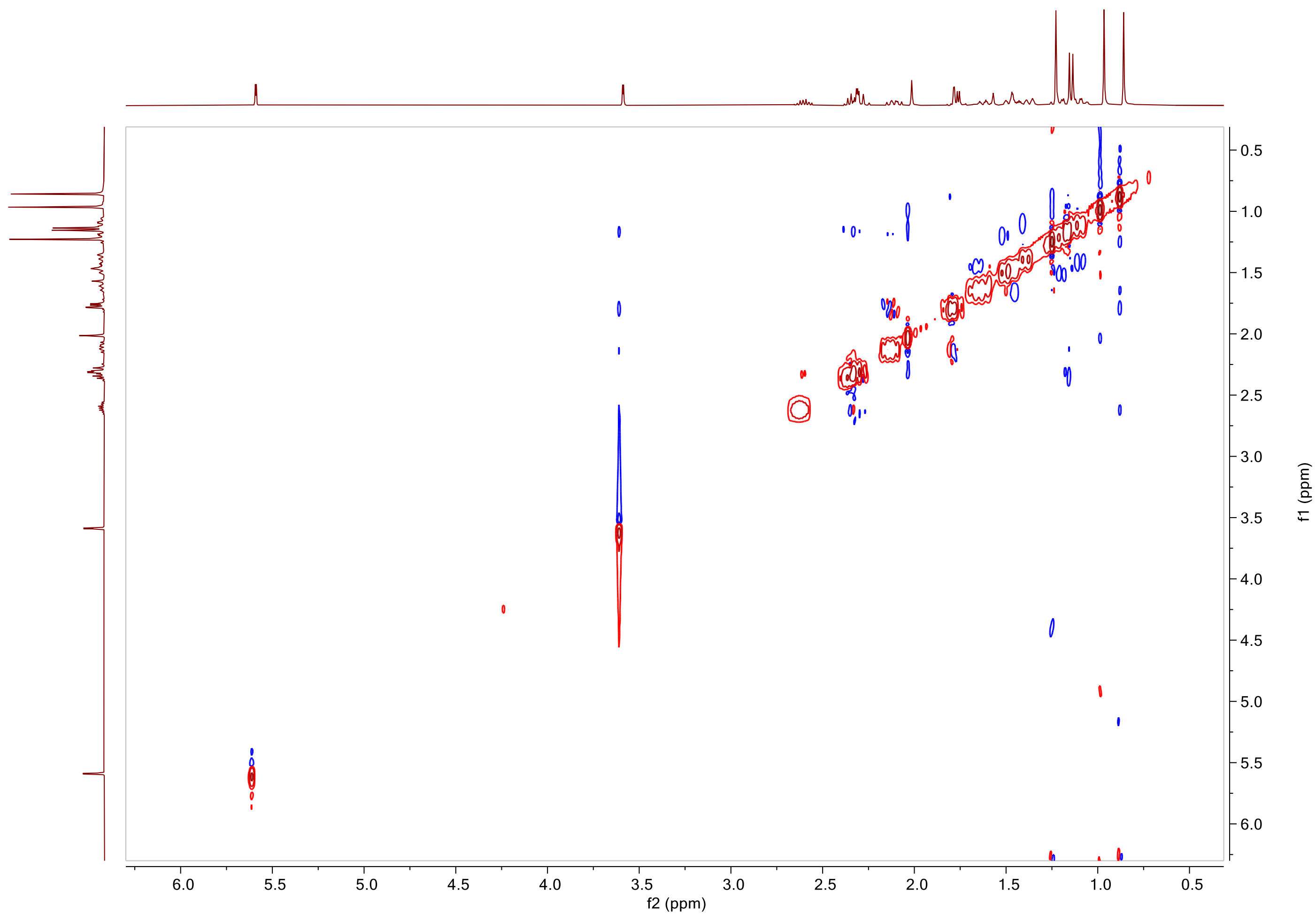

Figure S34. NOESY spectrum of (−)-(5*S*,8*S*,9*S*,10*R*,13*R*,14*R*,15*S*,16*R*)-15,16-epoxy-6-oxo-13-spirocassan-12,16-olide (isospirocaesalmin F) (**3b**), (300 MHz, $CDCl_3$).

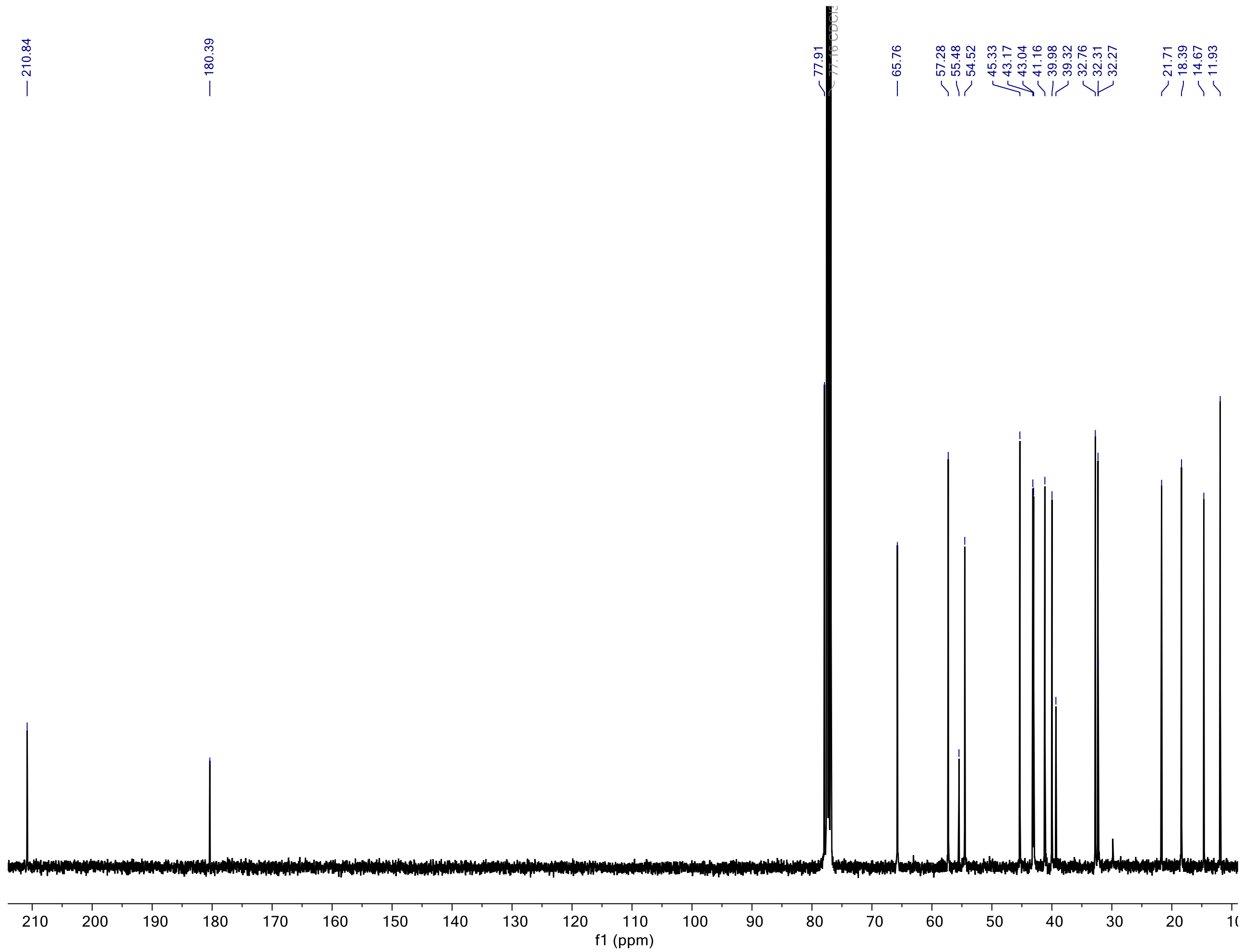

Figure S35. ^{13}C NMR spectrum of (−)-(5*S*,8*S*,9*S*,10*R*,13*R*,14*R*,15*S*,16*R*)-15,16-epoxy-6-oxo-13-spirocassan-12,16-olide (isospirocaesalmin F) (**3b**), (75 MHz, $CDCl_3$).

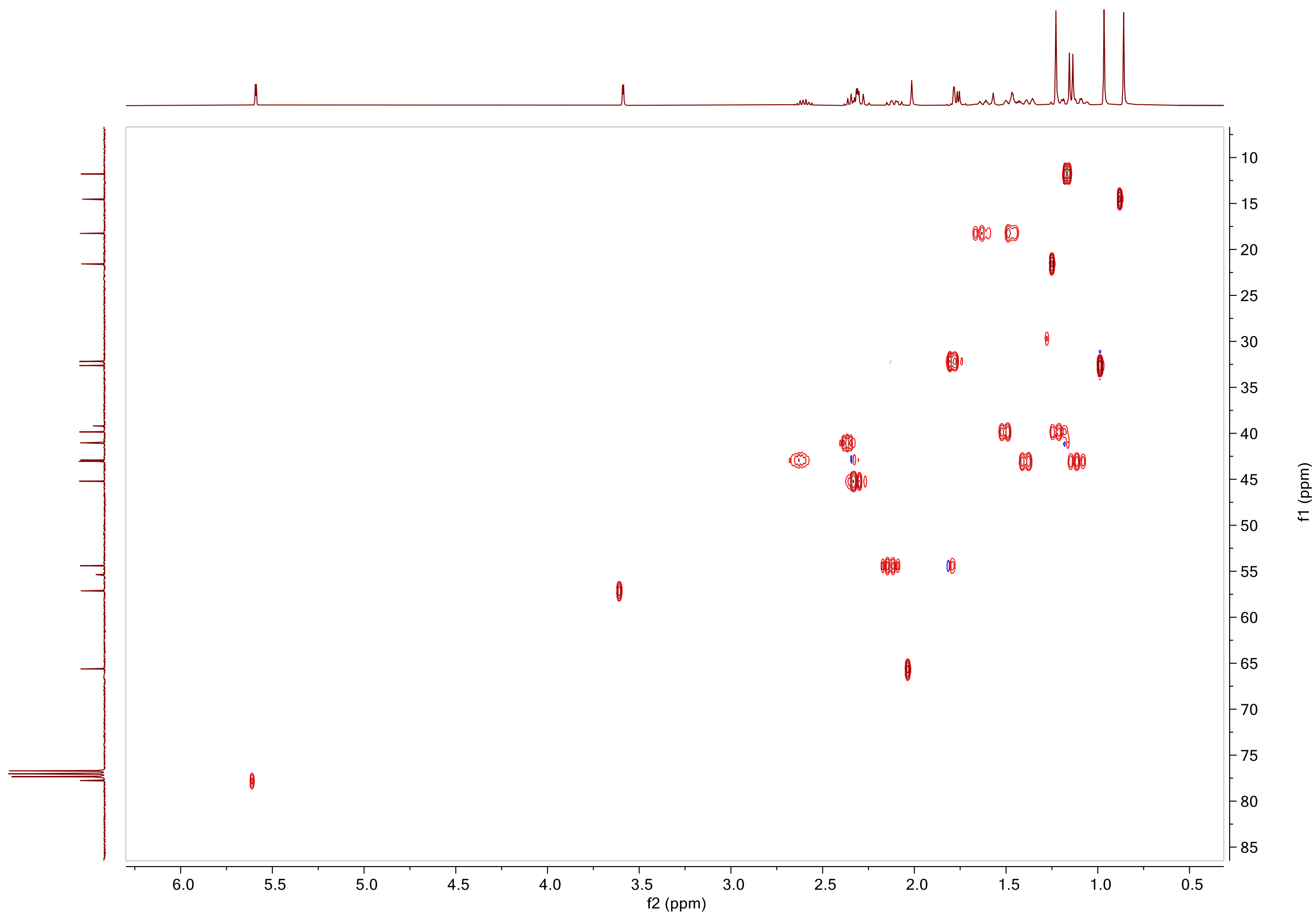

Figure S36. HSQC spectrum of (−)-(5*S*,8*S*,9*S*,10*R*,13*R*,14*R*,15*S*,16*R*)-15,16-epoxy-6-oxo-13-spirocassan-12,16-olide (isospirocaesalmin F) (**3b**), (300/75 MHz, $CDCl_3$).

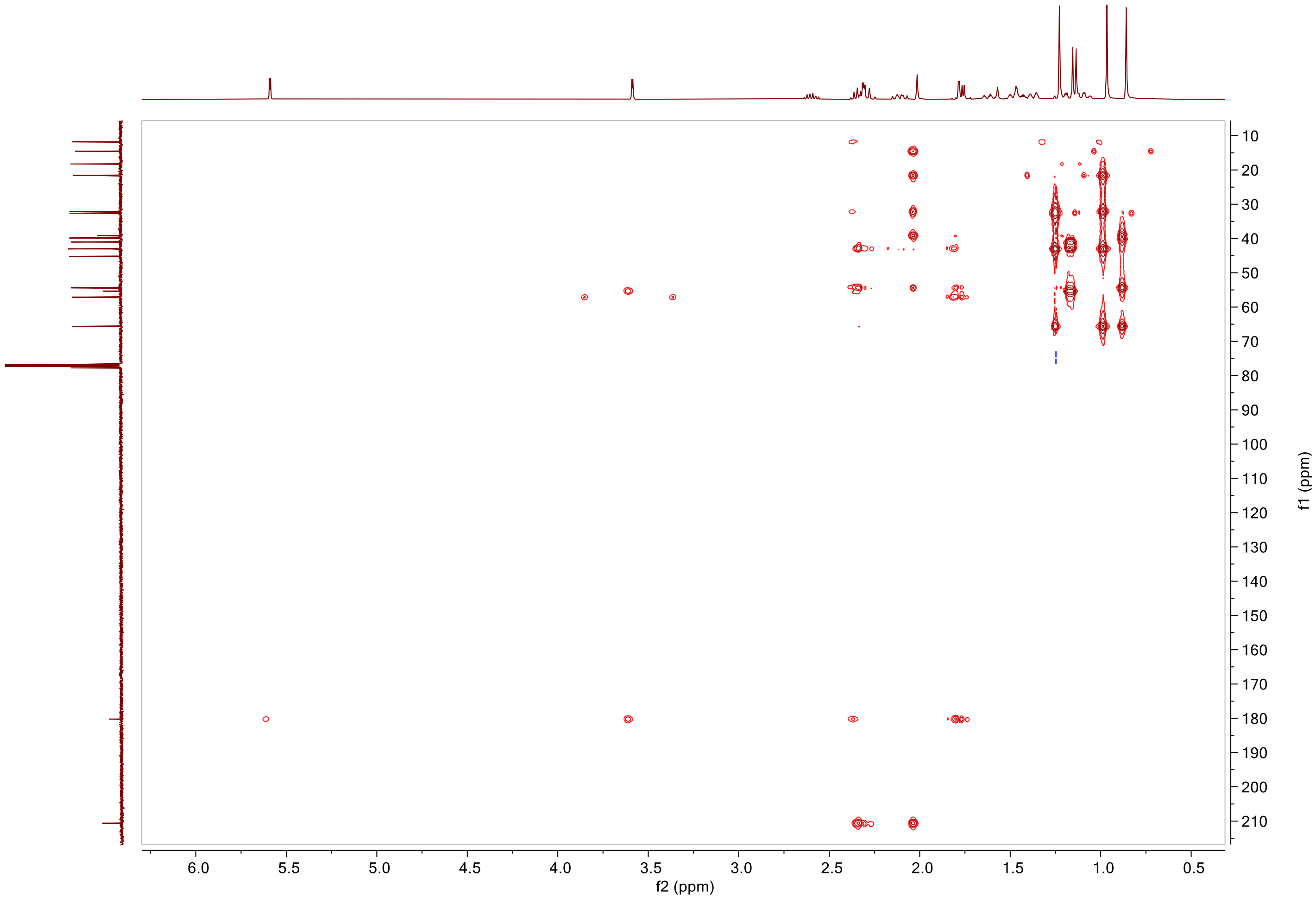

Figure S37. HMBC spectrum of (−)-(5*S*,8*S*,9*S*,10*R*,13*R*,14*R*,15*S*,16*R*)-15,16-epoxy-6-oxo-13-spirocassan-12,16-olide (isospirocaesalmin F) (**3b**), (300/75 MHz, $CDCl_3$).

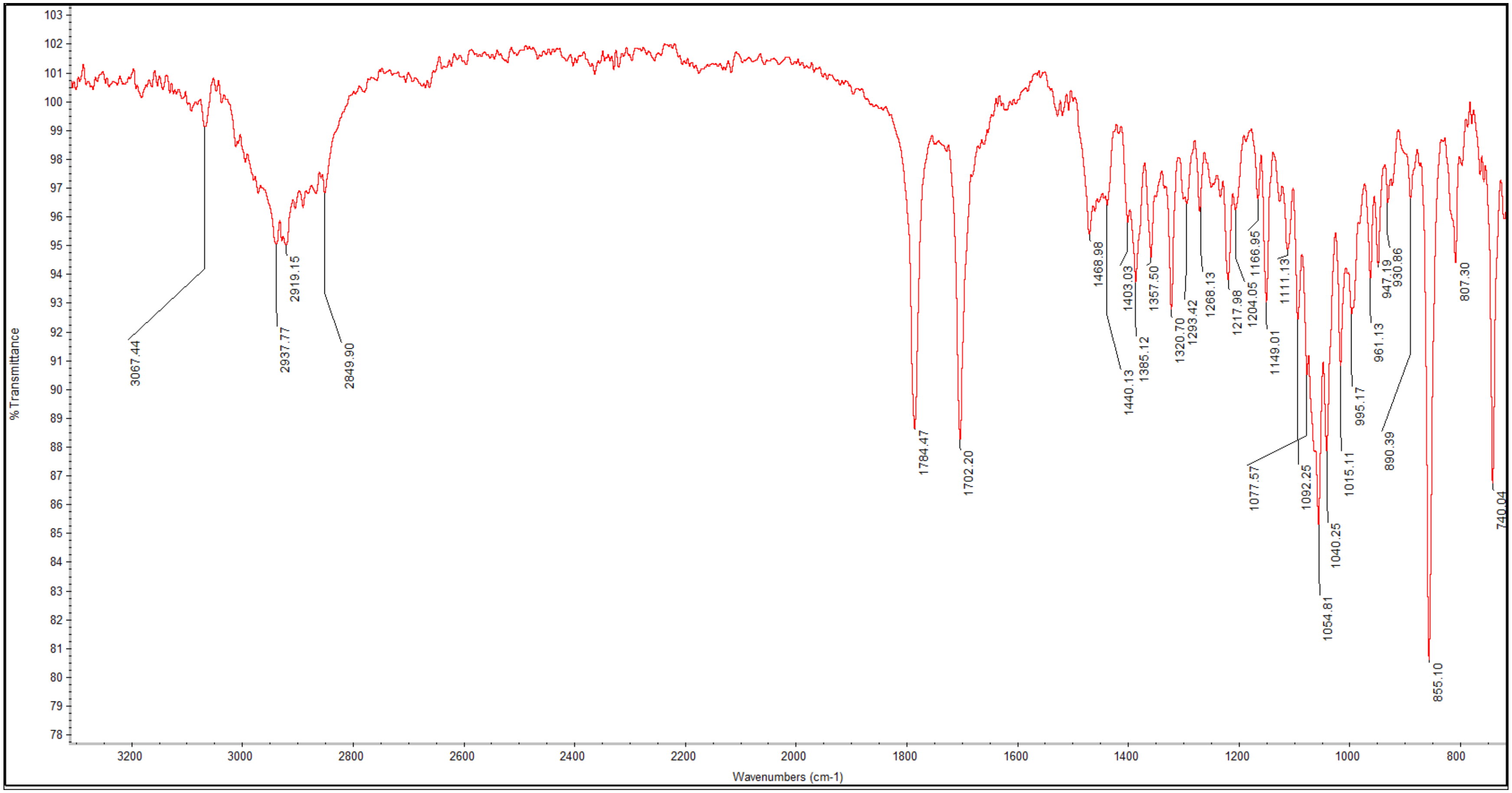

Figure S38. IR spectrum of (−)-(5*S*,8*S*,9*S*,10*R*,13*R*,14*R*,15*S*,16*R*)-15,16-epoxy-6-oxo-13-spirocassan-12,16-olide (isospirocaesalmin F) (**3b**).

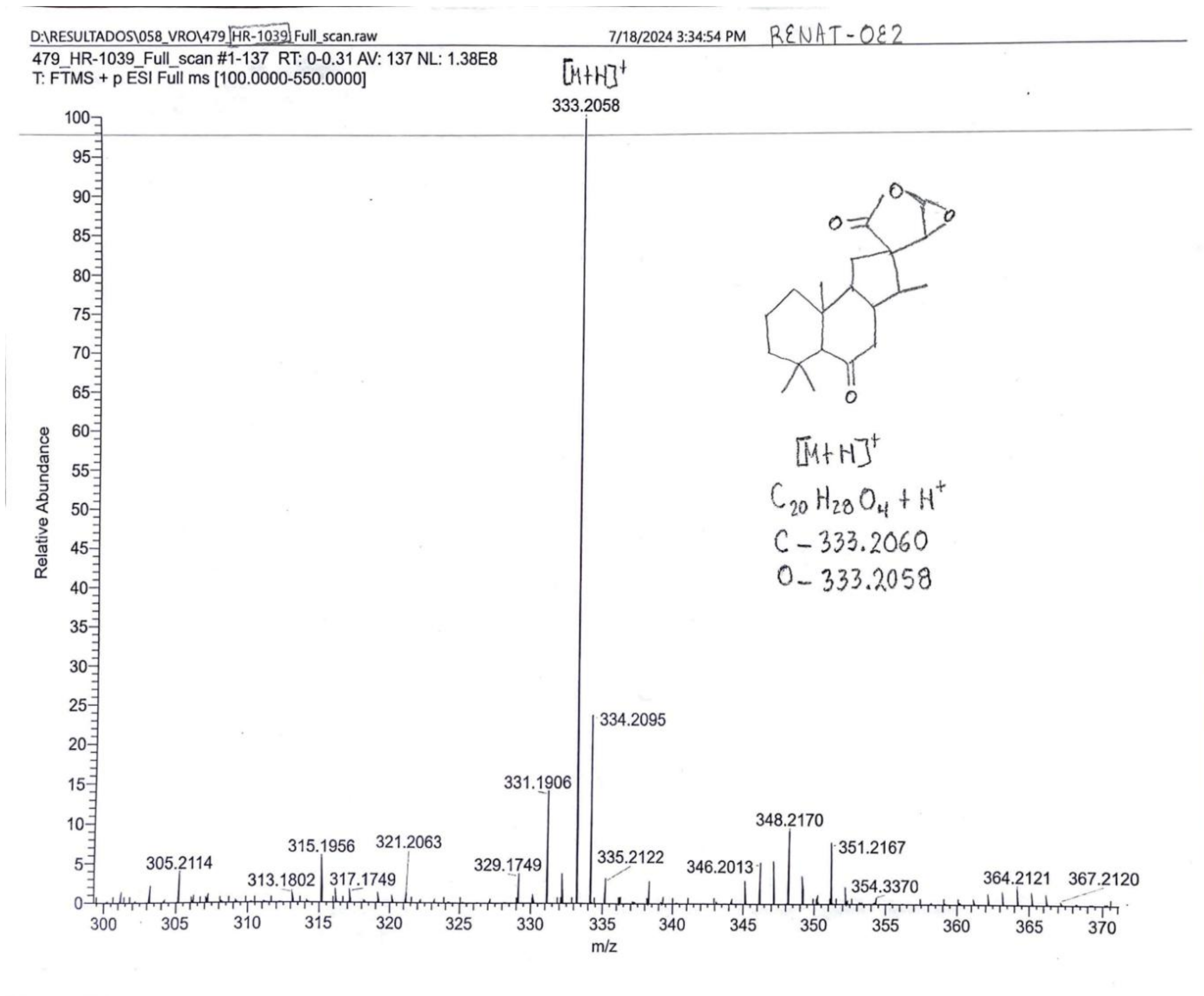

Figure S39. HRMS spectrum of (−)-(5*S*,8*S*,9*S*,10*R*,13*R*,14*R*,15*S*,16*R*)-15,16-epoxy-6-oxo-13-spirocassan-12,16-olide (isospirocaesalmin F) (**3b**).

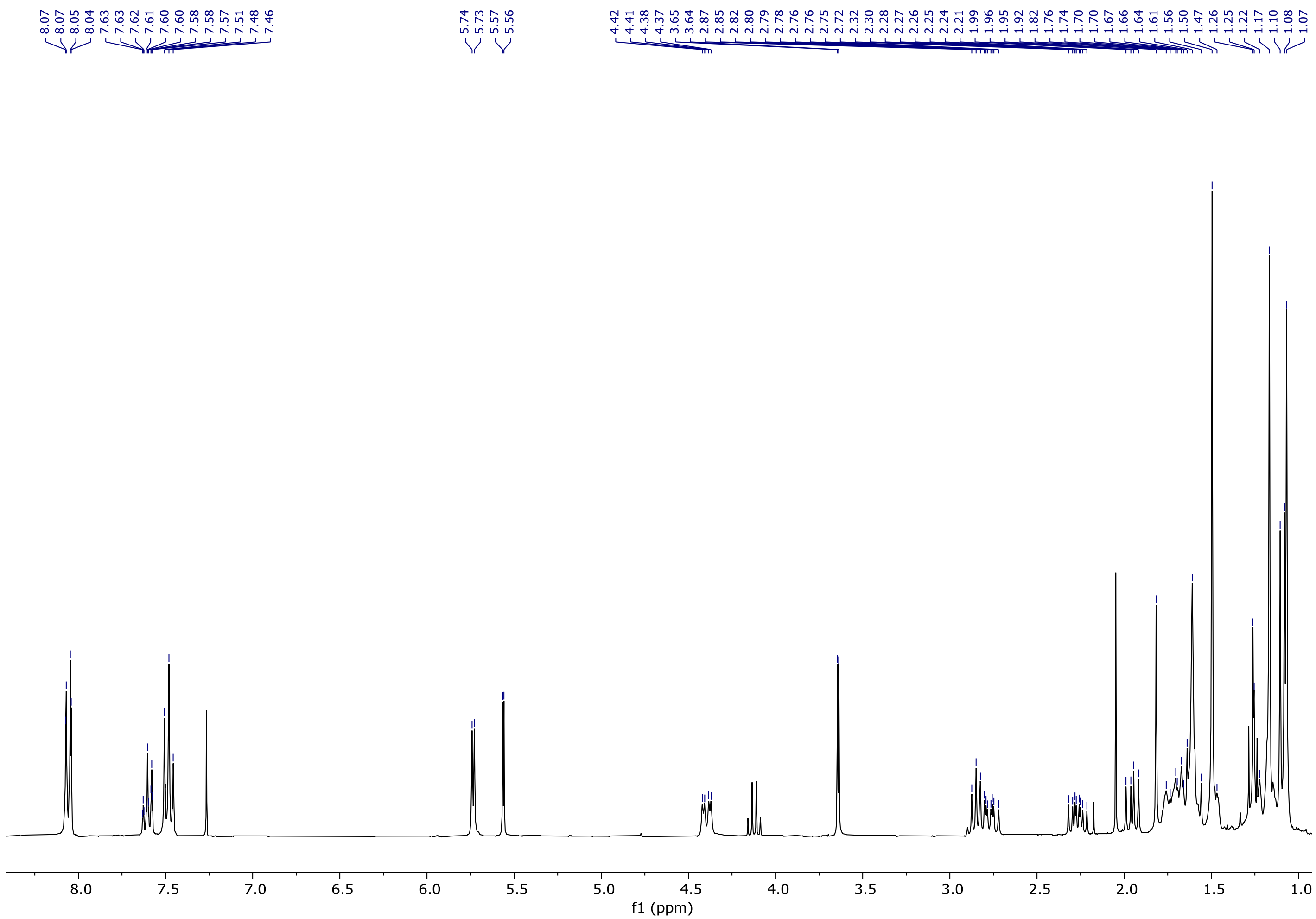

Figure S40. ^{1}H NMR spectrum of (+)-(5*R*,6*R*,7*R*,8*S*,9*S*,10*R*,13*S*,14*R*,15*R*,16*S*)-6-benzoyloxy-15,16-epoxy-5,7-dihydroxy-13-spirocassan-12,16-olide (spirocaesalmin G) (**4a**), (400 MHz, $CDCl_3$).

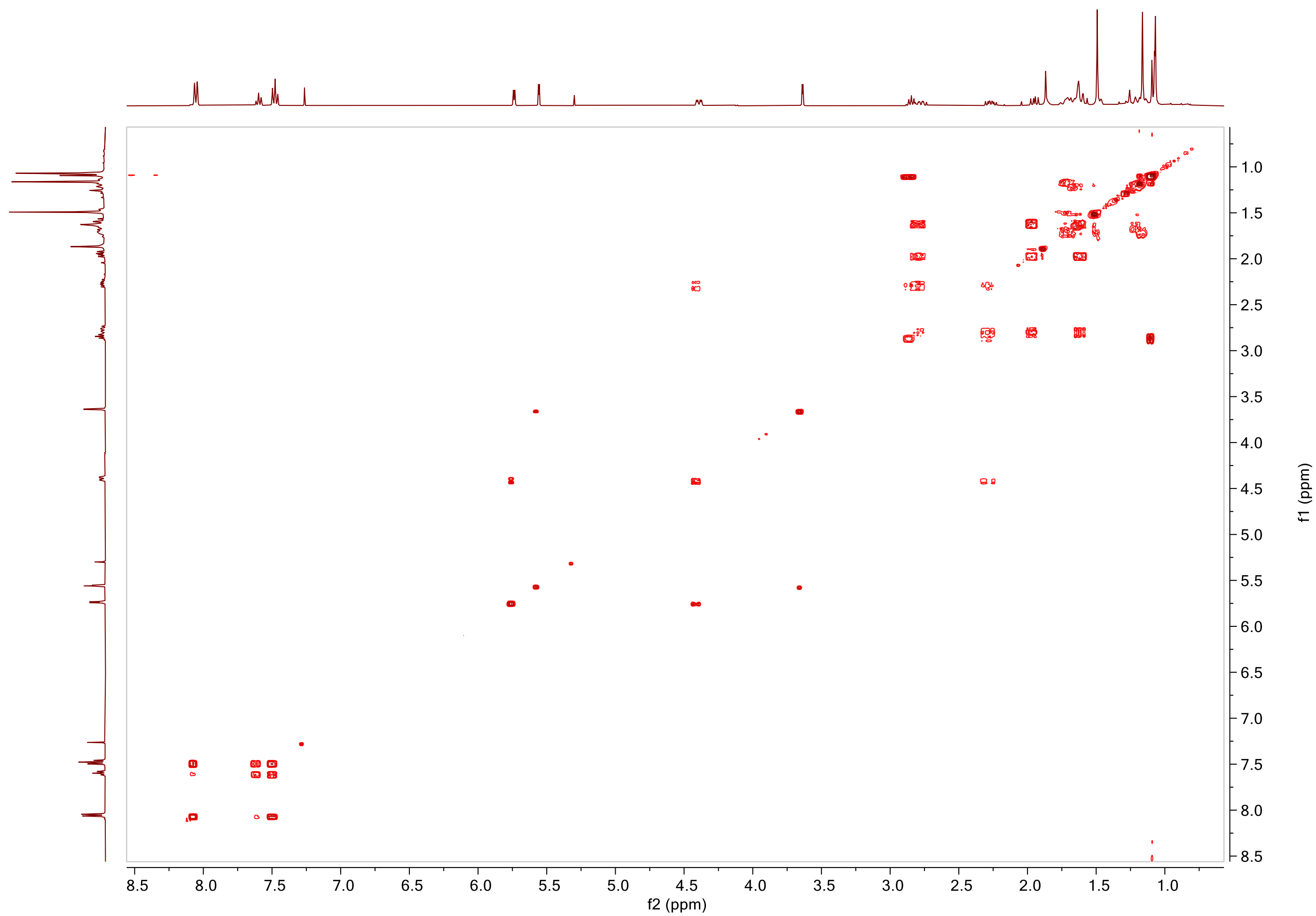

Figure S41. COSY spectrum of (+)-(5*R*,6*R*,7*R*,8*S*,9*S*,10*R*,13*S*,14*R*,15*R*,16*S*)-6-benzoyloxy-15,16-epoxy-5,7-dihydroxy-13-spirocassan-12,16-olide (spirocaesalmin G) (**4a**), (400 MHz, $CDCl_3$).

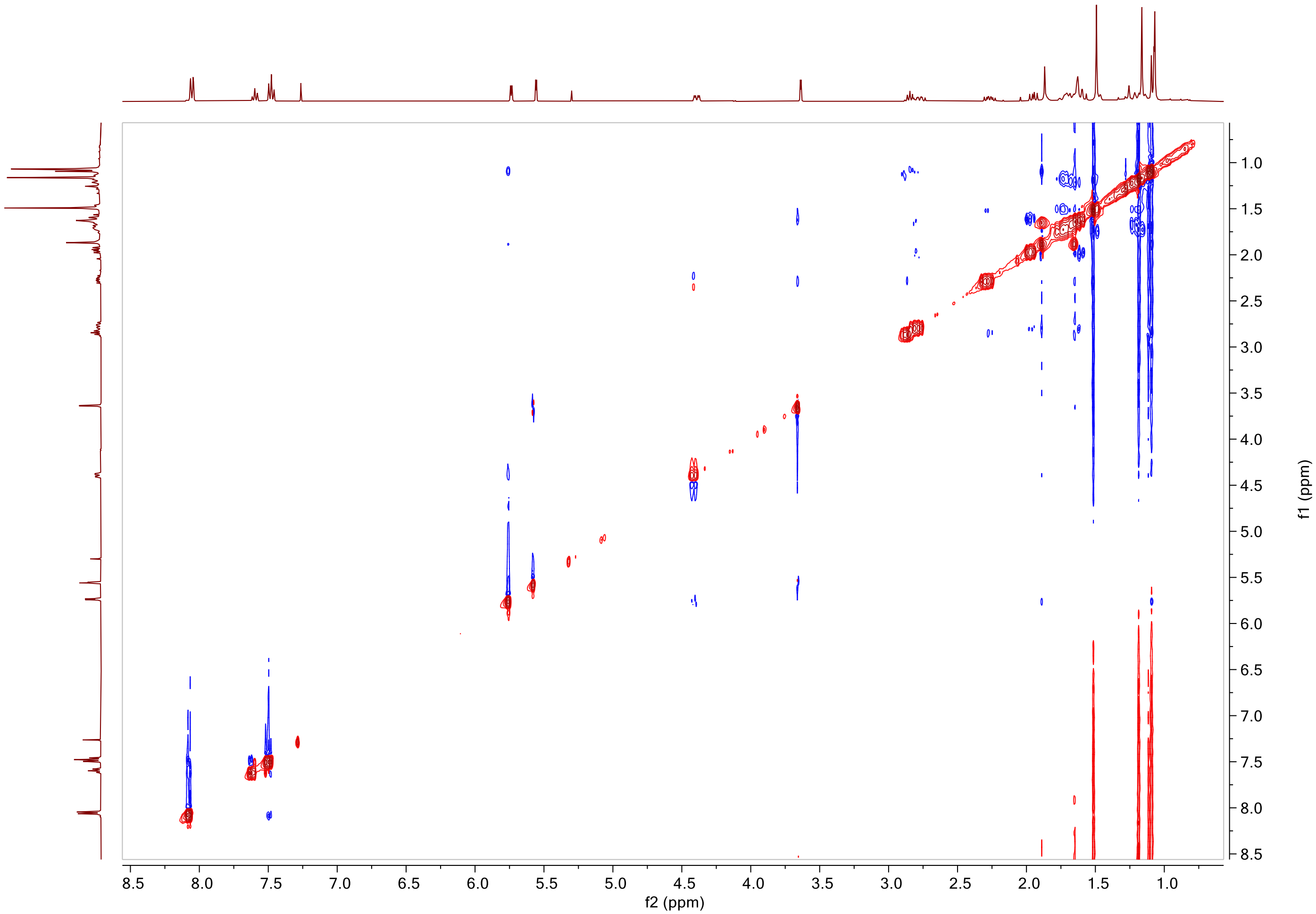

Figure S42. NOESY spectrum of (+)-(5*R*,6*R*,7*R*,8*S*,9*S*,10*R*,13*S*,14*R*,15*R*,16*S*)-6-benzoyloxy-15,16-epoxy-5,7-dihydroxy-13-spirocassan-12,16-olide (spirocaesalmin G) (**4a**), (400 MHz, $CDCl_3$).

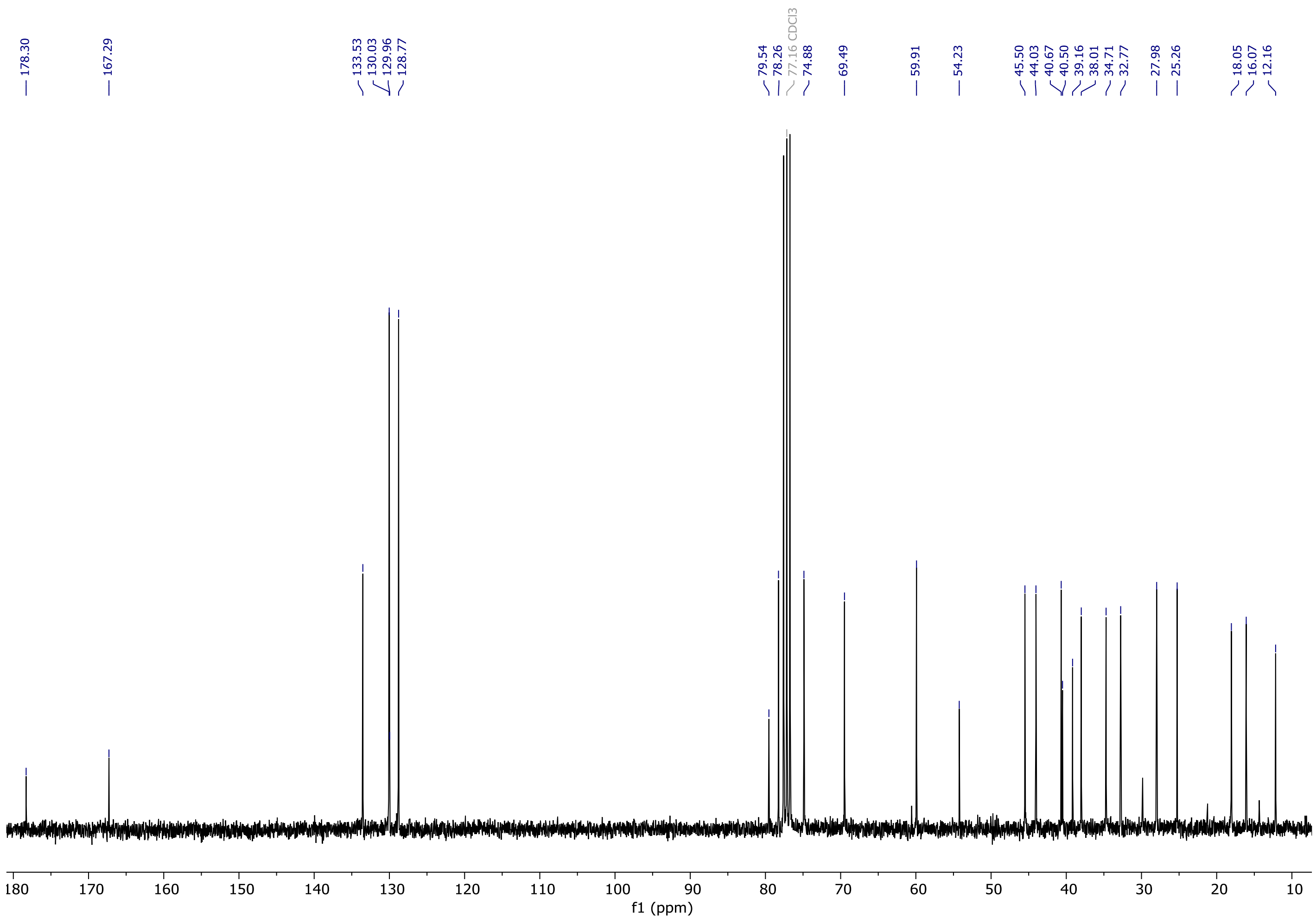

Figure S43. ^{13}C NMR spectrum of (+)-(5*R*,6*R*,7*R*,8*S*,9*S*,10*R*,13*S*,14*R*,15*R*,16*S*)-6-benzoyloxy-15,16-epoxy-5,7-dihydroxy-13-spirocassan-12,16-olide (spirocaesalmin G) (**4a**), (100 MHz, $CDCl_3$).

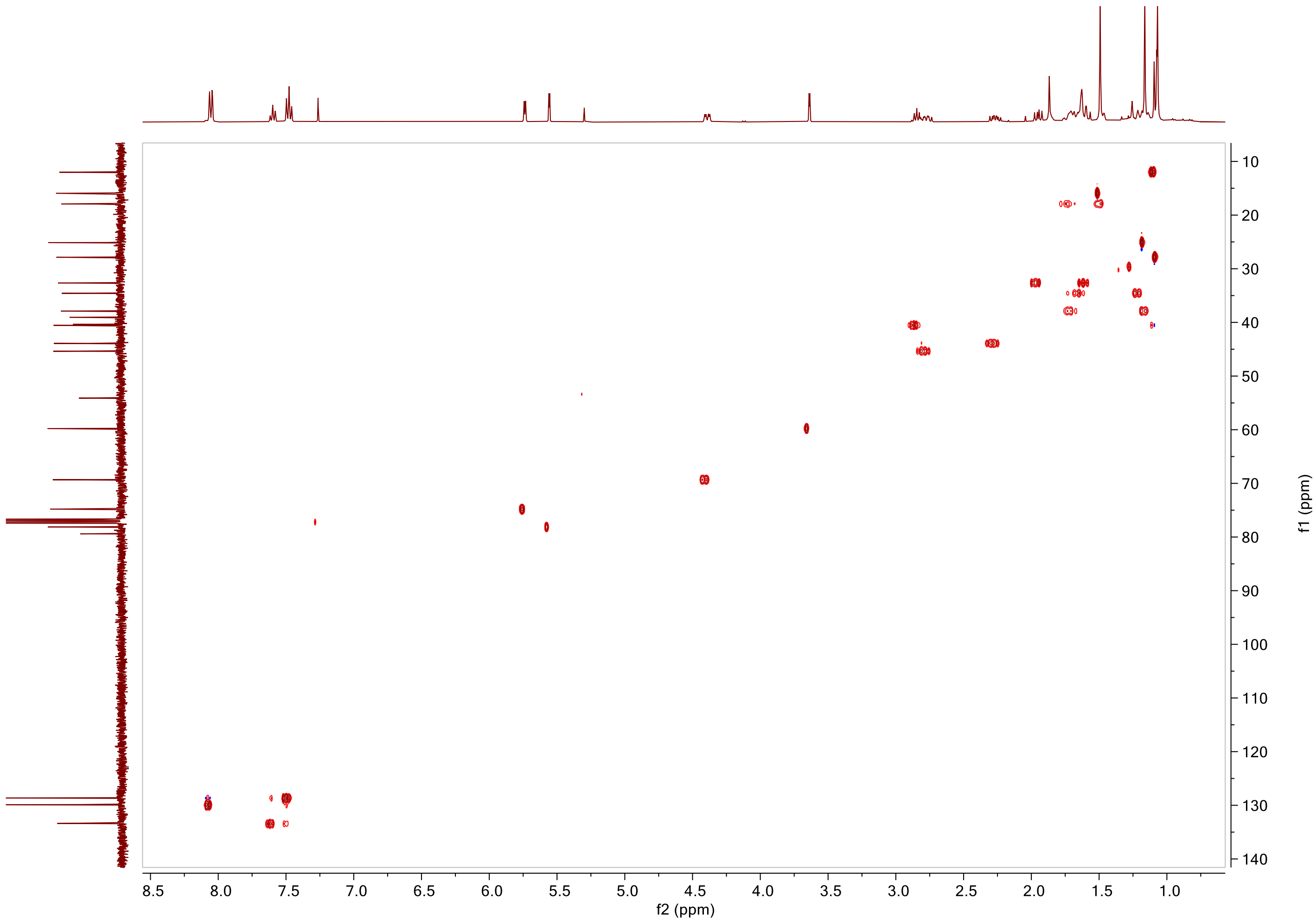

Figure S44. HSQC spectrum of (+)-(5*R*,6*R*,7*R*,8*S*,9*S*,10*R*,13*S*,14*R*,15*R*,16*S*)-6-benzoyloxy-15,16-epoxy-5,7-dihydroxy-13-spirocassan-12,16-olide (spirocaesalmin G) (**4a**), (400/100 MHz, $CDCl_3$).

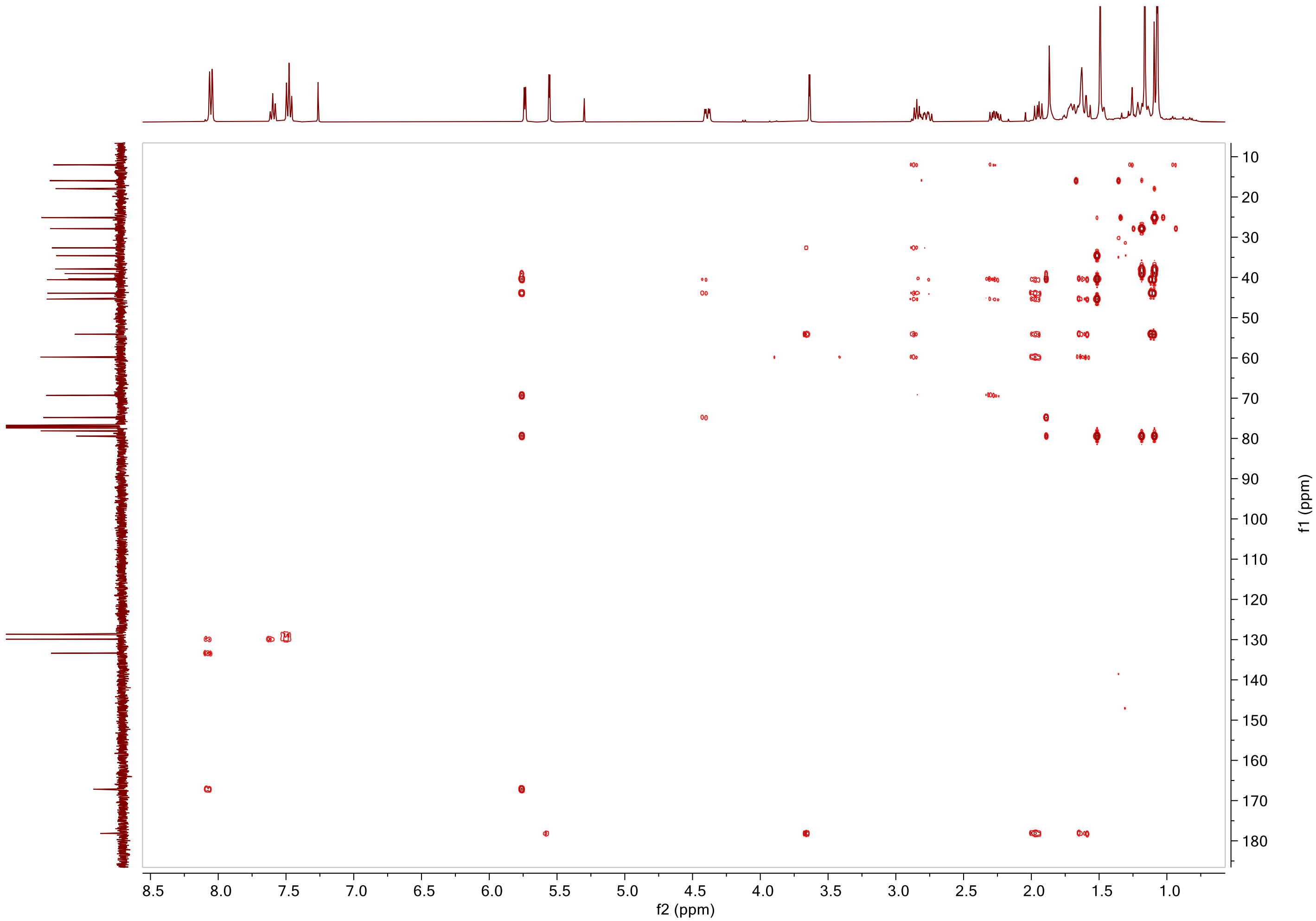

Figure S45. HMBC spectrum of (+)-(5*R*,6*R*,7*R*,8*S*,9*S*,10*R*,13*S*,14*R*,15*R*,16*S*)-6-benzoyloxy-15,16-epoxy-5,7-dihydroxy-13-spirocassan-12,16-olide (spirocaesalmin G) (**4a**), (400/100 MHz, $CDCl_3$).

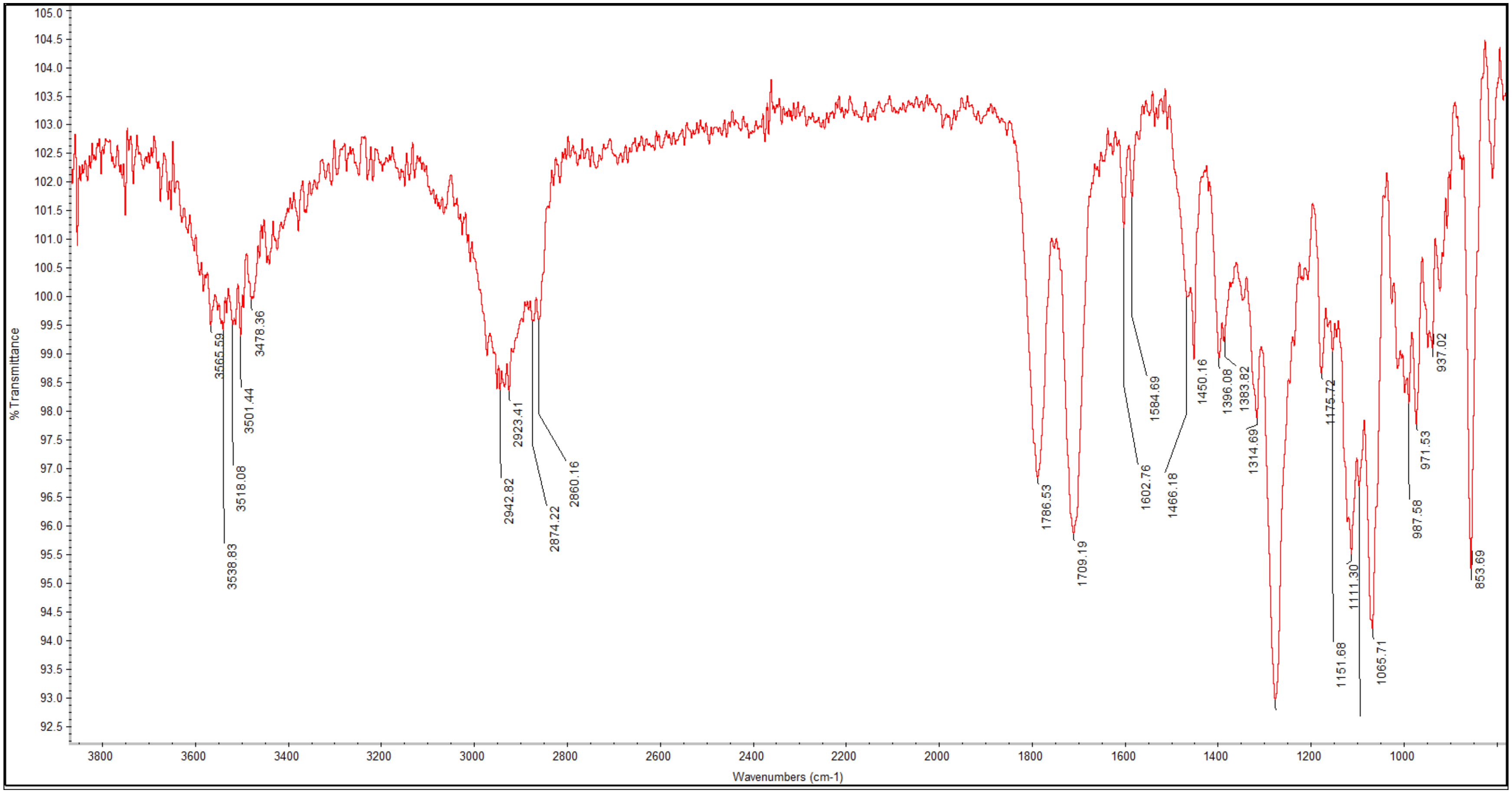

Figure S46. IR spectrum of (+)-(5*R*,6*R*,7*R*,8*S*,9*S*,10*R*,13*S*,14*R*,15*R*,16*S*)-6-benzoyloxy-15,16-epoxy-5,7-dihydroxy-13-spirocassan-12,16-olide (spirocaesalmin G) (**4a**).

Elemental Composition Report

Single Mass Analysis
Tolerance = 5.0 PPM / DBE: min = -1.5, max = 150.0
Element prediction: Off
Number of isotope peaks used for i-FIT = 3

Monoisotopic Mass, Even Electron Ions
228 formula(e) evaluated with 2 results within limits (all results (up to 1000) for each mass)
Elements Used:
C: 0-100 H: 0-100 O: 0-10 Br: 0-1 Na: 0-1

Armando TALAVERA 10-May-2023
Injdir - dilution 200
AT-2023-1-(01) 11 (0.098) Cm (11:14-1:5) 1: TOF MS ES+ 2.29e+003

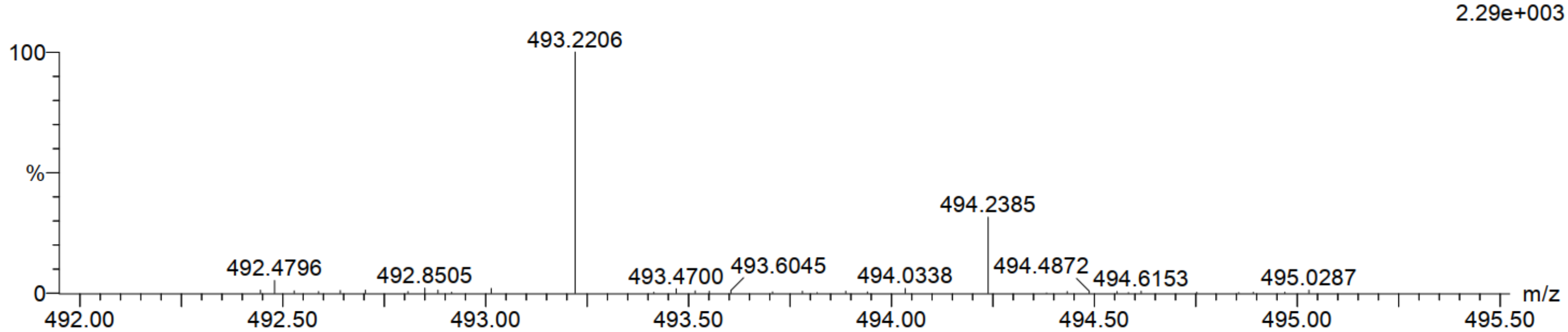

Minimum:				-1.5			
Maximum:		5.0	5.0	150.0			
Mass	Calc. Mass	mDa	PPM	DBE	i-FIT	i-FIT (Norm)	Formula
493.2206	493.2202	0.4	0.8	10.5	137.3	0.7	C27 H34 O7 Na
	493.2226	-2.0	-4.1	13.5	137.3	0.7	C29 H33 O7

Figure S47. HRMS spectrum of (+)-(5*R*,6*R*,7*R*,8*S*,9*S*,10*R*,13*S*,14*R*,15*R*,16*S*)-6-benzoyloxy-15,16-epoxy-5,7-dihydroxy-13-spirocassan-12,16-olide (spirocaesalmin G) (**4a**).

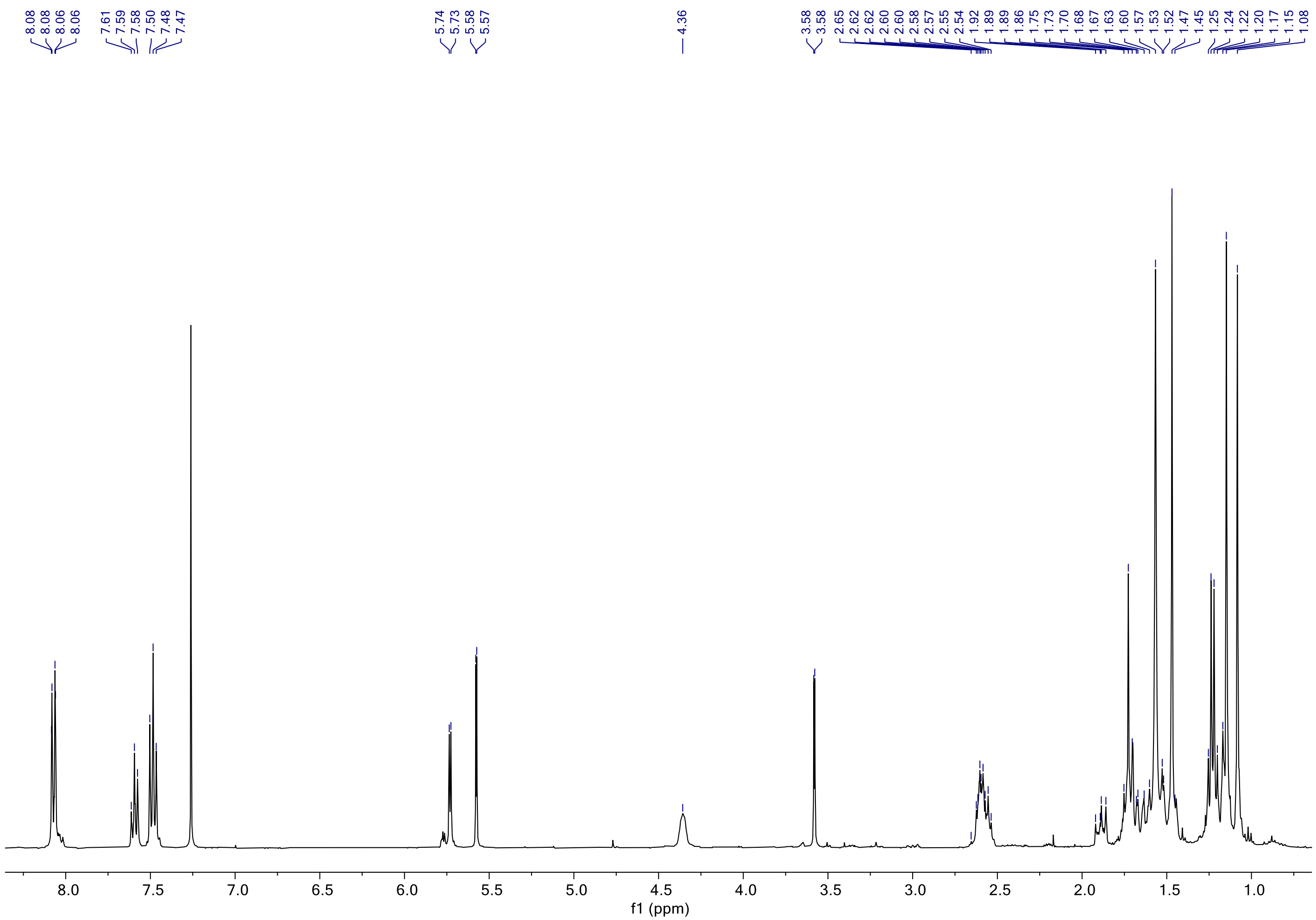

Figure S48. ^{1}H NMR spectrum of (−)-(5*R*,6*R*,7*R*,8*S*,9*S*,10*R*,13*R*,14*R*,15*S*,16*R*)-6-benzoyloxy-15,16-epoxy-5,7-dihydroxy-13-spirocassan-12,16-olide (isospirocaesalmin G) (**4b**), (400 MHz, $CDCl_3$).

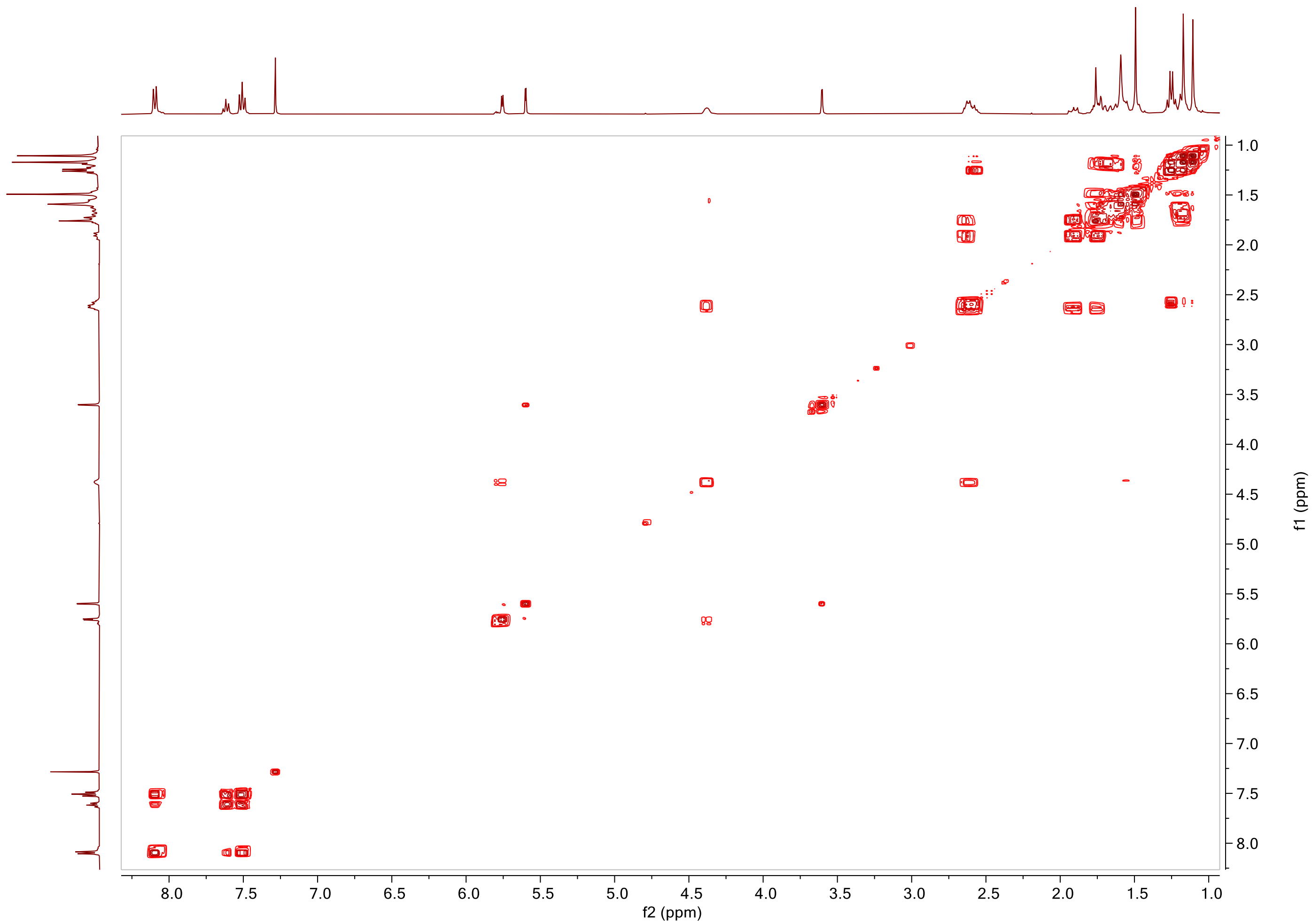

Figure S49. COSY spectrum of (−)-(5*R*,6*R*,7*R*,8*S*,9*S*,10*R*,13*R*,14*R*,15*S*,16*R*)-6-benzoyloxy-15,16-epoxy-5,7-dihydroxy-13-spirocassan-12,16-olide (isospirocaesalmin G) (**4b**), (400 MHz, $CDCl_3$).

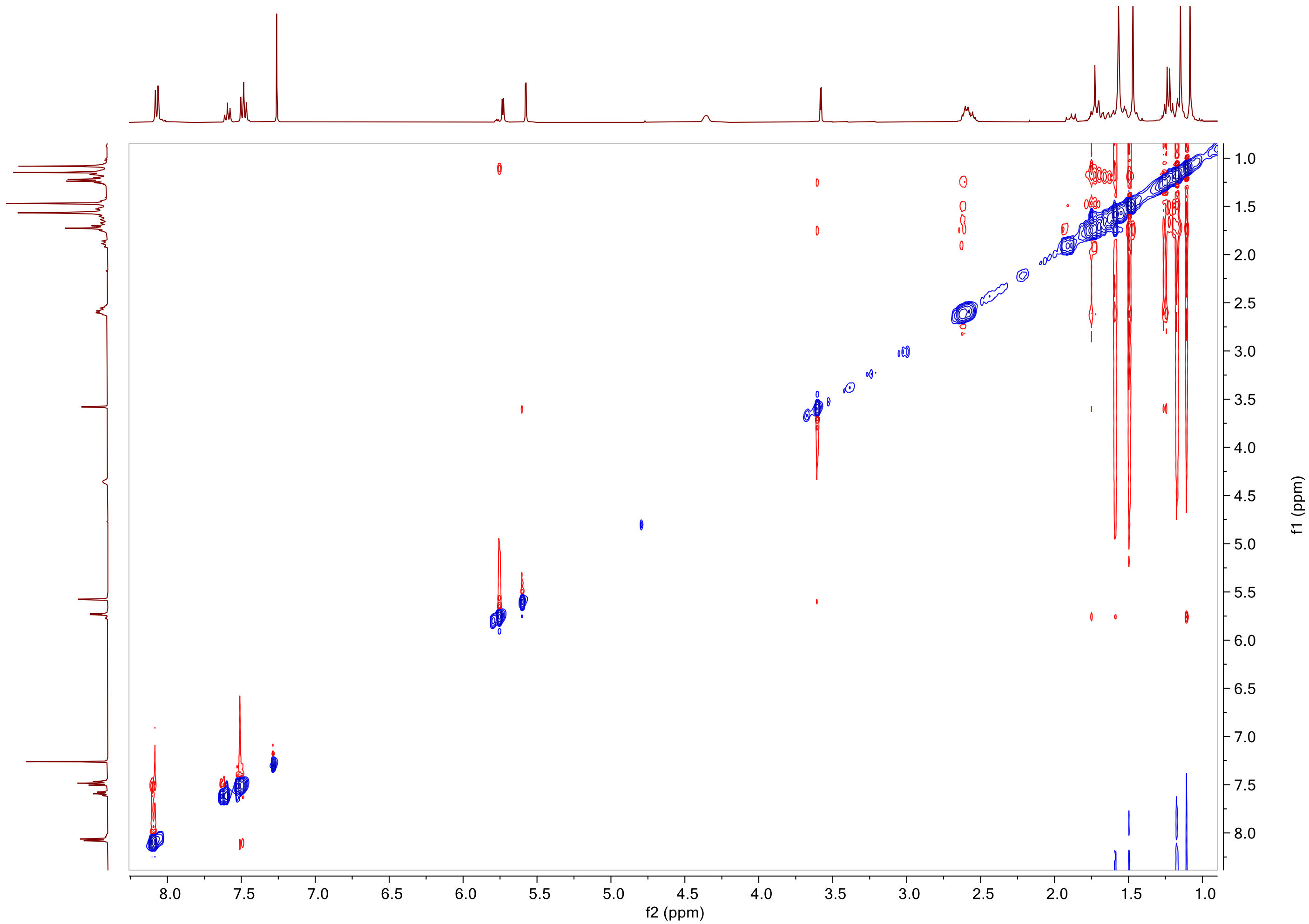

Figure S50. NOESY spectrum of (−)-(5*R*,6*R*,7*R*,8*S*,9*S*,10*R*,13*R*,14*R*,15*S*,16*R*)-6-benzoyloxy-15,16-epoxy-5,7-dihydroxy-13-spirocassan-12,16-olide (isospirocaesalmin G) (**4b**), (400 MHz, $CDCl_3$).

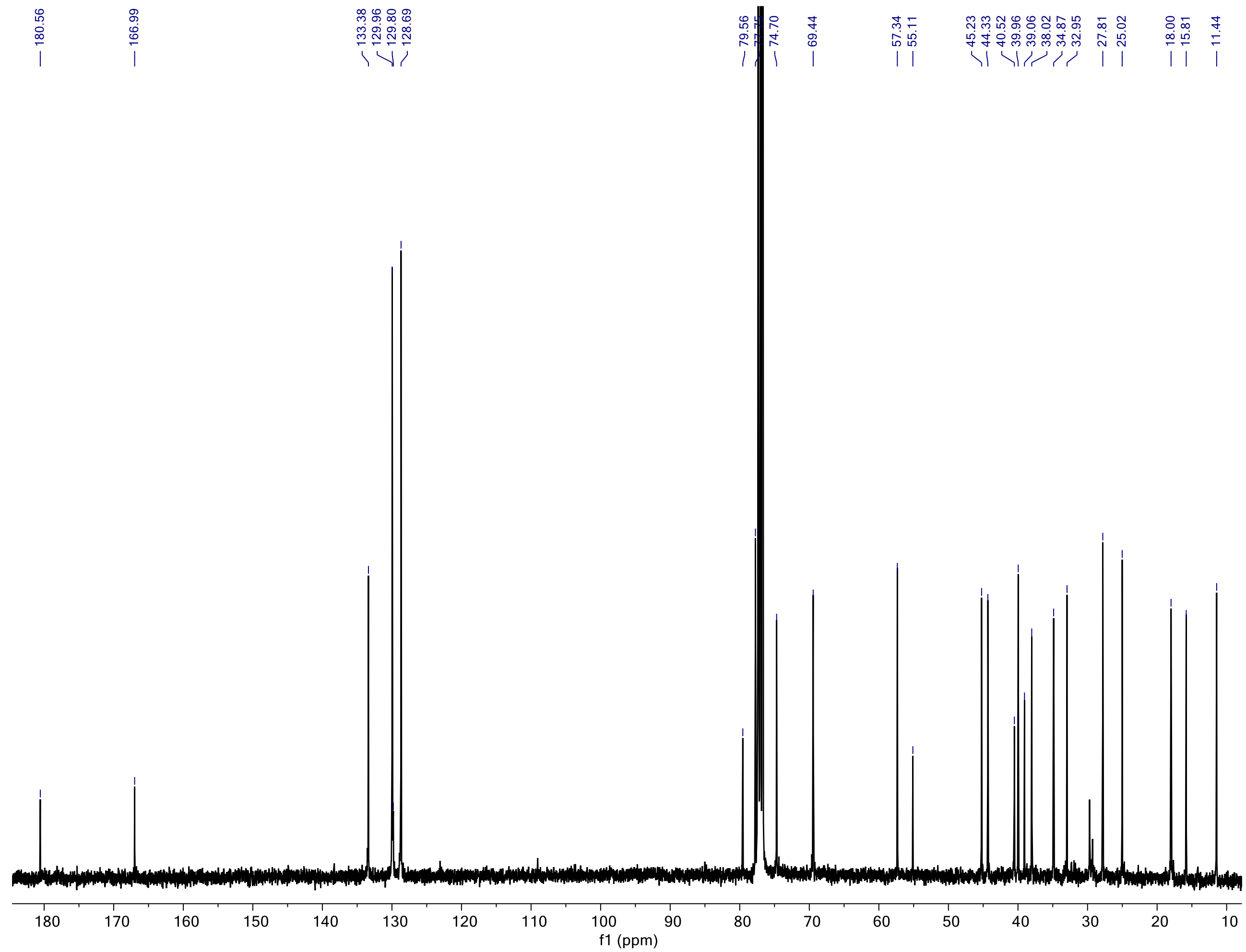

Figure S51. ^{13}C NMR spectrum of (−)-(5*R*,6*R*,7*R*,8*S*,9*S*,10*R*,13*R*,14*R*,15*S*,16*R*)-6-benzoyloxy-15,16-epoxy-5,7-dihydroxy-13-spirocassan-12,16-olide (isospirocaesalmin G) (**4b**), (100 MHz, $CDCl_3$).

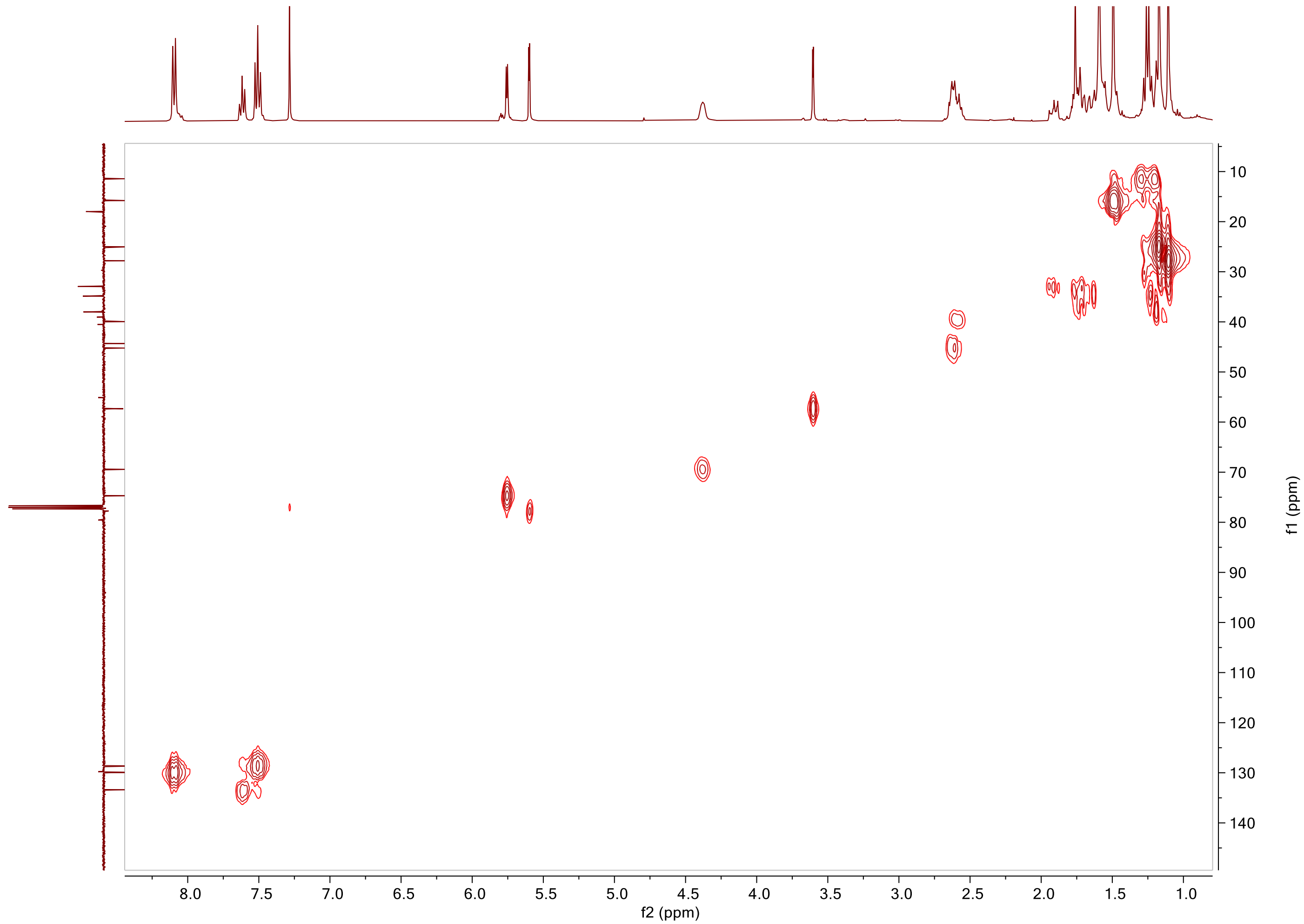

Figure S52. HMQC spectrum of (−)-(5*R*,6*R*,7*R*,8*S*,9*S*,10*R*,13*R*,14*R*,15*S*,16*R*)-6-benzoyloxy-15,16-epoxy-5,7-dihydroxy-13-spirocassan-12,16-olide (isospirocaesalmin G) (**4b**), (400/100 MHz, $CDCl_3$).

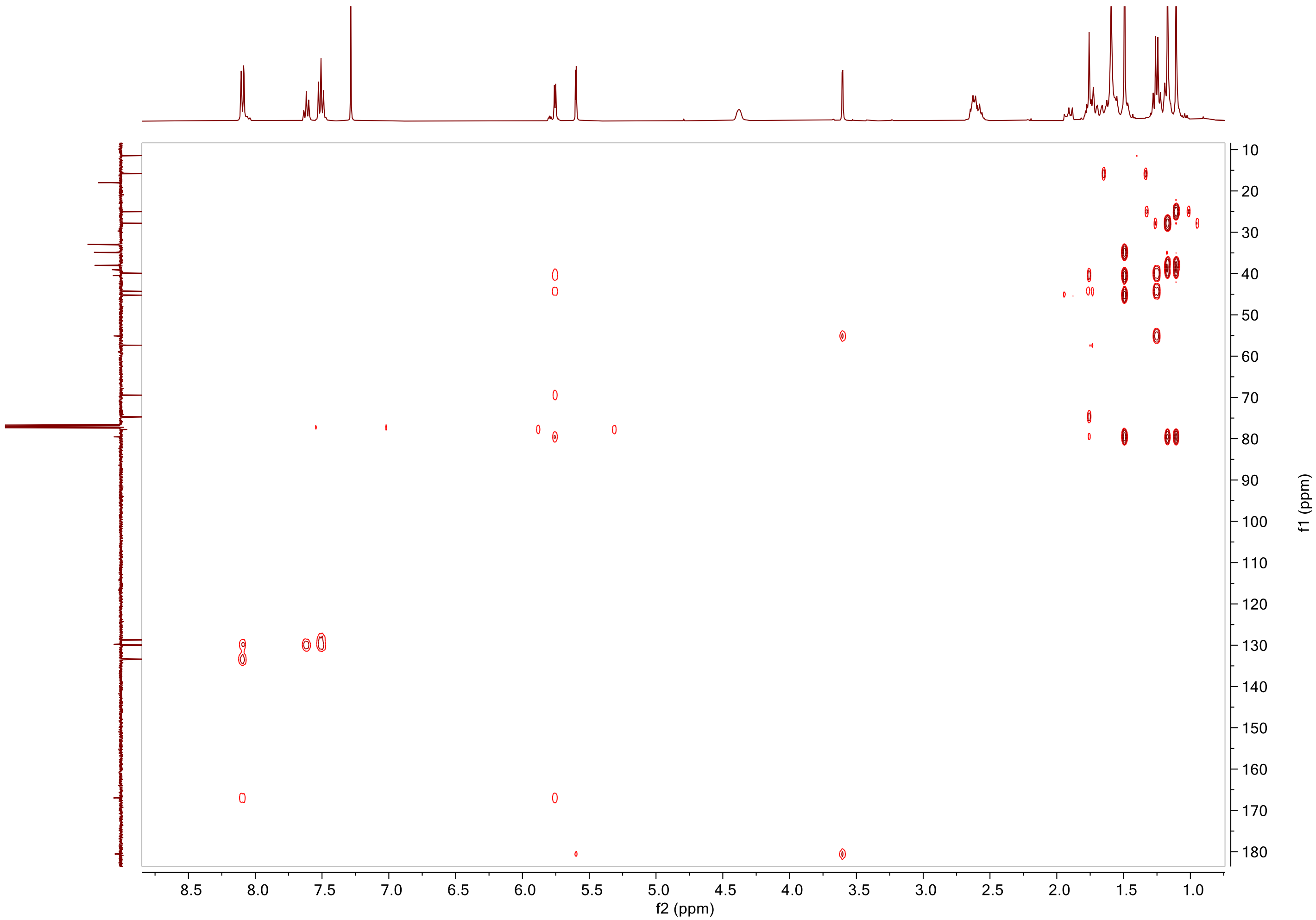

Figure S53. HMBC spectrum of (−)-(5*R*,6*R*,7*R*,8*S*,9*S*,10*R*,13*R*,14*R*,15*S*,16*R*)-6-benzoyloxy-15,16-epoxy-5,7-dihydroxy-13-spirocassan-12,16-olide (isospirocaesalmin G) (**4b**), (400/100 MHz, $CDCl_3$).

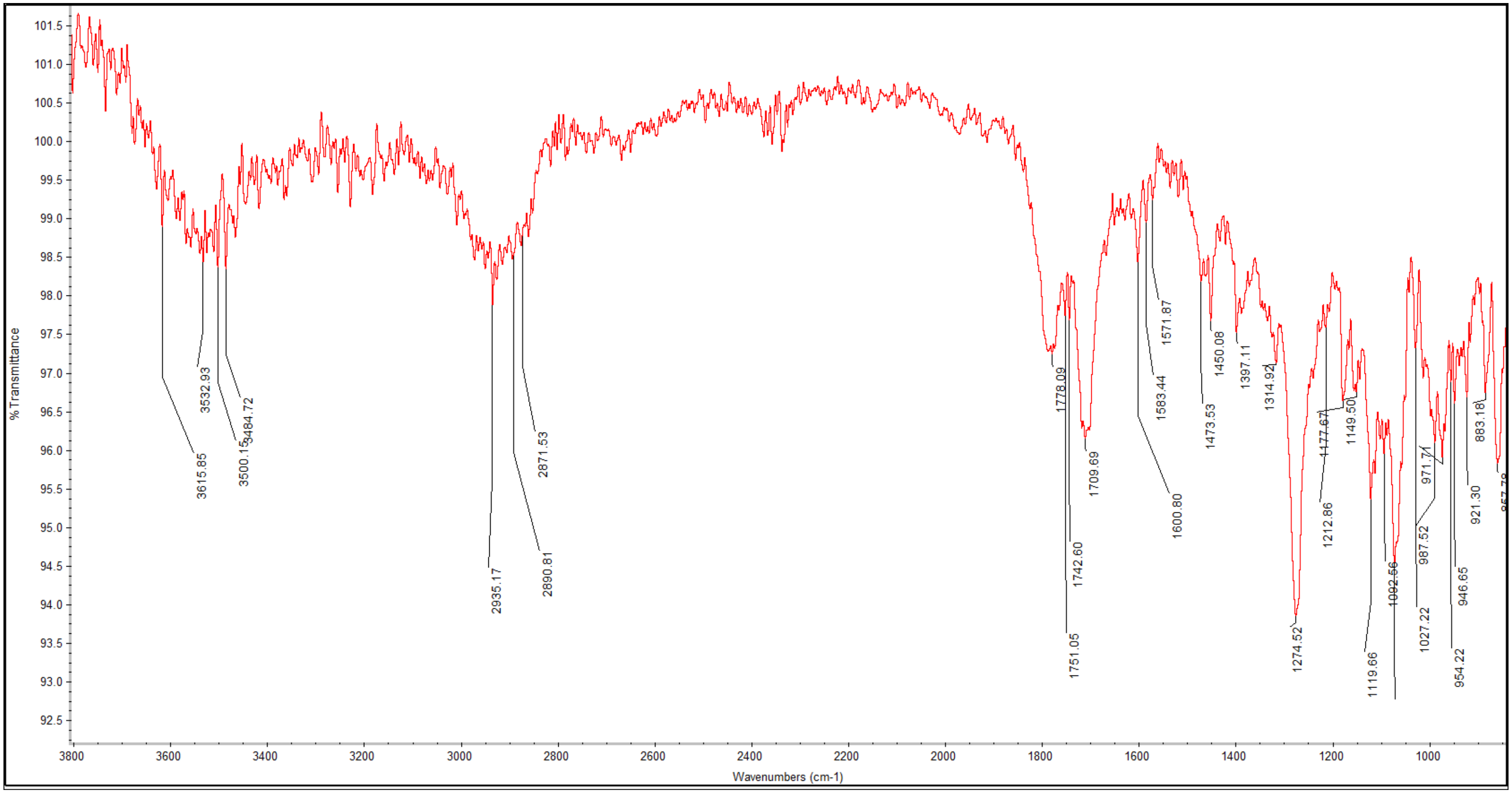

Figure S54. IR spectrum of (−)-(5*R*,6*R*,7*R*,8*S*,9*S*,10*R*,13*R*,14*R*,15*S*,16*R*)-6-benzoyloxy-15,16-epoxy-5,7-dihydroxy-13-spirocassan-12,16-olide (isospirocaesalmin G) (**4b**).

Elemental Composition Report

Page 1

Single Mass Analysis

Tolerance = 5.0 PPM / DBE: min = -1.5, max = 150.0
Element prediction: Off
Number of isotope peaks used for i-FIT = 3

Monoisotopic Mass, Even Electron Ions
115 formula(e) evaluated with 1 results within limits (all results (up to 1000) for each mass)
Elements Used:
C: 0-100 H: 0-100 O: 0-10 Br: 0-1

Armando TALAVERA
Injdir - dilution 200
AT-2023-2-(01) 17 (0.138) Cm (17:25-1:6)

10-May-2023
1: TOF MS ES+
9.13e+003

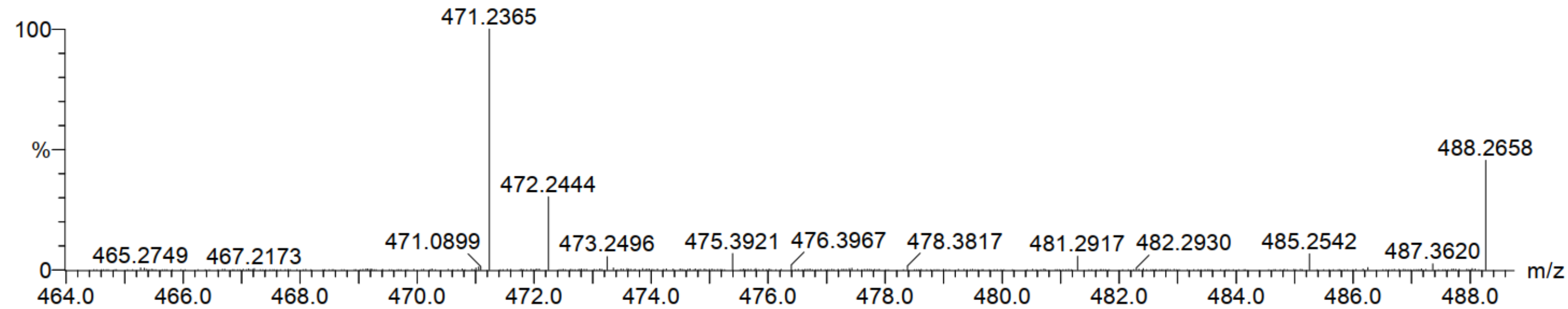

Minimum:				-1.5			
Maximum:		5.0	5.0	150.0			
Mass	Calc. Mass	mDa	PPM	DBE	i-FIT	i-FIT (Norm)	Formula
471.2365	471.2383	-1.8	-3.8	10.5	173.3	0.0	C27 H35 O7

Figure S55. HRMS spectrum of (−)-(5*R*,6*R*,7*R*,8*S*,9*S*,10*R*,13*R*,14*R*,15*S*,16*R*)-6-benzoyloxy-15,16-epoxy-5,7-dihydroxy-13-spirocassan-12,16-olide (isospirocaesalmin G) (**4b**).

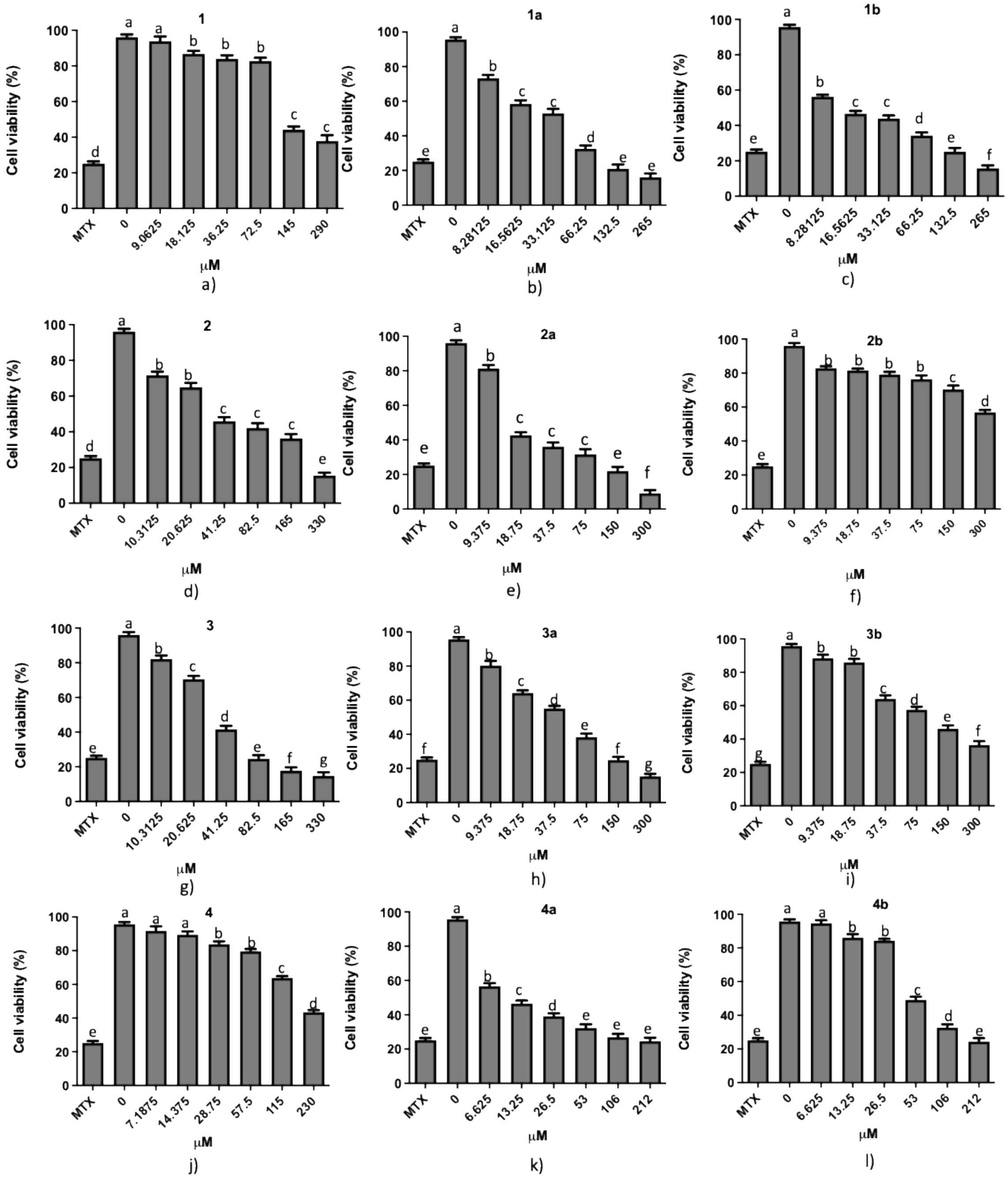

Figure S56. Effect of compounds **1**, **1a**, **1b**, **2**, **2a**, **2b**, **3**, **3a**, **3b**, **4**, **4a**, and **4b** on HeLa cell viability. HeLa cells were incubated containing these compounds for 24 h. (a) Viability was determined by the MTT assay and quantitation of fluorescence. Bars represent the mean value ± the standard error (SE) of three independent experiments. One-way analysis of variance was carried out, with Tukey's post hoc test; $n = 3$. Values for SE ($P < 0.05$) are shown in lowercase letters.

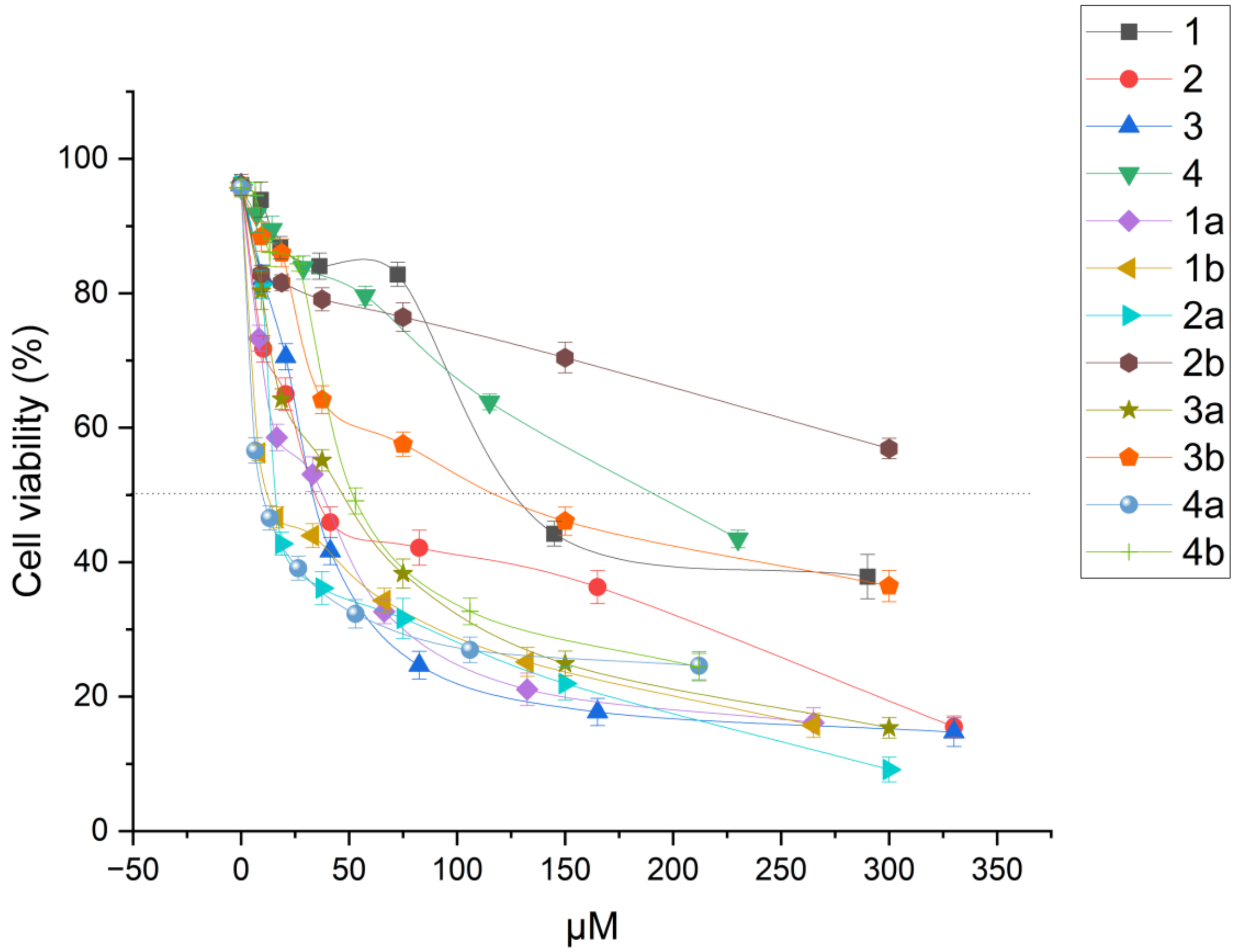

Figure S57. Dose-response effect of compounds **1-4**, **1a**, **1b**, **2a**, **2b**, **3a**, **3b**, **4a**, and **4b** on HeLa cell viability. HeLa cells were incubated containing these compounds for 24 h. Cell viability was determined by the MTT assay and quantitation of fluorescence. Bars represent the mean value ± the standard error (SE) of three independent experiments.

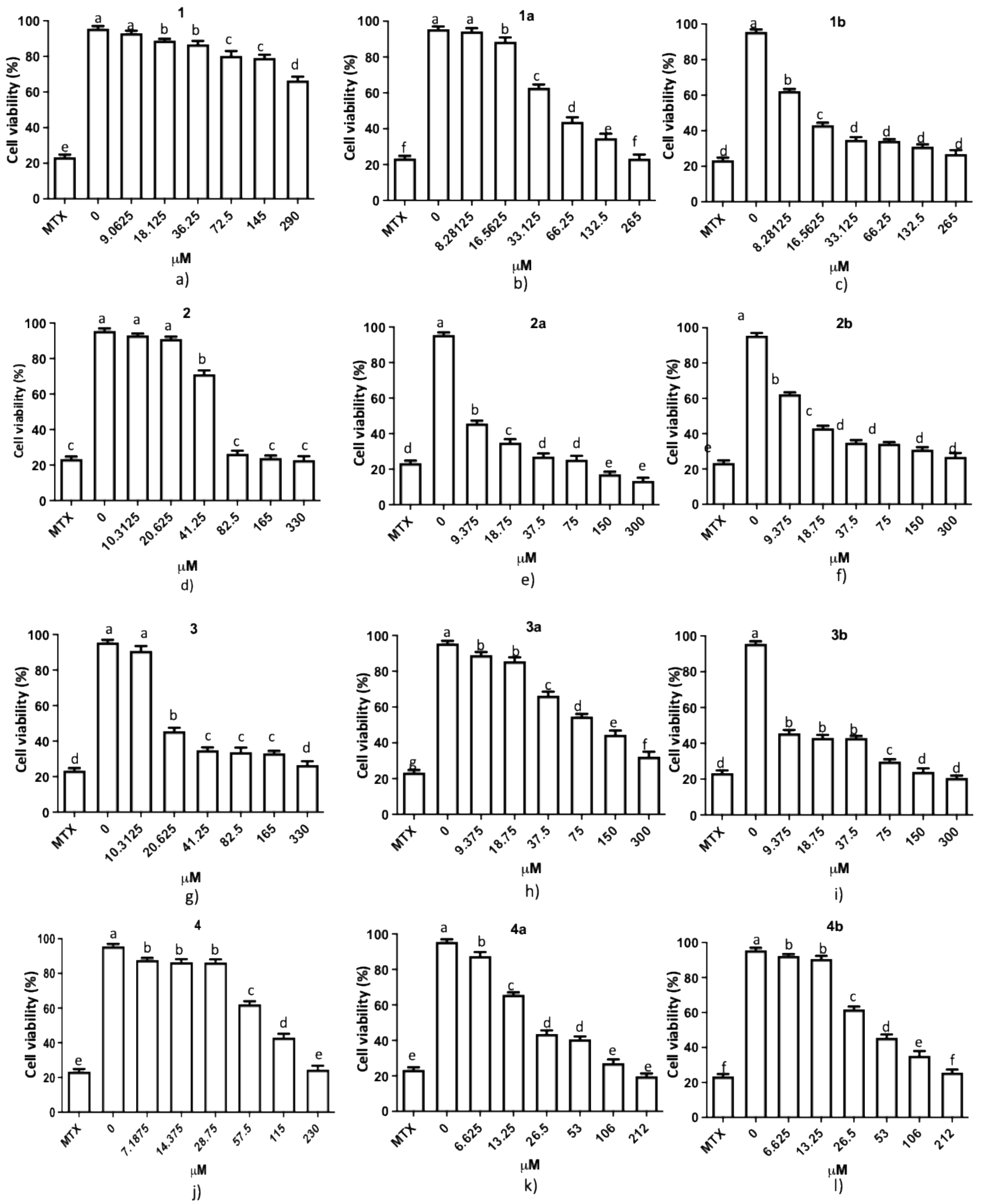

Figure S58. Effect of compounds **1**, **1a**, **1b**, **2**, **2a**, **2b**, **3**, **3a**, **3b**, **4**, **4a**, and **4b** on MDA-MB-231 cell viability. MDA-MB-231 cells were incubated containing these compounds for 24 h. (a) Viability was determined by the MTT assay and quantitation of fluorescence. Bars represent the mean value ± the standard error (SE) of three independent experiments. One-way analysis of variance was carried out, with Tukey's post hoc test; $n = 3$. Values for SE ($P < 0.05$) are shown in lowercase letters.

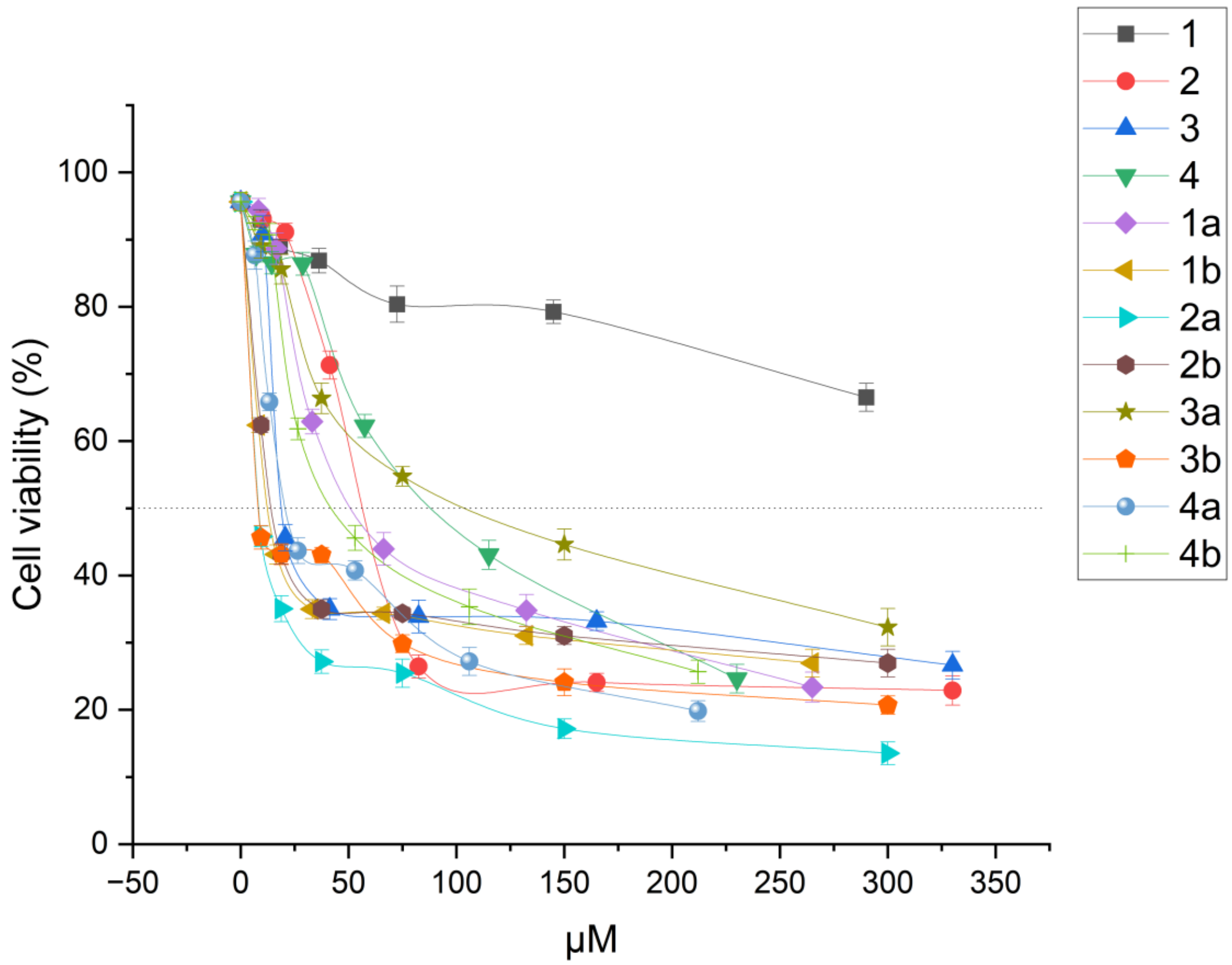

Figure S59. Dose-response effect of compounds **1-4**, **1a**, **1b**, **2a**, **2b**, **3a**, **3b**, **4a**, and **4b** on MDA-MB-231 cell viability. MDA-MB-231 cells were incubated containing these compounds for 24 h. (a) Cell viability was determined by the MTT assay and quantitation of fluorescence. Bars represent the mean value ± the standard error (SE) of three independent experiments.

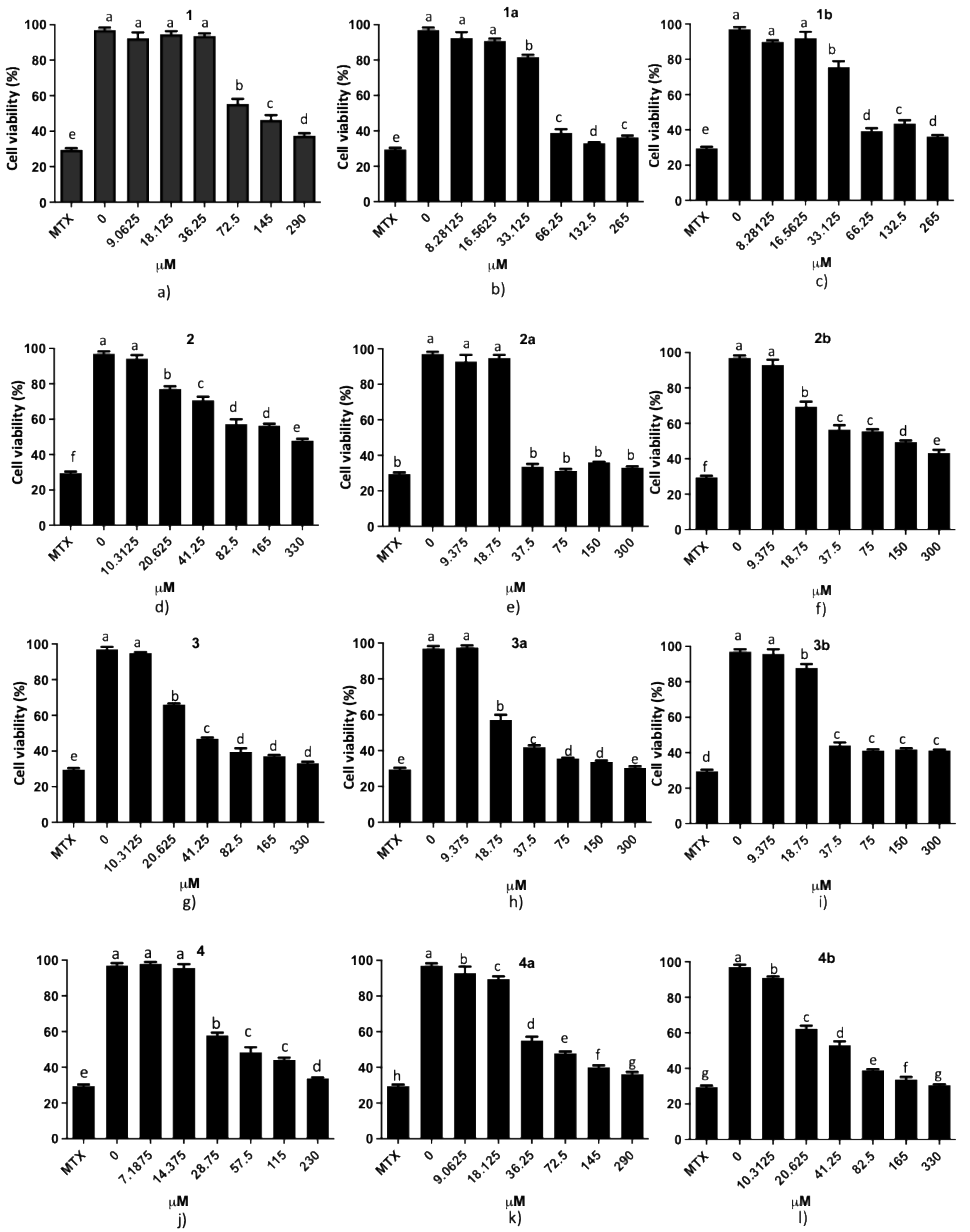

Figure S60. Effect of compounds **1**, **1a**, **1b**, **2**, **2a**, **2b**, **3**, **3a**, **3b**, **4**, **4a**, and **4b** on CCD-18Co Cell viability. CCD-18Co cells were incubated containing these compounds for 24 h. (a) Viability was determined by the MTT assay and quantitation of fluorescence. Bars represent the mean value ± the standard error (SE) of three independent experiments. One-way analysis of variance was carried out, with Tukey's post hoc test; $n = 3$. Values for SE ($P < 0.05$) are shown in lowercase letters.

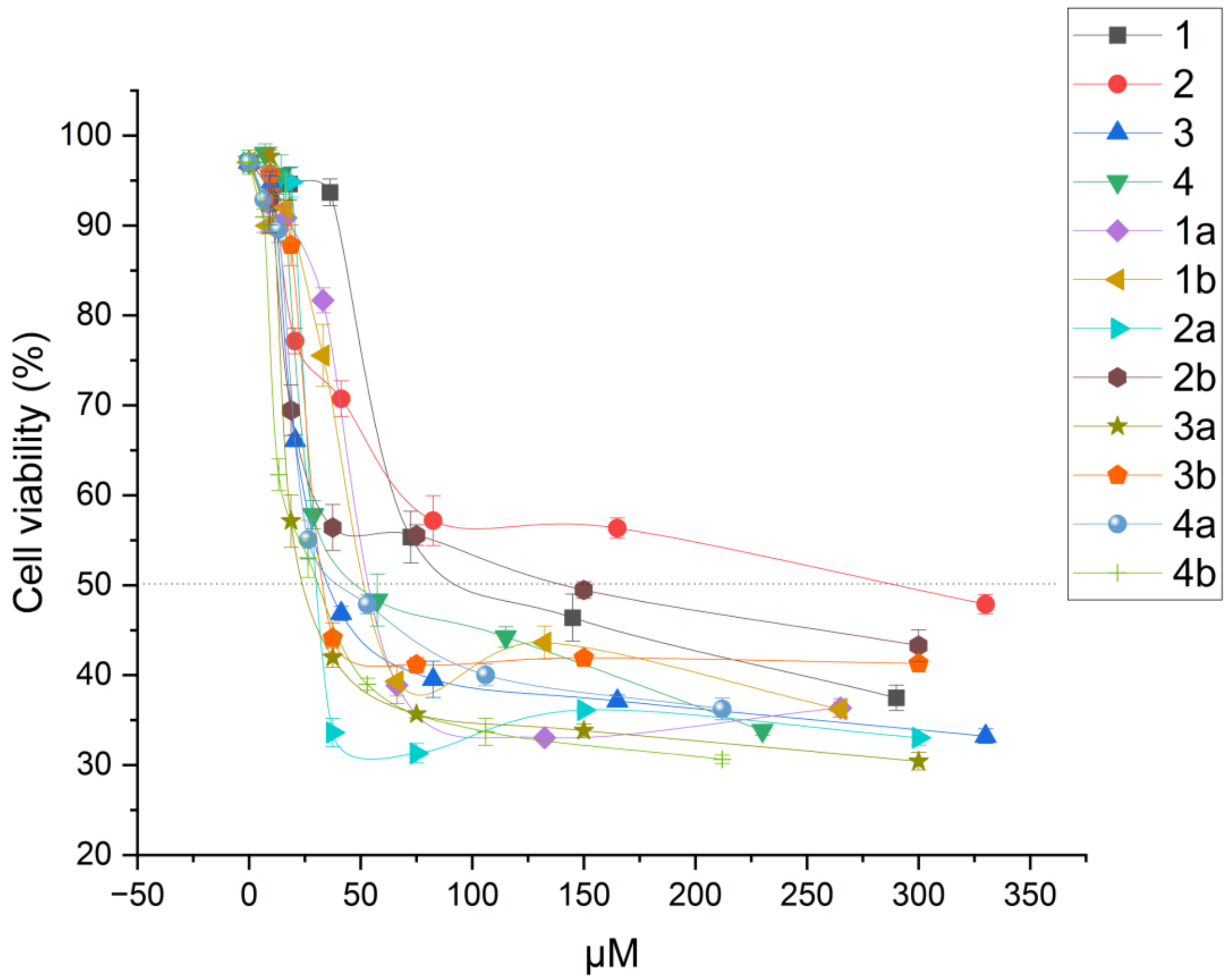

Figure S61. Dose-response effect of compounds **1-4**, **1a**, **1b**, **2a**, **2b**, **3a**, **3b**, **4a**, and **4b** on CCD-18Co cell viability. CCD-18Co cells were incubated containing these compounds for 24 h. Cell viability was determined by the MTT assay and quantitation of fluorescence. Bars represent the mean value ± the standard error (SE) of three independent experiments.

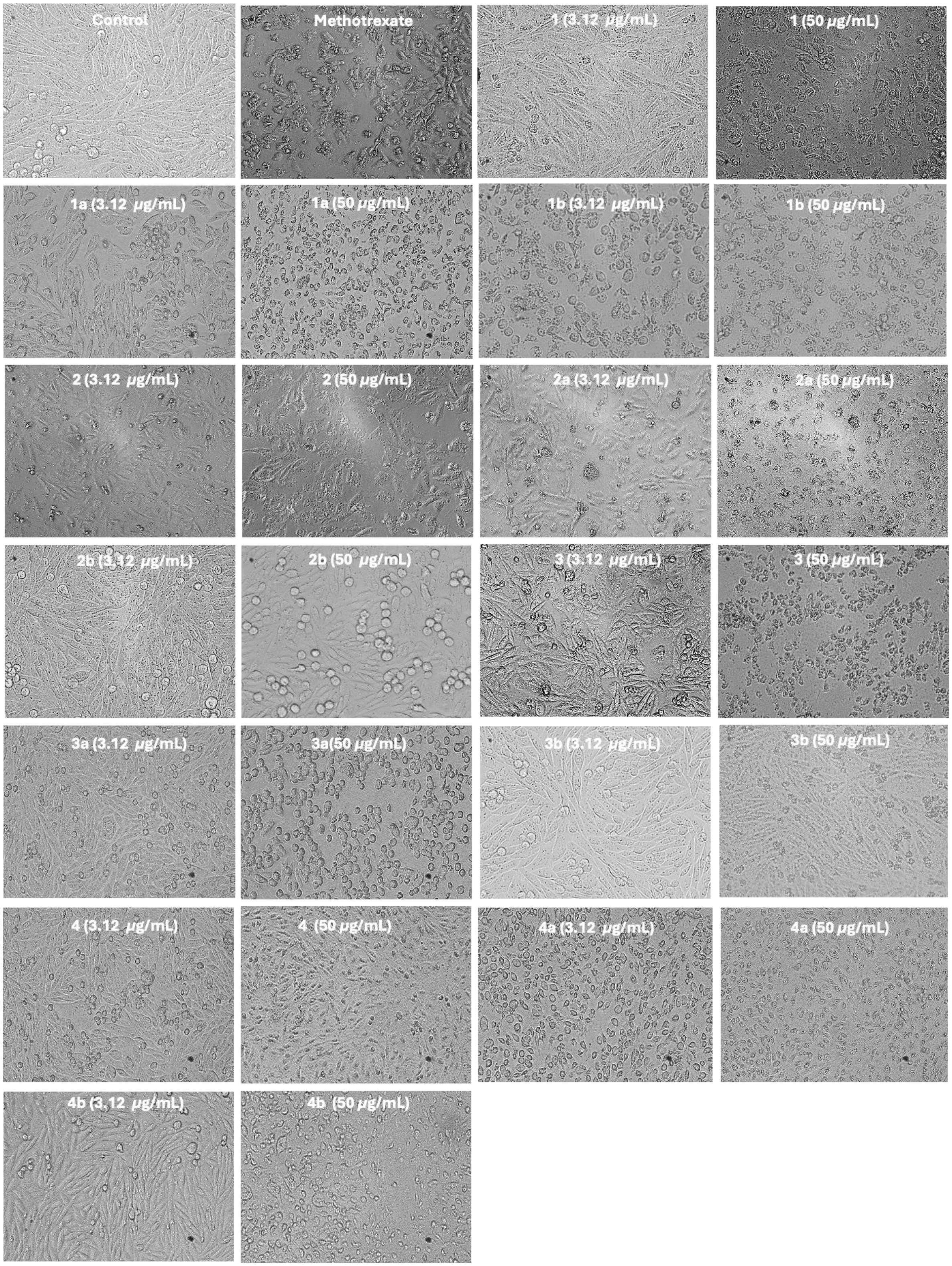

Figure S62. Morphological changes in HeLa cells, induced by compounds **1**, **1a**, **1b**, **2**, **2a**, **2b**, **3**, **3a**, **3b**, **4**, **4a**, and **4b**. Images of cells were taken under phase-contrast inverted microscopy after treatment with the compounds for 24 h. DMSO was used as a negative control at a concentration of 0.05%, and Methotrexate as positive control

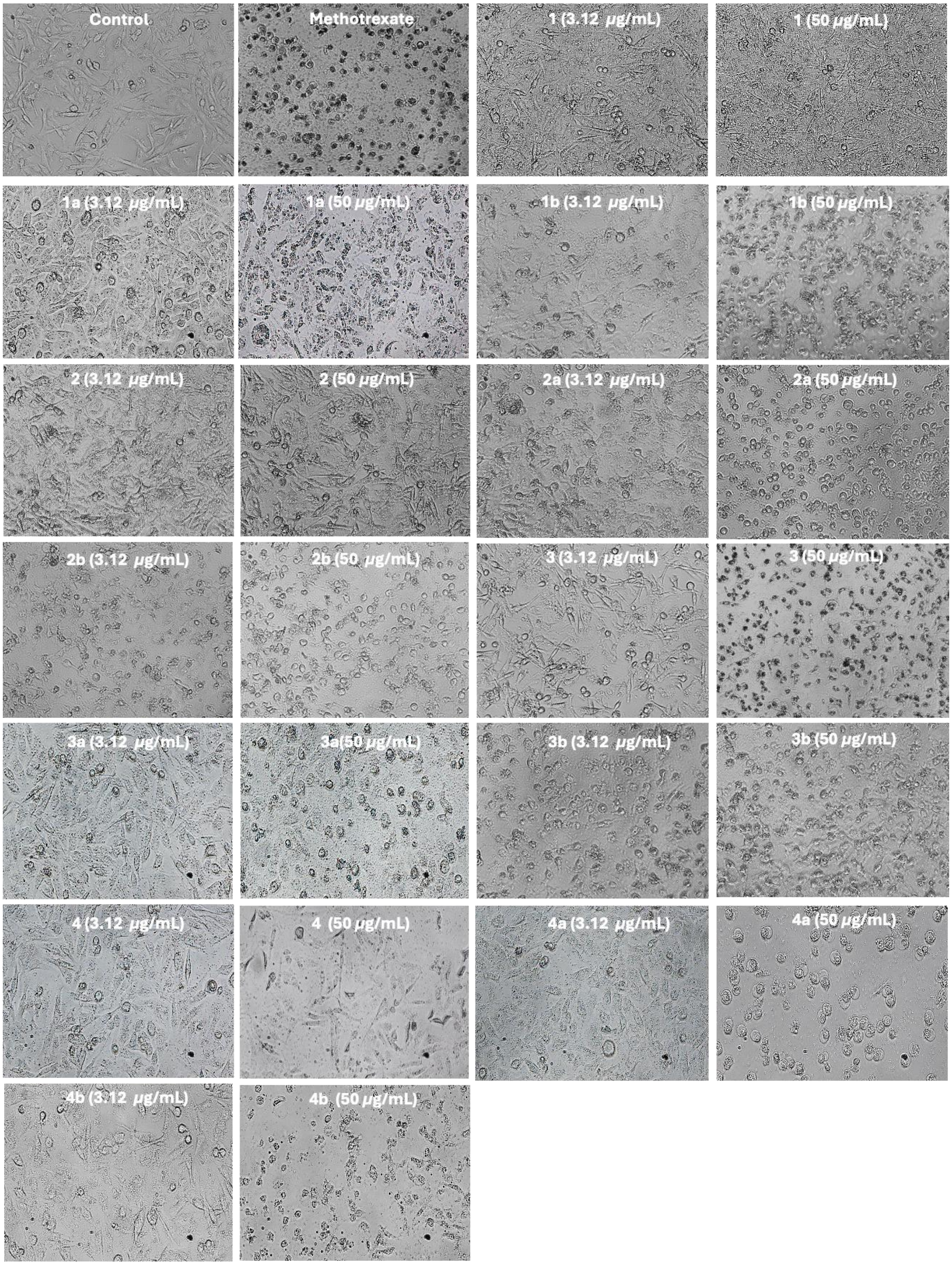

Figure S63. Morphological changes in MDA-MB-231 cells, induced by compounds **1**, **1a**, **1b**, **2**, **2a**, **2b**, **3**, **3a**, **3b**, **4**, **4a**, and **4b**. Images of cells were taken under phase-contrast inverted microscopy after treatment with the compounds for 24 h. DMSO was used as a negative control at a concentration of 0.05%, and Methotrexate as positive control.

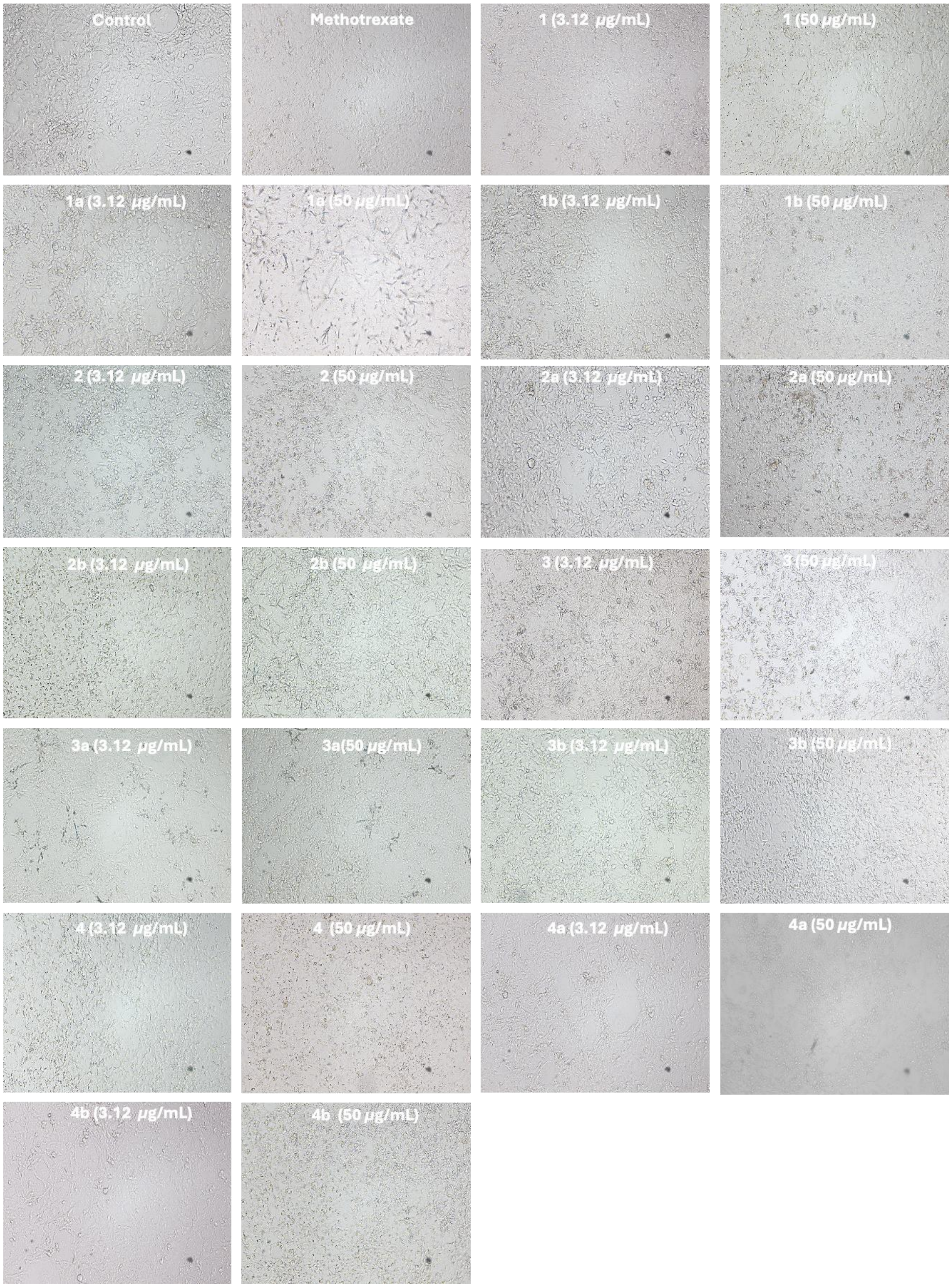

Figure S64. Morphological changes in CCD-18Co cells, induced by compounds **1**, **1a**, **1b**, **2**, **2a**, **2b**, **3**, **3a**, **3b**, **4**, **4a**, and **4b**. Images of cells were taken under phase-contrast inverted microscopy after treatment with the compounds for 24 h. DMSO was used as a negative control at a concentration of 0.05%, and Methotrexate as positive control.

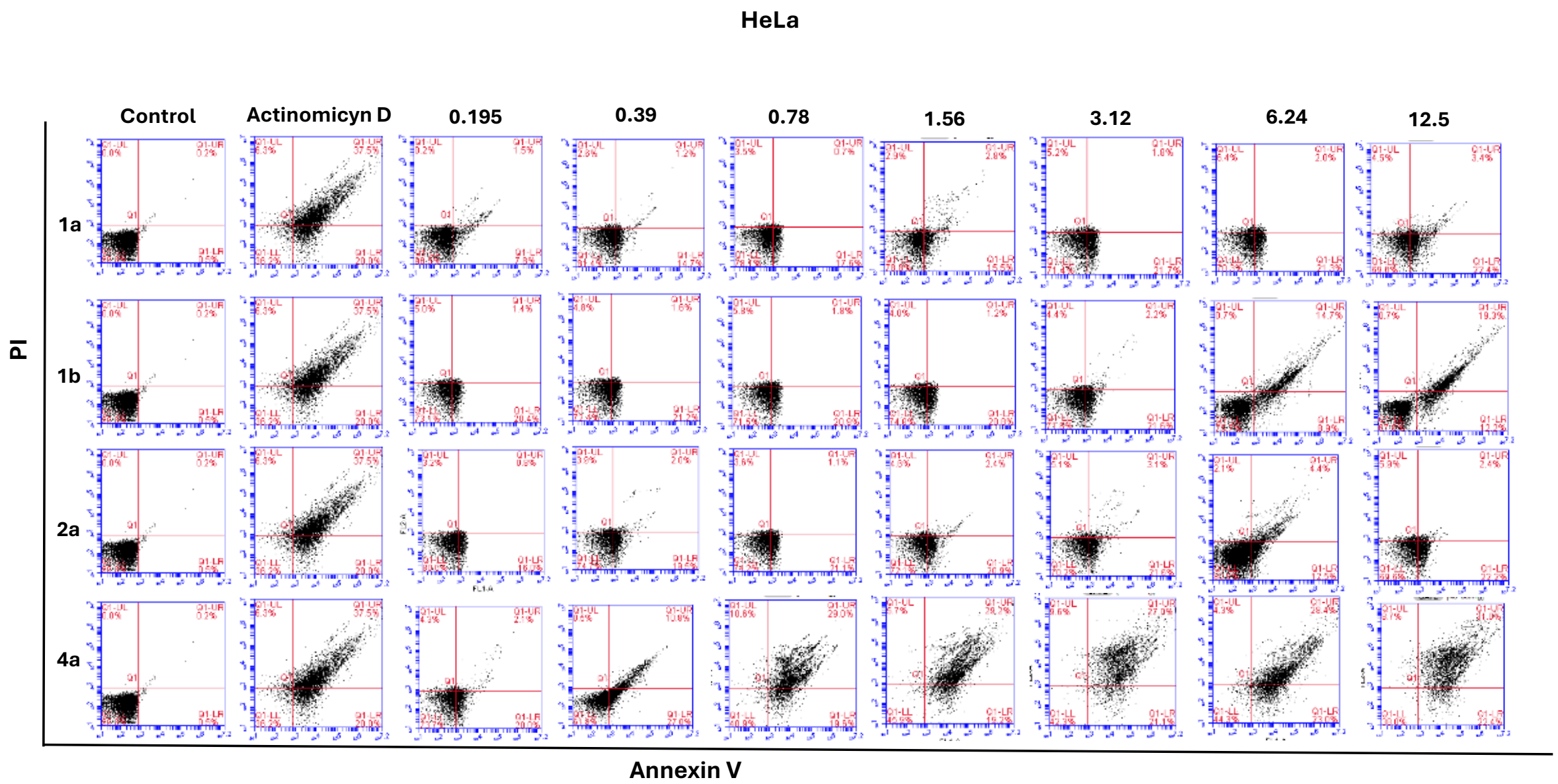

Figure S65. Induction of apoptosis in HeLa cells by compounds **1a**, **1b**, **2a**, and **4a**. HeLa cells were incubated with these compounds for 24 h. HeLa cells were stained with annexin V and propidium iodide and analyzed by flow cytometry. Schematic diagrams of dot plots showing the quadrant divisions for the determination of apoptosis using the annexin V and PI probes. The populations of cells for each of the treatments were gated in the forward scatter and side scatter analyses, in order to eliminate dead cells and cell debris. Populations corresponding to autofluorescence or basal fluorescence are located in the lower-left quadrants. Cells with increased fluorescence emission of at least one log unit are located in the lower-right quadrants. The percentage of fluorescent cells is indicated in the dot plots.

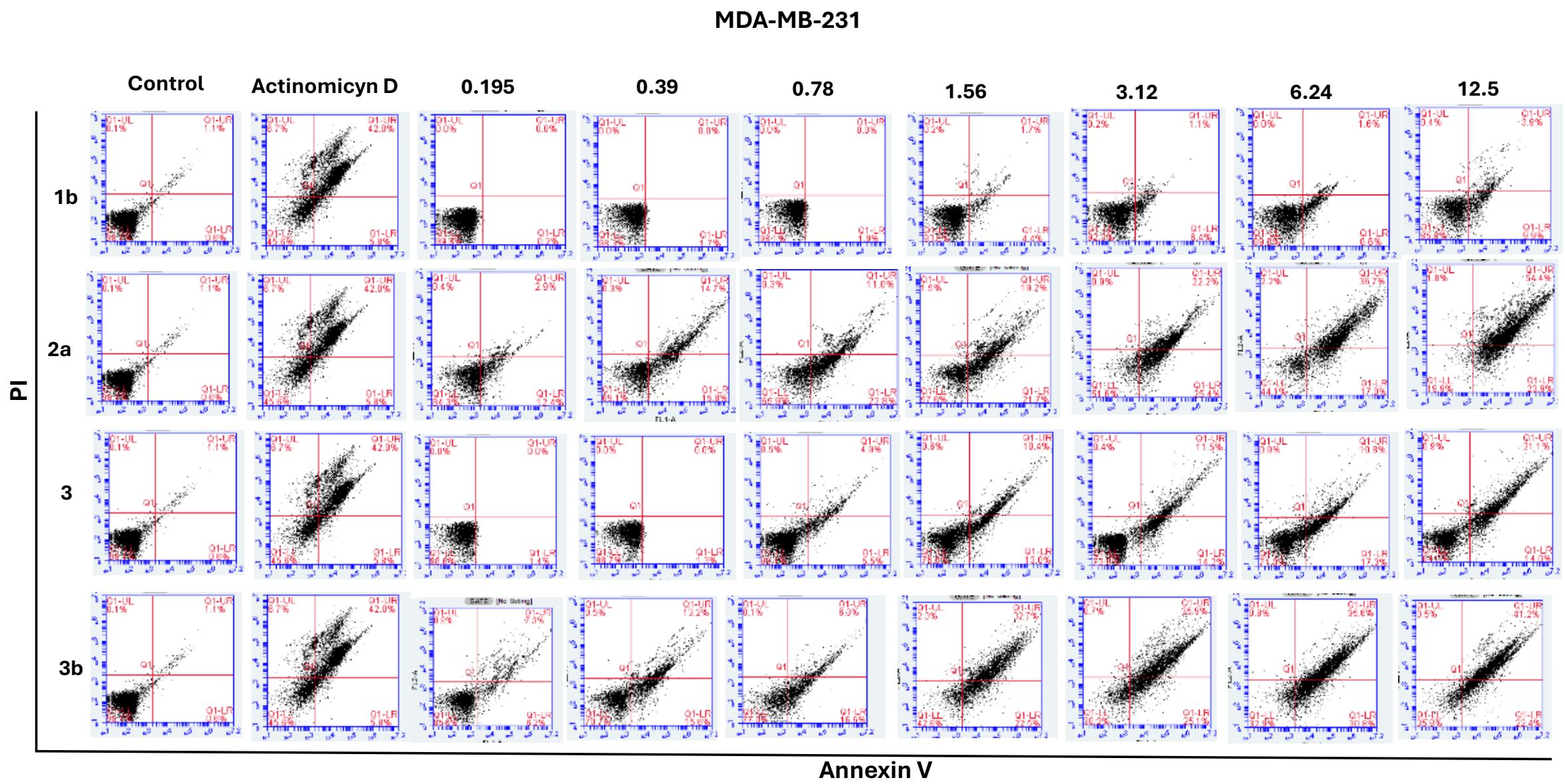

Figure S66. Induction of apoptosis in MDA-MB-231 cells by compounds **1b**, **2a**, **3**, and **3b**. MDA-MB-231 cells were incubated with these compounds for 24 h. MDA-MB-231 cells were stained with annexin V and propidium iodide and analyzed by flow cytometry. ((a)–(f)) Schematic diagrams of dot plots, showing the quadrant divisions for the determination of apoptosis using the annexin V and PI probes. The populations of cells for each of the treatments were gated in the forward scatter and side scatter analyses, in order to eliminate dead cells and cell debris. Populations corresponding to autofluorescence or basal fluorescence are located in the lower-left quadrants. Cells with increased fluorescence emission of at least one log unit are located in the lower-right quadrants. The percentage of fluorescent cells is indicated in the dot plots.

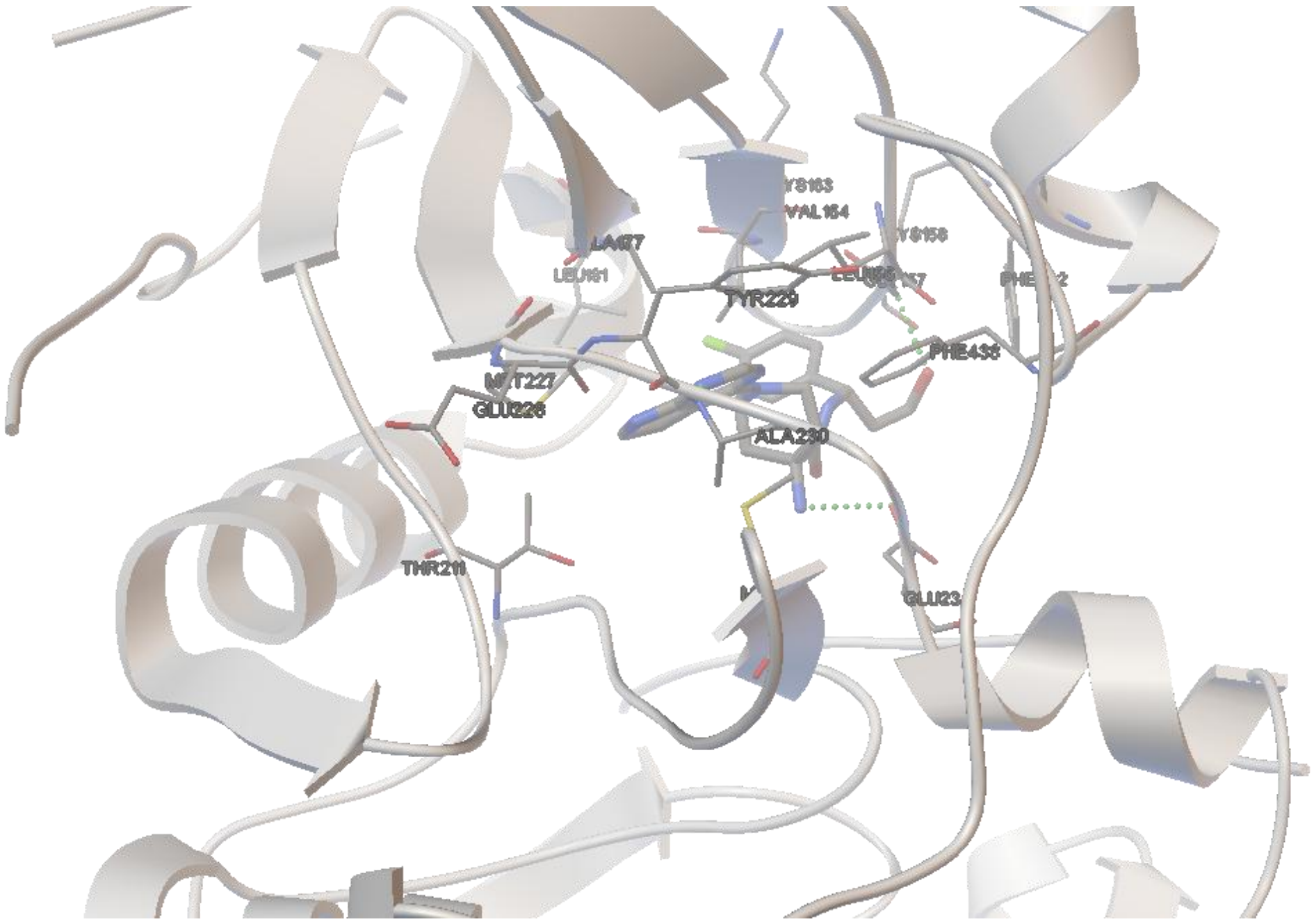

Figure S67. Redocking interactions of N-{(2S)-3-[(3S)-8',9'-dihydro-1H,3'H-spiro[piperidine-3,7'-pyrano[3,2-e]indazol]-1-yl]-2-hydroxypropyl}-N-(2-ethoxyethyl)-2,6-dimethylbenzenesulfonamide in the allosteric modulation ATP-binding site on Akt protein.

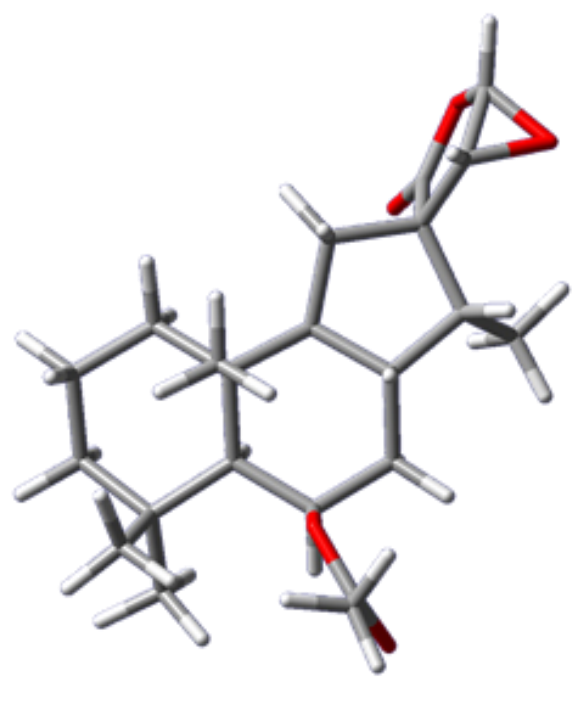

1a

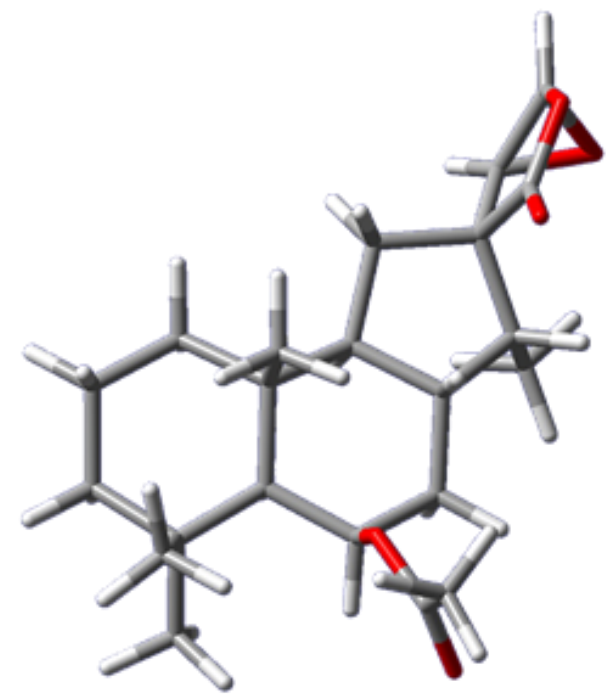

1b

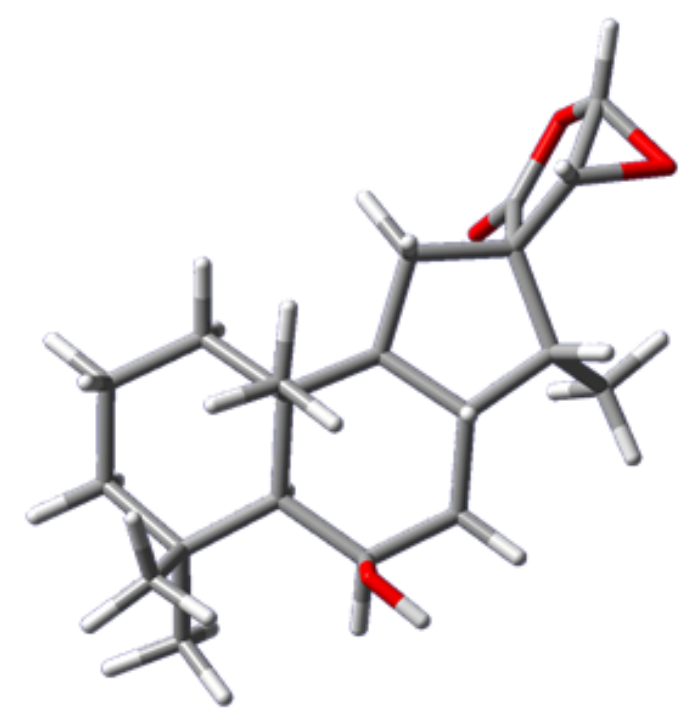

2a

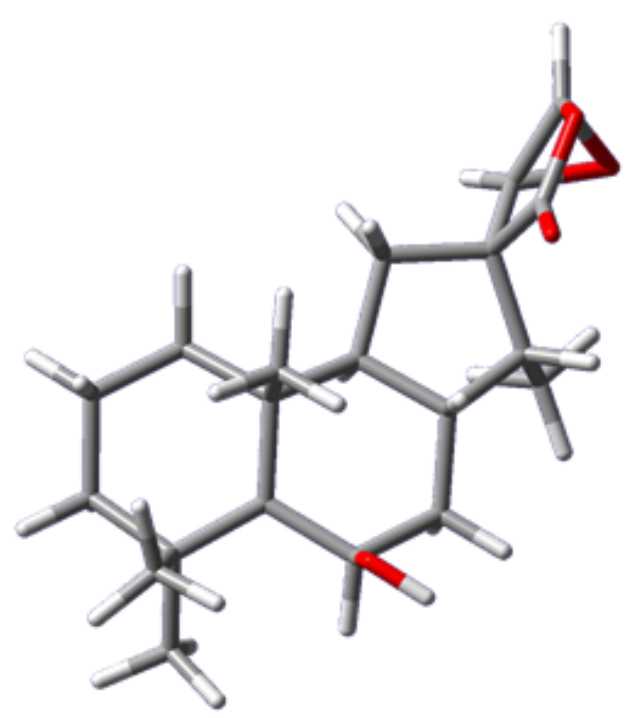

2b

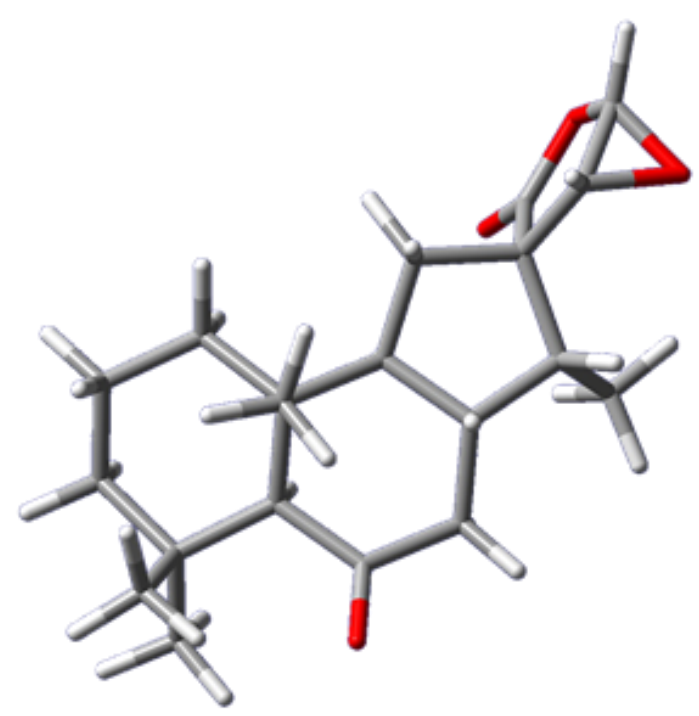

3a

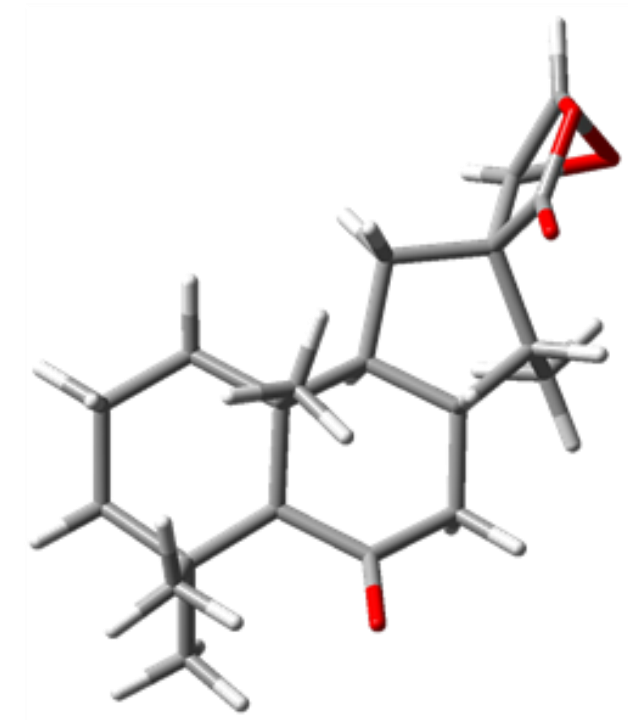

3b

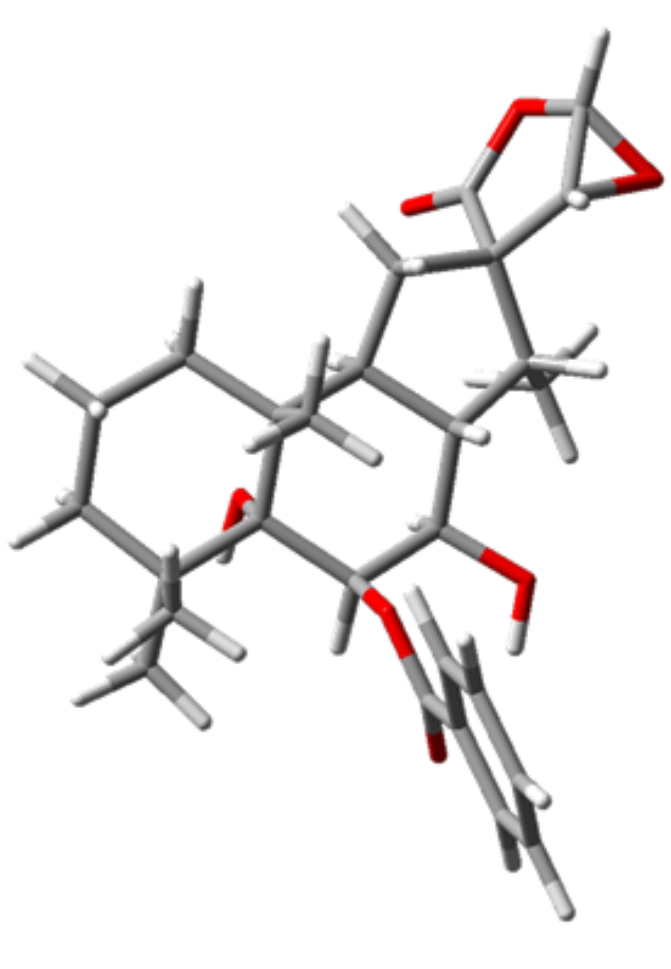

4a(a)

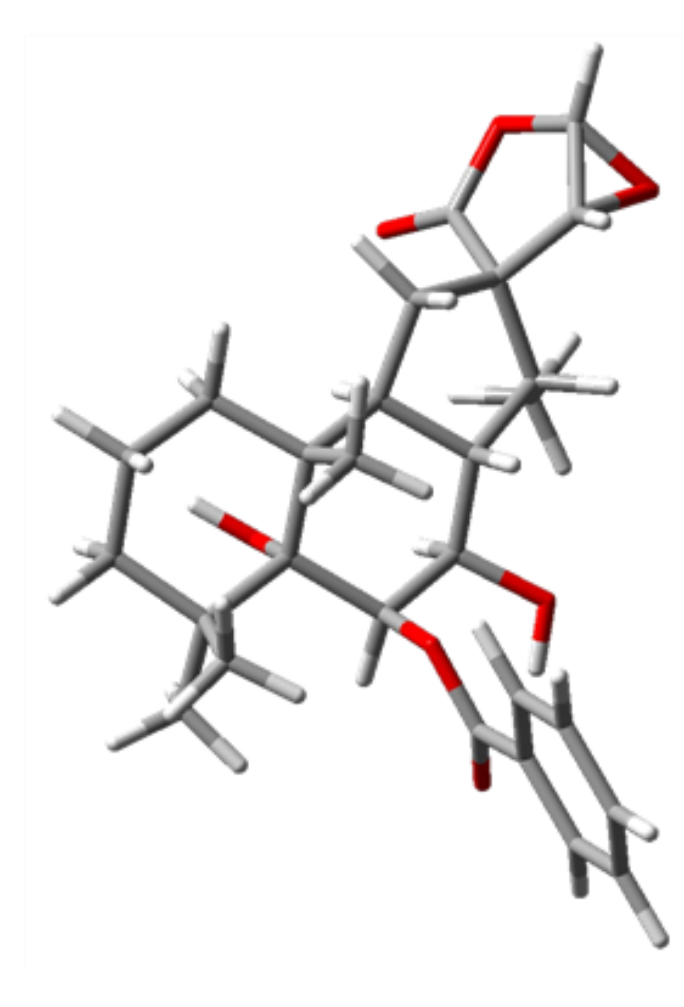

4a(b)

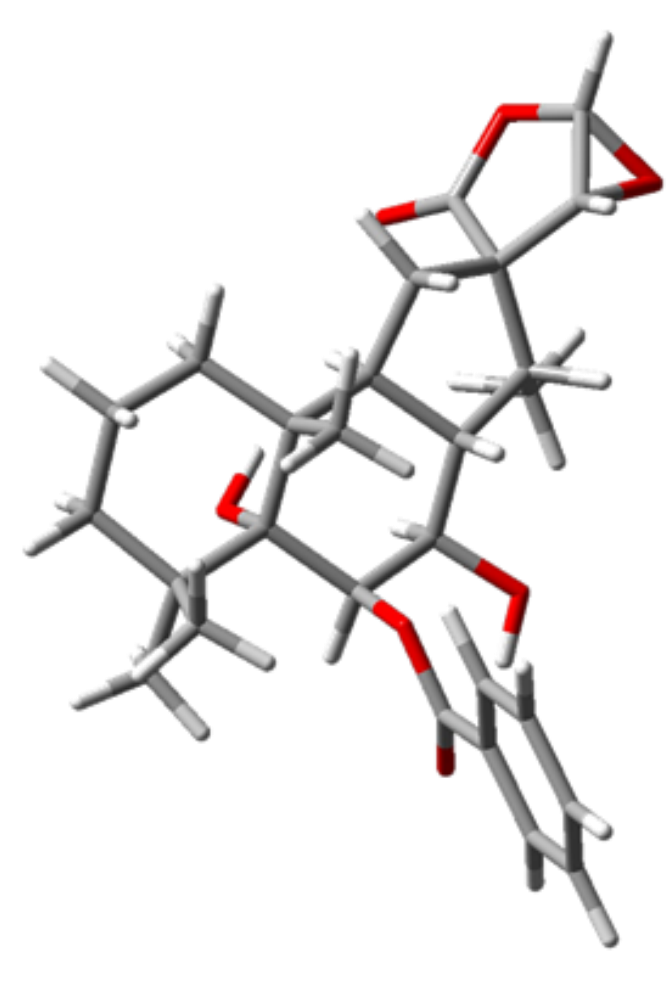

4a(c)

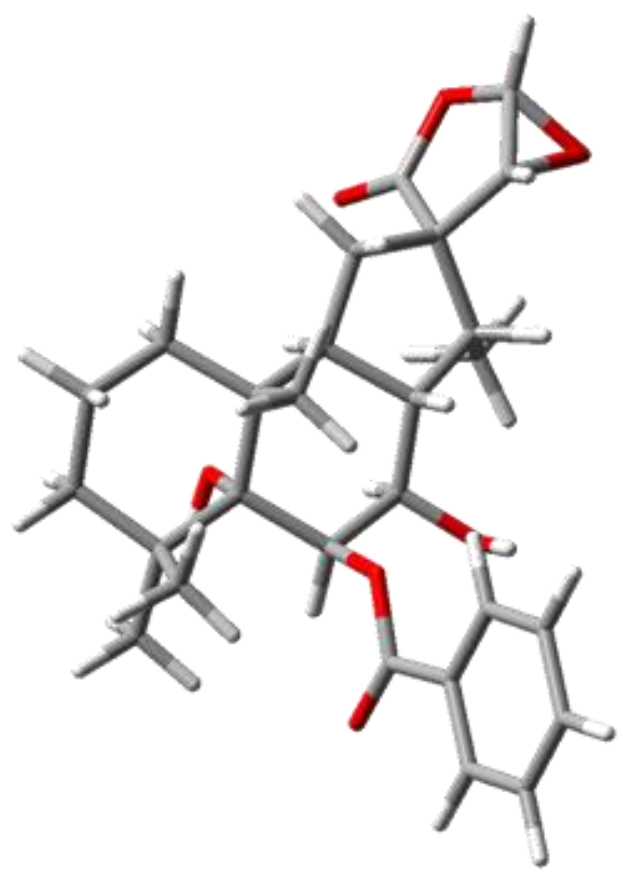

4a(d)

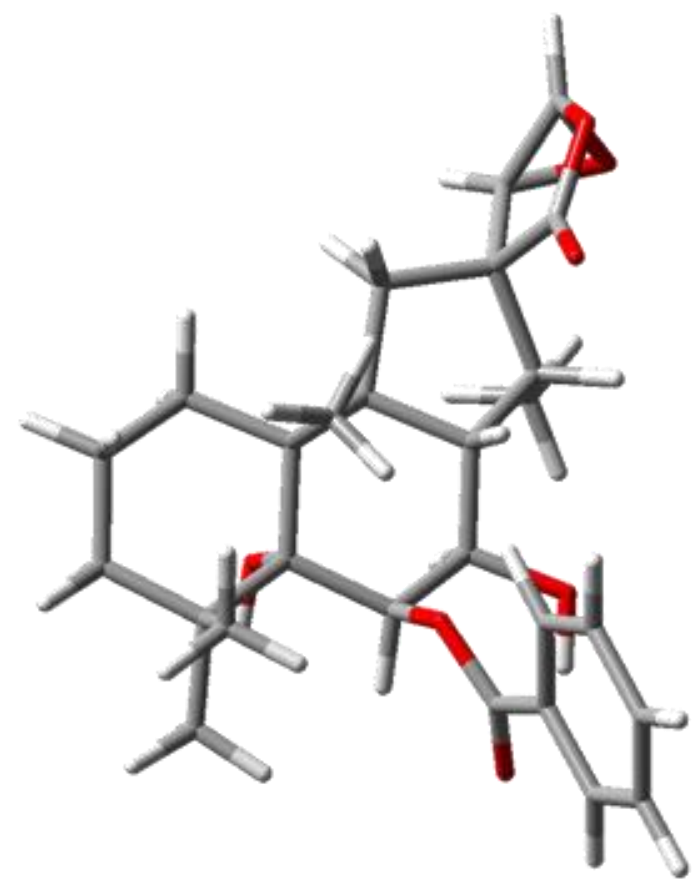

4b(a)

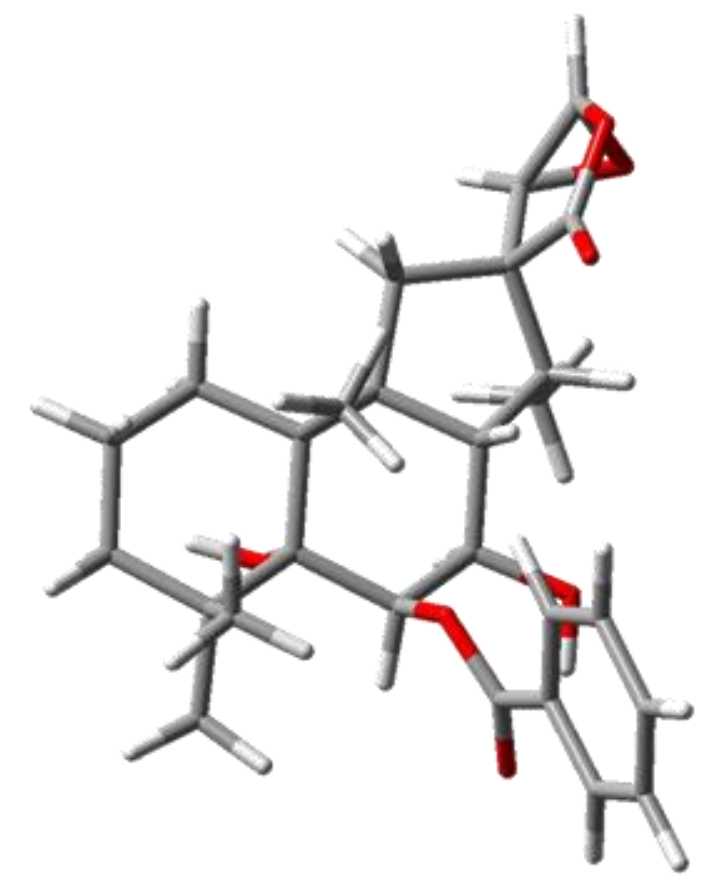

4b(b)

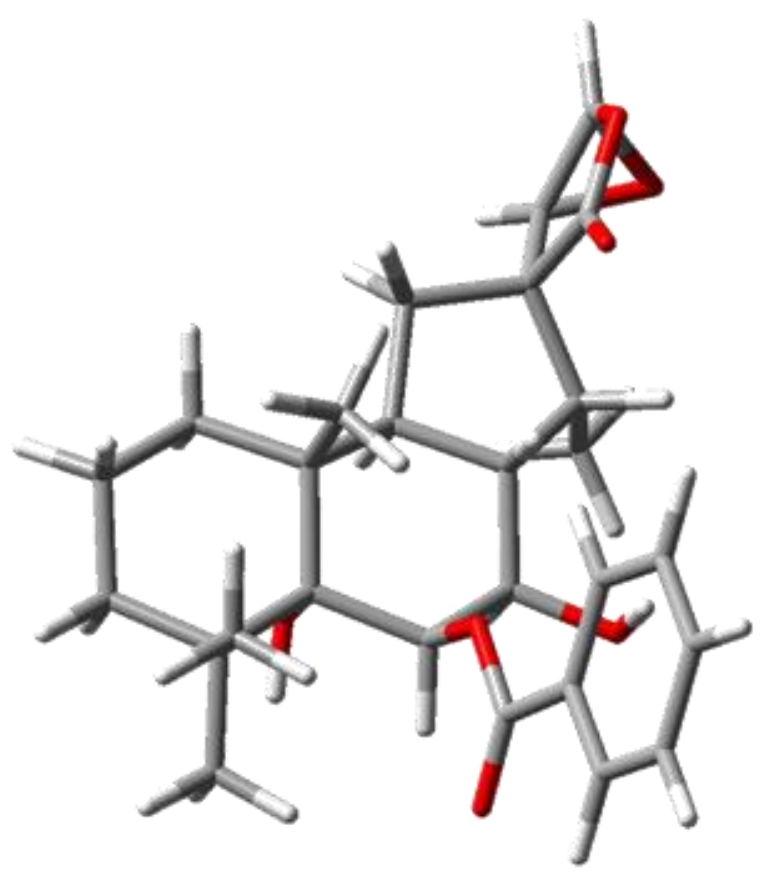

4b(c)

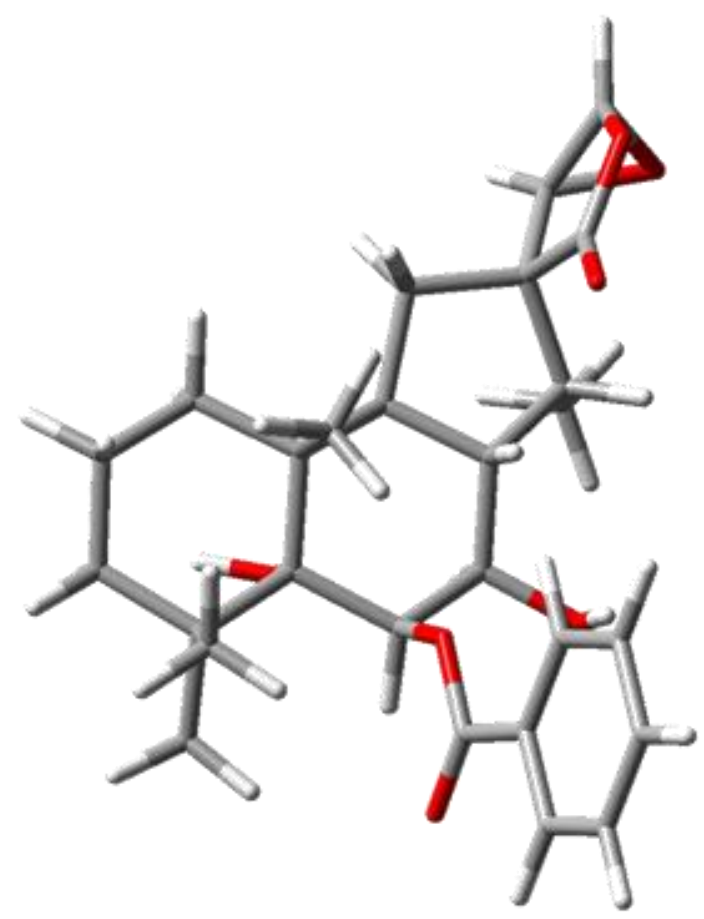

4b(d)

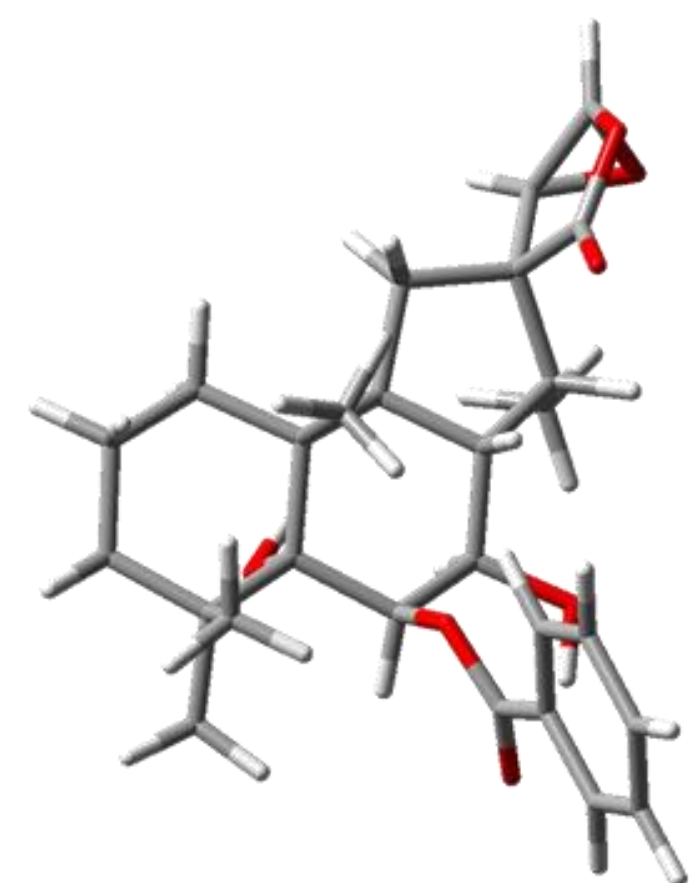

4b(e)

Figure S68. Calculated conformers of **1a**, **1b**, **2a**, **2b**, **3a**, **3b**, **4a**, and **4b** at the DFT B3LYP DGDZVP basis set. The Gibbs energies of conformers **1a**-**3b** are in kcal/mol as follows: **1a** *G* = −773965.6276, **1b** *G* = −773966.7483, **2a** *G* = −678173.31, **2b** *G* = −678174.3749, **3a** *G* = −677436.9779, **3b** *G* = −677437.9198. Thermochemical analysis of conformers **4a**(a)-**4a**(d) and **4b**(a)-**4b**(e) is given in Table S1.

Table S1. Thermochemical analysis of (+)-(5*R*,6*R*,7*R*,8*S*,9*S*,10*R*,13*S*,14*R*,15*R*,16*S*)-6-benzoyloxy-15,16-epoxy-5,7-dihydroxy-13-spirocassan-12,16-olide (**4a**), and (−)-(5*R*,6*R*,7*R*,8*S*,9*S*,10*R*,13*R*,14*R*,15*S*,16*R*)-6-benzoyloxy-15,16-epoxy-5,7-dihydroxy-13-spirocassan-12,16-olide (**4b**)

Conformer	ΔE_{MMFF}[a]	$\%_{MMFF}$[b]	ΔE_{DFT}[c]	$\%_{DFT}$[d]	ΔG_{OPT}[e]	$\%_{OPT}$[f]
**4a**(a)	1.31	10.0	0.00	50.0	0.00	56.2
**4a**(b)	0.00	89.8	0.26	32.3	0.32	32.8
**4a**(c)	3.76	0.2	0.64	17.1	1.38	5.5
**4a**(d)	5.64	0.0	2.61	0.6	1.38	5.5
**4b**(a)	0.40	33.6	0.00	62.9	0.00	40.3
**4b**(b)	0.00	65.7	0.86	14.9	0.25	26.6
**4b**(c)	2.79	0.6	0.75	17.9	0.29	24.8
**4b**(d)	3.74	0.1	2.51	0.9	1.09	6.5
**4b**(e)	4.29	0.0	1.73	3.5	1.85	1.8

[a]Molecular mechanics energies relative to **4a** E_{MMFF} = 156.76 kcal/mol, and to **4b**, E_{MMFF} = 156.86 kcal/mol. [b]Population in % calculated from the MMFF energies according to $\Delta E_{MMFF} \approx -RT \ln K$. [c]Single-point B3LYP/6-31G(d) energies relative to **4a**, $E_{6\text{-}31G(d)} = -988904.53$ kcal/mol, and to **4b** $E_{6\text{-}31G(d)} = -988906.17$. [d]Population in % calculated from B3LYP/6-31G(d) energies according to $\Delta E_{6\text{-}31G(d)} \approx -RT \ln K$. [e]Gibbs free energies relative to **4a** $G_{DGDZVP} = -988661.5346$ kcal/mol, and to **4b** $G_{DGDZVP} = -988662.4408$. [f]Population in % calculated from Gibbs free energies according to $\Delta G_{DGDZVP} \approx -RT \ln K$.

Table S2. Cytotoxicity IC_{50} in µg/mL of the vouacapane derivatives (**2**-**4**) and epoxyspirolactones (**1a**-**4a** and **1b**-**4b**) in human cell lines[a,b]

Compound	HeLa	MDA-MB-231	CCD-18Co
**2**	11.6 [11.1-16.6]	–[c]	–[c]
**3**	10.5 [10.2-13.6]	5.8 [5.5-8.3]	11.3 [11.0-14.4]
**4**	–[c]	–[c]	20.90 [20.3-27.8]
**1a**	12.8 [12.7-13.5]	18.5 [18.1-22.9]	–[c]
**1b**	5.2 [5.0-7.0]	4.2 [4.0-6.0]	–[c]
**2a**	5.6 [5.5-6.2]	2.9 [2.8-3.5]	10.3 [10.1-18.4]
**2b**	–[c]	4.8 [4.6-6.6]	–[c]
**3a**	–[c]	–[c]	8.2 [7.2-19.5]
**3b**	–[c]	2.9 [2.8-3.52]	11.4 [11.0-15.1]
**4a**	4.3 [3.5-13.1]	10.4 [9.2-24.2]	18.7 [17.5-32.5]
**4b**	–[c]	20.2 [19.2-32.1]	13.1 [12.6-18.7]

[a]Inhibitory concentrations IC_{50} in µg/mL [95% confidence intervals] were determined after 24 h of treatment. Nonlinear regression (curve fit), log (inhibitor/ inductor) vs response (variable slope), R^2 = 0.93–0.98 were calculated with GraphPad Prism 10.0. Methotrexate (100 µM) was used as the positive control showing for HeLa 25.2±1.3, for MDA-MB-231 23.5±1.4, and for CCD-18Co 30.8±0.9 µM.

[b]Confidence intervals were calculated with [AVG−0.52×SD — AVG+6.29×SD]

[c]Non-active (values above 50 µM).
