# Supplementary material for: Photooxidative Molecular Rearrangement of Vouacapane Diterpenes Generates Proapoptotic Spirocaesalmins
Source: ACS Omega. 2026 Aug 13;11(33):49592–606. doi: 10.1021/acsomega.6c03239 (PMC13520548; doi:10.1021/acsomega.6c03239)
Supplement: Supplementary file 2 [file ao6c03239_si_004.pdf]

′ Photooxidative molecular rearrangement of vouacapane diterpenes generates proapoptotic spirocaesalmins ′

Laura Hernández-Padilla,[a] Leonardo R. Alvarez-García,[a] Eva E. Soto-Guzmán,[a] Gabriela Rodríguez-García,[a] Edna M. Silva-García,[b] Lorena Martínez-Alcántar,[a] Jesús Campos-García,[a] Christine Thomassigny,[c] Rosa E. del Río,[a] Armando Talavera-Alemán,[a]* Mario A. Gómez-Hurtado,[a]* and Carlos M. Cerda-García-Rojas[d]*

[a]Instituto de Investigaciones Químico Biológicas, Universidad Michoacana de San Nicolas de Hidalgo, Ciudad Universitaria, Morelia, Michoacán, C.P. 58030 , México, [b]Universidad La Salle, Av. Universidad 500, Cto. Erandeni I, Tarímbaro, Michoacán, C.P. 58880 , México, [c]Universite de Versailles St-Quentin-en-Yvelines, 45 Avenue des Etats-Unis, 78035 Versailles, France, and [d]Departamento de Química, Centro de Investigación y de Estudios Avanzados del Instituto Politécnico Nacional, Apartado Postal 14-740, 07360 Ciudad de México, México

Correspondence email: armando.talavera@umich.mx; mario.gomez@umich.mx; ccerda@cinvestav.mx

Abstract

Table 1

Experimental details

Crystal data	
Chemical formula	$C_{20}H_{30}O_4$
M_r	334.44
Crystal system, space group	Triclinic, *P*1
Temperature (K)	296
a, *b*, *c* (Å)	6.211 (3), 7.454 (3), 21.325 (9)
α, β, γ (°)	87.839 (9), 85.546 (9), 66.036 (8)
V (Å^3)	899.5 (7)
Z	2
Radiation type	Mo *K*α
μ (mm^{-1})	0.08
Crystal size (mm)	0.37 × 0.2 × 0.09
Data collection	
Diffractometer	Bruker D8 VENTURE
Absorption correction	Multi-scan<br>*SADABS2016*/2 - Bruker AXS area detector scaling and absorption correcti
T_{min}, T_{max}	0.296, 0.745
No. of measured, independent and observed [$I > 2\sigma(I)$] reflections	29434, 5106, 2846
R_{int}	0.202
θ_{max} (°)	23.4
$(\sin \theta/\lambda)_{max}$ (Å^{-1})	0.558
Refinement	
$R[F^2 > 2\sigma(F^2)]$, $wR(F^2)$, S	0.088, 0.221, 1.03
No. of reflections	5106
No. of parameters	442

No. of restraints	3
H-atom treatment	H-atom parameters constrained
$\Delta\rho_{max}$, $\Delta\rho_{min}$ (e Å^{-3})	0.20, −0.20
Absolute structure	Flack x determined using 987 quotients [(I+)-(I-)]/[(I+)+(I-)] (Parsons, Flack and Wagner, Acta Cryst. B69 (2013) 249-259).
Absolute structure parameter	−0.5 (10)

Computer programs: Bruker *APEX3* software, *SAINT* V8.42 integration software, *SORTAV* (Blessing, 1995), *SHELXT* 2018/2 (Sheldrick, 2018), *SHELXL2025*/1 (Sheldrick, 2025), *ORTEP* for Windows (Farrugia, 2012), *WinGX* publication routines (Farrugia, 2012).

Table 2

Hydrogen-bond geometry (Å, º)

D—H···*A*	*D*—H	H···*A*	*D*···*A*	*D*—H···*A*
O1*A*—H1*AB*···O4*A*^{i}	0.82	2.39	3.181 (15)	163
C7*A*—H7*AA*···O4*A*^{i}	0.97	2.45	3.312 (17)	148
C16—H16*A*···O2*A*^{ii}	0.98	2.47	3.391 (18)	156
C16*A*—H16···O2^{iii}	0.98	2.38	3.19 (2)	140
O1*A*—H1*AB*···O4*A*^{i}	0.82	2.39	3.181 (15)	163
C7*A*—H7*AA*···O4*A*^{i}	0.97	2.45	3.312 (17)	148
C16—H16*A*···O2*A*^{ii}	0.98	2.47	3.391 (18)	156
C16*A*—H16···O2^{iii}	0.98	2.38	3.19 (2)	140

Symmetry codes: (i) *x*, *y*−1, *z*; (ii) *x*, *y*+1, *z*; (iii) *x*−1, *y*, *z*.

Acknowledgements

Funding information

References

Figure 1

supporting information

′ Photooxidative molecular rearrangement of vouacapane diterpenes generates proapoptotic spirocaesalmins ′

Computing details

Data collection: Bruker *APEX3* software; cell refinement: *SAINT* V8.42 integration software; data reduction: *SORTAV* (Blessing, 1995); program(s) used to solve structure: *SHELXT* 2018/2 (Sheldrick, 2018); program(s) used to refine structure: *SHELXL2025*/1 (Sheldrick, 2025); molecular graphics: *ORTEP* for Windows (Farrugia, 2012); software used to prepare material for publication: *WinGX* publication routines (Farrugia, 2012).

(shelx)

Crystal data

$C_{20}H_{30}O_4$	$Z = 2$
$M_r = 334.44$	$F(000) = 364$
Triclinic, $P1$	$D_x = 1.235$ Mg m^{-3}
Hall symbol: P 1	Mo $K\alpha$ radiation, $\lambda = 0.71073$ Å
$a = 6.211$ (3) Å	Cell parameters from 3571 reflections
$b = 7.454$ (3) Å	$\theta = 2.9$–$19.1°$
$c = 21.325$ (9) Å	$\mu = 0.08$ mm^{-1}
$\alpha = 87.839$ (9)°	$T = 296$ K
$\beta = 85.546$ (9)°	Plate, colourless
$\gamma = 66.036$ (8)°	0.37 × 0.2 × 0.09 mm
$V = 899.5$ (7) Å^3	

Data collection

Bruker D8 VENTURE diffractometer	29434 measured reflections
Radiation source: microfocus sealed tube	5106 independent reflections
Multilayer mirror monochromator	2846 reflections with $I > 2\sigma(I)$
Absorption correction: multi-scan *SADABS2016*/2 - Bruker AXS area detector scaling and absorption correcti	$R_{int} = 0.202$
	$\theta_{max} = 23.4°$, $\theta_{min} = 2.9°$
	$h = -6 \rightarrow 6$
	$k = -8 \rightarrow 8$
$T_{min} = 0.296$, $T_{max} = 0.745$	$l = -23 \rightarrow 23$

Refinement

Refinement on F^2	$w = 1/[\sigma^2(F_o^2) + (0.0637P)^2 + 1.1733P]$ where $P = (F_o^2 + 2F_c^2)/3$
Least-squares matrix: full	$(\Delta/\sigma)_{max} < 0.001$
$R[F^2 > 2\sigma(F^2)] = 0.088$	$\Delta\rho_{max} = 0.20$ e Å^{-3}
$wR(F^2) = 0.221$	$\Delta\rho_{min} = -0.20$ e Å^{-3}
$S = 1.03$	Extinction correction: *SHELXL2025*/1 (Sheldrick 2025), $Fc^* = kFc[1+0.001xFc^2\lambda^3/\sin(2\theta)]^{-1/4}$
5106 reflections	Extinction coefficient: 0.18 (3)
442 parameters	Absolute structure: Flack x determined using 987 quotients [(I+)-(I-)]/[(I+)+(I-)] (Parsons, Flack and Wagner, Acta Cryst. B69 (2013) 249-259).
3 restraints	Absolute structure parameter: −0.5 (10)
0 constraints	
Hydrogen site location: inferred from neighbouring sites	
H-atom parameters constrained	

Special details

Geometry. All e.s.d.'s (except the e.s.d. in the dihedral angle between two l.s. planes) are estimated using the full covariance matrix. The cell e.s.d.'s are taken into account individually in the estimation of e.s.d.'s in distances, angles and torsion angles; correlations between e.s.d.'s in cell parameters are only used when they are defined by crystal symmetry. An approximate (isotropic) treatment of cell e.s.d.'s is used for estimating e.s.d.'s involving l.s. planes.

Fractional atomic coordinates and isotropic or equivalent isotropic displacement parameters (Å^2)

	x	y	z	U_{iso}*/U_{eq}
O1	1.0863 (16)	0.3866 (15)	0.7697 (5)	0.109 (3)
H1	1.076981	0.476096	0.792448	0.164*
O1A	0.262 (2)	−0.0976 (16)	0.2251 (5)	0.131 (4)
H1AB	0.284409	−0.172845	0.255284	0.196*
C1	0.378 (2)	0.8425 (19)	0.8414 (6)	0.089 (4)
H1AA	0.252024	0.799175	0.837467	0.107*
H1BB	0.320912	0.979221	0.828496	0.107*
C1A	0.141 (2)	0.5117 (16)	0.1574 (6)	0.073 (4)
H1CC	0.00059	0.626025	0.170639	0.087*
H1DD	0.276075	0.542418	0.162176	0.087*
O2	0.463 (2)	0.9417 (18)	0.5172 (5)	0.116 (4)
O2A	0.0479 (18)	0.5529 (15)	0.4848 (4)	0.111 (3)
C15	0.470 (2)	0.9784 (18)	0.5832 (8)	0.087 (4)
H15	0.57614	1.035744	0.596049	0.104*
C15A	−0.048 (3)	0.590 (2)	0.4231 (6)	0.089 (4)
H13	−0.207766	0.60056	0.418073	0.107*
C2	0.436 (3)	0.826 (2)	0.9109 (7)	0.099 (5)
H2A	0.559093	0.871845	0.915961	0.119*
H2B	0.296858	0.905881	0.936911	0.119*
C2A	0.131 (3)	0.4788 (19)	0.0894 (7)	0.090 (4)
H2AA	0.136676	0.589618	0.06507	0.107*
H2AB	−0.016927	0.46959	0.082686	0.107*
O3	0.0854 (17)	1.0454 (16)	0.5659 (5)	0.106 (3)
O3A	0.184 (2)	0.7615 (16)	0.4279 (6)	0.107 (3)
C3	0.519 (2)	0.6113 (18)	0.9307 (6)	0.083 (4)
H3A	0.390311	0.57048	0.927189	0.099*
H3B	0.553246	0.599769	0.974626	0.099*
C3A	0.336 (2)	0.2914 (16)	0.0666 (6)	0.076 (4)
H3AA	0.31444	0.26953	0.023461	0.091*
H3AB	0.48031	0.312498	0.066474	0.091*
O4	0.019 (2)	0.8439 (18)	0.6328 (7)	0.151 (5)
O4A	0.4438 (18)	0.6343 (16)	0.3457 (6)	0.109 (3)
C13	0.156 (2)	0.899 (2)	0.6121 (8)	0.092 (4)
C14	0.575 (3)	0.6281 (17)	0.6213 (6)	0.082 (4)
H14	0.699571	0.613555	0.588099	0.098*
C14A	0.280 (3)	0.636 (2)	0.3786 (8)	0.092 (5)
C4	0.738 (2)	0.4701 (18)	0.8920 (6)	0.073 (3)
C4A	0.370 (2)	0.1095 (19)	0.1034 (6)	0.072 (3)
C12	0.397 (2)	0.8554 (17)	0.6272 (6)	0.066 (3)
C12A	0.139 (2)	0.5116 (17)	0.3717 (6)	0.069 (3)
C5	0.687 (2)	0.5066 (14)	0.8202 (6)	0.062 (3)
H5	0.552339	0.471048	0.817866	0.075*
C5A	0.3655 (19)	0.1541 (15)	0.1734 (6)	0.065 (3)

H5A	0.49965	0.191475	0.173369	0.078*
C11A	0.043 (2)	0.5525 (17)	0.3053 (6)	0.075 (4)
H11A	−0.115337	0.556267	0.306519	0.089*
H11B	0.03956	0.676458	0.288703	0.089*
C6	0.869 (2)	0.364 (2)	0.7752 (7)	0.083 (4)
H6	0.902014	0.231546	0.791282	0.099*
C6A	0.440 (2)	−0.0207 (18)	0.2198 (6)	0.076 (4)
H6A	0.587419	−0.123375	0.20219	0.092*
C7	0.784 (2)	0.3782 (15)	0.7095 (6)	0.079 (4)
H7A	0.915254	0.299342	0.680925	0.095*
H7B	0.66469	0.325336	0.710156	0.095*
C7A	0.485 (3)	0.0341 (17)	0.2837 (6)	0.093 (4)
H7AA	0.51664	−0.076645	0.312317	0.111*
H7AB	0.622848	0.064953	0.279971	0.111*
C9	0.492 (2)	0.7194 (15)	0.7331 (5)	0.065 (3)
H9	0.366619	0.671563	0.739135	0.078*
C9A	0.218 (2)	0.3790 (15)	0.2647 (6)	0.068 (3)
H9A	0.362679	0.401999	0.257854	0.082*
C10	0.5843 (19)	0.7246 (16)	0.7984 (6)	0.063 (3)
C10A	0.1570 (19)	0.3460 (15)	0.2010 (5)	0.056 (3)
C8	0.684 (2)	0.5840 (16)	0.6861 (6)	0.066 (3)
H8	0.812612	0.628486	0.683362	0.08*
C8A	0.276 (2)	0.2078 (16)	0.3099 (6)	0.067 (3)
H8A	0.140028	0.171517	0.313696	0.08*
C20	0.758 (3)	0.824 (2)	0.7945 (7)	0.107 (5)
H20A	0.673088	0.962577	0.800656	0.161*
H20B	0.842845	0.800473	0.753924	0.161*
H20C	0.868198	0.77059	0.826569	0.161*
C11	0.398 (3)	0.9118 (18)	0.6958 (7)	0.115 (6)
H21A	0.499103	0.981569	0.698628	0.137*
H21B	0.239693	0.995399	0.711985	0.137*
C20A	−0.088 (2)	0.331 (2)	0.2073 (7)	0.095 (5)
H20D	−0.193923	0.429235	0.235902	0.142*
H20E	−0.067763	0.203127	0.223138	0.142*
H20F	−0.152014	0.350954	0.166864	0.142*
C18	0.766 (3)	0.268 (2)	0.9121 (7)	0.123 (6)
H18A	0.905855	0.172707	0.890954	0.185*
H18B	0.6308	0.246524	0.901448	0.185*
H18C	0.779277	0.253863	0.956725	0.185*
C18A	0.599 (2)	−0.0543 (19)	0.0821 (7)	0.093 (4)
H18D	0.599352	−0.177436	0.096966	0.14*
H18E	0.727643	−0.035028	0.098659	0.14*
H18F	0.617566	−0.054933	0.036956	0.14*
C16	0.271 (3)	1.094 (2)	0.5477 (8)	0.106 (5)
H16A	0.241284	1.231061	0.537175	0.127*
C16A	−0.011 (3)	0.742 (3)	0.4545 (7)	0.100 (5)
H16	−0.146557	0.857393	0.470986	0.12*
C17	0.457 (3)	0.4914 (19)	0.6085 (9)	0.123 (6)
H17A	0.399486	0.517403	0.567168	0.185*
H17B	0.327716	0.512906	0.639144	0.185*
H17C	0.569709	0.357624	0.611023	0.185*
C17A	0.543 (3)	0.2332 (19)	0.3944 (8)	0.103 (5)

H17D	0.539125	0.275762	0.436606	0.154*
H17E	0.620464	0.294948	0.36615	0.154*
H17F	0.62769	0.093257	0.392222	0.154*
C19	0.956 (3)	0.502 (3)	0.9108 (7)	0.123 (6)
H19A	0.929381	0.63755	0.904465	0.184*
H19B	1.09328	0.421156	0.885236	0.184*
H19C	0.980612	0.467582	0.954259	0.184*
C19A	0.175 (3)	0.042 (2)	0.0891 (7)	0.098 (4)
H19D	0.025204	0.152621	0.090486	0.147*
H19E	0.169314	−0.053764	0.119912	0.147*
H19F	0.209026	−0.015561	0.048021	0.147*
C28	0.294 (2)	0.2893 (17)	0.3762 (6)	0.076 (4)
H28	0.215894	0.23382	0.408084	0.091*

Atomic displacement parameters (Å²)

	U^{11}	U^{22}	U^{33}	U^{12}	U^{13}	U^{23}
O1	0.069 (6)	0.121 (8)	0.108 (7)	−0.008 (6)	−0.012 (5)	0.009 (6)
O1A	0.213 (13)	0.120 (8)	0.117 (8)	−0.128 (9)	−0.018 (8)	0.014 (6)
C1	0.092 (10)	0.068 (9)	0.076 (9)	0.004 (8)	−0.019 (8)	−0.006 (7)
C1A	0.080 (9)	0.038 (7)	0.083 (9)	−0.004 (6)	−0.022 (7)	0.009 (6)
O2	0.109 (8)	0.130 (9)	0.081 (7)	−0.023 (7)	−0.004 (6)	0.020 (6)
O2A	0.115 (8)	0.112 (9)	0.072 (7)	−0.010 (6)	−0.023 (6)	0.007 (6)
C15	0.059 (8)	0.056 (8)	0.147 (15)	−0.022 (7)	−0.027 (9)	0.007 (8)
C15A	0.094 (10)	0.097 (10)	0.060 (9)	−0.022 (8)	−0.012 (8)	0.011 (8)
C2	0.086 (10)	0.089 (11)	0.100 (12)	−0.010 (9)	−0.017 (9)	−0.014 (9)
C2A	0.106 (11)	0.068 (9)	0.089 (11)	−0.027 (8)	−0.024 (8)	0.016 (7)
O3	0.068 (7)	0.107 (8)	0.122 (8)	−0.009 (6)	−0.042 (6)	0.011 (7)
O3A	0.098 (8)	0.111 (8)	0.120 (9)	−0.049 (7)	−0.027 (7)	0.005 (7)
C3	0.097 (11)	0.073 (10)	0.076 (9)	−0.032 (8)	−0.010 (8)	0.005 (7)
C3A	0.085 (9)	0.055 (8)	0.081 (9)	−0.019 (7)	−0.027 (7)	0.005 (7)
O4	0.082 (8)	0.143 (10)	0.249 (16)	−0.070 (8)	0.025 (9)	−0.034 (10)
O4A	0.063 (6)	0.116 (8)	0.154 (10)	−0.044 (6)	−0.023 (7)	0.039 (7)
C13	0.048 (9)	0.095 (11)	0.128 (13)	−0.025 (8)	0.006 (9)	−0.010 (10)
C14	0.114 (11)	0.050 (8)	0.068 (8)	−0.017 (7)	−0.022 (8)	0.001 (6)
C14A	0.060 (10)	0.090 (11)	0.100 (12)	−0.001 (9)	−0.023 (9)	0.016 (10)
C4	0.053 (8)	0.072 (9)	0.087 (9)	−0.018 (7)	−0.020 (7)	0.013 (7)
C4A	0.061 (7)	0.077 (9)	0.077 (9)	−0.024 (7)	−0.019 (6)	−0.004 (7)
C12	0.058 (8)	0.065 (8)	0.064 (8)	−0.011 (6)	−0.017 (6)	0.010 (6)
C12A	0.065 (8)	0.073 (9)	0.083 (9)	−0.042 (7)	−0.011 (7)	0.004 (7)
C5	0.056 (7)	0.033 (6)	0.082 (8)	−0.002 (6)	−0.012 (6)	0.007 (5)
C5A	0.052 (7)	0.039 (6)	0.099 (9)	−0.013 (6)	−0.013 (6)	0.008 (6)
C11A	0.076 (8)	0.062 (8)	0.076 (9)	−0.015 (7)	−0.031 (7)	0.013 (7)
C6	0.049 (8)	0.080 (9)	0.103 (11)	−0.008 (7)	−0.014 (8)	0.011 (8)
C6A	0.076 (9)	0.061 (8)	0.089 (10)	−0.022 (7)	−0.017 (7)	0.003 (7)
C7	0.111 (10)	0.038 (7)	0.064 (8)	−0.004 (6)	−0.015 (7)	−0.002 (6)
C7A	0.110 (11)	0.043 (7)	0.090 (10)	0.004 (7)	−0.019 (8)	0.025 (7)
C9	0.070 (8)	0.043 (7)	0.065 (8)	−0.004 (6)	−0.014 (6)	0.006 (6)
C9A	0.058 (7)	0.051 (7)	0.075 (9)	−0.002 (6)	−0.003 (6)	0.022 (6)
C10	0.043 (6)	0.048 (7)	0.088 (9)	−0.007 (6)	−0.016 (6)	0.006 (6)
C10A	0.053 (7)	0.052 (7)	0.063 (7)	−0.017 (6)	−0.026 (6)	0.002 (5)
C8	0.055 (7)	0.050 (7)	0.086 (9)	−0.013 (6)	−0.010 (7)	0.005 (6)

C8A	0.056 (7)	0.064 (8)	0.086 (9)	−0.026 (6)	−0.021 (6)	0.007 (7)
C20	0.145 (14)	0.113 (12)	0.085 (10)	−0.075 (11)	−0.015 (9)	0.007 (8)
C11	0.136 (13)	0.044 (8)	0.108 (13)	0.022 (8)	−0.033 (10)	0.024 (8)
C20A	0.045 (7)	0.148 (13)	0.113 (11)	−0.059 (8)	−0.006 (7)	−0.033 (9)
C18	0.151 (15)	0.098 (12)	0.084 (11)	−0.012 (10)	−0.016 (10)	0.017 (9)
C18A	0.086 (10)	0.063 (8)	0.109 (11)	−0.007 (7)	−0.003 (8)	−0.011 (7)
C16	0.096 (12)	0.087 (11)	0.107 (12)	−0.009 (10)	−0.023 (11)	0.039 (9)
C16A	0.090 (12)	0.104 (13)	0.080 (10)	−0.011 (10)	−0.017 (9)	−0.008 (10)
C17	0.161 (15)	0.051 (8)	0.166 (16)	−0.039 (9)	−0.087 (13)	0.001 (9)
C17A	0.098 (11)	0.065 (9)	0.129 (12)	−0.008 (8)	−0.054 (10)	0.004 (8)
C19	0.083 (11)	0.182 (17)	0.092 (11)	−0.040 (11)	−0.033 (9)	0.019 (11)
C19A	0.102 (11)	0.095 (10)	0.108 (11)	−0.049 (9)	−0.019 (9)	0.003 (8)
C28	0.081 (9)	0.054 (8)	0.085 (9)	−0.021 (7)	−0.009 (7)	0.021 (7)

Geometric parameters (Å, º)

O1—C6	1.418 (15)	C5A—C6A	1.540 (16)
O1—H1	0.82	C5A—C10A	1.584 (15)
O1A—C6A	1.434 (15)	C5A—H5A	0.98
O1A—H1AB	0.82	C11A—C9A	1.549 (16)
C1—C10	1.490 (16)	C11A—H11A	0.97
C1—C2	1.541 (18)	C11A—H11B	0.97
C1—H1AA	0.97	C6—C7	1.522 (17)
C1—H1BB	0.97	C6—H6	0.98
C1A—C2A	1.492 (18)	C6A—C7A	1.515 (17)
C1A—C10A	1.492 (15)	C6A—H6A	0.98
C1A—H1CC	0.97	C7—C8	1.485 (15)
C1A—H1DD	0.97	C7—H7A	0.97
O2—C16	1.404 (19)	C7—H7B	0.97
O2—C15	1.451 (17)	C7A—C8A	1.501 (16)
O2A—C16A	1.446 (19)	C7A—H7AA	0.97
O2A—C15A	1.456 (16)	C7A—H7AB	0.97
C15—C16	1.440 (19)	C9—C11	1.529 (16)
C15—C12	1.461 (17)	C9—C8	1.534 (15)
C15—H15	0.98	C9—C10	1.552 (15)
C15A—C16A	1.44 (2)	C9—H9	0.98
C15A—C12A	1.478 (18)	C9A—C10A	1.493 (16)
C15A—H13	0.98	C9A—C8A	1.508 (15)
C2—C3	1.522 (18)	C9A—H9A	0.98
C2—H2A	0.97	C10—C20	1.535 (18)
C2—H2B	0.97	C10A—C20A	1.565 (15)
C2A—C3A	1.522 (17)	C8—H8	0.98
C2A—H2AA	0.97	C8A—C28	1.590 (16)
C2A—H2AB	0.97	C8A—H8A	0.98
O3—C16	1.368 (19)	C20—H20A	0.96
O3—C13	1.396 (18)	C20—H20B	0.96
O3A—C16A	1.358 (19)	C20—H20C	0.96
O3A—C14A	1.364 (17)	C11—H21A	0.97
C3—C4	1.542 (17)	C11—H21B	0.97
C3—H3A	0.97	C20A—H20D	0.96
C3—H3B	0.97	C20A—H20E	0.96
C3A—C4A	1.488 (17)	C20A—H20F	0.96

C3A—H3AA	0.97	C18—H18A	0.96
C3A—H3AB	0.97	C18—H18B	0.96
O4—C13	1.145 (15)	C18—H18C	0.96
O4A—C14A	1.184 (18)	C18A—H18D	0.96
C13—C12	1.454 (18)	C18A—H18E	0.96
C14—C17	1.520 (18)	C18A—H18F	0.96
C14—C8	1.550 (17)	C16—H16A	0.98
C14—C12	1.608 (16)	C16A—H16	0.98
C14—H14	0.98	C17—H17A	0.96
C14A—C12A	1.53 (2)	C17—H17B	0.96
C4—C18	1.498 (19)	C17—H17C	0.96
C4—C19	1.549 (19)	C17A—C28	1.508 (18)
C4—C5	1.579 (16)	C17A—H17D	0.96
C4A—C18A	1.503 (17)	C17A—H17E	0.96
C4A—C5A	1.538 (17)	C17A—H17F	0.96
C4A—C19A	1.541 (17)	C19—H19A	0.96
C12—C11	1.538 (19)	C19—H19B	0.96
C12A—C28	1.546 (16)	C19—H19C	0.96
C12A—C11A	1.550 (16)	C19A—H19D	0.96
C5—C6	1.503 (17)	C19A—H19E	0.96
C5—C10	1.553 (14)	C19A—H19F	0.96
C5—H5	0.98	C28—H28	0.98

C6—O1—H1	109.5	C8—C7—C6	111.7 (10)
C6A—O1A—H1AB	109.5	C8—C7—H7A	109.3
C10—C1—C2	113.2 (11)	C6—C7—H7A	109.3
C10—C1—H1AA	108.9	C8—C7—H7B	109.3
C2—C1—H1AA	108.9	C6—C7—H7B	109.3
C10—C1—H1BB	108.9	H7A—C7—H7B	107.9
C2—C1—H1BB	108.9	C8A—C7A—C6A	110.8 (11)
H1AA—C1—H1BB	107.7	C8A—C7A—H7AA	109.5
C2A—C1A—C10A	116.4 (10)	C6A—C7A—H7AA	109.5
C2A—C1A—H1CC	108.2	C8A—C7A—H7AB	109.5
C10A—C1A—H1CC	108.2	C6A—C7A—H7AB	109.5
C2A—C1A—H1DD	108.2	H7AA—C7A—H7AB	108.1
C10A—C1A—H1DD	108.2	C11—C9—C8	101.4 (10)
H1CC—C1A—H1DD	107.3	C11—C9—C10	117.3 (10)
C16—O2—C15	60.6 (9)	C8—C9—C10	113.3 (9)
C16A—O2A—C15A	59.5 (9)	C11—C9—H9	108.1
C16—C15—O2	58.1 (10)	C8—C9—H9	108.1
C16—C15—C12	108.0 (12)	C10—C9—H9	108.1
O2—C15—C12	115.1 (11)	C10A—C9A—C8A	115.8 (10)
C16—C15—H15	119.9	C10A—C9A—C11A	120.0 (9)
O2—C15—H15	119.9	C8A—C9A—C11A	102.5 (10)
C12—C15—H15	119.9	C10A—C9A—H9A	105.8
C16A—C15A—O2A	59.9 (9)	C8A—C9A—H9A	105.8
C16A—C15A—C12A	108.5 (14)	C11A—C9A—H9A	105.8
O2A—C15A—C12A	112.3 (12)	C1—C10—C20	107.9 (11)
C16A—C15A—H13	120.4	C1—C10—C9	107.9 (9)
O2A—C15A—H13	120.4	C20—C10—C9	111.5 (11)
C12A—C15A—H13	120.4	C1—C10—C5	109.9 (10)
C3—C2—C1	108.0 (11)	C20—C10—C5	115.3 (10)

C3—C2—H2A	110.1	C9—C10—C5	104.1 (9)
C1—C2—H2A	110.1	C1A—C10A—C9A	111.1 (9)
C3—C2—H2B	110.1	C1A—C10A—C20A	108.7 (10)
C1—C2—H2B	110.1	C9A—C10A—C20A	108.8 (10)
H2A—C2—H2B	108.4	C1A—C10A—C5A	107.5 (9)
C1A—C2A—C3A	111.1 (11)	C9A—C10A—C5A	107.5 (9)
C1A—C2A—H2AA	109.4	C20A—C10A—C5A	113.4 (9)
C3A—C2A—H2AA	109.4	C7—C8—C9	111.3 (10)
C1A—C2A—H2AB	109.4	C7—C8—C14	119.0 (10)
C3A—C2A—H2AB	109.4	C9—C8—C14	106.0 (9)
H2AA—C2A—H2AB	108	C7—C8—H8	106.6
C16—O3—C13	108.7 (11)	C9—C8—H8	106.6
C16A—O3A—C14A	110.4 (13)	C14—C8—H8	106.6
C2—C3—C4	114.5 (11)	C7A—C8A—C9A	110.1 (10)
C2—C3—H3A	108.6	C7A—C8A—C28	117.7 (10)
C4—C3—H3A	108.6	C9A—C8A—C28	105.9 (9)
C2—C3—H3B	108.6	C7A—C8A—H8A	107.6
C4—C3—H3B	108.6	C9A—C8A—H8A	107.6
H3A—C3—H3B	107.6	C28—C8A—H8A	107.6
C4A—C3A—C2A	116.6 (11)	C10—C20—H20A	109.5
C4A—C3A—H3AA	108.2	C10—C20—H20B	109.5
C2A—C3A—H3AA	108.2	H20A—C20—H20B	109.5
C4A—C3A—H3AB	108.2	C10—C20—H20C	109.5
C2A—C3A—H3AB	108.2	H20A—C20—H20C	109.5
H3AA—C3A—H3AB	107.3	H20B—C20—H20C	109.5
O4—C13—O3	117.2 (15)	C9—C11—C12	106.3 (11)
O4—C13—C12	132.2 (18)	C9—C11—H21A	110.5
O3—C13—C12	110.5 (13)	C12—C11—H21A	110.5
C17—C14—C8	111.4 (11)	C9—C11—H21B	110.5
C17—C14—C12	114.1 (12)	C12—C11—H21B	110.5
C8—C14—C12	102.6 (9)	H21A—C11—H21B	108.7
C17—C14—H14	109.5	C10A—C20A—H20D	109.5
C8—C14—H14	109.5	C10A—C20A—H20E	109.5
C12—C14—H14	109.5	H20D—C20A—H20E	109.5
O4A—C14A—O3A	122.8 (17)	C10A—C20A—H20F	109.5
O4A—C14A—C12A	127.1 (16)	H20D—C20A—H20F	109.5
O3A—C14A—C12A	110.0 (14)	H20E—C20A—H20F	109.5
C18—C4—C3	105.7 (11)	C4—C18—H18A	109.5
C18—C4—C19	108.5 (13)	C4—C18—H18B	109.5
C3—C4—C19	108.3 (11)	H18A—C18—H18B	109.5
C18—C4—C5	111.0 (10)	C4—C18—H18C	109.5
C3—C4—C5	107.7 (9)	H18A—C18—H18C	109.5
C19—C4—C5	115.2 (11)	H18B—C18—H18C	109.5
C3A—C4A—C18A	110.2 (12)	C4A—C18A—H18D	109.5
C3A—C4A—C5A	108.2 (10)	C4A—C18A—H18E	109.5
C18A—C4A—C5A	110.9 (10)	H18D—C18A—H18E	109.5
C3A—C4A—C19A	108.8 (11)	C4A—C18A—H18F	109.5
C18A—C4A—C19A	105.8 (11)	H18D—C18A—H18F	109.5
C5A—C4A—C19A	112.9 (11)	H18E—C18A—H18F	109.5
C13—C12—C15	103.5 (11)	O3—C16—O2	113.6 (14)
C13—C12—C11	109.9 (12)	O3—C16—C15	109.1 (12)
C15—C12—C11	111.3 (12)	O2—C16—C15	61.3 (10)

C13—C12—C14	114.9 (11)	O3—C16—H16A	119.7
C15—C12—C14	111.5 (10)	O2—C16—H16A	119.7
C11—C12—C14	105.8 (9)	C15—C16—H16A	119.7
C15A—C12A—C14A	101.1 (12)	O3A—C16A—C15A	109.9 (13)
C15A—C12A—C28	115.0 (10)	O3A—C16A—O2A	111.6 (12)
C14A—C12A—C28	112.2 (10)	C15A—C16A—O2A	60.6 (10)
C15A—C12A—C11A	113.4 (11)	O3A—C16A—H16	120.2
C14A—C12A—C11A	107.5 (11)	C15A—C16A—H16	120.2
C28—C12A—C11A	107.4 (10)	O2A—C16A—H16	120.2
C6—C5—C10	115.7 (10)	C14—C17—H17A	109.5
C6—C5—C4	115.5 (9)	C14—C17—H17B	109.5
C10—C5—C4	115.7 (9)	H17A—C17—H17B	109.5
C6—C5—H5	102.2	C14—C17—H17C	109.5
C10—C5—H5	102.2	H17A—C17—H17C	109.5
C4—C5—H5	102.2	H17B—C17—H17C	109.5
C4A—C5A—C6A	117.6 (10)	C28—C17A—H17D	109.5
C4A—C5A—C10A	117.7 (9)	C28—C17A—H17E	109.5
C6A—C5A—C10A	113.8 (10)	H17D—C17A—H17E	109.5
C4A—C5A—H5A	101.1	C28—C17A—H17F	109.5
C6A—C5A—H5A	101.1	H17D—C17A—H17F	109.5
C10A—C5A—H5A	101.1	H17E—C17A—H17F	109.5
C9A—C11A—C12A	104.7 (9)	C4—C19—H19A	109.5
C9A—C11A—H11A	110.8	C4—C19—H19B	109.5
C12A—C11A—H11A	110.8	H19A—C19—H19B	109.5
C9A—C11A—H11B	110.8	C4—C19—H19C	109.5
C12A—C11A—H11B	110.8	H19A—C19—H19C	109.5
H11A—C11A—H11B	108.9	H19B—C19—H19C	109.5
O1—C6—C5	113.4 (12)	C4A—C19A—H19D	109.5
O1—C6—C7	107.9 (12)	C4A—C19A—H19E	109.5
C5—C6—C7	112.6 (10)	H19D—C19A—H19E	109.5
O1—C6—H6	107.6	C4A—C19A—H19F	109.5
C5—C6—H6	107.6	H19D—C19A—H19F	109.5
C7—C6—H6	107.6	H19E—C19A—H19F	109.5
O1A—C6A—C7A	110.9 (12)	C17A—C28—C12A	116.3 (10)
O1A—C6A—C5A	108.8 (10)	C17A—C28—C8A	114.6 (11)
C7A—C6A—C5A	112.5 (10)	C12A—C28—C8A	103.6 (9)
O1A—C6A—H6A	108.1	C17A—C28—H28	107.3
C7A—C6A—H6A	108.1	C12A—C28—H28	107.3
C5A—C6A—H6A	108.1	C8A—C28—H28	107.3

C16—O2—C15—C12	96.3 (13)	C12A—C11A—C9A—C8A	37.7 (12)
C16A—O2A—C15A—C12A	99.2 (15)	C2—C1—C10—C20	−70.8 (15)
C10—C1—C2—C3	−60.2 (16)	C2—C1—C10—C9	168.6 (11)
C10A—C1A—C2A—C3A	−53.4 (16)	C2—C1—C10—C5	55.7 (15)
C1—C2—C3—C4	59.2 (15)	C11—C9—C10—C1	68.5 (15)
C1A—C2A—C3A—C4A	52.7 (16)	C8—C9—C10—C1	−173.9 (10)
C16—O3—C13—O4	179.0 (15)	C11—C9—C10—C20	−49.8 (15)
C16—O3—C13—C12	2.8 (17)	C8—C9—C10—C20	67.8 (13)
C16A—O3A—C14A—O4A	175.5 (13)	C11—C9—C10—C5	−174.8 (12)
C16A—O3A—C14A—C12A	−2.0 (15)	C8—C9—C10—C5	−57.1 (12)
C2—C3—C4—C18	−171.5 (12)	C6—C5—C10—C1	170.0 (11)
C2—C3—C4—C19	72.4 (15)	C4—C5—C10—C1	−50.3 (13)

C2—C3—C4—C5	−52.8 (14)	C6—C5—C10—C20	−67.8 (15)
C2A—C3A—C4A—C18A	−170.8 (11)	C4—C5—C10—C20	71.9 (14)
C2A—C3A—C4A—C5A	−49.3 (14)	C6—C5—C10—C9	54.6 (13)
C2A—C3A—C4A—C19A	73.6 (15)	C4—C5—C10—C9	−165.7 (10)
O4—C13—C12—C15	−179.2 (18)	C2A—C1A—C10A—C9A	167.6 (11)
O3—C13—C12—C15	−3.8 (15)	C2A—C1A—C10A—C20A	−72.8 (14)
O4—C13—C12—C11	−60 (2)	C2A—C1A—C10A—C5A	50.3 (14)
O3—C13—C12—C11	115.2 (13)	C8A—C9A—C10A—C1A	−171.1 (10)
O4—C13—C12—C14	59 (2)	C11A—C9A—C10A—C1A	65.1 (14)
O3—C13—C12—C14	−125.6 (12)	C8A—C9A—C10A—C20A	69.3 (13)
C16—C15—C12—C13	3.3 (16)	C11A—C9A—C10A—C20A	−54.5 (14)
O2—C15—C12—C13	−59.2 (13)	C8A—C9A—C10A—C5A	−53.8 (13)
C16—C15—C12—C11	−114.6 (14)	C11A—C9A—C10A—C5A	−177.6 (10)
O2—C15—C12—C11	−177.2 (11)	C4A—C5A—C10A—C1A	−48.9 (13)
C16—C15—C12—C14	127.4 (12)	C6A—C5A—C10A—C1A	167.6 (10)
O2—C15—C12—C14	64.9 (13)	C4A—C5A—C10A—C9A	−168.5 (10)
C17—C14—C12—C13	−7.1 (17)	C6A—C5A—C10A—C9A	48.0 (13)
C8—C14—C12—C13	−127.8 (13)	C4A—C5A—C10A—C20A	71.3 (14)
C17—C14—C12—C15	−124.5 (14)	C6A—C5A—C10A—C20A	−72.2 (13)
C8—C14—C12—C15	114.9 (12)	C6—C7—C8—C9	−53.0 (15)
C17—C14—C12—C11	114.3 (14)	C6—C7—C8—C14	−176.7 (11)
C8—C14—C12—C11	−6.3 (14)	C11—C9—C8—C7	−173.4 (12)
C16A—C15A—C12A—C14A	0.2 (13)	C10—C9—C8—C7	60.0 (14)
O2A—C15A—C12A—C14A	−64.1 (13)	C11—C9—C8—C14	−42.6 (13)
C16A—C15A—C12A—C28	121.2 (12)	C10—C9—C8—C14	−169.2 (10)
O2A—C15A—C12A—C28	57.0 (16)	C17—C14—C8—C7	34.0 (17)
C16A—C15A—C12A—C11A	−114.6 (13)	C12—C14—C8—C7	156.5 (11)
O2A—C15A—C12A—C11A	−178.9 (11)	C17—C14—C8—C9	−92.3 (13)
O4A—C14A—C12A—C15A	−176.3 (13)	C12—C14—C8—C9	30.2 (12)
O3A—C14A—C12A—C15A	1.1 (13)	C6A—C7A—C8A—C9A	−56.9 (14)
O4A—C14A—C12A—C28	60.7 (18)	C6A—C7A—C8A—C28	−178.4 (10)
O3A—C14A—C12A—C28	−122.0 (11)	C10A—C9A—C8A—C7A	60.3 (14)
O4A—C14A—C12A—C11A	−57.1 (17)	C11A—C9A—C8A—C7A	−167.2 (11)
O3A—C14A—C12A—C11A	120.2 (12)	C10A—C9A—C8A—C28	−171.4 (10)
C18—C4—C5—C6	−57.3 (15)	C11A—C9A—C8A—C28	−38.9 (12)
C3—C4—C5—C6	−172.5 (10)	C8—C9—C11—C12	38.0 (15)
C19—C4—C5—C6	66.5 (15)	C10—C9—C11—C12	161.9 (11)
C18—C4—C5—C10	162.9 (11)	C13—C12—C11—C9	105.0 (14)
C3—C4—C5—C10	47.7 (13)	C15—C12—C11—C9	−141.0 (12)
C19—C4—C5—C10	−73.2 (14)	C14—C12—C11—C9	−19.7 (16)
C3A—C4A—C5A—C6A	−169.4 (10)	C13—O3—C16—O2	65.7 (16)
C18A—C4A—C5A—C6A	−48.4 (15)	C13—O3—C16—C15	−0.6 (18)
C19A—C4A—C5A—C6A	70.2 (14)	C15—O2—C16—O3	−99.7 (13)
C3A—C4A—C5A—C10A	48.5 (14)	O2—C15—C16—O3	107.1 (16)
C18A—C4A—C5A—C10A	169.5 (11)	C12—C15—C16—O3	−1.8 (18)
C19A—C4A—C5A—C10A	−71.9 (14)	C12—C15—C16—O2	−108.9 (12)
C15A—C12A—C11A—C9A	−150.5 (11)	C14A—O3A—C16A—C15A	2.1 (16)
C14A—C12A—C11A—C9A	98.6 (12)	C14A—O3A—C16A—O2A	67.3 (15)
C28—C12A—C11A—C9A	−22.3 (13)	O2A—C15A—C16A—O3A	104.2 (13)
C10—C5—C6—O1	69.5 (14)	C12A—C15A—C16A—O3A	−1.4 (16)
C4—C5—C6—O1	−70.2 (14)	C12A—C15A—C16A—O2A	−105.5 (12)
C10—C5—C6—C7	−53.3 (15)	C15A—O2A—C16A—O3A	−101.4 (14)

C4—C5—C6—C7	167.0 (11)	C15A—C12A—C28—C17A	−107.3 (14)
C4A—C5A—C6A—O1A	−70.2 (14)	C14A—C12A—C28—C17A	7.5 (16)
C10A—C5A—C6A—O1A	73.3 (13)	C11A—C12A—C28—C17A	125.5 (12)
C4A—C5A—C6A—C7A	166.4 (11)	C15A—C12A—C28—C8A	126.1 (12)
C10A—C5A—C6A—C7A	−50.1 (14)	C14A—C12A—C28—C8A	−119.1 (12)
O1—C6—C7—C8	−75.9 (14)	C11A—C12A—C28—C8A	−1.2 (12)
C5—C6—C7—C8	50.0 (16)	C7A—C8A—C28—C17A	21.1 (15)
O1A—C6A—C7A—C8A	−68.5 (14)	C9A—C8A—C28—C17A	−102.5 (11)
C5A—C6A—C7A—C8A	53.7 (15)	C7A—C8A—C28—C12A	148.8 (11)
C12A—C11A—C9A—C10A	167.7 (10)	C9A—C8A—C28—C12A	25.2 (12)

Hydrogen-bond geometry (Å, º)

D—H···*A*	*D*—H	H···*A*	*D*···*A*	*D*—H···*A*
O1*A*—H1*AB*···O4*A*^{i}	0.82	2.39	3.181 (15)	163
C7*A*—H7*AA*···O4*A*^{i}	0.97	2.45	3.312 (17)	148
C16—H16*A*···O2*A*^{ii}	0.98	2.47	3.391 (18)	156
C16*A*—H16···O2^{iii}	0.98	2.38	3.19 (2)	140
O1*A*—H1*AB*···O4*A*^{i}	0.82	2.39	3.181 (15)	163
C7*A*—H7*AA*···O4*A*^{i}	0.97	2.45	3.312 (17)	148
C16—H16*A*···O2*A*^{ii}	0.98	2.47	3.391 (18)	156
C16*A*—H16···O2^{iii}	0.98	2.38	3.19 (2)	140

Symmetry codes: (i) *x*, *y*−1, *z*; (ii) *x*, *y*+1, *z*; (iii) *x*−1, *y*, *z*.
