# Supplementary material for: Photooxidative Molecular Rearrangement of Vouacapane Diterpenes Generates Proapoptotic Spirocaesalmins
Source: ACS Omega. 2026 Aug 13;11(33):49592–606. doi: 10.1021/acsomega.6c03239 (PMC13520548; doi:10.1021/acsomega.6c03239)
Supplement: Supplementary file 3 [file ao6c03239_si_005.pdf]

′ Photooxidative molecular rearrangement of vouacapane diterpenes generates proapoptotic spirocaesalmins ′

Laura Hernández-Padilla,[a] Leonardo R. Alvarez-García,[a] Eva E. Soto-Guzmán,[a] Gabriela Rodríguez-García,[a] Edna M. Silva-García,[b] Lorena Martínez-Alcántar,[a] Jesús Campos-García,[a] Christine Thomassigny,[c] Rosa E. del Río,[a] Armando Talavera-Alemán,[a]* Mario A. Gómez-Hurtado,[a]* and Carlos M. Cerda-García-Rojas[d]*

[a]Instituto de Investigaciones Químico Biológicas, Universidad Michoacana de San Nicolas de Hidalgo, Ciudad Universitaria, Morelia, Michoacán, C.P. 58030, México, [b]Universidad La Salle, Av. Universidad 500, Cto. Erandeni I, Tarímbaro, Michoacán, C.P. 58880, México, [c]Universite de Versailles St-Quentin-en-Yvelines, 45 Avenue des Etats-Unis, 78035 Versailles, France, and [d]Departamento de Química, Centro de Investigación y de Estudios Avanzados del Instituto Politécnico Nacional, Apartado Postal 14-740, 07360 Ciudad de México, México
Correspondence email: armando.talavera@umich.mx; mario.gomez@umich.mx; ccerda@cinvestav.mx

Abstract

Table 1
Experimental details

Crystal data	
Chemical formula	$C_{20}H_{28}O_4$
M_r	332.42
Crystal system, space group	Orthorhombic, $P2_12_12_1$
Temperature (K)	295
a, *b*, *c* (Å)	7.303 (3), 11.389 (5), 20.777 (9)
V (Å^3)	1728.2 (13)
Z	4
Radiation type	Mo *Kα*
μ (mm^{-1})	0.09
Crystal size (mm)	0.15 × 0.12 × 0.02
Data collection	
Diffractometer	Bruker D8 VENTURE
Absorption correction	Multi-scan *SADABS2016*/2 - Bruker AXS area detector scaling and absorption correcti
T_{min}, T_{max}	0.53, 0.745
No. of measured, independent and observed [$I > 2\sigma(I)$] reflections	33738, 1846, 1151
R_{int}	0.280
θ_{max} (°)	21.0
$(\sin \theta/\lambda)_{max}$ (Å^{-1})	0.504
Refinement	
$R[F^2 > 2\sigma(F^2)]$, $wR(F^2)$, S	0.066, 0.164, 1.08
No. of reflections	1846
No. of parameters	223
No. of restraints	1
H-atom treatment	H-atom parameters constrained

$\Delta\rho_{max}$, $\Delta\rho_{min}$ (e Å^{-3})	0.19, −0.18
Absolute structure	Refined as an inversion twin.
Absolute structure parameter	−1 (6)

Computer programs: Bruker *APEX3* software, *SAINT* V8.38A integration software, *SORTAV* (Blessing, 1995), *SHELXT* 2018/2 (Sheldrick, 2018), *SHELXL2025*/1 (Sheldrick, 2025), *ORTEP* for Windows (Farrugia, 2012), *WinGX* publication routines (Farrugia, 2012).

Table 2

Hydrogen-bond geometry (Å, º)

D—H···*A*	*D*—H	H···*A*	*D*···*A*	*D*—H···*A*
C13—H13···O1^{i}	0.98	2.57	3.392 (14)	142

Symmetry code: (i) $-x+1$, $y-1/2$, $-z+3/2$.

Acknowledgements

Funding information

References

Figure 1

1 supporting information

2 ′ Photooxidative molecular rearrangement of vouacapane diterpenes generates 3 proapoptotic spirocaesalmins ′

4 Computing details

5 Data collection: Bruker *APEX3* software; cell refinement: *SAINT* V8.38A integration software; data reduction: *SORTAV* 6 (Blessing, 1995); program(s) used to solve structure: *SHELXT* 2018/2 (Sheldrick, 2018); program(s) used to refine 7 structure: *SHELXL2025*/1 (Sheldrick, 2025); molecular graphics: *ORTEP* for Windows (Farrugia, 2012); software used 8 to prepare material for publication: *WinGX* publication routines (Farrugia, 2012).

9 (shelx)

10 *Crystal data*

11	$C_{20}H_{28}O_4$	$F(000) = 720$
12	$M_r = 332.42$	$D_x = 1.278$ Mg m^{-3}
13	Orthorhombic, $P2_12_12_1$	Mo $K\alpha$ radiation, $\lambda = 0.71073$ Å
14	Hall symbol: P 2ac 2ab	Cell parameters from 719 reflections
15	$a = 7.303$ (3) Å	$\theta = 2.6$–18.0°
16	$b = 11.389$ (5) Å	$\mu = 0.09$ mm^{-1}
17	$c = 20.777$ (9) Å	$T = 295$ K
18	$V = 1728.2$ (13) Å^3	Plate, colourless
19	$Z = 4$	0.15 × 0.12 × 0.02 mm

20 *Data collection*

21	Bruker D8 VENTURE diffractometer	33738 measured reflections<br>1846 independent reflections
22	Radiation source: microfocus sealed tube	1151 reflections with $I > 2\sigma(I)$
23	Multilayer mirror monochromator	$R_{int} = 0.280$
24	φ or ω oscillation scans	$\theta_{max} = 21.0°$, $\theta_{min} = 2.0°$
25	Absorption correction: multi-scan *SADABS2016*/2 - Bruker AXS area detector scaling and absorption correcti	$h = -7 \rightarrow 7$<br>$k = -11 \rightarrow 11$<br>$l = -20 \rightarrow 20$
26	$T_{min} = 0.53$, $T_{max} = 0.745$	

27 *Refinement*

28	Refinement on F^2	H-atom parameters constrained
29	Least-squares matrix: full	$w = 1/[\sigma^2(F_o^2) + (0.0752P)^2 + 0.5911P]$ where $P = (F_o^2 + 2F_c^2)/3$
30	$R[F^2 > 2\sigma(F^2)] = 0.066$	
31	$wR(F^2) = 0.164$	$(\Delta/\sigma)_{max} < 0.001$
32	$S = 1.08$	$\Delta\rho_{max} = 0.19$ e Å^{-3}
33	1846 reflections	$\Delta\rho_{min} = -0.18$ e Å^{-3}
34	223 parameters	Extinction correction: *SHELXL2025*/1 (Sheldrick 2025), $Fc^* = kFc[1+0.001xFc^2\lambda^3/\sin(2\theta)]^{-1/4}$
35	1 restraint	
36	0 constraints	Extinction coefficient: 0.014 (4)
37	Hydrogen site location: inferred from neighbouring sites	Absolute structure: Refined as an inversion twin.<br>Absolute structure parameter: −1 (6)

Special details

Geometry. All e.s.d.'s (except the e.s.d. in the dihedral angle between two l.s. planes) are estimated using the full covariance matrix. The cell e.s.d.'s are taken into account individually in the estimation of e.s.d.'s in distances, angles and torsion angles; correlations between e.s.d.'s in cell parameters are only used when they are defined by crystal symmetry. An approximate (isotropic) treatment of cell e.s.d.'s is used for estimating e.s.d.'s involving l.s. planes.

Refinement. Refined as a 2-component inversion twin.

Fractional atomic coordinates and isotropic or equivalent isotropic displacement parameters (Å²)

	x	*y*	*z*	U_{iso}*/U_{eq}
O2	0.5691 (11)	−0.0593 (6)	0.6393 (4)	0.080 (2)
O1	0.0737 (10)	0.5095 (7)	0.7357 (3)	0.085 (2)
C1	0.5338 (14)	0.5381 (9)	0.5747 (4)	0.063 (3)
H1A	0.481138	0.509224	0.534819	0.075*
H1B	0.661672	0.515218	0.575359	0.075*
C2	0.5221 (16)	0.6727 (9)	0.5751 (5)	0.074 (3)
H2A	0.585036	0.703355	0.612592	0.089*
H2B	0.581269	0.704005	0.536956	0.089*
C20	0.5495 (12)	0.4975 (8)	0.6924 (4)	0.066 (3)
H20A	0.483181	0.466234	0.728446	0.098*
H20B	0.571123	0.579804	0.699091	0.098*
H20C	0.664588	0.457438	0.6884	0.098*
C19	0.0145 (15)	0.6928 (8)	0.6240 (5)	0.086 (4)
H19A	−0.055149	0.667707	0.660715	0.129*
H19B	−0.027974	0.65236	0.586344	0.129*
H19C	−0.000546	0.775891	0.618197	0.129*
C3	0.3233 (17)	0.7102 (9)	0.5764 (5)	0.073 (3)
H3A	0.263633	0.682004	0.537642	0.087*
H3B	0.317634	0.795256	0.57597	0.087*
C18	0.2785 (15)	0.7320 (10)	0.6950 (5)	0.077 (3)
H18A	0.409323	0.728109	0.698827	0.115*
H18B	0.223027	0.697513	0.732346	0.115*
H18C	0.241148	0.812571	0.691426	0.115*
C17	0.1562 (13)	0.1087 (9)	0.6041 (5)	0.083 (4)
H17A	0.180829	0.030637	0.589007	0.125*
H17B	0.137282	0.159859	0.568006	0.125*
H17C	0.048275	0.108023	0.630511	0.125*
O4	0.4223 (11)	0.1426 (8)	0.4960 (4)	0.095 (3)
C4	0.2179 (14)	0.6649 (9)	0.6349 (5)	0.060 (3)
C16	0.6913 (18)	−0.0223 (11)	0.5927 (7)	0.084 (4)
H16	0.817106	−0.052176	0.593566	0.125*
O3	0.6105 (12)	0.0038 (8)	0.5335 (4)	0.088 (2)
C15	0.5004 (17)	0.1019 (12)	0.5410 (6)	0.072 (3)
C8	0.3070 (13)	0.2802 (9)	0.6654 (5)	0.055 (3)
H8	0.374338	0.286095	0.706027	0.066*
C7	0.1186 (14)	0.3337 (9)	0.6767 (5)	0.067 (3)
H7A	0.041055	0.319216	0.639577	0.08*
H7B	0.061975	0.297296	0.713884	0.08*
C10	0.4369 (12)	0.4802 (8)	0.6305 (4)	0.048 (2)
C9	0.4142 (12)	0.3493 (8)	0.6156 (4)	0.053 (3)
H9	0.346447	0.343739	0.574983	0.063*
C6	0.1364 (13)	0.4646 (10)	0.6876 (5)	0.062 (3)

C5	0.2388 (12)	0.5307 (8)	0.6361 (5)	0.052 (3)
H5	0.179741	0.505268	0.596083	0.063*
C11	0.5848 (14)	0.2724 (8)	0.6086 (5)	0.063 (3)
H11A	0.669066	0.285243	0.643975	0.075*
H11B	0.647413	0.288376	0.568359	0.075*
C14	0.3185 (14)	0.1526 (9)	0.6436 (5)	0.062 (3)
H14	0.325545	0.103889	0.682433	0.074*
C13	0.6351 (15)	0.0618 (10)	0.6404 (5)	0.067 (3)
H13	0.720606	0.087949	0.673771	0.081*
C12	0.5082 (13)	0.1459 (9)	0.6097 (5)	0.054 (3)

Atomic displacement parameters (Å^2)

	U^{11}	U^{22}	U^{33}	U^{12}	U^{13}	U^{23}
O2	0.073 (6)	0.061 (5)	0.105 (6)	−0.008 (4)	0.001 (5)	0.012 (4)
O1	0.078 (5)	0.098 (6)	0.079 (5)	−0.011 (5)	0.023 (4)	−0.016 (5)
C1	0.051 (7)	0.071 (8)	0.066 (7)	−0.009 (6)	−0.001 (6)	0.006 (6)
C2	0.084 (10)	0.065 (9)	0.074 (8)	0.001 (7)	0.019 (7)	0.008 (6)
C20	0.045 (6)	0.073 (7)	0.078 (7)	−0.003 (6)	0.000 (5)	−0.003 (6)
C19	0.069 (8)	0.071 (8)	0.119 (10)	0.019 (6)	−0.019 (7)	−0.013 (7)
C3	0.079 (9)	0.059 (7)	0.081 (8)	0.004 (7)	−0.006 (7)	−0.006 (6)
C18	0.077 (8)	0.075 (8)	0.078 (8)	0.004 (7)	−0.002 (7)	−0.007 (7)
C17	0.052 (7)	0.077 (8)	0.121 (9)	−0.012 (6)	0.001 (7)	−0.019 (7)
O4	0.085 (6)	0.124 (7)	0.075 (6)	0.001 (6)	−0.010 (5)	0.002 (5)
C4	0.049 (7)	0.063 (8)	0.068 (7)	0.008 (6)	−0.002 (6)	−0.006 (7)
C16	0.072 (9)	0.081 (10)	0.098 (10)	0.008 (8)	−0.018 (8)	0.003 (9)
O3	0.089 (6)	0.092 (6)	0.083 (6)	0.022 (5)	0.007 (5)	−0.021 (5)
C15	0.054 (8)	0.090 (10)	0.072 (10)	−0.015 (8)	0.001 (7)	0.006 (8)
C8	0.039 (6)	0.062 (7)	0.065 (7)	0.007 (6)	0.008 (6)	0.003 (6)
C7	0.060 (8)	0.061 (8)	0.079 (8)	−0.004 (6)	0.013 (6)	−0.004 (6)
C10	0.041 (6)	0.053 (6)	0.050 (6)	−0.007 (5)	0.002 (5)	0.003 (5)
C9	0.038 (6)	0.067 (7)	0.053 (6)	0.001 (6)	0.001 (5)	0.000 (6)
C6	0.042 (7)	0.088 (10)	0.058 (8)	0.002 (6)	0.003 (6)	0.003 (7)
C5	0.040 (6)	0.062 (8)	0.055 (7)	−0.001 (5)	−0.007 (5)	−0.001 (6)
C11	0.052 (7)	0.064 (7)	0.071 (7)	−0.008 (6)	0.013 (6)	0.009 (6)
C14	0.056 (7)	0.055 (7)	0.074 (7)	−0.001 (6)	0.011 (6)	0.010 (6)
C13	0.067 (8)	0.072 (8)	0.064 (7)	−0.003 (7)	−0.003 (6)	−0.010 (7)
C12	0.051 (6)	0.056 (7)	0.056 (7)	0.000 (6)	0.000 (6)	0.001 (6)

Geometric parameters (Å, º)

O2—C16	1.382 (13)	C17—H17C	0.96
O2—C13	1.462 (12)	O4—C15	1.190 (11)
O1—C6	1.213 (10)	C4—C5	1.537 (12)
C1—C10	1.511 (11)	C16—O3	1.396 (12)
C1—C2	1.535 (12)	C16—C13	1.438 (14)
C1—H1A	0.97	C16—H16	0.98
C1—H1B	0.97	O3—C15	1.384 (13)
C2—C3	1.514 (15)	C15—C12	1.515 (14)
C2—H2A	0.97	C8—C9	1.517 (11)
C2—H2B	0.97	C8—C7	1.523 (13)
C20—C10	1.540 (11)	C8—C14	1.525 (12)

C20—H20A	0.96	C8—H8	0.98
C20—H20B	0.96	C7—C6	1.514 (13)
C20—H20C	0.96	C7—H7A	0.97
C19—C4	1.536 (14)	C7—H7B	0.97
C19—H19A	0.96	C10—C9	1.531 (12)
C19—H19B	0.96	C10—C5	1.561 (12)
C19—H19C	0.96	C9—C11	1.530 (12)
C3—C4	1.529 (14)	C9—H9	0.98
C3—H3A	0.97	C6—C5	1.508 (13)
C3—H3B	0.97	C5—H5	0.98
C18—C4	1.528 (13)	C11—C12	1.546 (13)
C18—H18A	0.96	C11—H11A	0.97
C18—H18B	0.96	C11—H11B	0.97
C18—H18C	0.96	C14—C12	1.556 (13)
C17—C14	1.526 (13)	C14—H14	0.98
C17—H17A	0.96	C13—C12	1.477 (13)
C17—H17B	0.96	C13—H13	0.98

C16—O2—C13	60.7 (7)	O3—C15—C12	110.6 (10)
C10—C1—C2	113.9 (8)	C9—C8—C7	111.3 (8)
C10—C1—H1A	108.8	C9—C8—C14	105.3 (7)
C2—C1—H1A	108.8	C7—C8—C14	118.5 (9)
C10—C1—H1B	108.8	C9—C8—H8	107
C2—C1—H1B	108.8	C7—C8—H8	107
H1A—C1—H1B	107.7	C14—C8—H8	107
C3—C2—C1	109.6 (10)	C6—C7—C8	109.8 (9)
C3—C2—H2A	109.8	C6—C7—H7A	109.7
C1—C2—H2A	109.8	C8—C7—H7A	109.7
C3—C2—H2B	109.8	C6—C7—H7B	109.7
C1—C2—H2B	109.8	C8—C7—H7B	109.7
H2A—C2—H2B	108.2	H7A—C7—H7B	108.2
C10—C20—H20A	109.5	C1—C10—C9	108.7 (8)
C10—C20—H20B	109.5	C1—C10—C20	109.6 (7)
H20A—C20—H20B	109.5	C9—C10—C20	110.6 (7)
C10—C20—H20C	109.5	C1—C10—C5	109.2 (8)
H20A—C20—H20C	109.5	C9—C10—C5	105.9 (8)
H20B—C20—H20C	109.5	C20—C10—C5	112.7 (7)
C4—C19—H19A	109.5	C8—C9—C11	100.8 (7)
C4—C19—H19B	109.5	C8—C9—C10	115.0 (8)
H19A—C19—H19B	109.5	C11—C9—C10	119.2 (8)
C4—C19—H19C	109.5	C8—C9—H9	107
H19A—C19—H19C	109.5	C11—C9—H9	107
H19B—C19—H19C	109.5	C10—C9—H9	107
C2—C3—C4	113.7 (9)	O1—C6—C5	124.2 (11)
C2—C3—H3A	108.8	O1—C6—C7	120.5 (10)
C4—C3—H3A	108.8	C5—C6—C7	115.3 (10)
C2—C3—H3B	108.8	C6—C5—C4	117.3 (9)
C4—C3—H3B	108.8	C6—C5—C10	109.2 (8)
H3A—C3—H3B	107.7	C4—C5—C10	117.2 (8)
C4—C18—H18A	109.5	C6—C5—H5	103.7
C4—C18—H18B	109.5	C4—C5—H5	103.7
H18A—C18—H18B	109.5	C10—C5—H5	103.7

C4—C18—H18C	109.5	C9—C11—C12	103.7 (8)
H18A—C18—H18C	109.5	C9—C11—H11A	111
H18B—C18—H18C	109.5	C12—C11—H11A	111
C14—C17—H17A	109.5	C9—C11—H11B	111
C14—C17—H17B	109.5	C12—C11—H11B	111
H17A—C17—H17B	109.5	H11A—C11—H11B	109
C14—C17—H17C	109.5	C8—C14—C17	115.4 (9)
H17A—C17—H17C	109.5	C8—C14—C12	103.3 (8)
H17B—C17—H17C	109.5	C17—C14—C12	115.6 (8)
C18—C4—C3	109.6 (9)	C8—C14—H14	107.4
C18—C4—C19	107.3 (8)	C17—C14—H14	107.4
C3—C4—C19	107.4 (9)	C12—C14—H14	107.4
C18—C4—C5	117.1 (9)	C16—C13—O2	56.9 (7)
C3—C4—C5	107.3 (8)	C16—C13—C12	108.3 (9)
C19—C4—C5	107.7 (9)	O2—C13—C12	113.5 (9)
O2—C16—O3	114.1 (10)	C16—C13—H13	120.5
O2—C16—C13	62.4 (8)	O2—C13—H13	120.5
O3—C16—C13	110.1 (10)	C12—C13—H13	120.5
O2—C16—H16	119.1	C13—C12—C15	102.4 (9)
O3—C16—H16	119.1	C13—C12—C11	112.6 (8)
C13—C16—H16	119.1	C15—C12—C11	107.9 (8)
C15—O3—C16	108.6 (9)	C13—C12—C14	113.3 (8)
O4—C15—O3	120.3 (11)	C15—C12—C14	114.2 (8)
O4—C15—C12	129.0 (13)	C11—C12—C14	106.5 (8)

C10—C1—C2—C3	−56.5 (12)	C1—C10—C5—C6	174.6 (8)
C1—C2—C3—C4	59.4 (12)	C9—C10—C5—C6	57.7 (10)
C2—C3—C4—C18	72.7 (11)	C20—C10—C5—C6	−63.3 (11)
C2—C3—C4—C19	−171.1 (9)	C1—C10—C5—C4	−49.0 (12)
C2—C3—C4—C5	−55.5 (12)	C9—C10—C5—C4	−165.8 (9)
C13—O2—C16—O3	−101.0 (11)	C20—C10—C5—C4	73.1 (11)
O2—C16—O3—C15	67.0 (12)	C8—C9—C11—C12	40.5 (10)
C13—C16—O3—C15	−0.9 (14)	C10—C9—C11—C12	167.4 (8)
C16—O3—C15—O4	177.5 (11)	C9—C8—C14—C17	−96.0 (10)
C16—O3—C15—C12	0.5 (12)	C7—C8—C14—C17	29.3 (13)
C9—C8—C7—C6	−48.9 (12)	C9—C8—C14—C12	31.1 (10)
C14—C8—C7—C6	−171.2 (8)	C7—C8—C14—C12	156.4 (9)
C2—C1—C10—C9	165.2 (9)	O3—C16—C13—O2	107.5 (11)
C2—C1—C10—C20	−73.8 (10)	O2—C16—C13—C12	−106.6 (9)
C2—C1—C10—C5	50.1 (11)	O3—C16—C13—C12	0.9 (14)
C7—C8—C9—C11	−174.7 (9)	C16—O2—C13—C12	97.2 (10)
C14—C8—C9—C11	−45.1 (10)	C16—C13—C12—C15	−0.5 (12)
C7—C8—C9—C10	55.7 (11)	O2—C13—C12—C15	−61.6 (11)
C14—C8—C9—C10	−174.7 (8)	C16—C13—C12—C11	−116.2 (11)
C1—C10—C9—C8	−175.9 (8)	O2—C13—C12—C11	−177.3 (9)
C20—C10—C9—C8	63.7 (10)	C16—C13—C12—C14	122.9 (10)
C5—C10—C9—C8	−58.7 (10)	O2—C13—C12—C14	61.8 (12)
C1—C10—C9—C11	64.2 (11)	O4—C15—C12—C13	−176.6 (11)
C20—C10—C9—C11	−56.2 (11)	O3—C15—C12—C13	0.0 (10)
C5—C10—C9—C11	−178.6 (8)	O4—C15—C12—C11	−57.6 (14)
C8—C7—C6—O1	−125.0 (10)	O3—C15—C12—C11	119.0 (9)
C8—C7—C6—C5	53.7 (11)	O4—C15—C12—C14	60.6 (15)

O1—C6—C5—C4	−17.1 (15)	O3—C15—C12—C14	−122.8 (9)
C7—C6—C5—C4	164.2 (9)	C9—C11—C12—C13	−146.7 (8)
O1—C6—C5—C10	119.3 (10)	C9—C11—C12—C15	101.0 (10)
C7—C6—C5—C10	−59.4 (11)	C9—C11—C12—C14	−22.0 (10)
C18—C4—C5—C6	60.0 (12)	C8—C14—C12—C13	119.1 (9)
C3—C4—C5—C6	−176.4 (8)	C17—C14—C12—C13	−113.9 (10)
C19—C4—C5—C6	−61.0 (12)	C8—C14—C12—C15	−124.2 (10)
C18—C4—C5—C10	−72.9 (12)	C17—C14—C12—C15	2.8 (13)
C3—C4—C5—C10	50.7 (12)	C8—C14—C12—C11	−5.2 (10)
C19—C4—C5—C10	166.1 (8)	C17—C14—C12—C11	121.8 (10)

Hydrogen-bond geometry (Å, º)

D—H···*A*	*D*—H	H···*A*	*D*···*A*	*D*—H···*A*
C13—H13···O1^{i}	0.98	2.57	3.392 (14)	142

Symmetry code: (i) $-x+1$, $y-1/2$, $-z+3/2$.
